# Supplementary material for: Understory plants evade shading in a temperate deciduous forest amid climate variability by shifting phenology in synchrony with canopy trees
Source: PLoS One. 2024 Jun 26;19(6):e0306023. doi: 10.1371/journal.pone.0306023 (PMC11207122; doi:10.1371/journal.pone.0306023)
Supplement: S1 Table — These basal areas values were normalized so that they sum to 100%, which in our analysis has the effect of treating other tree species (representing <4% of basal area) as though they have phenology that is equivalent to the overall average of the observed species. There was minimal change in species’ basal area from 1995–2015. Relative basal areas changed dramatically when the emerald ash borer killed virtually all ash trees in Trelease Woods between 2015–2018. Note that three Ohio buckeye trees were added to the census in 1996, hence the apparent increase from 1995 to 2022. (DOCX) [file pone.0306023.s001.docx]

Supporting Information 1 for Augspurger CK, Salk CF. Understory plants reduce light loss in a temperate deciduous forest amid climate variability by shifting phenology in synchrony with canopy trees. PLoS One. In review.

Supporting Information 1. Species of canopy trees used in this analysis, the number of living trees of each species in the census in 1995 and 2022, and the relative basal areas of these species in Trelease Woods at two times during the study period. These basal areas values were normalized so that they sum to 100%, which in our analysis has the effect of treating other tree species (representing <4% of basal area) as though they have phenology that is equivalent to the overall average of the observed species. There was minimal change in species' basal area from 1995-2015. Relative basal areas changed dramatically when the emerald ash borer killed virtually all ash trees in Trelease Woods between 2015-2018. Note that three Ohio buckeye trees were added to the census in 1996, hence the apparent increase from 1995 to 2022.

| Species | Common name | Living trees in 1995 | Living trees in 2022 | 2005 Basal area (%) * | 2022 Basal area (%) |
| --- | --- | --- | --- | --- | --- |
| *Acer saccharum* Marsh. | sugar maple | 14 | 11 | 19.58 | 25.34 |
| *Aesculus glabra* Willd. | Ohio buckeye | 13 | 14 | 4.58 | 5.92 |
| *Carya cordiformis* (Wangenh.) K.Koch | bitternut hickory | 5 | 3 | 0.55 | 0.71 |
| *Carya laciniosa* (Mill.) K.Koch | shellbark hickory | 15 | 14 | 1.61 | 2.09 |
| *Celtis occidentalis* L. | common hackberry | 15 | 14 | 17.18 | 22.23 |
| *Fraxinus americana* L. | white ash | 10 | 0 | 17.96 | 0 |
| *Fraxinus pennsylvanica* Marshall | green ash | 3 | 0 | 1.25 | 0 |
| *Fraxinus quadrangulata* Michx. | blue ash | 1 | 1 | 1.78 | 2.3 |
| *Gymnocladus dioicus* (L.) K. Koch | Kentucky coffee tree | 11 | 11 | 0.87 | 1.12 |
| *Juglans nigra* L. | black walnut | 20 | 20 | 6.62 | 8.57 |
| *Quercus macrocarpa* Michx. | bur oak | 15 | 13 | 7.91 | 10.24 |
| *Quercus rubra* L. | northern red oak | 12 | 11 | 5.69 | 7.36 |
| *Tilia americana* L. | American basswood | 16 | 16 | 8.56 | 11.07 |
| *Ulmus americana* L. | American elm | 4 | 2 | 1.62 | 2.1 |
| *Ulmus rubra* Muhl. | slippery elm | 16 | 3 | 4.25 | 0.94 |

* Based on a complete census of Trelease Woods in 2005 by J. Edgington. Species’ basal area values for all years are found in Supporting Information 5.
