# Supplementary material for: Understory plants evade shading in a temperate deciduous forest amid climate variability by shifting phenology in synchrony with canopy trees
Source: PLoS One. 2024 Jun 26;19(6):e0306023. doi: 10.1371/journal.pone.0306023 (PMC11207122; doi:10.1371/journal.pone.0306023)
Supplement: S2 Table — On a given census date, the stage documented represents the tree crown as a whole, i.e., the dominant condition of buds, shoots or leaves. (DOCX) [file pone.0306023.s002.docx]

Supporting Information 2 for Augspurger CK, Salk CF. Understory plants reduce light loss in a temperate deciduous forest amid climate variability by shifting phenology in synchrony with canopy trees. PLoS One. In review.

Supporting Information 2. Description of events within each phenophase used to quantify light interception. On a given census date, the stage documented represents the tree crown as a whole, i.e., the dominant condition of buds, shoots or leaves.

| **Code** | **Phase** | **Stage** | **Description of Stage** |
| --- | --- | --- | --- |
| B | Bud Burst/  Leafing Out | B1 | Bud burst; first leaf tips visible beyond bud scales |
|  |  | B2 | Leaf/shoot emergence well beyond bud scales, but  leaves not yet unfolding and entire petiole not visible |
|  |  | B3 | Individual leaf blade and petiole visible; leaf unfolded but not expanded |
| F | Leaf Expansion | F1 | Leaf 1/3 of final size |
|  |  | F2 | Leaf 2/3 of final size |
|  |  | F3 | Leaf final size but not necessarily final green color |
| D | Leaf Drop | D1 | 1/3 of leaves fallen |
|  |  | D2 | 2/3 of leaves fallen |
|  |  | D3 | All leaves fallen |
