# Supplementary material for: Understory plants evade shading in a temperate deciduous forest amid climate variability by shifting phenology in synchrony with canopy trees
Source: PLoS One. 2024 Jun 26;19(6):e0306023. doi: 10.1371/journal.pone.0306023 (PMC11207122; doi:10.1371/journal.pone.0306023)
Supplement: S3 Table — Also shown is the phenological seasonality of each species. (DOCX) [file pone.0306023.s003.docx]

Supporting Information 3 for Augspurger CK, Salk CF. Understory plants reduce light loss in a temperate deciduous forest amid climate variability by shifting phenology in synchrony with canopy trees. PLoS One. In review.

Supporting Information 3. Herb species-cohort combinations (referred to as “species” in the main text) observed from 1995-2017 in Trelease Woods. Also shown is the phenological seasonality of each species.

| **Species** | **Phenological Seasonality** |
| --- | --- |
| *Allium canadense* L. | Spring Ephemeral |
| *Allium tricoccum* Aiton | Spring Ephemeral |
| *Aplectrum hyemale* (Muhl. ex Willd.) Torr. | Winter Perennial |
| *Arisaema dracontium* (L.) Schott | Spring-Summer |
| *Arisaema triphyllum* (L.) Schott | Spring-Summer |
| *Asarum canadense* L. | Spring-Autumn |
| *Cardamine concatenata* (Michx.) Sw. | Spring Ephemeral |
| *Cardamine douglassii* Britton | Winter Annual |
| *Carex albursina* Sheldon | Quasi-evergreen |
| *Claytonia virginica* L. | Spring Ephemeral |
| *Cryptotaenia canadensis* (L.) DC. | Spring-Autumn |
| *Cystopteris protrusa* (Weath.) Blasdell | Spring-Summer |
| *Dicentra cucullaria* (L.) Bernh. | Spring Ephemeral |
| *Erythronium albidum* Nutt. | Spring Ephemeral |
| *Floerkea proserpinacoides* Wild. | Spring Ephemeral |
| *Geranium maculatum* L. | Spring-Summer |
| *Hydrophyllum appendiculatum* Michx.* yr 1a | Spring-Summer |
| *Hydrophyllum appendiculatum* Michx.* yr 2 | Spring-Summer |
| *Hydrophyllum virginianum* L. ** I | Spring-Summer |
| *Hydrophyllum virginianum* L. ** II | Autumn-Winter |
| *Laportea canadensis* (L.) Weddell | Spring-Autumn |
| *Lilium philadelphicum* L. | Spring-Summer |
| *Mertensia virginica* (L.) Pers. ex Link | Spring Ephemeral |
| *Phlox divaricata* L. | Quasi-evergreen |
| *Pilea pumila* (L.) A. Gray | Spring-Autumn |
| *Podophyllum peltatum* L*.* | Spring-Summer |
| *Polygonum virginianum L.* | Spring-Autumn |
| *Prenanthes crepidinea* Michx. | Spring Ephemeral |
| *Ranunculus hispidus var. nitidus (*Chapm.) T. Duncan | Quasi-evergreen |
| *Sanicula odorata* (Raf.) K.M. Pryer & L.R. Phillippe *** Cohort 1 | Spring-Autumn |
| *Trillium recurvatum* Beck | Spring Ephemeral |
| *Viola pubescens* Aiton | Spring-Autumn |
| *Viola sororia* Willd. | Spring-Autumn |

* In first year has spring-summer (yr1a) and autumn (yr1b) non-reproductive cohorts and one reproductive cohort in the second year (yr2); cohort 1b was not used due to insufficient data.

** Has 2 cohorts: spring through summer (I) and autumn into winter (II); both were used.

*** Has 2 cohorts: spring into autumn (Cohort 1) and autumn into winter (Cohort 2); cohort 2 had insufficient data.
