# Supplementary material for: Understory plants evade shading in a temperate deciduous forest amid climate variability by shifting phenology in synchrony with canopy trees
Source: PLoS One. 2024 Jun 26;19(6):e0306023. doi: 10.1371/journal.pone.0306023 (PMC11207122; doi:10.1371/journal.pone.0306023)
Supplement: S1 File — (DOCX) [file pone.0306023.s005.docx]

Supporting Information 4 for Augspurger CK, Salk CF. Understory plants reduce light loss in a temperate deciduous forest amid climate variability by shifting phenology in synchrony with canopy trees. PLoS One. In review.

Supporting Information 4. Details of the herb data cleaning process.

This document explains details of how the herb phenology dataset (Herb Data.csv) was cleaned and processed. In this document Em = shoot emergence (stems, cotyledons or leaves depending on species), Ex = full leaf expansion, Se = leaf senescence and Do = plant dormancy.

**Fill in missing datapoints or delete existing datapoints according to the following rules**:

1) If only one of the four phenological events (Em, Ex, Se, Do) has a date, do not use any data from that year/plot/species combination. This is done near the very end of the procedure for computational reasons.

2) Missing values of either leaf emergence (Em) or full expansion (Ex), but not both:

2a) If >5 plots in the same year have this species present with no missing values of either Em or Ex, use the average difference between Ex and Em to calculate the missing value by adding or subtracting this value from the non-missing value.

2b) If (2a) is not satisfied, but the plot in question has complete data on Em and Ex for at least 12 years, use the average Ex-Em difference in the same way as in (2a).

2c) If neither (2a) nor (2b) is met, then use the average Ex-Em difference over all plots and years.

3) Missing values of either senescence (Se) or dormancy (Do), but not both, are processed in the same way as missing values in Em or Ex.

**Special rules for certain species. These rules override the previous rules**:

1) Quasi-evergreen species (*Carex albursina*, *Carex blanda*, *Carex grayi*) and *Polygonum virginianum*:

1a) Use the overall mean date of senescence as the value for all missing Se values.

1b) Use the next year’s emergence date for the same plot as the dormancy date.

2) *Phlox divaricata*: All the Se and Do dates are set to 335 (Dec 1).

3) *Cystopteris protrusa*: Se is always 30 days before observed Do.

4) *Cardamine douglassii* cotyledon missing values:

4a) If >=6 plots have values for a given year, use the average of those values to fill in missing values.

4b) If <6 plots have values in a given year, use the plot-level average across years if >=12 years have values.

4c) Otherwise, use the mean value across all years/plots

**Final cleaning steps:**

Avoid senescence dates happening earlier than expansion: If the Se date calculated by the above rules is earlier than the Em date, then set the Se date equal to the date of Em.

Drop all rows containing any remaining NA values. (This is Rule 1 from above)

Round all values to the nearest integer; some decimal points will have resulted from taking average dates.

**Estimation of understory plants’ relative leaf area**

Next, for all species except *Cardamine douglassii*, we calculated the leaf status on each day for which a plant had leaves displayed in a given plot:

1) Leaf status increased linearly from 0 to 1 from Em to Ex.

2) Leaf status was 1 between Ex and Se

3) Leaf status decreased linearly to 0 from Se to Do

*Cardamine douglassii* is an annual that germinates in the late fall, and has partially expanded cotyledons that begin growing to their full size around January 1. The cotyledons reach their full size around the time the shoot begins to bolt, which we called Em for this species. We used the following rules:

1) From the date of cotyledon emergence to January 1, leaf status was assigned a value of .05. This value was determined by finding the average ratio between the approximate size of newly emerged cotyledons (1 cm^2^) and the total leaf area of a mature *Cardamine douglassii* plant. Leaf areas were measured for 10 plants in spring/winter 2019 and averaged.

2) From Jan 1 to Em, leaf status increased linearly from .05 to .29 as the cotyledon grew from its initial size to its final size. The value of .29 was determined by measuring fully expanded leaves and cotyledons for the same 10 plants in the above note and taking the average ratio between full cotyledon size and total leaf area of fruiting plants.

3) From Em to Ex, leaf status increased linearly from .29 to 1.

4) From Ex to Se, leaf status was 1.

5) From Se to Do, leaf status decreased linearly from 1 to 0.
