# Supplementary material for: Understory plants evade shading in a temperate deciduous forest amid climate variability by shifting phenology in synchrony with canopy trees
Source: PLoS One. 2024 Jun 26;19(6):e0306023. doi: 10.1371/journal.pone.0306023 (PMC11207122; doi:10.1371/journal.pone.0306023)
Supplement: S2 File — (DOCX) [file pone.0306023.s006.docx]

Supporting Information 6 for Augspurger CK, Salk CF. Understory plants reduce light loss in a temperate deciduous forest amid climate variability by shifting phenology in synchrony with canopy trees. PLoS One. In review.

Supporting Information 6. Graphical explanation and full equations for the light-partitioning process applied in this study.

This document gives explicit equations for the light partitioning procedure explained in Methods Section 5 of the main text. The symbols L, U, T, C and R used in this document represent variables that are explained in more detail in Methods Section 4 of the main text:

*L* = light intercepted by a plant

*U* = leaf phenological status of an understory plant

*T* = an indicator (0 or 1) of whether the temperature is warm enough for photosynthesis

*C* = canopy light transmittance

*R* = solar photosynthetically active radiation

The figure below is a schematic depiction of the light partitioning calculations used in this study. In this figure, we depict only two of the four variables, the date-specific understory phenology (*U_d,y_* - the percent of possible leaf area an understory plant has on a given day *d* in year *y*) and canopy light transmittance (*C_d,y_* – the percent of light transmitted by the canopy on a given day, which deviates from the long-term average, depending on canopy phenology; - see Figure 2 in the main text). We omit temperature and solar radiation so that this approach can be visualized in two dimensions. The blue square in all four diagrams represents *L̅_d,_* the usable light interception on day *d* when *U_d,y_ = U̅_d_* and *C_d,y_ = C̅_d_* (i.e., both understory phenology and canopy light transmittance have average values). For simplicity, it is labeled *L̅_d_* in only panel A of the figure. The daily deviations from the long-term date-specific averages of *U̅_d_* and *C̅_d_* are *δU= U_d,y_-U̅_d_* and *δC=C_d,y_-C̅_d_*. The upper left diagram shows the simplest case, where both *δU* and *δC* are positive (i.e., the understory plant has leafed early and the canopy has leafed late). Note that 'positive' reflects an understory plant's perspective; this plant has grown its leaves early and the canopy has grown its leaves late, both of which mean the understory plant will intercept more light. Thus, both *Lu_d,y_* and *Lc_d,y_*, which are the deviation from *L̅_d_* due to understory phenology and canopy phenology, respectively, are both positive. When *δU*, *δC* or both are negative, the calculation is more complicated. In these cases, we assign a hierarchy such that *U* is more important than *C,* reflecting an understory plant-centered perspective. If it has no leaves, the state of the canopy on this day is of no consequence; the understory plant cannot photosynthesize. In the full calculation with all four variables, the hierarchy is understory phenology (*U*) > cold temperatures (*T*) > canopy phenology (*C*) > solar radiation (*R*). Because *U* is the highest ranking variable, all loss due to *δU<0* is attributed to *Lu_d,y_*. Panel B shows the case where *δU<0* and *δC>0*. Because of the hierarchy among the variables, *δU* is accounted for first (the tan shaded area is a negative contribution to *Lu_d,y_* – the understory plant has grown its leaves late) and then *Lc_d,y_* receives its positive contribution from *δC* (due to a late-leafing canopy). When *δU>0* (i.e. the understory plant has leafed early) and *δC<0* (i.e. the canopy is early, shading the understory more than average; see panel C), the contribution of *δU* to *Lu_d,y_* is first calculated, then the remaining impact of *δC* on *Lc_d,y_* is computed; because the higher-ranking *U* variable first gets credit due to early understory phenology, there is a greater loss due to a negative *δC* canopy phenology. Thus, the dark gray area is a positive contribution to *Lu_d,y_* and a negative contribution to *Lc_d,y_*. When both *δU<0* and *δC<0*, as in panel D, the impact on *Lu_d,y_* is calculated first, and *Lc_d,y_* loses value from only the remaining potential light interception after the impact of *δU* has been subtracted. The total light available to the understory plant is the blue outlined area, plus or minus the areas with colored fill. Note that *U̅_d_* and *C̅_d_* are *δU= U_d,y_-U̅_d_*  and *δC=C_d,y_-C̅_d_*. Panel A shows the simplest case, with *U̅_d_* and *C̅_d_* depicted as being the same size in this diagram (hence the blue outlined area is a square) to avoid implying that one is more important than the other. In reality, *U̅_d_* will typically be bigger in the early spring, and *C̅_d_* bigger in the late spring.


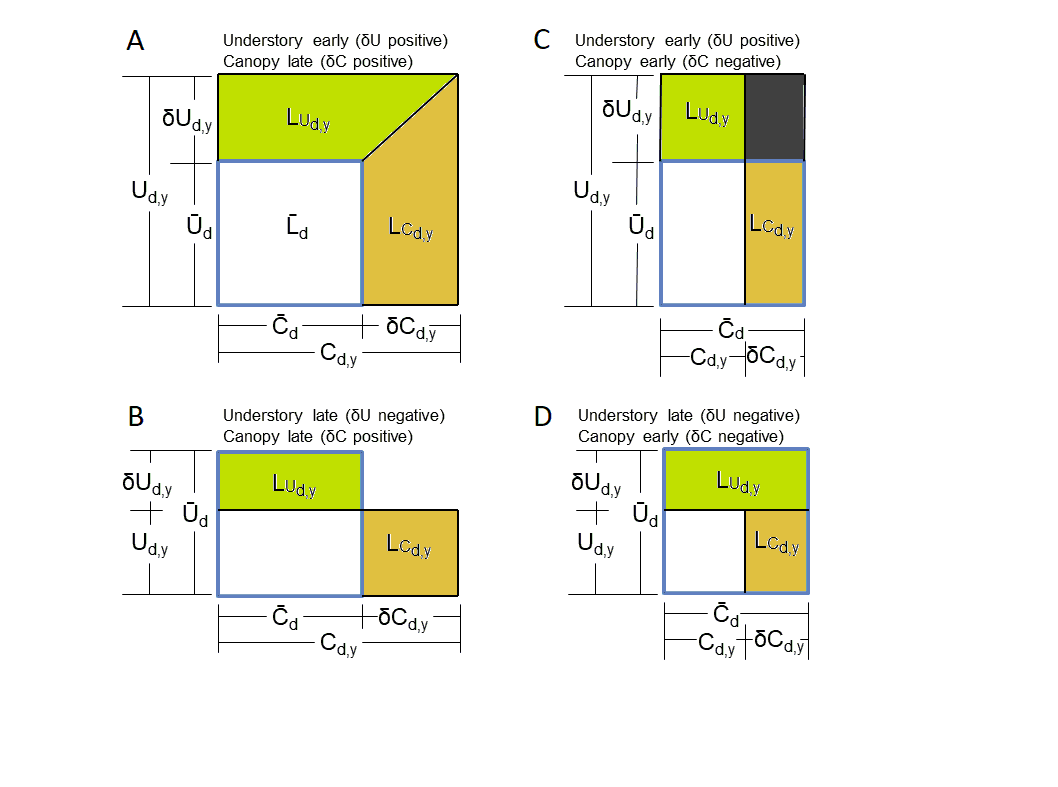


In the following equations, *δLX_d,y_* is the proportion of deviation in light interception that is attributable to variable *X* on day *d* and year *y*, *Xobs_d,y_* is the observed value of variable *X*, and *X̅_d_* is mean phenological status across all years on day *d*. Thus, *δLX_d,y_* = *Xobs_d,y_ - X̅_d_*. The *f^+^()* notation returns 1 if all elements inside the parentheses are positive, and 0 otherwise. *Xmin_d,y_* takes the value of *Xobs_d,y_* or *X̅_d_*, whichever is smaller. A key part of these equations is the terms of the form *(Umin_d,y_ + δU_d,y_ f^+^(δU_d,y_) f^+^(-δT_d,y_))* which allow for the hierarchical partitioning of *δL* described above. The particular example here returns *Umin_d,y_* unless *δU_d,y_* is positive and *δT_d,y_* is negative, in which case it returns the observed value of *U_d,y_*. Note that it is possible for the values of *δLU, δLT* and *δLC* (but not *δLR*) to be bigger than the total value of *δL*; the difference between the bigger values of a partitioned variable and *δL* is offset with negative partitioned values due to other factors.

*δLU_d,y_ = δU_d,y_ T̅_d_ C̅_d_ R̅_d_ +*

*(1/2) (f^+^(δU_d,y_,δT_d,y_) δU_d,y_ δT_d,y_ C̅_d_ R̅_d_ + f^+^( δU_d,y_,δC_d,y_) δU_d,y_ T̅_d_ δC_d,y_ R̅_d_ + f^+^(δU_d,y_,δR_d,y_) δU_d,y_ T̅_d_ C̅_d_ δR_d,y_) +*

*(1/3) (f^+^(δU_d,y_,δT_d,y_,δC_d,y_) δU_d,y_ δT_d,y_ δC_d,y_ R̅_d_ + f^+^(δU_d,y_,δT_d,y_,δR_d,y_) δU_d,y_ δT_d,y_ C̅_d_ δR_d,y_ + f^+^(δU_d,y_,δC_d,y_,δR_d,y_) δU_d,y_ T̅_d_ δC_d,y_ δR_d,y_) +*

*(1/4) f^+^(δU_d,y_,δT_d,y_,δC_d,y_,δR_d,y_) δU_d,y_ δT_d,y_ δC_d,y_ δR_d,y_*

*δLT_d,y_ = (Umin_d,y_ + δU_d,y_ f^+^(δU_d,y_) f^+^(-δT_d,y_)) δT_d,y_ C̅_d_ R̅_d_ +*

*(1/2) (f^+^(δU_d,y_,δT_d,y_) δU_d,y_ δT_d,y_ C̅_d_ R̅_d_ + f^+^(δT_d,y_,δC_d,y_) U̅ δT_d,y_ δC_d,y_ R̅_d_ + f^+^(δT_d,y_,δR_d,y_) U̅_d_ δT_d,y_ C̅ δR_d,y_) +*

*(1/3) (f^+^(δT_d,y_,δC_d,y_,δR_d,y_) U̅ _d_ δT_d,y_ δC_d,y_ δR_d,y_ + f^+^(δU_d,y_,δT_d,y_,δR_d,y_) δU_d,y_ δT_d,y_ C̅_d_ δR_d,y_ + f^+^(δU_d,y_,δT_d,y_,δC_d,y_) δU_d,y_ δT_d,y_ δC_d,y_ R̅_d_) +*

*(1/4) f^+^(δU_d,y_,δT_d,y_,δC_d,y_,δR_d,y_) δU_d,y_ δT_d,y_ δC_d,y_ δR_d,y_*

*δLC_d,y_ = (Umin_d,y_ + δU_d,y_ f^+^(δU_d,y_) f^+^(-δC_d,y_)) (Tmin_d,y_ + δT_d,y_ f^+^(δT_d,y_) f^+^(-δC_d,y_)) δC_d,y_ R̅_d_ +*

*(1/2) (f^+^(δU_d,y_,δC_d,y_) δU_d,y_ T̅_d_ δC_d,y_ R̅_d_ + f^+^(δT_d,y_,δC_d,y_) U̅_d_ δT_d,y_ δC_d,y_ R̅_d_ + f^+^(δC_d,y_,δR_d,y_) U̅_d_ T̅_d_ δC_d,y_ δR_d,y_) +*

*(1/3) (f^+^(δT_d,y_,δC_d,y_,δR_d,y_) U̅_d_ δT_d,y_ δC_d,y_ δR_d,y_ + f^+^(δU_d,y_,δC_d,y_,δR_d,y_) δU_d,y_ T̅_d_ δC_d,y_ δR_d,y_ + f^+^(δU_d,y_,δT_d,y_,δC_d,y_) δU_d,y_ δT_d,y_ δC_d,y_ R̅_d_) +*

*(1/4) f^+^(δU_d,y_,δT_d,y_,δC_d,y_,δR_d,y_) δU_d,y_ δT_d,y_ δC_d,y_ δR_d,y_*

*δLR_d,y_ = (Umin_d,y_ + δU_d,y_ f^+^(δU_d,y_) f^+^(-δR_d,y_))(Tmin_d,y_ + δT_d,y_ f^+^(δT_d_) f^+^(-δR_d,y_))(Cmin_d,y_ + δC_d,y_ f^+^(δC_d,y_) f^+^(-δR_d,y_)) δR_d,y_ +*

*(1/2) (f^+^(δU_d,y_,δR_d,y_) δU_d,y_ T̅_d_ C̅_d_ δR + f^+^(δT_d,y_,δR_d,y_) U̅_d_ δT_d,y_ C̅_d_ δR_d,y_ + f^+^(δC_d,y_,δR_d,y_) U̅_d_ T̅_d_ δC_d,y_ δR_d,y_) +*

*(1/3) (f^+^(δT_d,y_,δC_d,y_,δR_d,y_) U̅_d_ δT_d,y_ δC_d,y_ δR_d,y_ + f^+^(δU_d,y_,δC_d,y_,δR_d,y_) δU_d,y_ T̅_d_ δC_d,y_ δR_d,y_ + f^+^(δU_d,y_,δT_d,y_,δR_d,y_) δU_d,y_ δT_d,y_ C̅_d_ δR_d,y_) +*

*(1/4) f^+^(δU_d,y_,δT_d,y_,δC_d,y_,δR_d,y_) δU_d,y_ δT_d,y_ δC_d,y_ δR_d,y_*

The remainder of this document shows the specific cases of the above general equations for each of the 16 possible combinations of positive or negative deviation for each of the four contributing variables. The names of the cases [in brackets] indicate whether a particular variable is above or below average; **U^+^** means that *U> U̅* (e.g., *δU>0*), and **U^-^** means the opposite. If a variable has a value of 0, both the + and – cases for that variable will give the same result.

**[U^+^T^+^C^+^R^+^]**

L_U_ = δU T̅ C̅ R̅ +

(1/2) (δU δT C̅ R̅ + δU T̅ δC R̅ + δU T̅ C̅ δR) +

(1/3) (δU δT δC R̅ + δU δT C̅ δR + δU T̅ δC δR) +

(1/4) δU δT δC δR

L_T_ = U̅ δT C̅ R̅ +

(1/2) (δU δT C̅ R̅ + U̅ δT δC R̅ + U̅ δT C̅ δR) +

(1/3) (U̅ δT δC δR + δU δT C̅ δR + δU δT δC R̅) +

(1/4) δU δT δC δR

L_C_ = U̅ T̅ δC R̅ +

(1/2) (δU T̅ δC R̅ + U̅ δT δC R̅ + U̅ T̅ δC δR) +

(1/3) (U̅ δT δC δR + δU T̅ δC δR + δU δT δC R̅) +

(1/4) δU δT δC δR

L_R_ = U̅ T̅ C̅ δR +

(1/2) (δU T̅ C̅ δR + U̅ δT C̅ δR + U̅ T̅ δC δR) +

(1/3) (U̅ δT δC δR + δU T̅ δC δR + δU δT C̅ δR) +

(1/4) δU δT δC δR

**[U^-^T^-^C^-^R^-^]**

L_U_ = δU T̅ C̅ R̅

L_T_ = U δT C̅ R̅

L_C_ = U T δC R̅

L_R_ = U T C δR

**[U^+^T^-^C^-^R^-^]**

L_U_ = δU T̅ C̅ R̅

L_T_ = U δT C̅ R̅

L_C_ = U T δC R̅

L_R_ = U T C δR

**[U^-^T^+^C^-^R^-^]**

L_U_ = δU T̅ C̅ R̅

L_T_ = U δT C̅ R̅

L_C_ = U T δC R̅

L_R_ = U T C δR

**[U^-^T^-^C^+^R^-^]**

L_U_ = δU T̅ C̅ R̅

L_T_ = U δT C̅ R̅

L_C_ = U T δC R̅

L_R_ = U T C δR

**[U^-^T^-^C^-^R^+^]**

L_U_ = δU T̅ C̅ R̅

L_T_ = U δT C̅ R̅

L_C_ = U T δC R̅

L_R_ = U T C δR

**[U^+^T^+^C^-^R^-^]**

L_U_ = δU T̅ C̅ R̅ +

(1/2) δU δT C̅ R̅

L_T_ = U̅ δT C̅ R̅+

(1/2) δU δT C̅ R̅

L_C_ = U T δC R̅

L_R_ = U T C δR

**[U^+^T^-^C^+^R^-^]**

L_U_ = δU T̅ C̅ R̅ +

(1/2) δU T̅ δC R

L_T_ = U δT C̅ R̅

L_C_ = U̅ T δC R̅ +

(1/2) δU T̅ δC R

L_R_ = U T C δR

**[U^+^T^-^C^-^R^+^]**

L_U_ = δU T̅ C̅ R̅ +

(1/2) δU T C δR

L_T_ = U δT C̅ R̅

L_C_ = U T δC R̅

L_R_ = U̅ T C δR +

(1/2) δU T C δR

**[U^-^T^+^C^+^R^-^]**

L_U_ = δU T̅ C̅ R̅

L_T_ = U δT C̅ R̅ +

(1/2) U δT δC R

L_C_ = U T̅ δC R̅ +

(1/2) U δT δC R

L_R_ = U T C δR

**[U^-^T^+^C^-^R^+^]**

L_U_ = δU T̅ C̅ R̅

L_T_ = U δT C̅ R̅ +

(1/2) U δT C δR

L_C_ = U T δC R̅

L_R_ = U T̅ C δR +

(1/2) U δT C δR

**[U^-^T^-^C^+^R^+^]**

L_U_ = δU T̅ C̅ R̅

L_T_ = U δT C̅ R̅

L_C_ = U T δC R̅ +

(1/2) U T δC δR

L_R_ = U T C̅ δR +

(1/2) U T δC δR

**[U^-^T^+^C^+^R^+^]**

L_U_ = δU T̅ C̅ R̅

L_T_ = U δT C̅ R̅ +

(1/2) (U δT C̅ δR + U T̅ δC δR) +

(1/3) (U δT δC)

L_C_ = U T δC R̅ +

(1/2) (U δT δC R̅ +U T̅ δC δR) +

(1/3) (U δT δC δR) +

L_R_ = U T C̅ δR +

(1/2) (U δT C̅ δR + U T̅ δC δR) +

(1/3) (U δT δC δR) +

**[U^+^T^-^C^+^R^+^]**

L_U_ = δU T̅ C̅ R̅ +

(1/2) (δU T̅ δC R̅ + δU T̅ C̅ δR) +

(1/3) (δU T̅ δC δR) +

L_T_ = U δT C̅ R̅

L_C_ = U̅ T δC R̅ +

(1/2) (δU T δC R̅ + U̅ T̅ δC δR) +

(1/3) (δU T δC δR) +

L_R_ = U̅ T C̅ δR +

(1/2) (δU T C̅ δR + U̅ T δC δR) +

(1/3) (δU T δC δR) +

**[U^+^T^+^C^-^R^+^]**

L_U_ = δU T̅ C̅ R̅ +

(1/2) (δU δT C R̅ + δU T̅ C δR) +

(1/3) (δU δT C δR) +

L_T_ = U̅ δT C̅ R̅ +

(1/2) (δU δT C R̅ +U̅ δT C δR) +

(1/3) (δU δT C δR) +

L_C_ = U T δC R̅

L_R_ = U̅ T̅ C δR +

(1/2) (δU T̅ C δR + U̅ δT C δR) +

(1/3) (δU δT C δR) +

**[U^+^T^+^C^+^R^-^]**

L_U_ = δU T̅ C̅ R̅ +

(1/2) (δU δT C̅ R + δU T̅ δC R) +

(1/3) (δU δT δC R) +

L_T_ = U̅ δT C̅ R̅ +

(1/2) (δU δT C̅ R + U̅ δT δC R) +

(1/3) (δU δT δC R) +

L_C_ = U̅ T̅ δC R̅ +

(1/2) (δU T̅ δC R + U̅ δT δC R) +

(1/3) (δU δT δC R) +

L_R_ = U T C δR
