# Supplementary material for: Understory plants evade shading in a temperate deciduous forest amid climate variability by shifting phenology in synchrony with canopy trees
Source: PLoS One. 2024 Jun 26;19(6):e0306023. doi: 10.1371/journal.pone.0306023 (PMC11207122; doi:10.1371/journal.pone.0306023)
Supplement: S1 Fig — The y-axis units are relative measures of light interception, and best used for comparisons within species (see Methods: Section 4). (PDF) [file pone.0306023.s007.pdf]

Supporting Information 7 for Augspurger CK, Salk CF. Understory plants reduce light loss in a temperate deciduous forest amid climate variability by shifting phenology in synchrony with canopy trees. PLoS One. In review.

Supporting Information 7. The relative impact of sapling phenology, temperature, canopy phenology and solar radiation on sapling species' light interception, by year. The y−axis units are relative measures of light interception, and best used for comparisons within species (see Methods: Section 4).

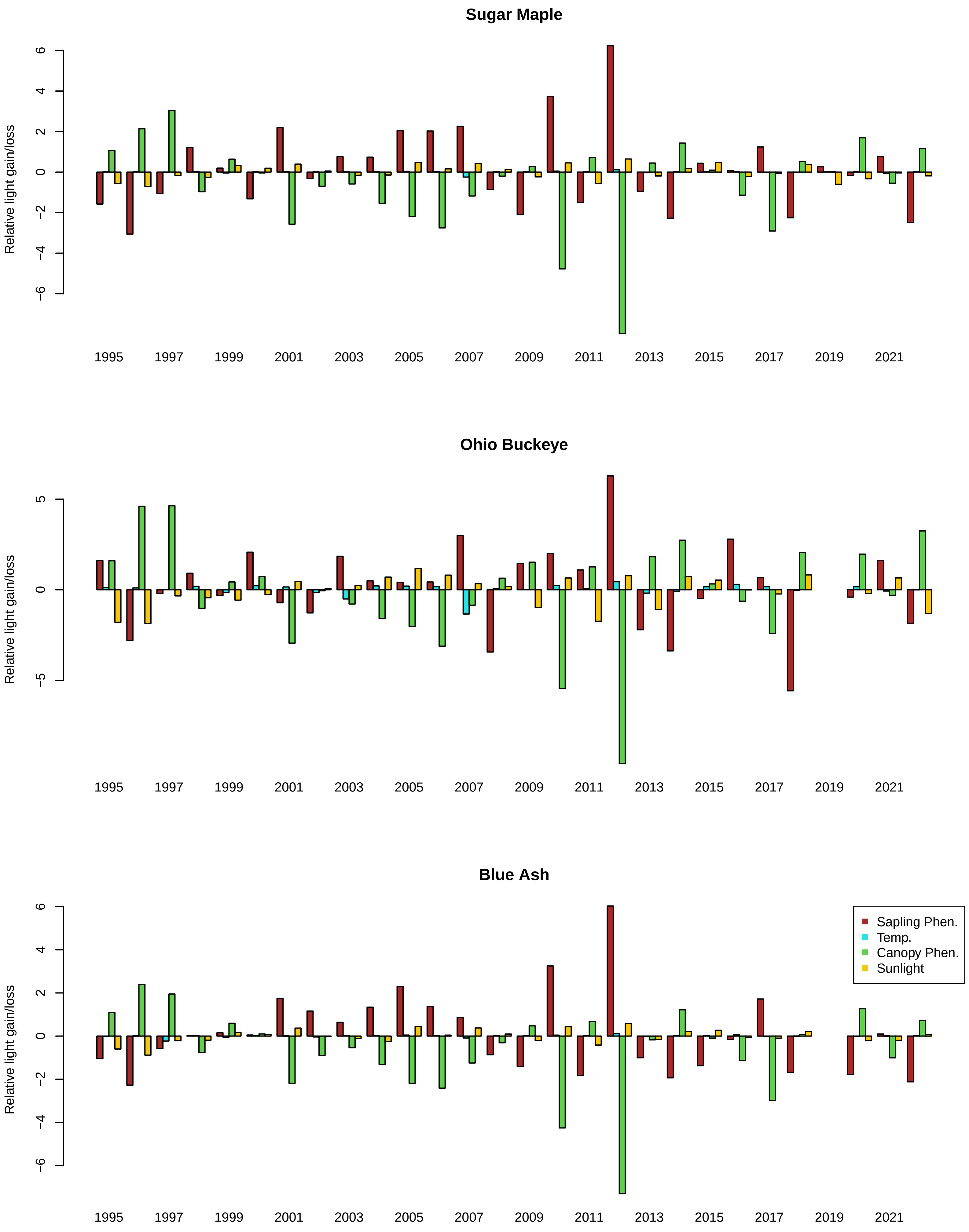

Sugar Maple
Relative light gain/loss
6
4
2
0
−2
−4
−6
1995
1997
1999
2001
2003
2005
2007
2009
2011
2013
2015
2017
2019
2021
Ohio Buckeye
Relative light gain/loss
5
0
−5
1995
1997
1999
2001
2003
2005
2007
2009
2011
2013
2015
2017
2019
2021
Blue Ash
Relative light gain/loss
6
4
2
0
−2
−4
−6
1995
1997
1999
2001
2003
2005
2007
2009
2011
2013
2015
2017
2019
2021
Sapling Phen.
Temp.
Canopy Phen.
Sunlight
