# Supplementary material for: Understory plants evade shading in a temperate deciduous forest amid climate variability by shifting phenology in synchrony with canopy trees
Source: PLoS One. 2024 Jun 26;19(6):e0306023. doi: 10.1371/journal.pone.0306023 (PMC11207122; doi:10.1371/journal.pone.0306023)
Supplement: S2 Fig — The y-axis units are relative measures of light interception, and best used for comparisons within species (see Methods: Section 4). (PDF) [file pone.0306023.s008.pdf]

Supporting Information 8 for Augspurger CK, Salk CF. Understory plants reduce light loss in a temperate deciduous forest amid climate variability by shifting phenology in synchrony with canopy trees. PLoS One. In review.

Supporting Information 8. The relative impact of herb phenology, temperature, understory phenology and solar radiation on herb species' light interception, by year. The y−axis units are relative measures of light interception, and best used for comparisons within species (see Methods: Section 4).

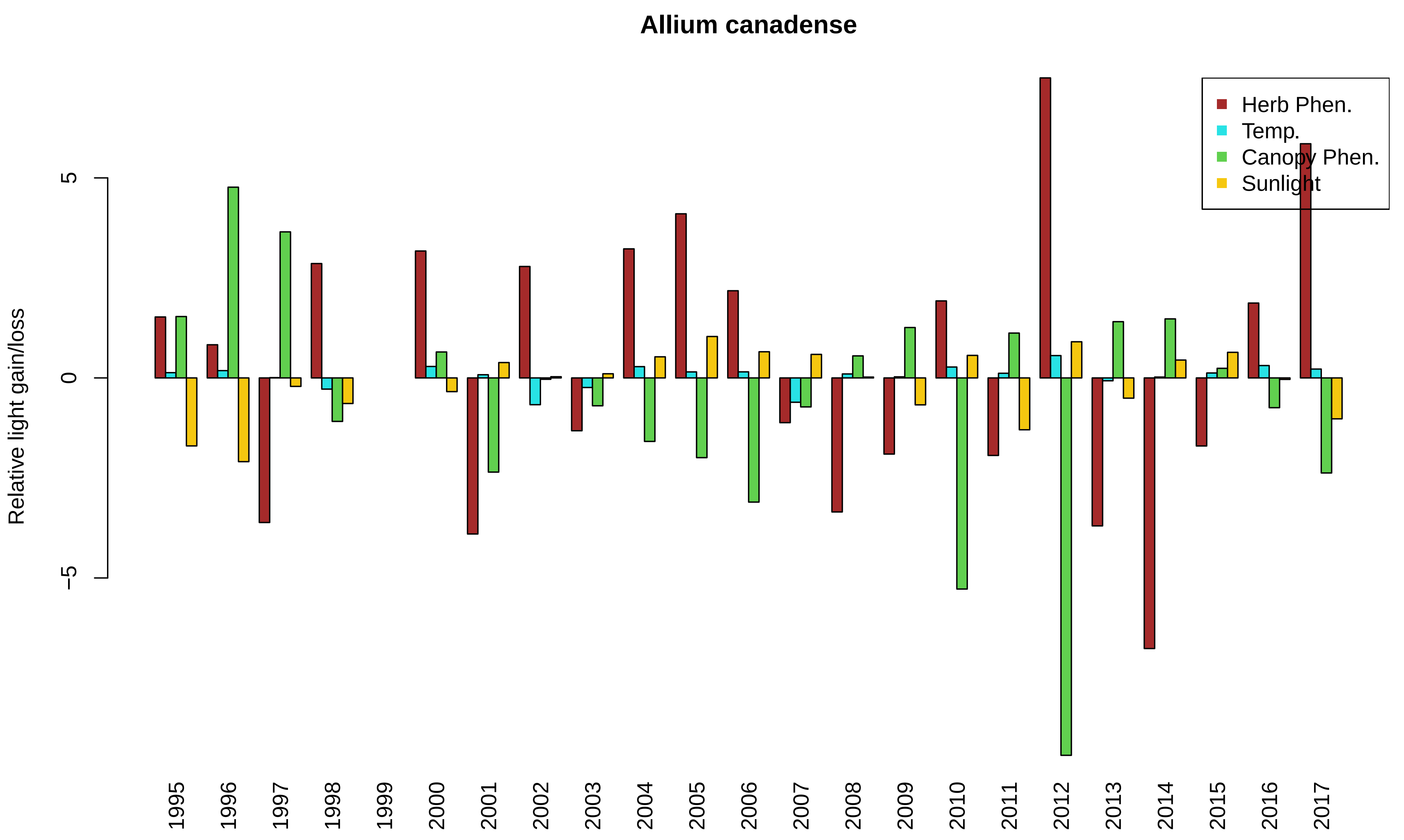
Allium canadense
Herb Phen.
Temp.
Canopy Phen.
Sunlight
Relative light gain/loss
5
0
−5
1995
1996
1997
1998
1999
2000
2001
2002
2003
2004
2005
2006
2007
2008
2009
2010
2011
2012
2013
2014
2015
2016
2017

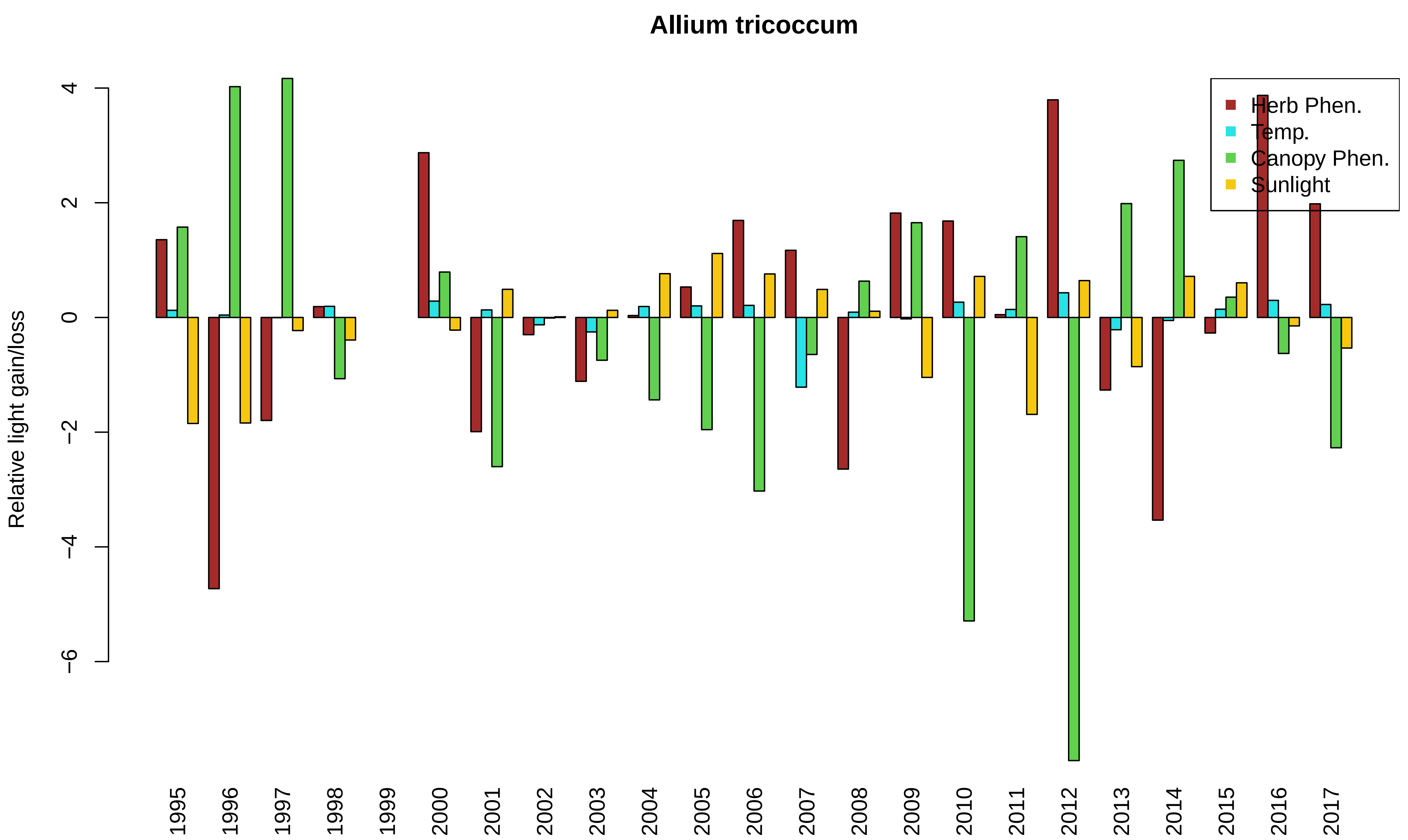
Allium tricoccum
Herb Phen.
Temp.
Canopy Phen.
Sunlight
Relative light gain/loss
4
2
0
−2
−4
−6
1995
1996
1997
1998
1999
2000
2001
2002
2003
2004
2005
2006
2007
2008
2009
2010
2011
2012
2013
2014
2015
2016
2017

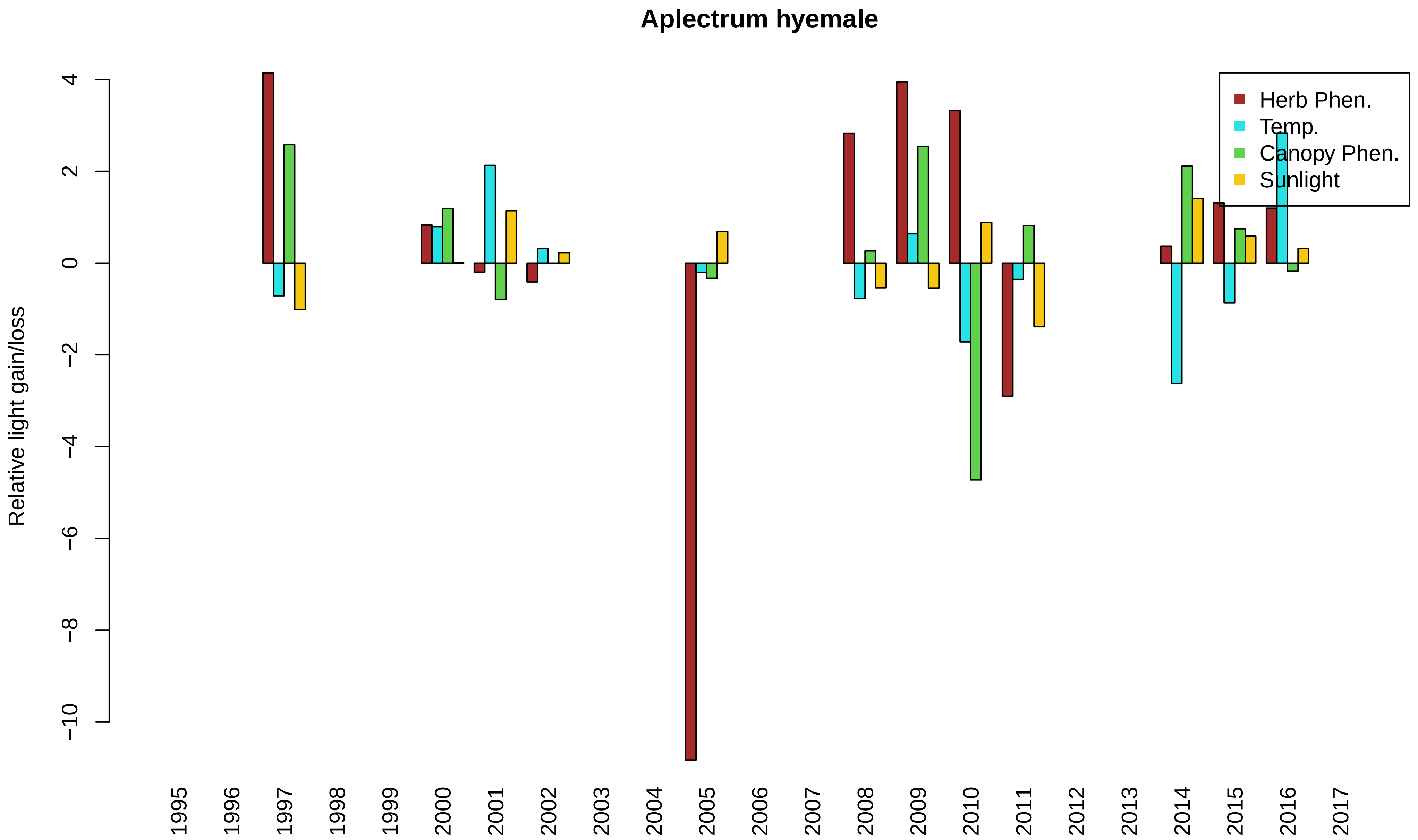

Aplectrum hyemale
Relative light gain/loss
4
2
0
−2
−4
−6
−8
−10
1995
1996
1997
1998
1999
2000
2001
2002
2003
2004
2005
2006
2007
2008
2009
2010
2011
2012
2013
2014
2015
2016
2017
Herb Phen.
Temp.
Canopy Phen.
Sunlight

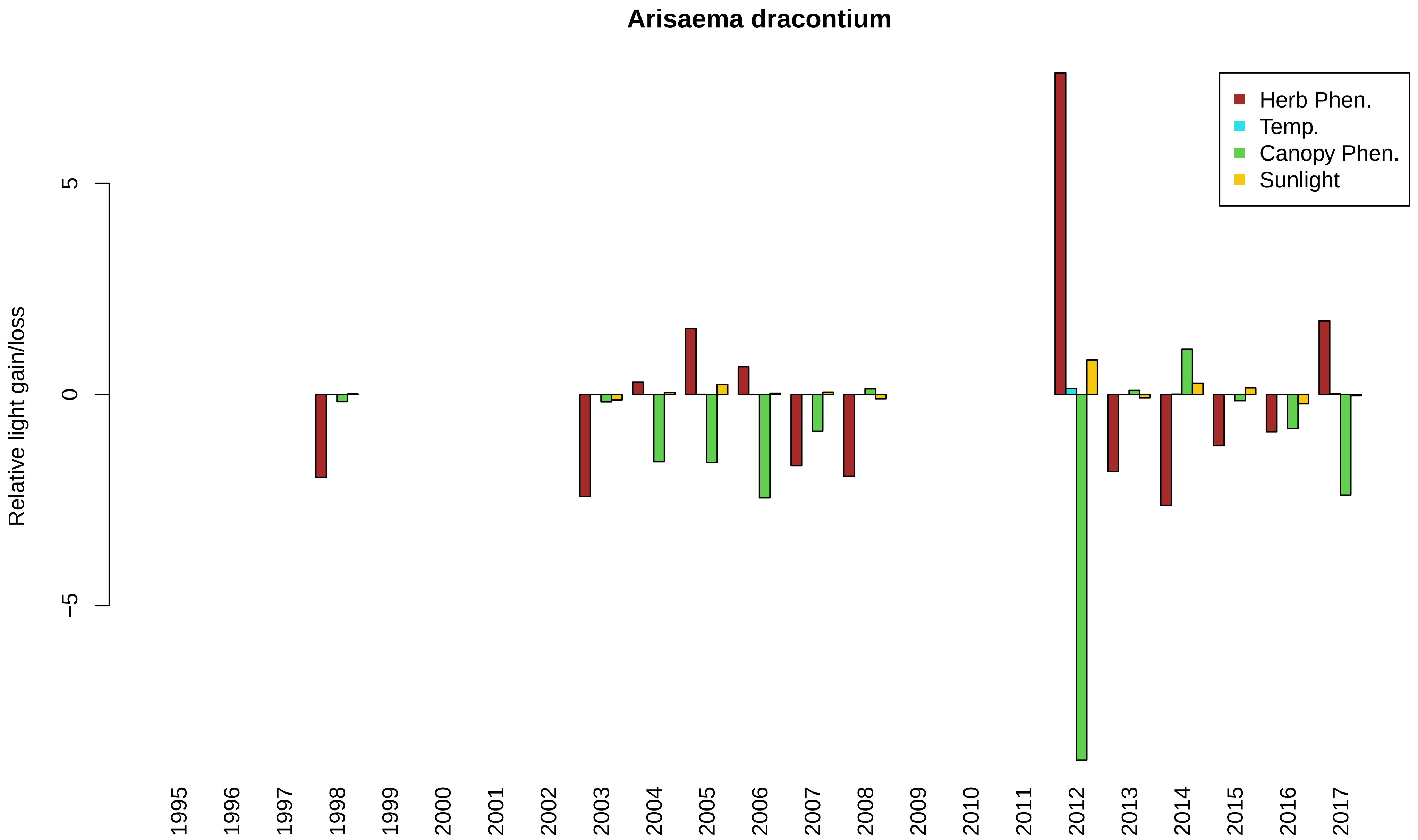
Arisaema dracontium
Herb Phen.
Temp.
Canopy Phen.
Sunlight
Relative light gain/loss
5
0
−5
1995
1996
1997
1998
1999
2000
2001
2002
2003
2004
2005
2006
2007
2008
2009
2010
2011
2012
2013
2014
2015
2016
2017

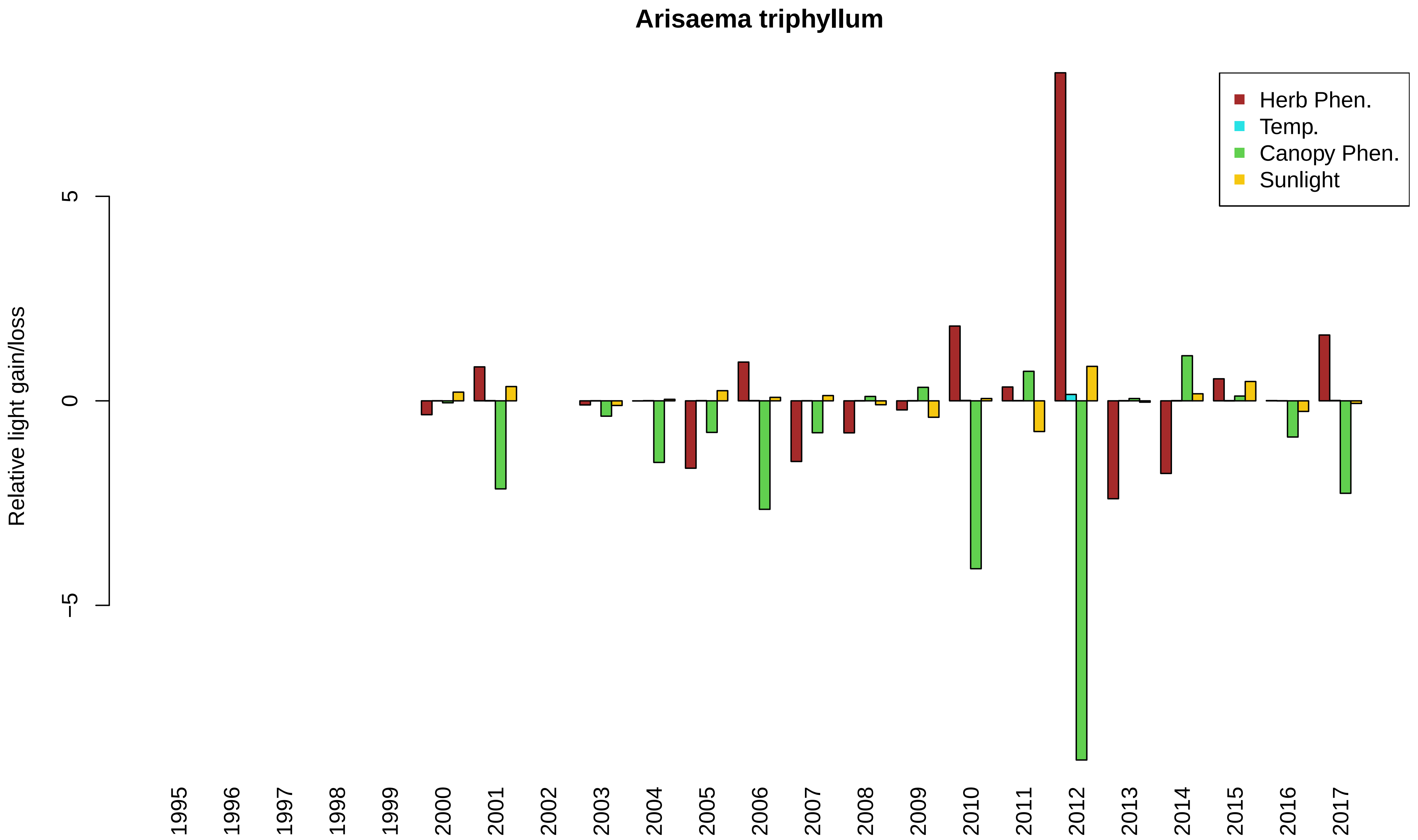
Arisaema triphyllum
Herb Phen.
Temp.
Canopy Phen.
Sunlight
Relative light gain/loss
5
0
−5
1995
1996
1997
1998
1999
2000
2001
2002
2003
2004
2005
2006
2007
2008
2009
2010
2011
2012
2013
2014
2015
2016
2017

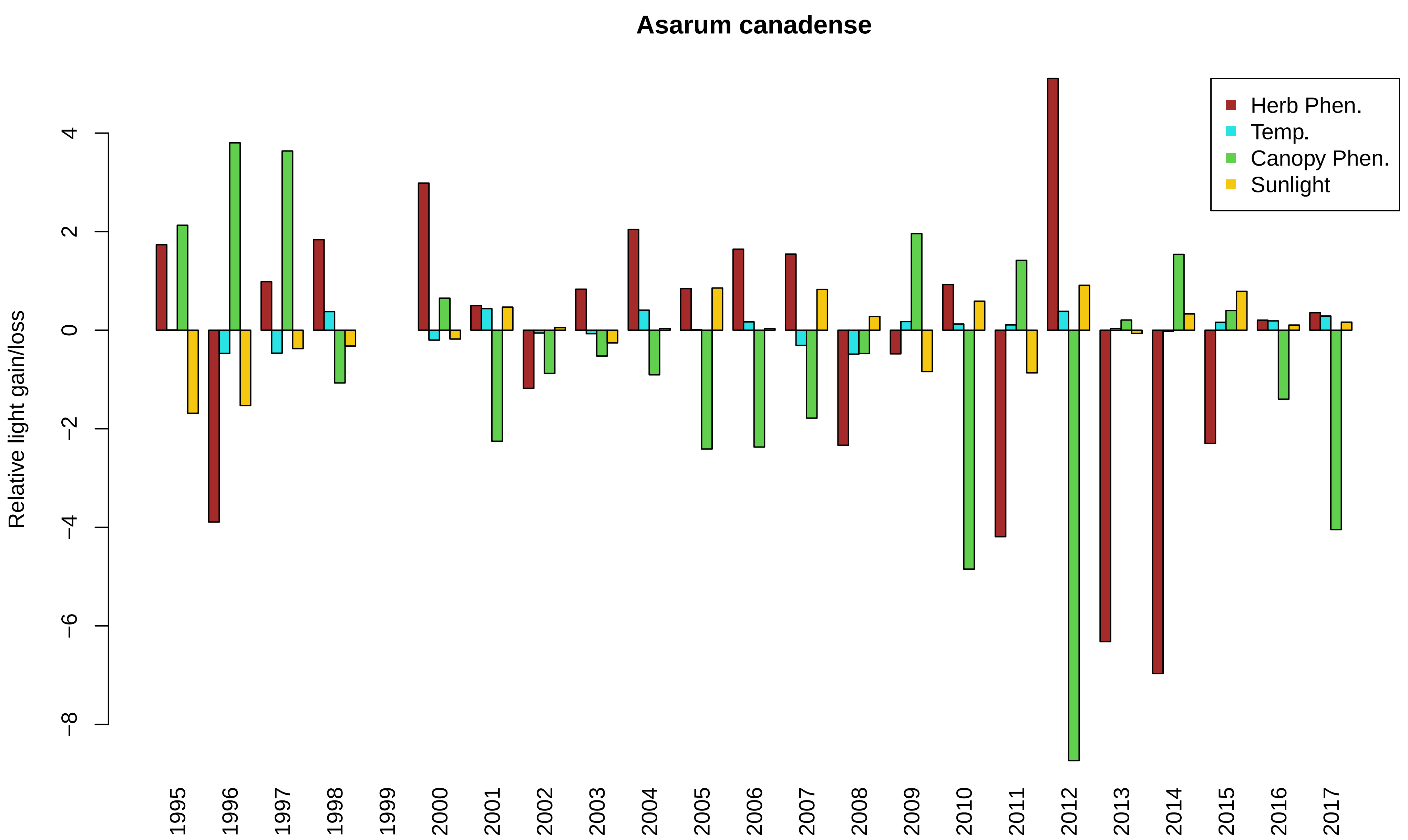

Asarum canadense
Herb Phen.
Temp.
Canopy Phen.
Sunlight
Relative light gain/loss
4
2
0
−2
−4
−6
−8
1995
1996
1997
1998
1999
2000
2001
2002
2003
2004
2005
2006
2007
2008
2009
2010
2011
2012
2013
2014
2015
2016
2017

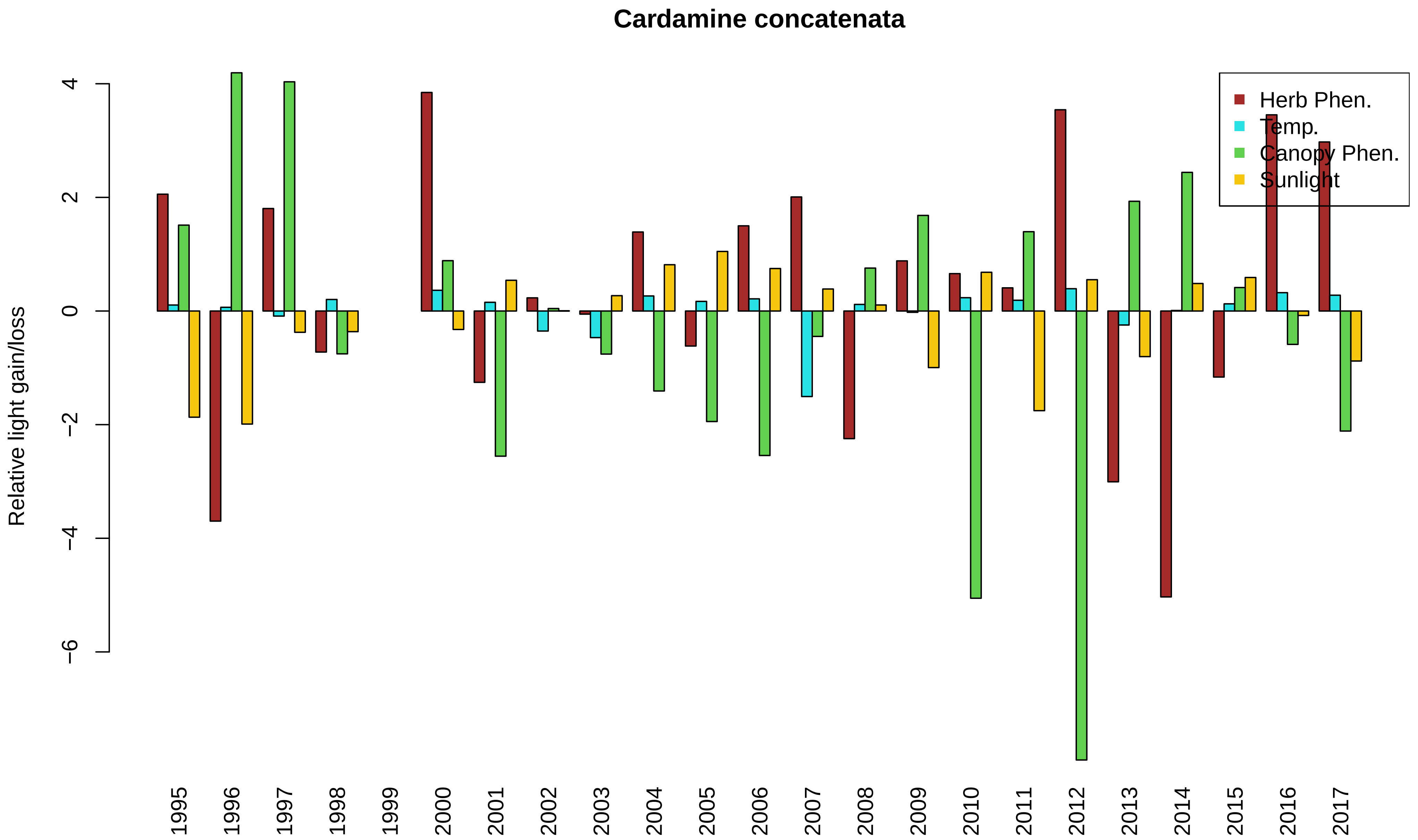
Cardamine concatenata
Relative light gain/loss
4
2
0
−2
−4
−6
Herb Phen.
Temp.
Canopy Phen.
Sunlight
1995
1996
1997
1998
1999
2000
2001
2002
2003
2004
2005
2006
2007
2008
2009
2010
2011
2012
2013
2014
2015
2016
2017

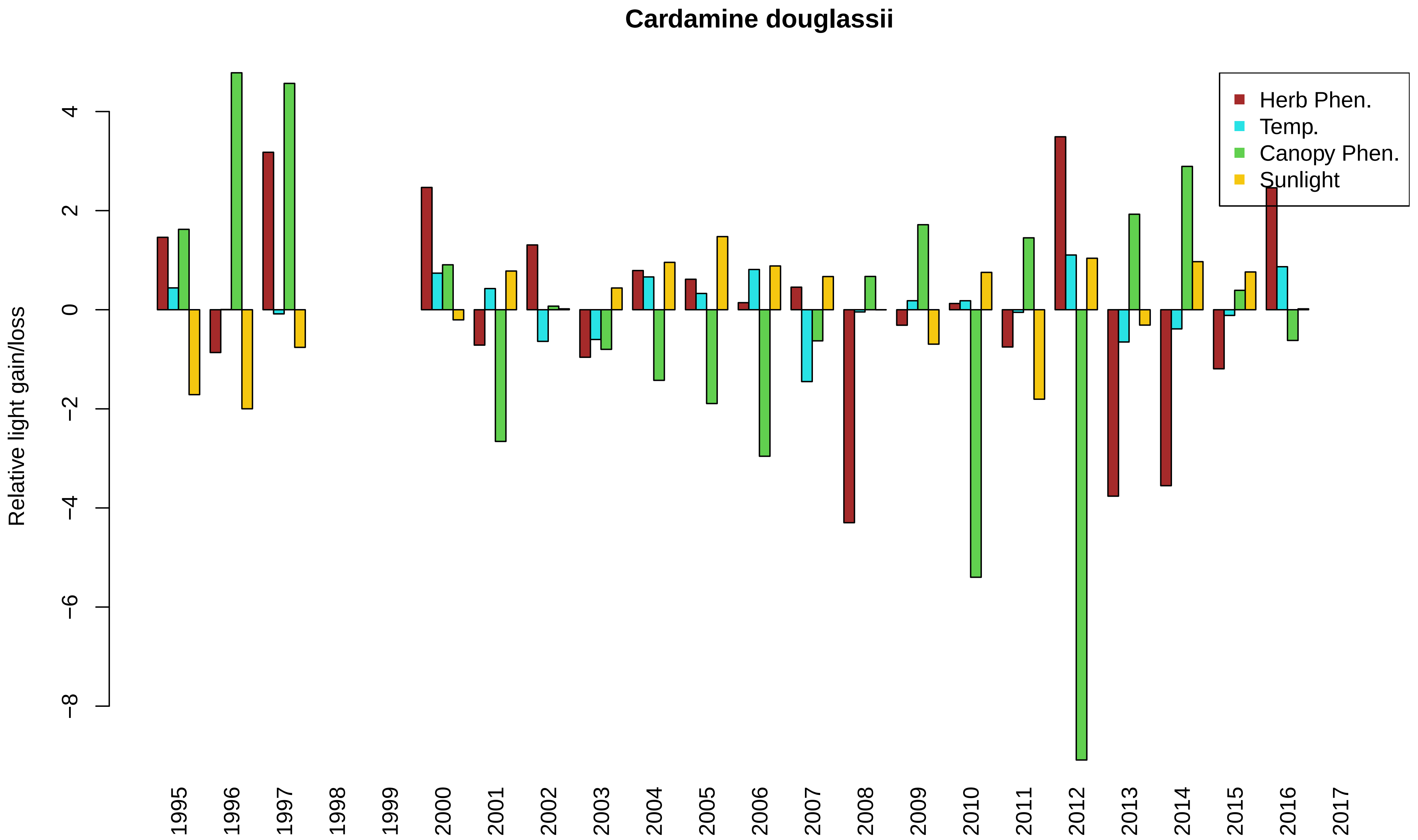
Cardamine douglassii
Herb Phen.
Temp.
Canopy Phen.
Sunlight
Relative light gain/loss
4
2
0
−2
−4
−6
−8
1995
1996
1997
1998
1999
2000
2001
2002
2003
2004
2005
2006
2007
2008
2009
2010
2011
2012
2013
2014
2015
2016
2017

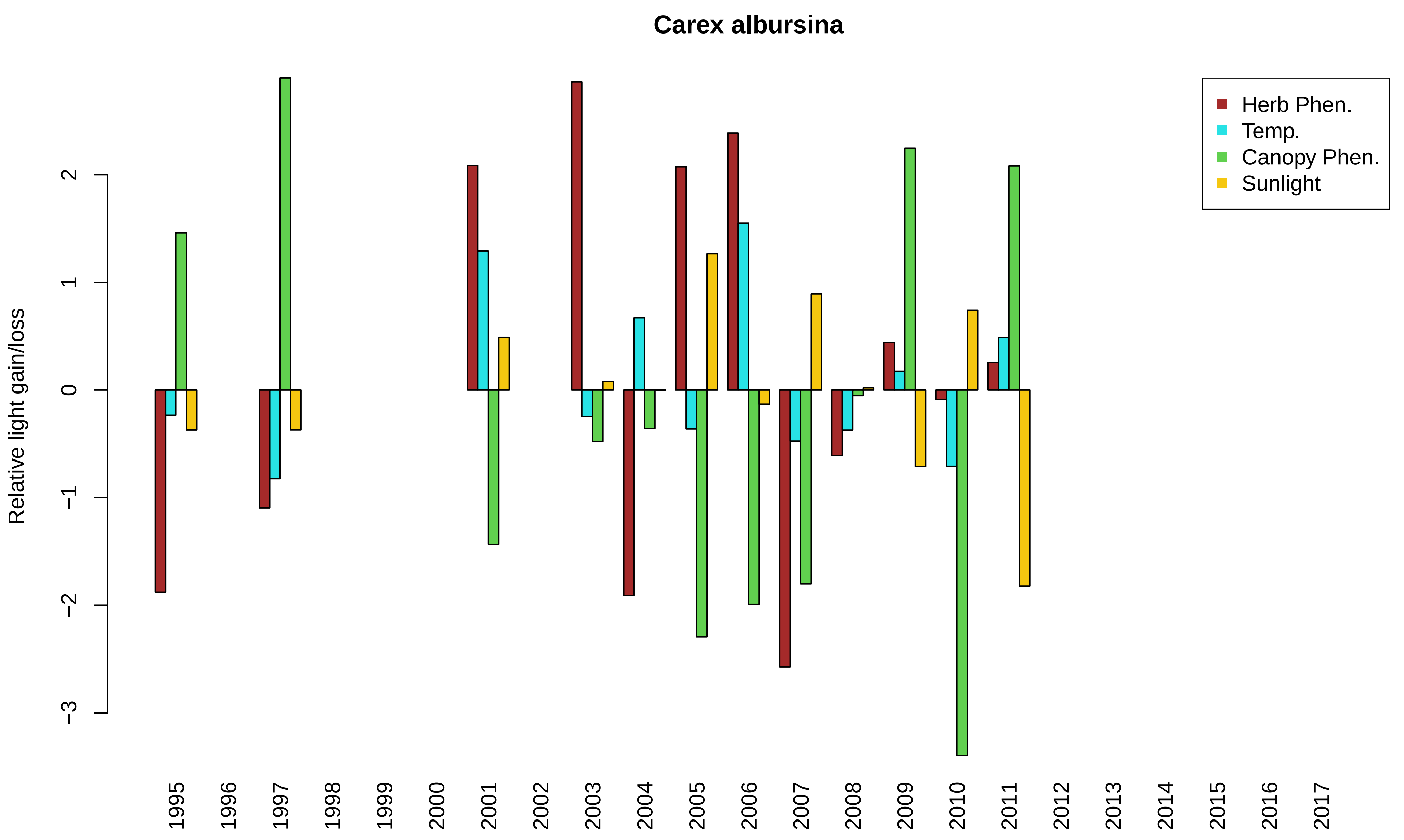
Carex albursina
Relative light gain/loss
−3
−2
−1
0
1
2
Herb Phen.
Temp.
Canopy Phen.
Sunlight
1995
1996
1997
1998
1999
2000
2001
2002
2003
2004
2005
2006
2007
2008
2009
2010
2011
2012
2013
2014
2015
2016
2017

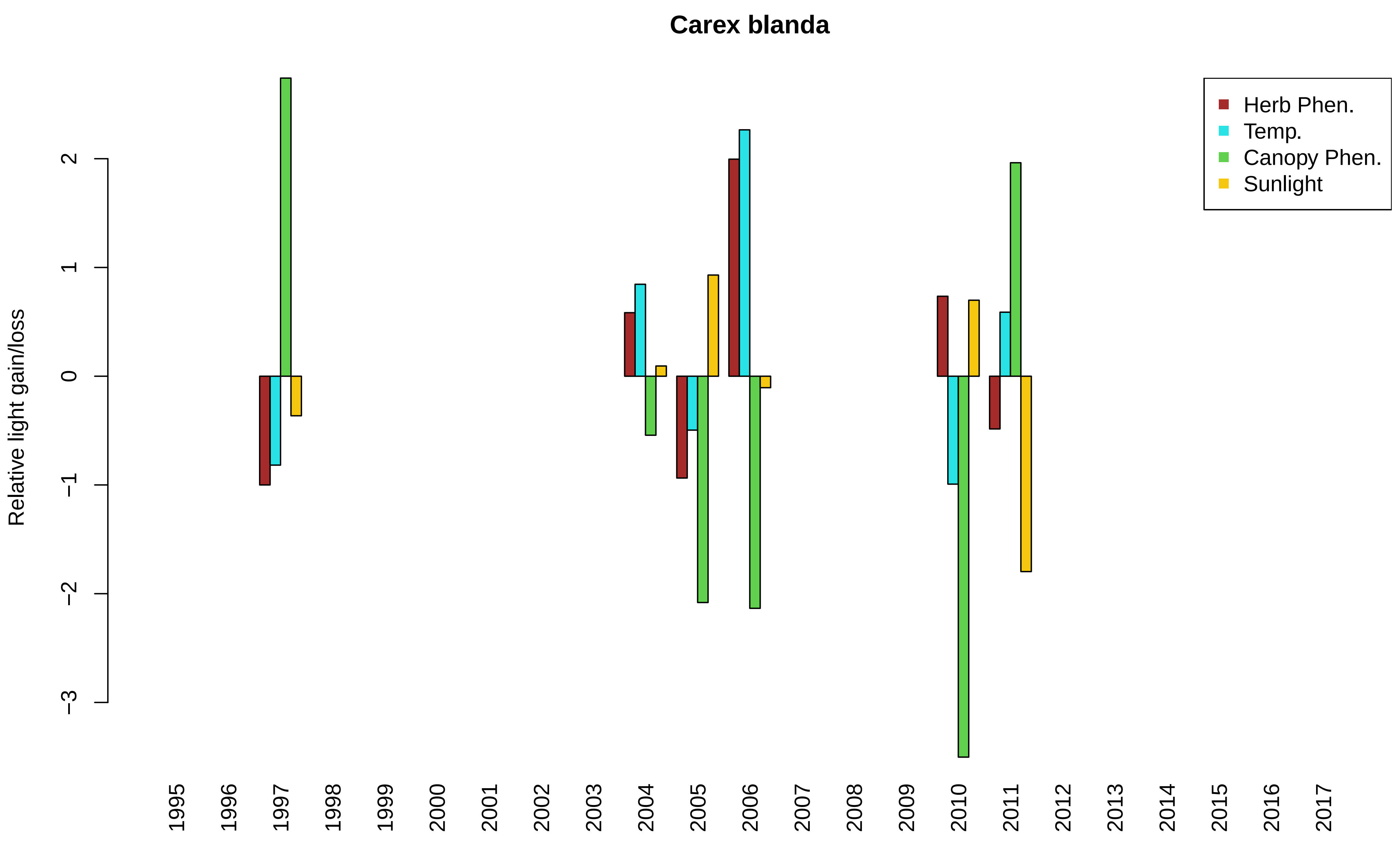
Carex blanda
Herb Phen.
Temp.
Canopy Phen.
Sunlight
Relative light gain/loss
2
1
0
−1
−2
−3
1995
1996
1997
1998
1999
2000
2001
2002
2003
2004
2005
2006
2007
2008
2009
2010
2011
2012
2013
2014
2015
2016
2017

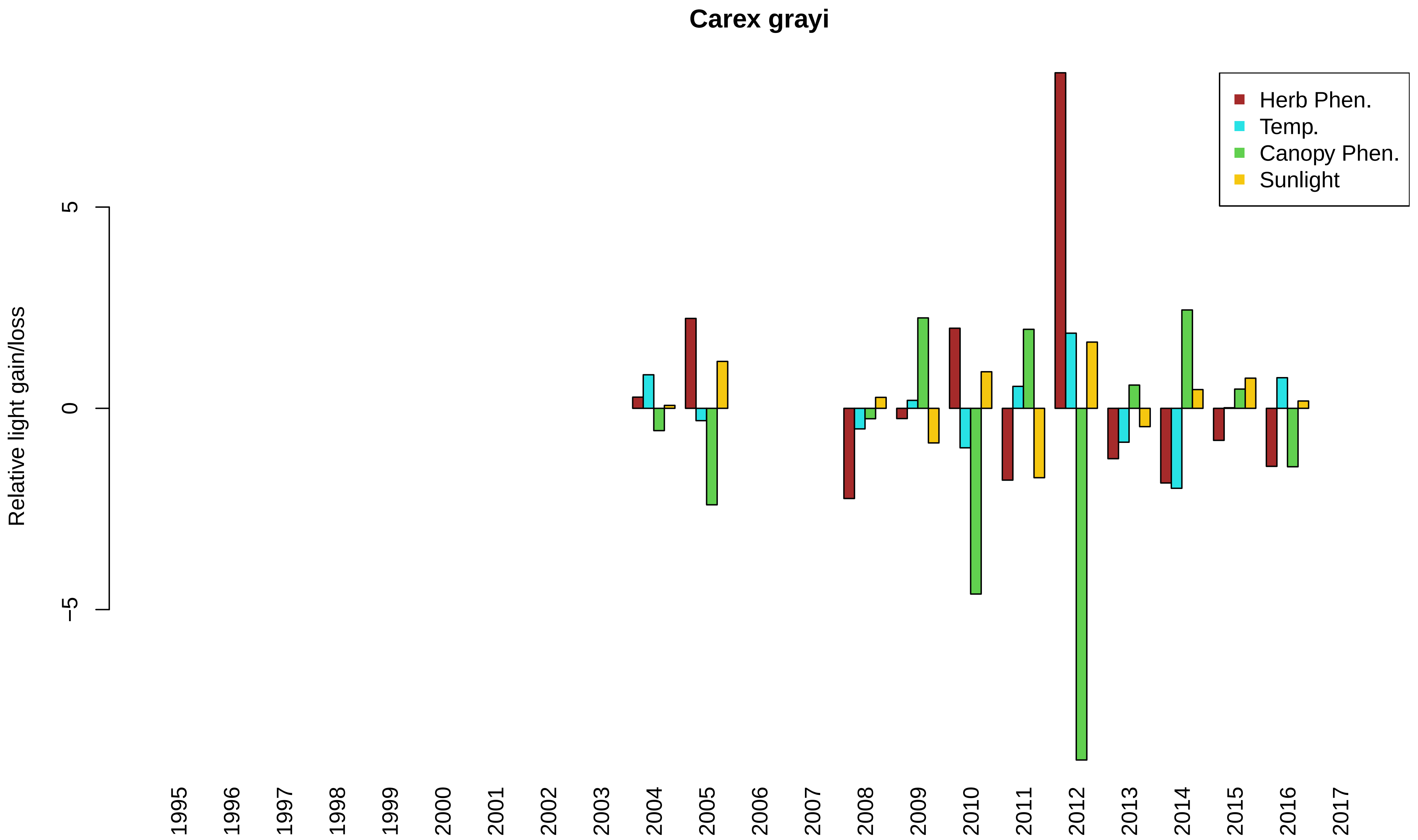

Carex grayi
Herb Phen.
Temp.
Canopy Phen.
Sunlight
Relative light gain/loss
5
0
−5
1995
1996
1997
1998
1999
2000
2001
2002
2003
2004
2005
2006
2007
2008
2009
2010
2011
2012
2013
2014
2015
2016
2017

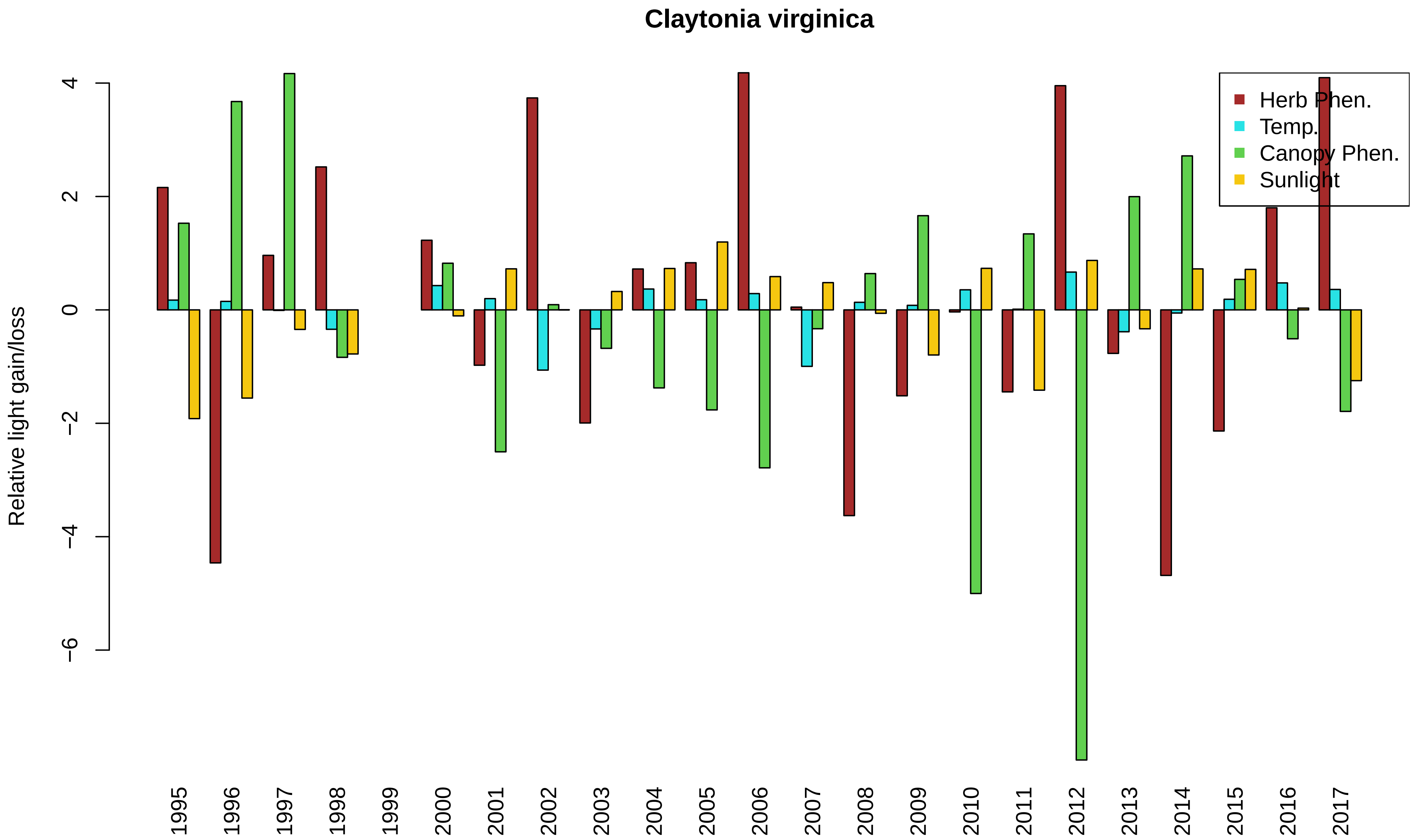

Claytonia virginica
Herb Phen.
Temp.
Canopy Phen.
Sunlight
Relative light gain/loss
4
2
0
−2
−4
−6
1995
1996
1997
1998
1999
2000
2001
2002
2003
2004
2005
2006
2007
2008
2009
2010
2011
2012
2013
2014
2015
2016
2017

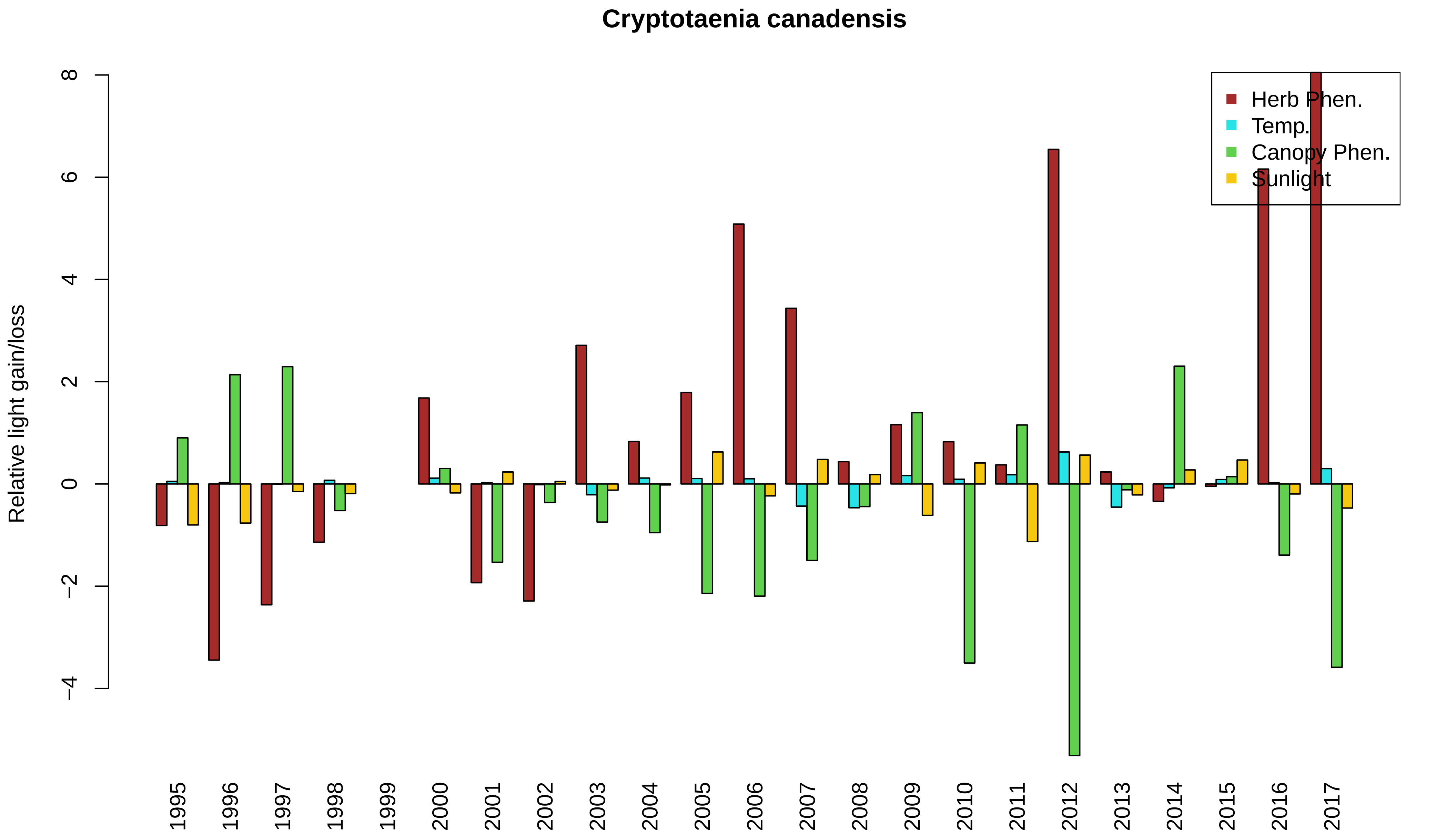

Cryptotaenia canadensis
Relative light gain/loss
8
6
4
2
0
−2
−4
Herb Phen.
Temp.
Canopy Phen.
Sunlight
1995
1996
1997
1998
1999
2000
2001
2002
2003
2004
2005
2006
2007
2008
2009
2010
2011
2012
2013
2014
2015
2016
2017

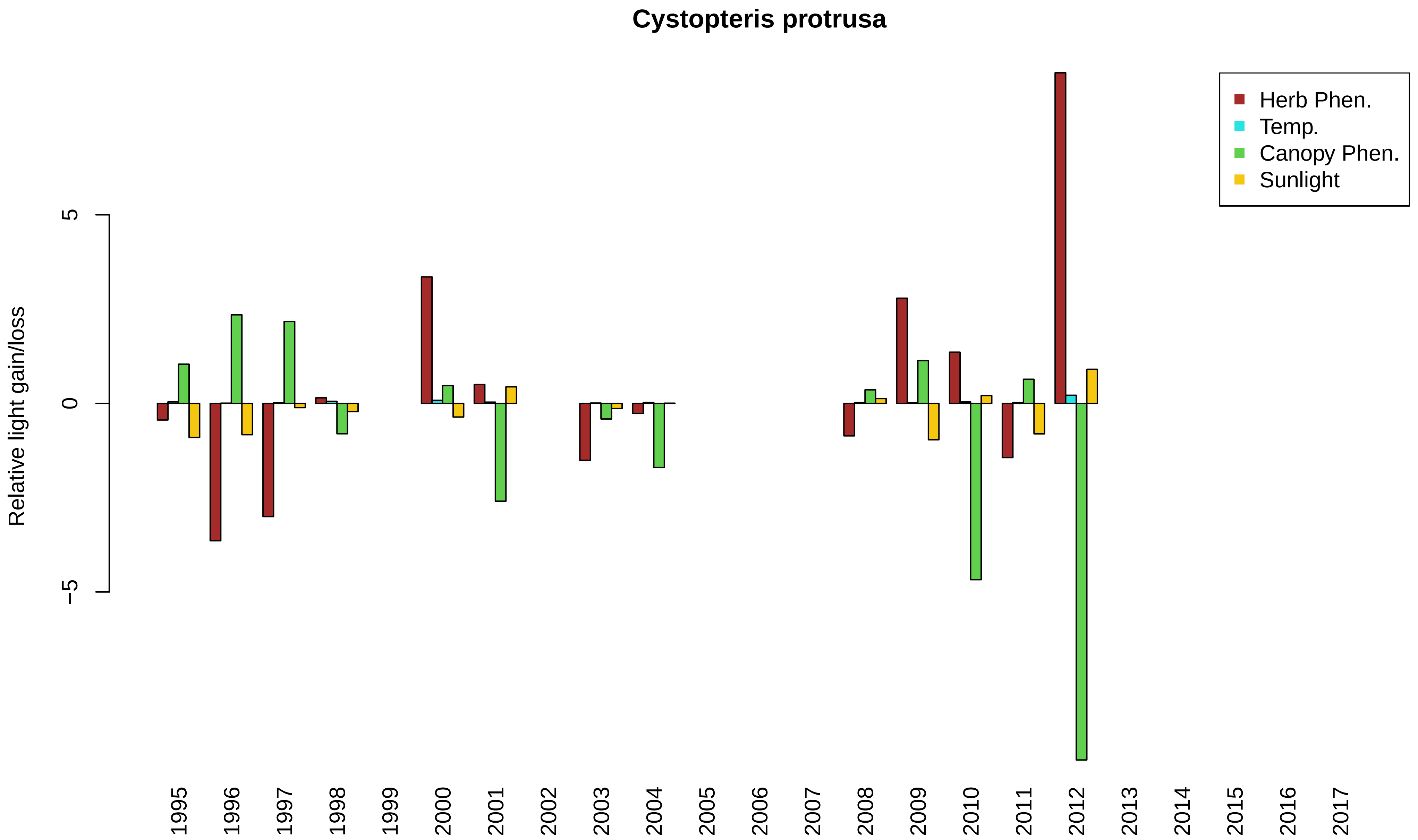

Cystopteris protrusa
Herb Phen.
Temp.
Canopy Phen.
Sunlight
Relative light gain/loss
5
0
−5
1995
1996
1997
1998
1999
2000
2001
2002
2003
2004
2005
2006
2007
2008
2009
2010
2011
2012
2013
2014
2015
2016
2017

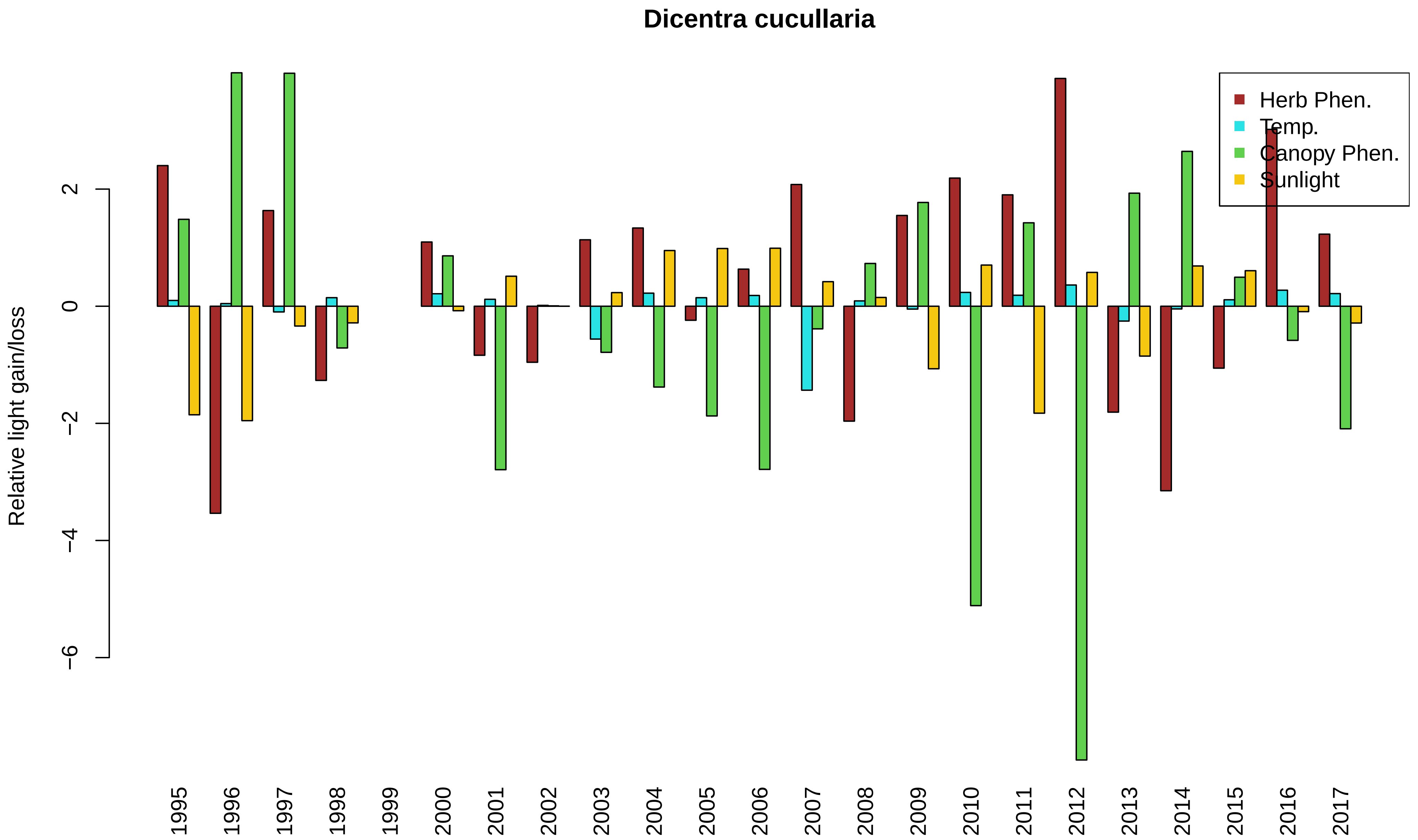

Dicentra cucullaria
Herb Phen.
Temp.
Canopy Phen.
Sunlight
Relative light gain/loss
2
0
−2
−4
−6
1995
1996
1997
1998
1999
2000
2001
2002
2003
2004
2005
2006
2007
2008
2009
2010
2011
2012
2013
2014
2015
2016
2017

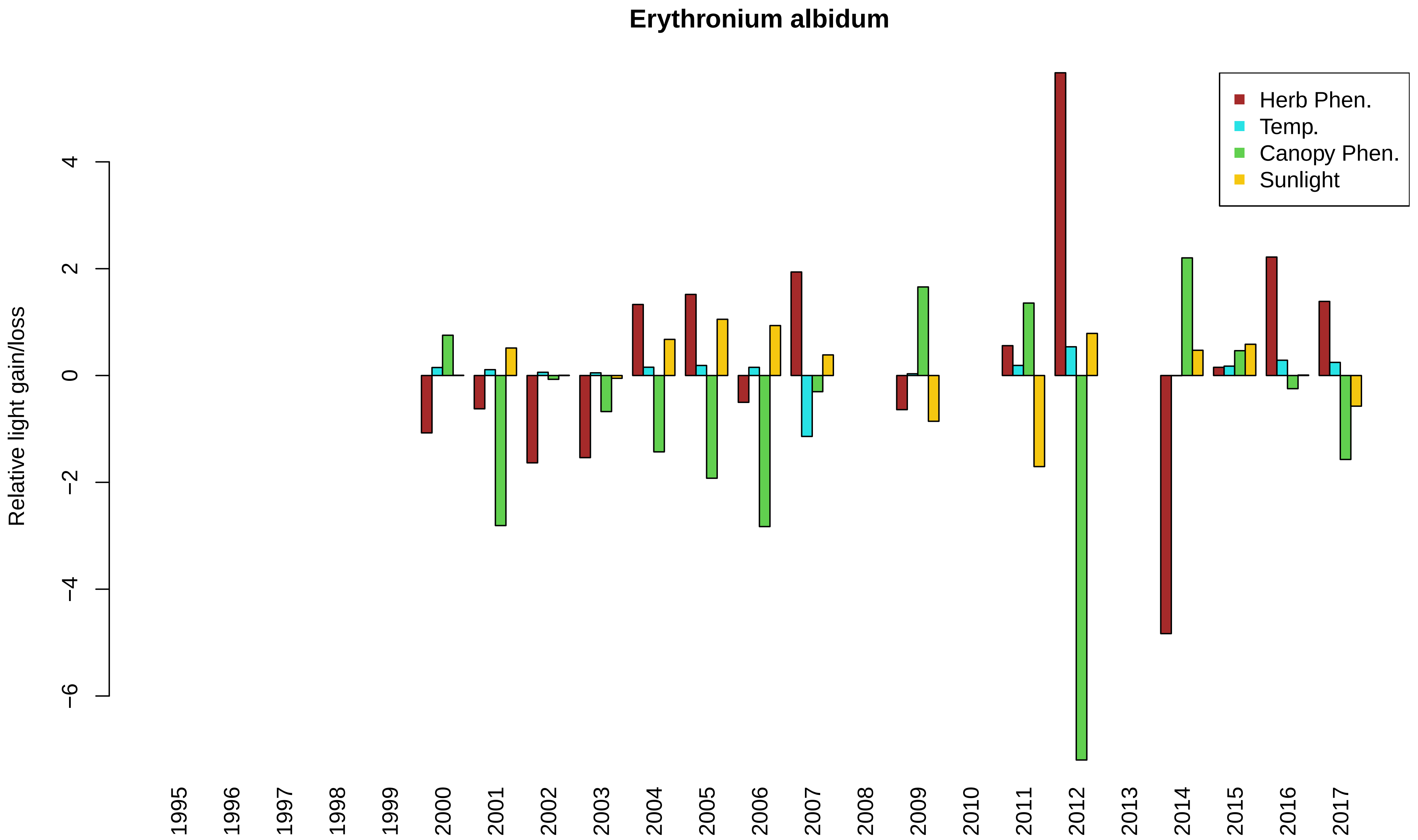

Erythronium albidum
Herb Phen.
Temp.
Canopy Phen.
Sunlight
Relative light gain/loss
4
2
0
−2
−4
−6
1995
1996
1997
1998
1999
2000
2001
2002
2003
2004
2005
2006
2007
2008
2009
2010
2011
2012
2013
2014
2015
2016
2017

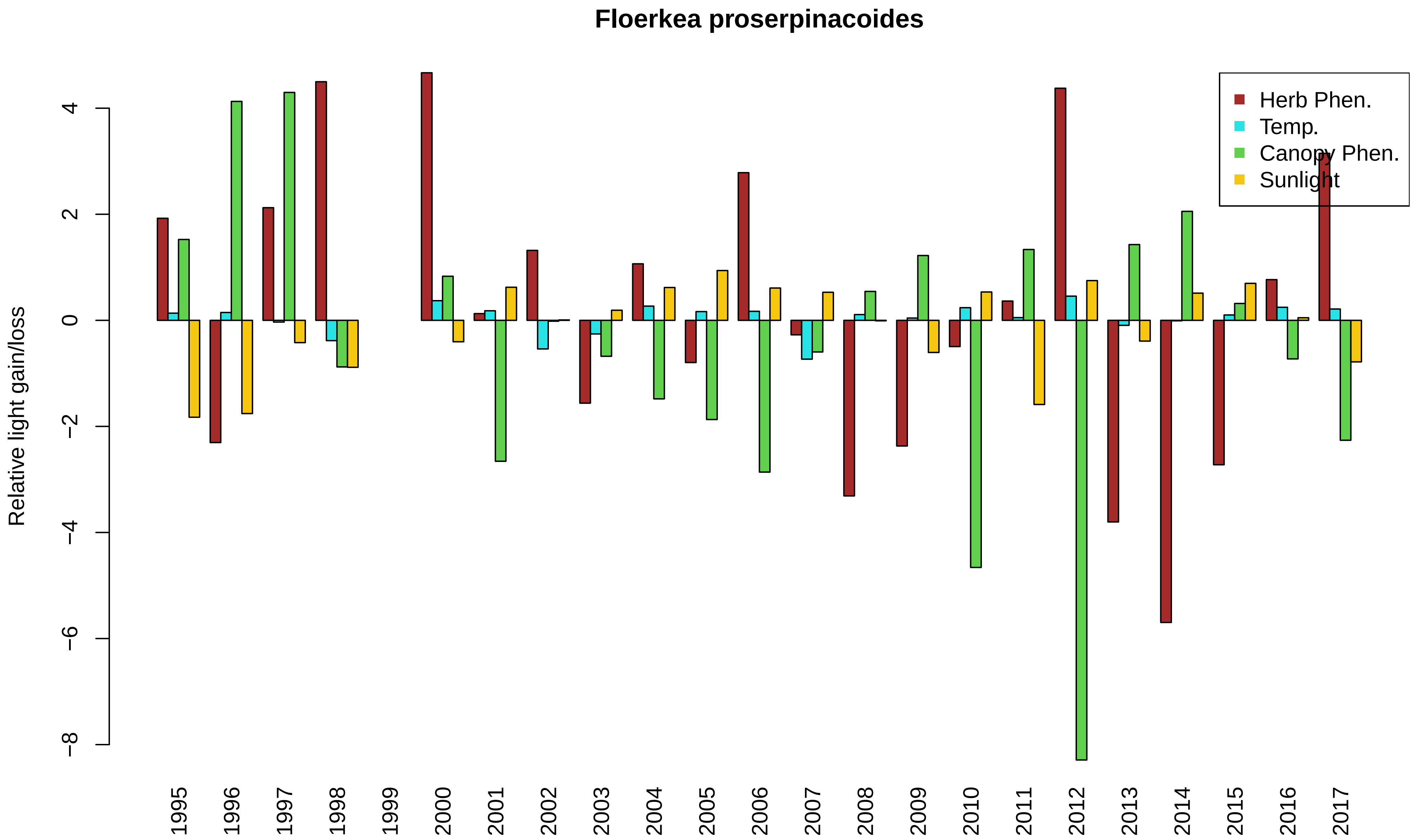
Floerkea proserpinacoides
Herb Phen.
Temp.
Canopy Phen.
Sunlight
Relative light gain/loss
4
2
0
−2
−4
−6
−8
1995
1996
1997
1998
1999
2000
2001
2002
2003
2004
2005
2006
2007
2008
2009
2010
2011
2012
2013
2014
2015
2016
2017

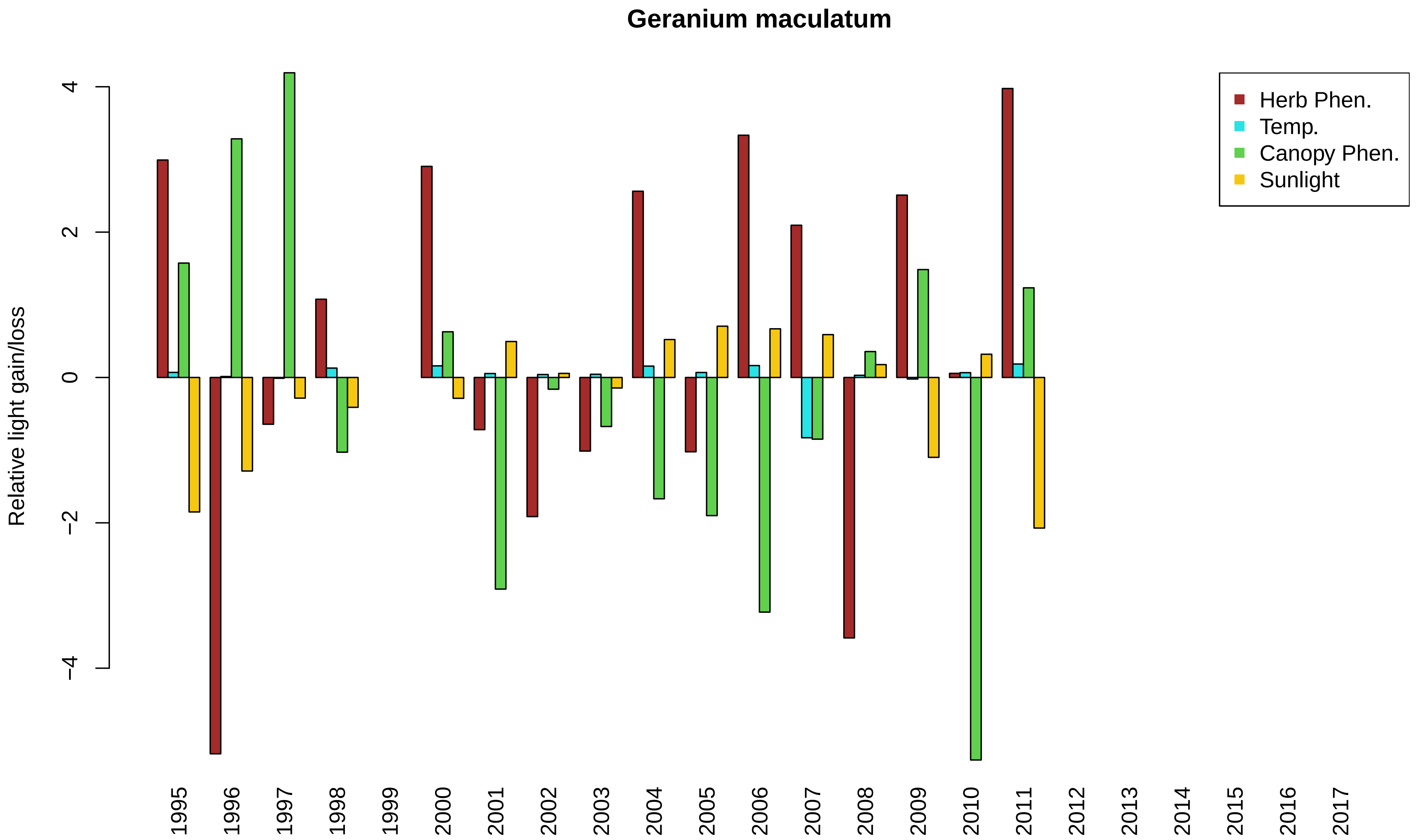
Geranium maculatum
Herb Phen.
Temp.
Canopy Phen.
Sunlight
Relative light gain/loss
4
2
0
−2
−4
1995
1996
1997
1998
1999
2000
2001
2002
2003
2004
2005
2006
2007
2008
2009
2010
2011
2012
2013
2014
2015
2016
2017

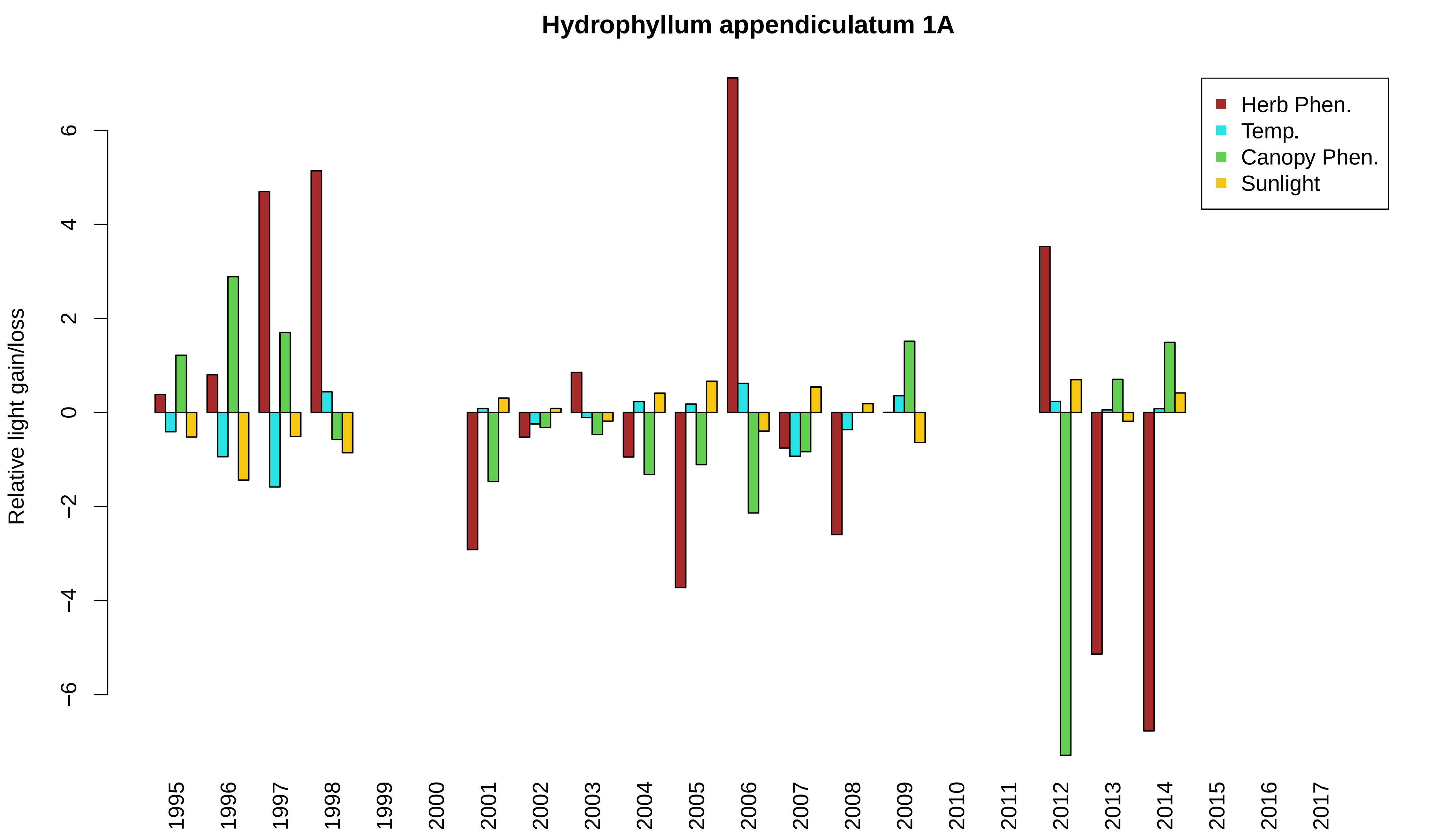
Hydrophyllum appendiculatum 1A
Herb Phen.
Temp.
Canopy Phen.
Sunlight
Relative light gain/loss
6
4
2
0
−2
−4
−6
1995
1996
1997
1998
1999
2000
2001
2002
2003
2004
2005
2006
2007
2008
2009
2010
2011
2012
2013
2014
2015
2016
2017

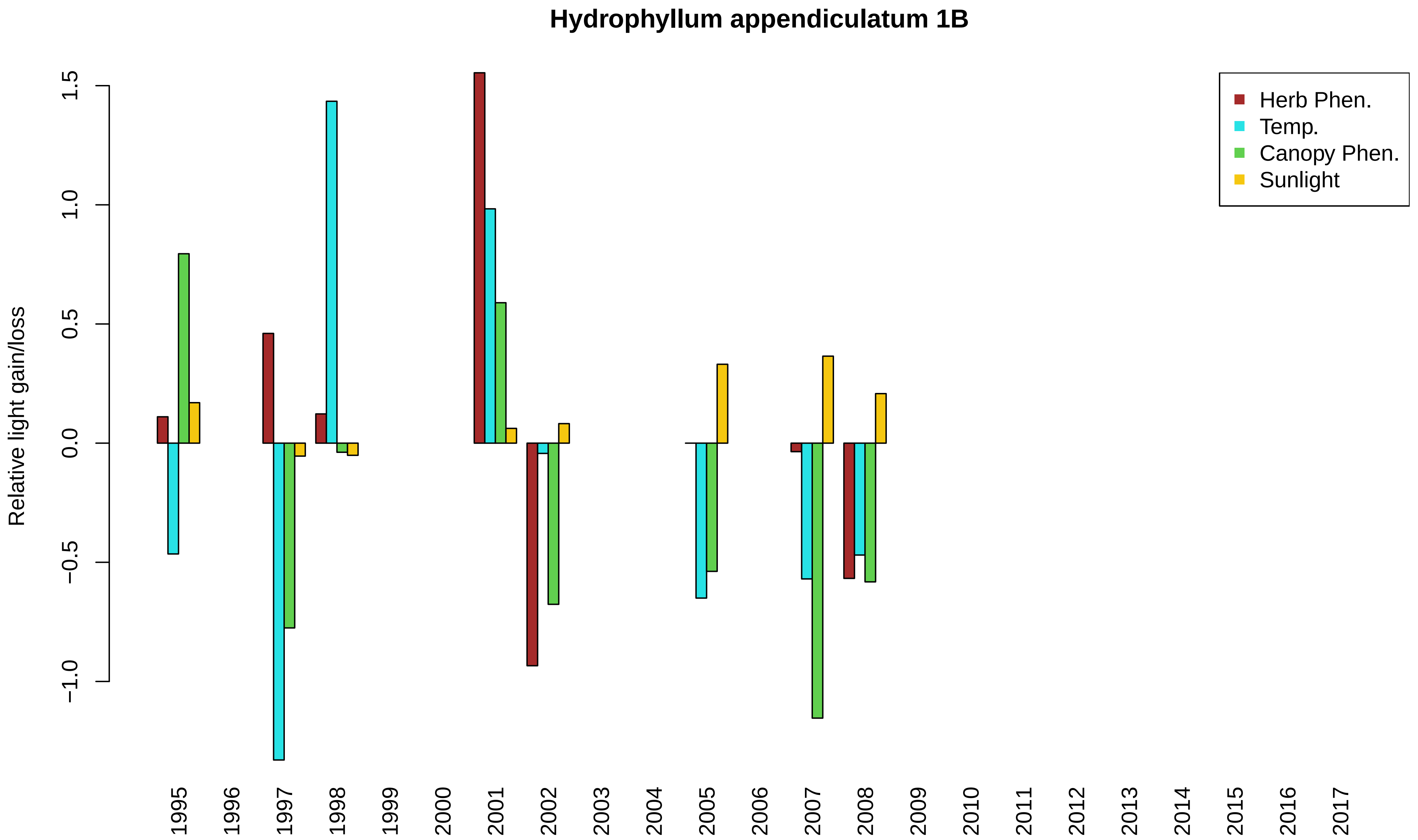

Hydrophyllum appendiculatum 1B
Relative light gain/loss
1.5
1.0
0.5
0.0
−0.5
−1.0
Herb Phen.
Temp.
Canopy Phen.
Sunlight
1995
1996
1997
1998
1999
2000
2001
2002
2003
2004
2005
2006
2007
2008
2009
2010
2011
2012
2013
2014
2015
2016
2017

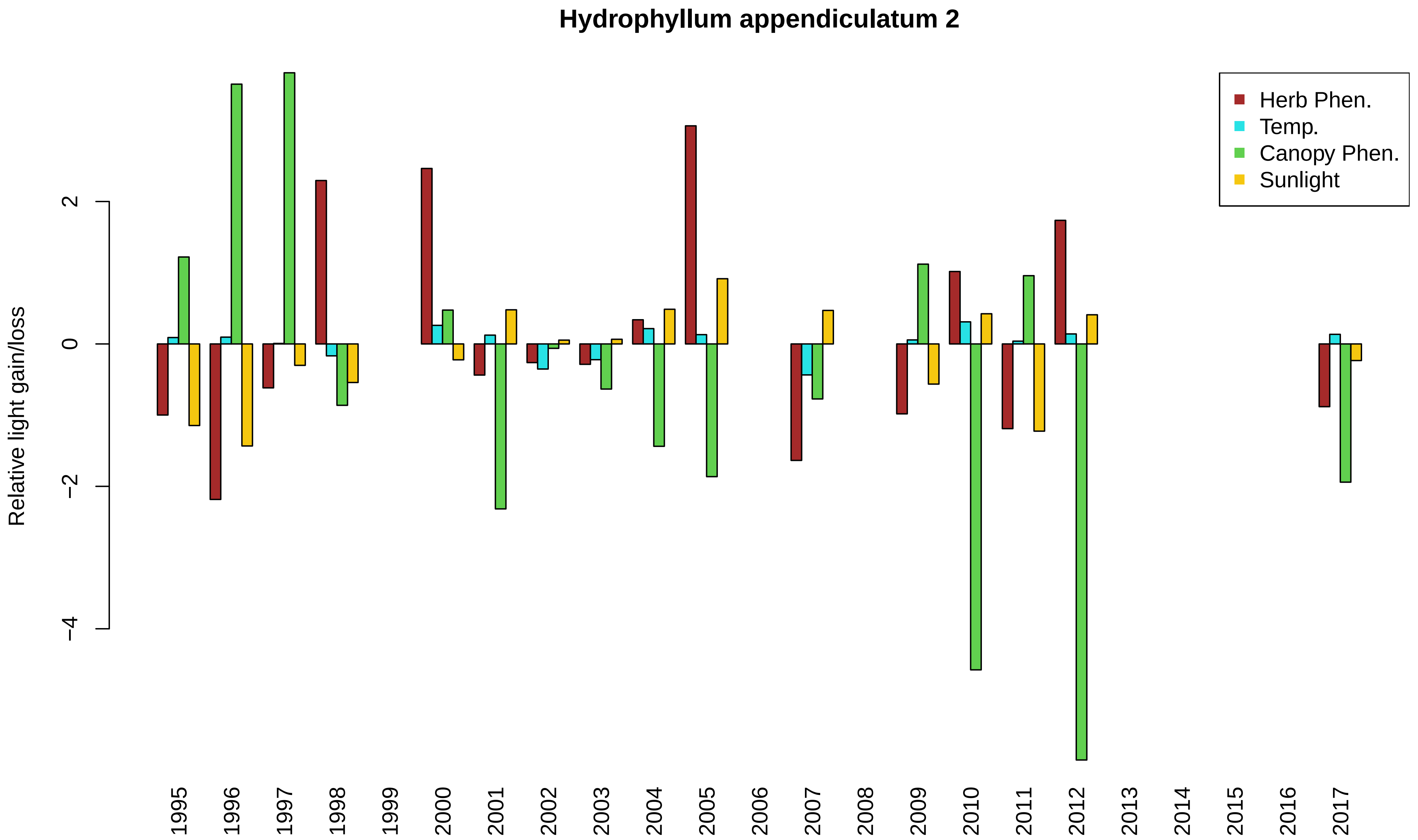
Hydrophyllum appendiculatum 2
Herb Phen.
Temp.
Canopy Phen.
Sunlight
Relative light gain/loss
2
0
−2
−4
1995
1996
1997
1998
1999
2000
2001
2002
2003
2004
2005
2006
2007
2008
2009
2010
2011
2012
2013
2014
2015
2016
2017

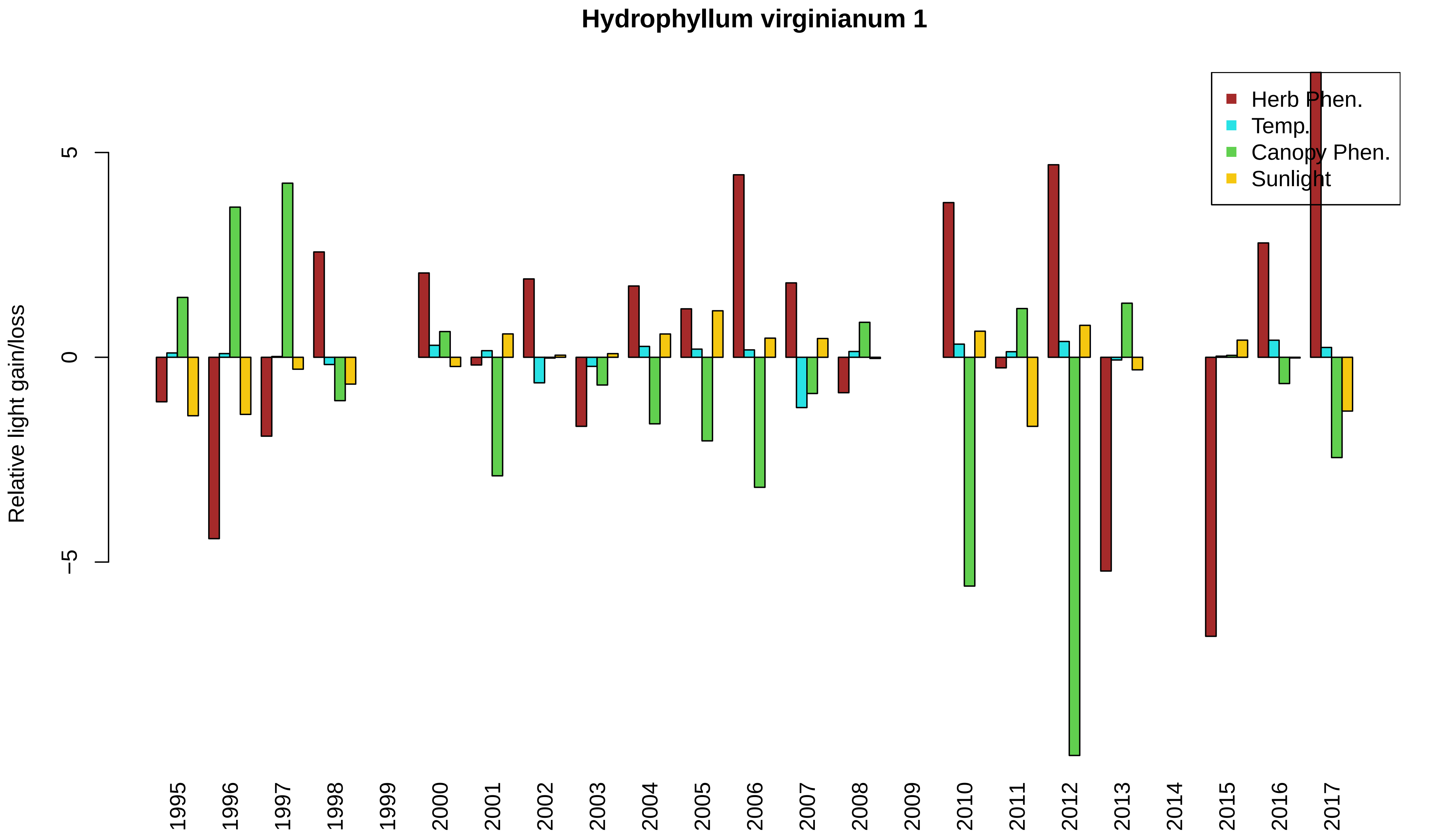
Hydrophyllum virginianum 1
Herb Phen.
Temp.
Canopy Phen.
Sunlight
Relative light gain/loss
5
0
−5
1995
1996
1997
1998
1999
2000
2001
2002
2003
2004
2005
2006
2007
2008
2009
2010
2011
2012
2013
2014
2015
2016
2017

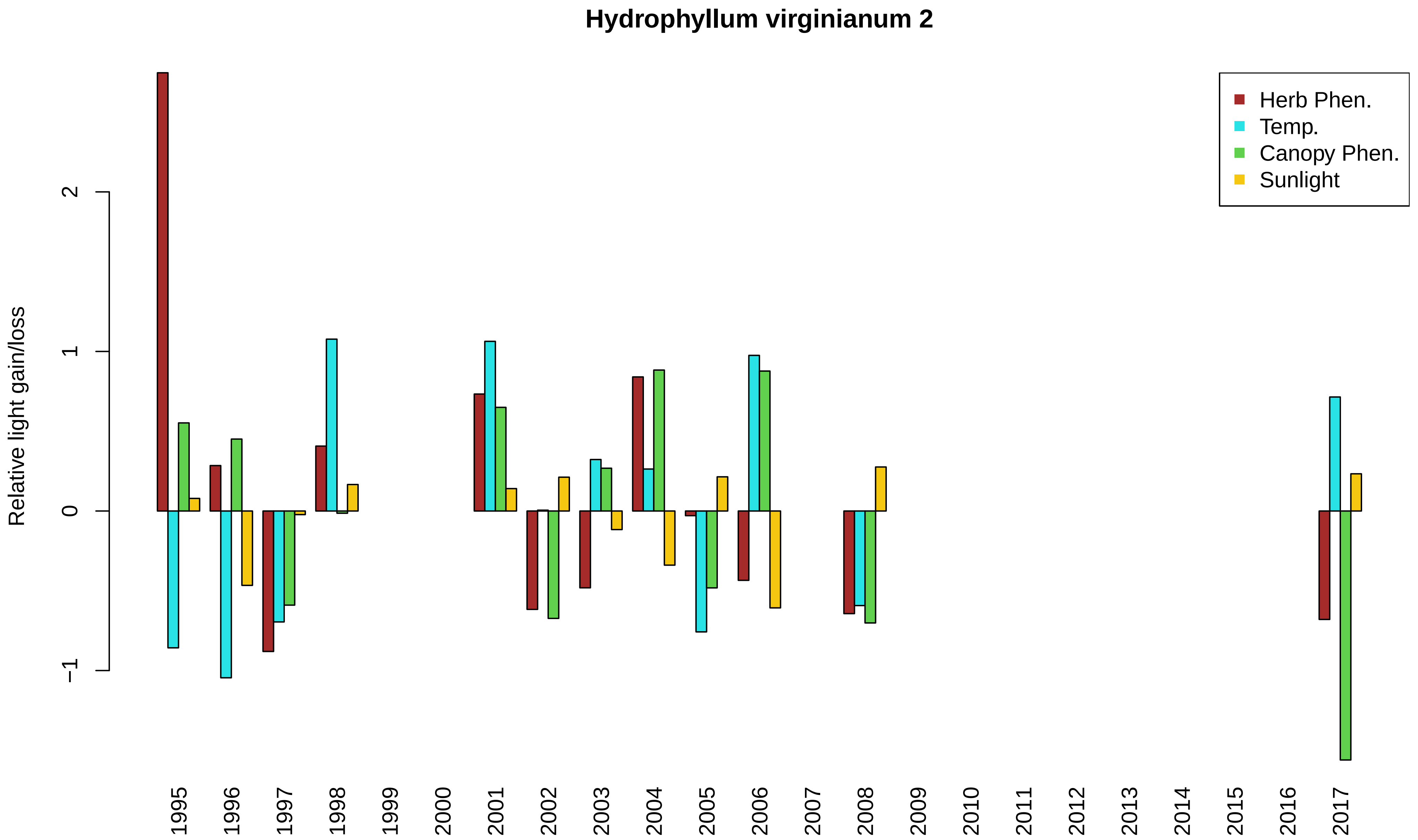
Hydrophyllum virginianum 2
Herb Phen.
Temp.
Canopy Phen.
Sunlight
Relative light gain/loss
2
1
0
−1
1995
1996
1997
1998
1999
2000
2001
2002
2003
2004
2005
2006
2007
2008
2009
2010
2011
2012
2013
2014
2015
2016
2017

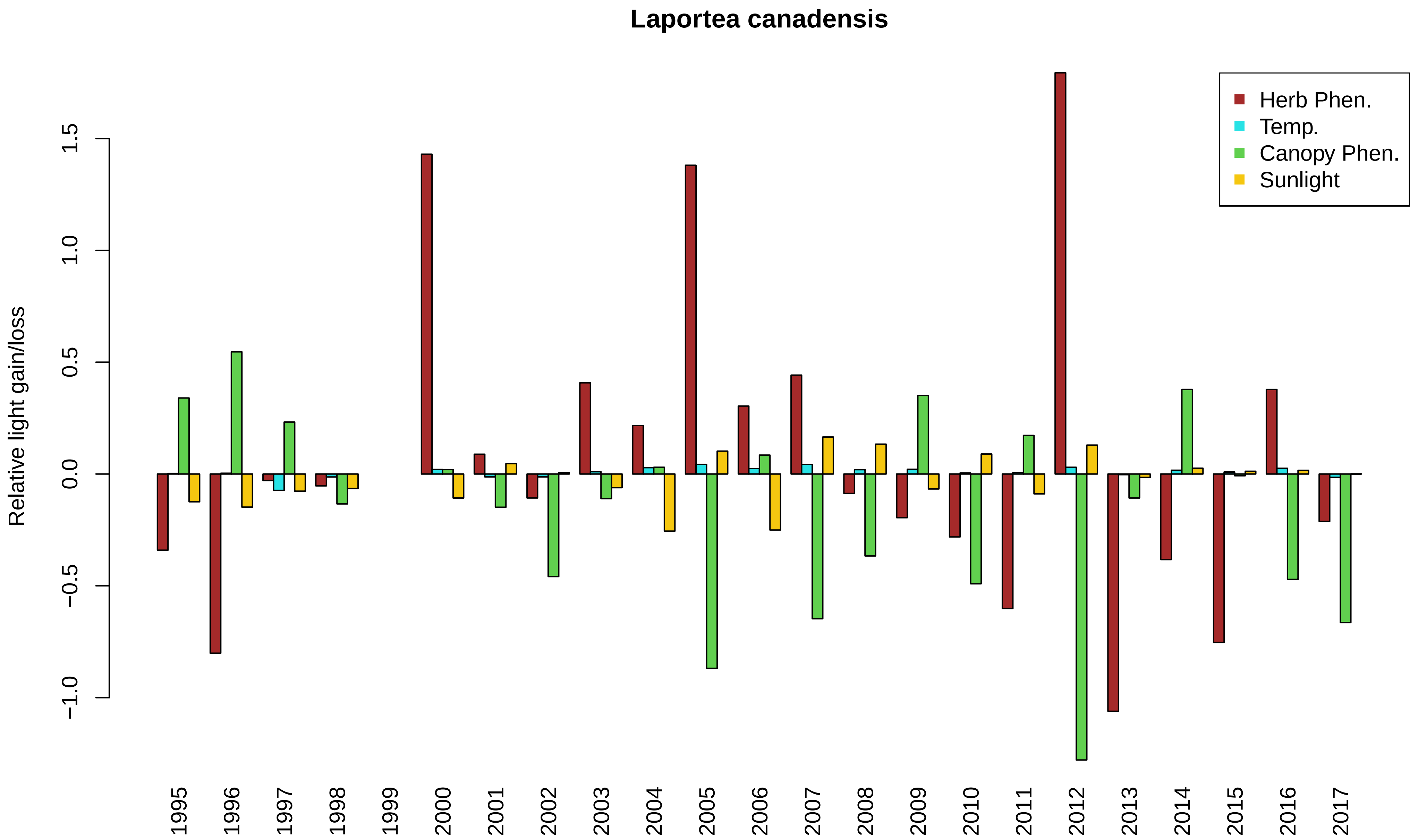

Laportea canadensis
Herb Phen.
Temp.
Canopy Phen.
Sunlight
Relative light gain/loss
1.5
1.0
0.5
0.0
−0.5
−1.0
1995
1996
1997
1998
1999
2000
2001
2002
2003
2004
2005
2006
2007
2008
2009
2010
2011
2012
2013
2014
2015
2016
2017

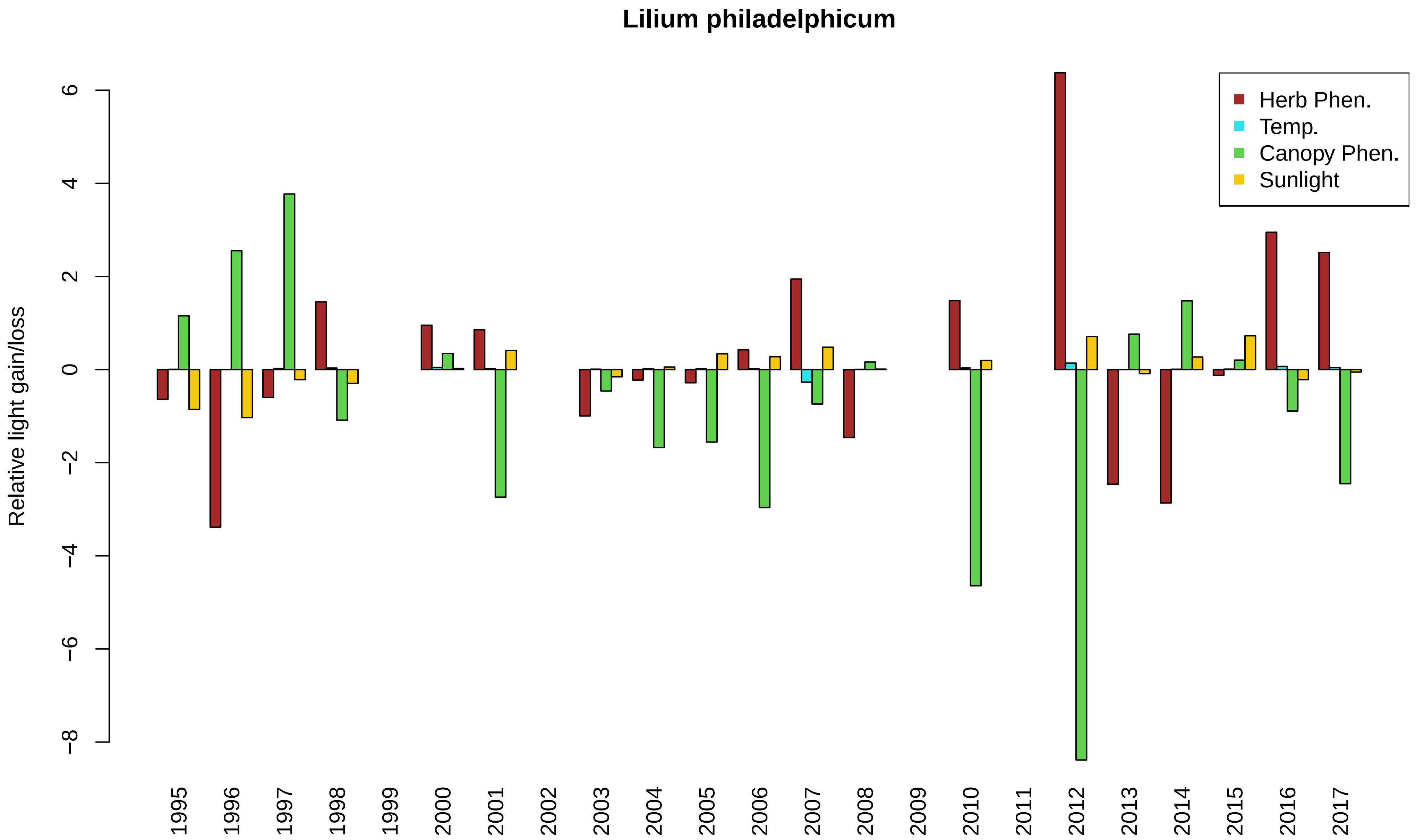
Lilium philadelphicum
Relative light gain/loss
6
4
2
0
−2
−4
−6
−8
Herb Phen.
Temp.
Canopy Phen.
Sunlight
1995
1996
1997
1998
1999
2000
2001
2002
2003
2004
2005
2006
2007
2008
2009
2010
2011
2012
2013
2014
2015
2016
2017

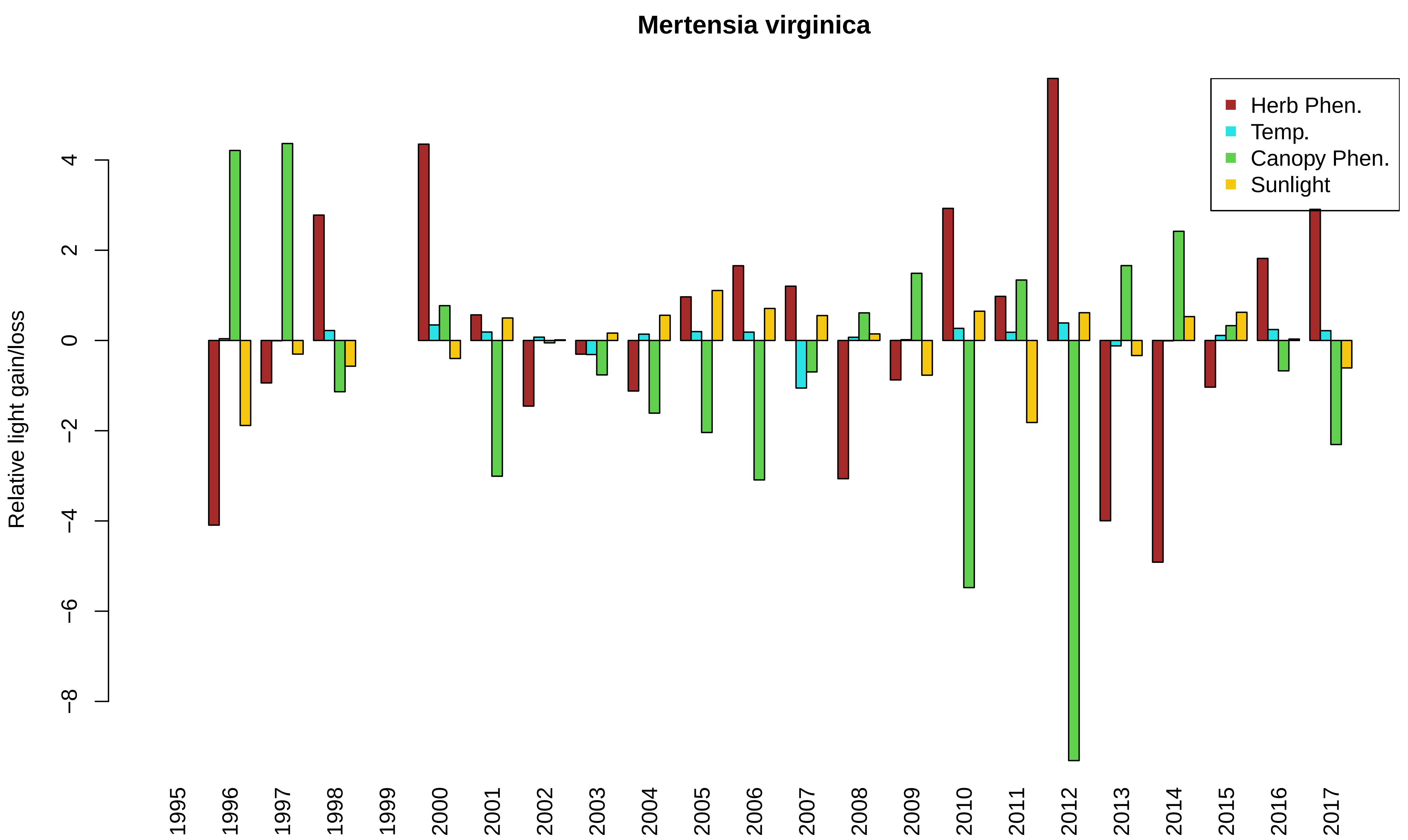
Mertensia virginica
Herb Phen.
Temp.
Canopy Phen.
Sunlight
Relative light gain/loss
4
2
0
−2
−4
−6
−8
1995
1996
1997
1998
1999
2000
2001
2002
2003
2004
2005
2006
2007
2008
2009
2010
2011
2012
2013
2014
2015
2016
2017

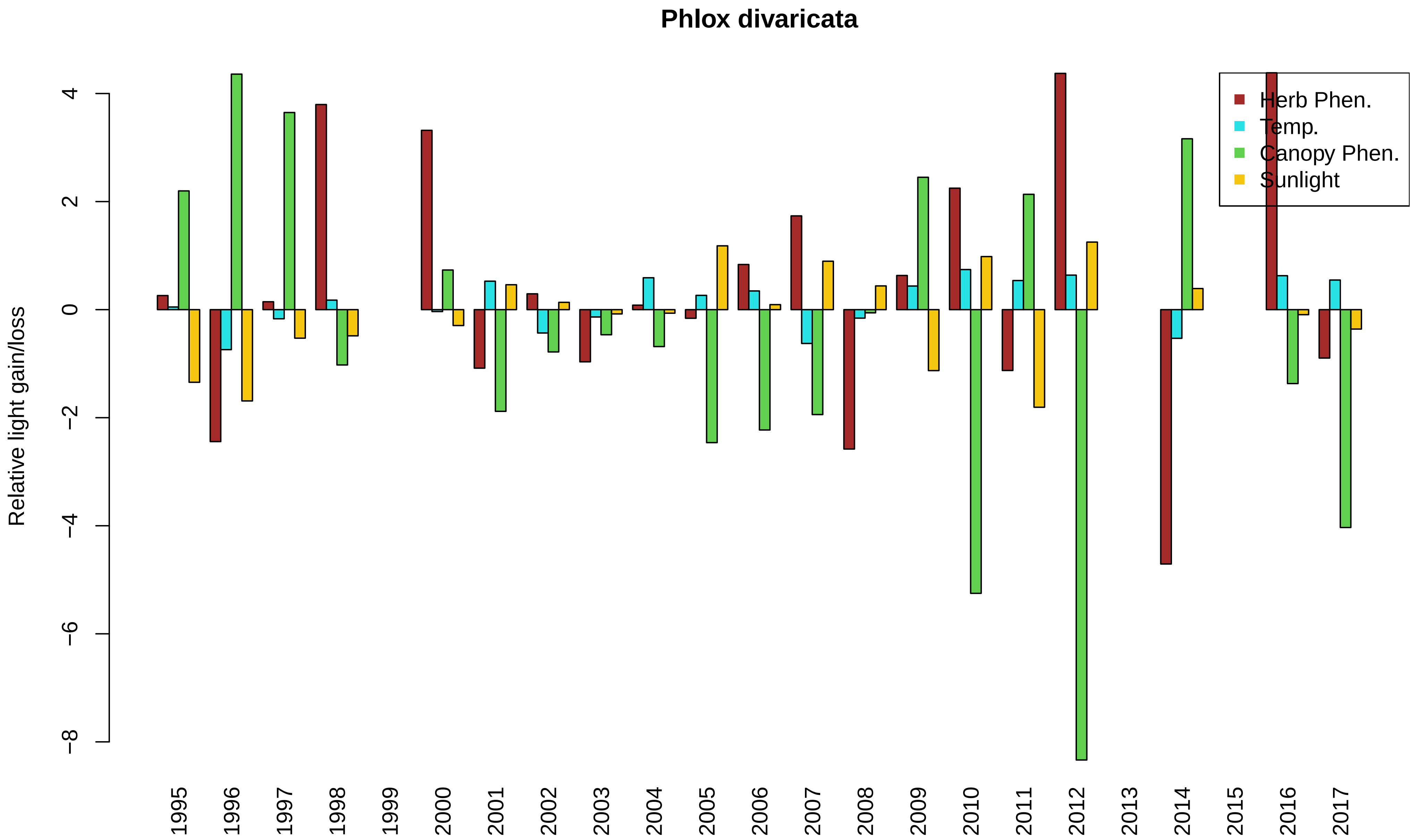

Phlox divaricata
Relative light gain/loss
4
2
0
−2
−4
−6
−8
Herb Phen.
Temp.
Canopy Phen.
Sunlight
1995
1996
1997
1998
1999
2000
2001
2002
2003
2004
2005
2006
2007
2008
2009
2010
2011
2012
2013
2014
2015
2016
2017

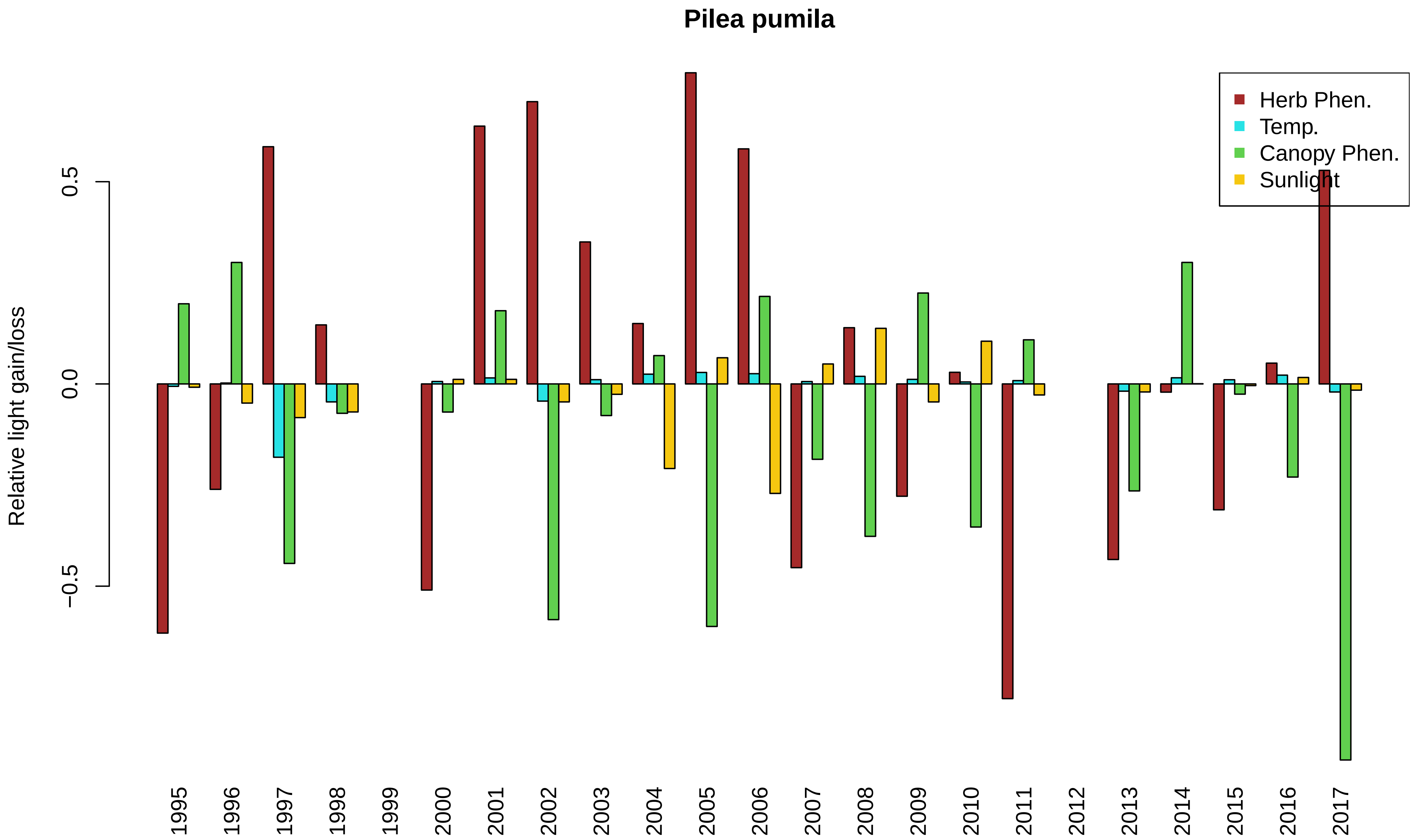

Pilea pumila
Herb Phen.
Temp.
Canopy Phen.
Sunlight
Relative light gain/loss
0.5
0.0
−0.5
1995
1996
1997
1998
1999
2000
2001
2002
2003
2004
2005
2006
2007
2008
2009
2010
2011
2012
2013
2014
2015
2016
2017

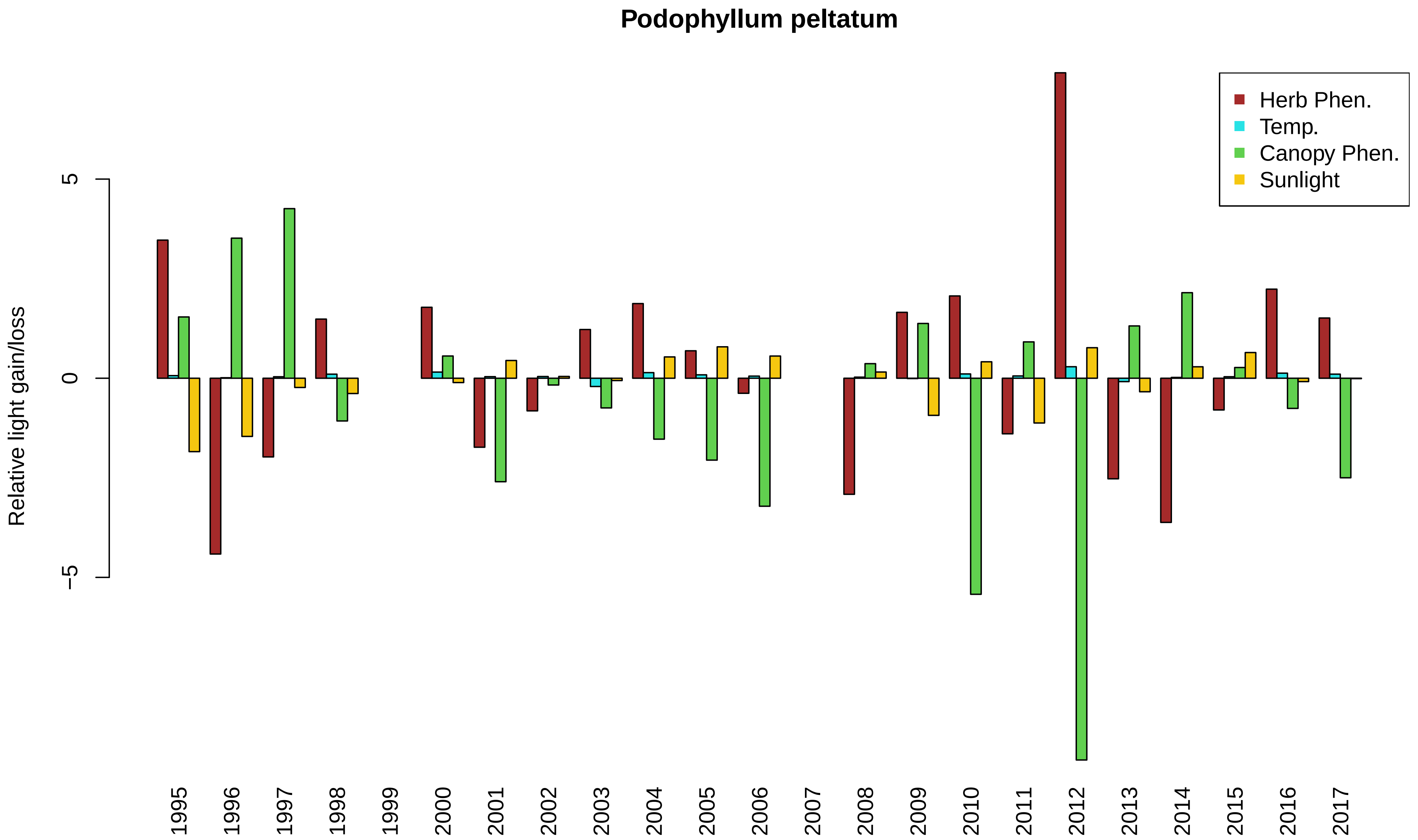

Podophyllum peltatum
Herb Phen.
Temp.
Canopy Phen.
Sunlight
Relative light gain/loss
5
0
−5
1995
1996
1997
1998
1999
2000
2001
2002
2003
2004
2005
2006
2007
2008
2009
2010
2011
2012
2013
2014
2015
2016
2017

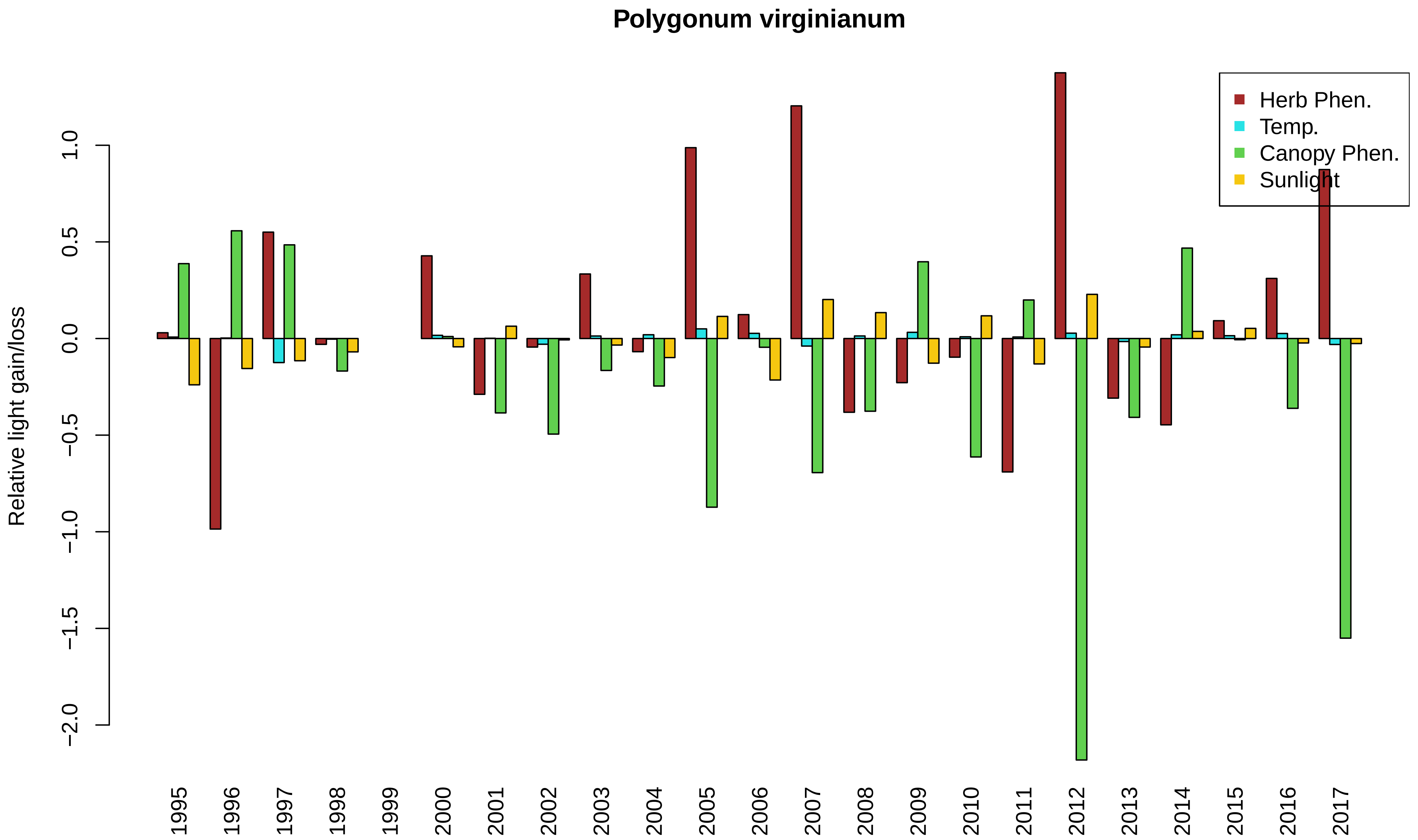
Polygonum virginianum
Herb Phen.
Temp.
Canopy Phen.
Sunlight
Relative light gain/loss
1.0
0.5
0.0
−0.5
−1.0
−1.5
−2.0
1995
1996
1997
1998
1999
2000
2001
2002
2003
2004
2005
2006
2007
2008
2009
2010
2011
2012
2013
2014
2015
2016
2017

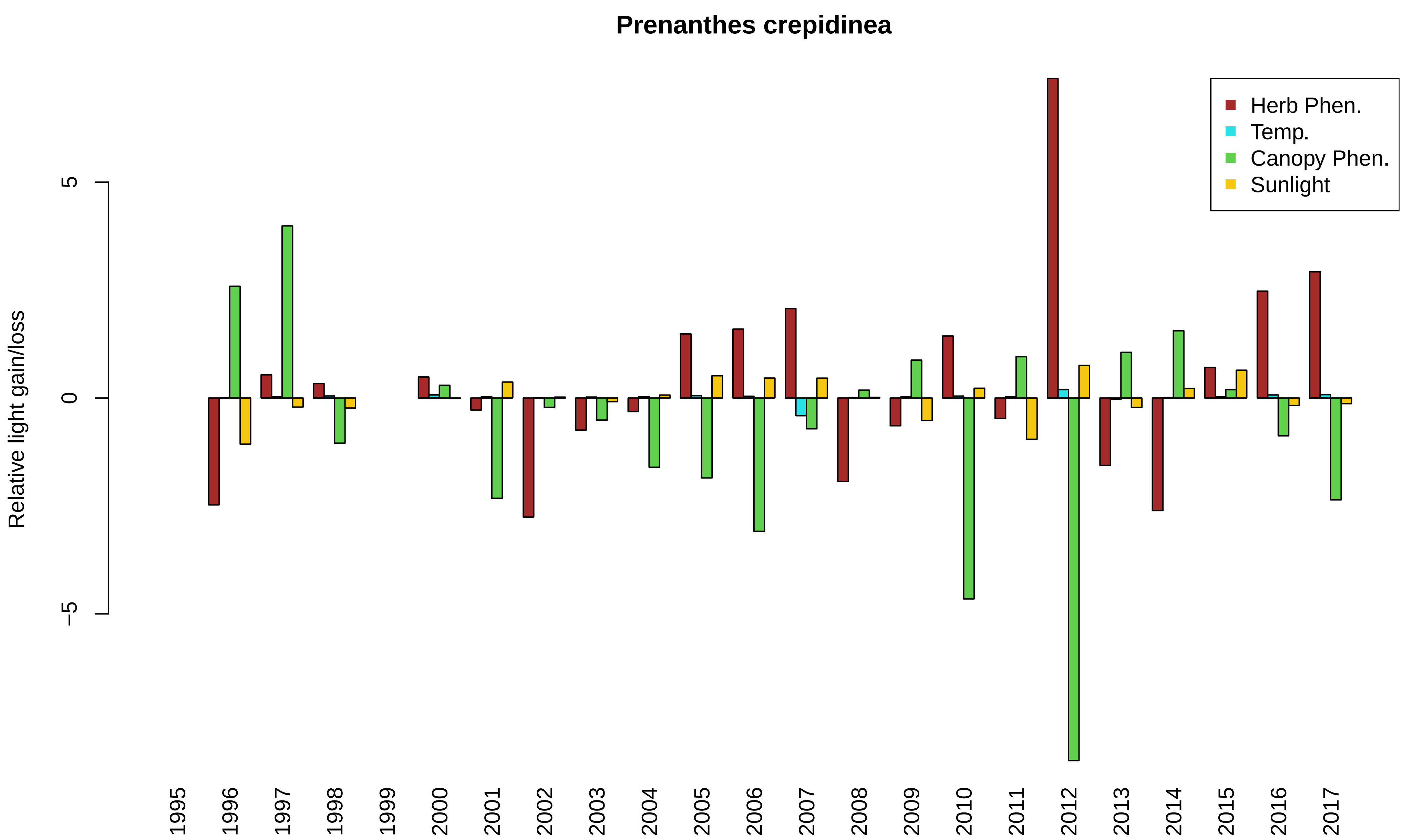

Prenanthes crepidinea
Herb Phen.
Temp.
Canopy Phen.
Sunlight
Relative light gain/loss
5
0
−5
1995
1996
1997
1998
1999
2000
2001
2002
2003
2004
2005
2006
2007
2008
2009
2010
2011
2012
2013
2014
2015
2016
2017

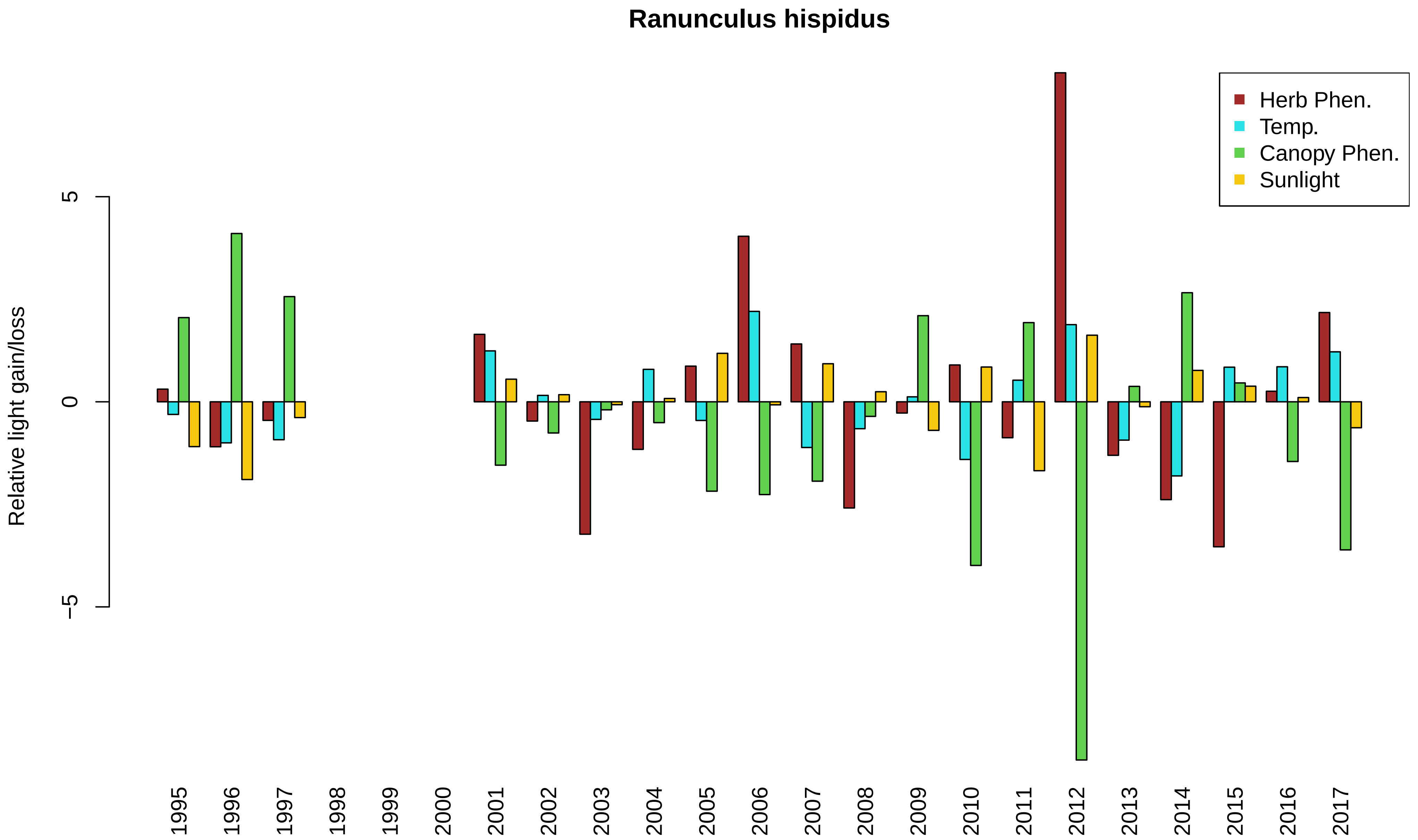
Ranunculus hispidus
Herb Phen.
Temp.
Canopy Phen.
Sunlight
Relative light gain/loss
5
0
−5
1995
1996
1997
1998
1999
2000
2001
2002
2003
2004
2005
2006
2007
2008
2009
2010
2011
2012
2013
2014
2015
2016
2017

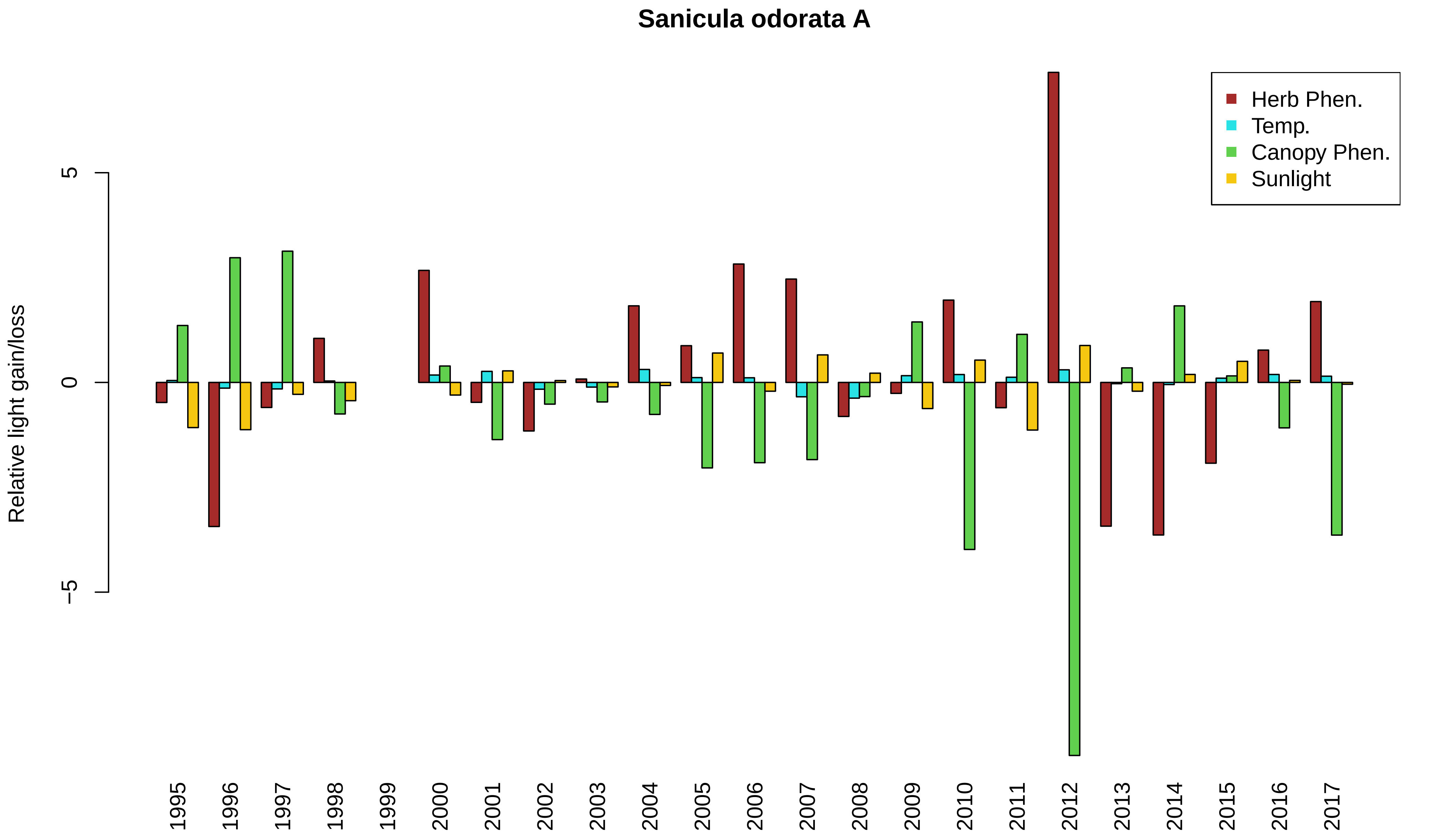

Sanicula odorata A
Herb Phen.
Temp.
Canopy Phen.
Sunlight
Relative light gain/loss
5
0
−5
1995
1996
1997
1998
1999
2000
2001
2002
2003
2004
2005
2006
2007
2008
2009
2010
2011
2012
2013
2014
2015
2016
2017

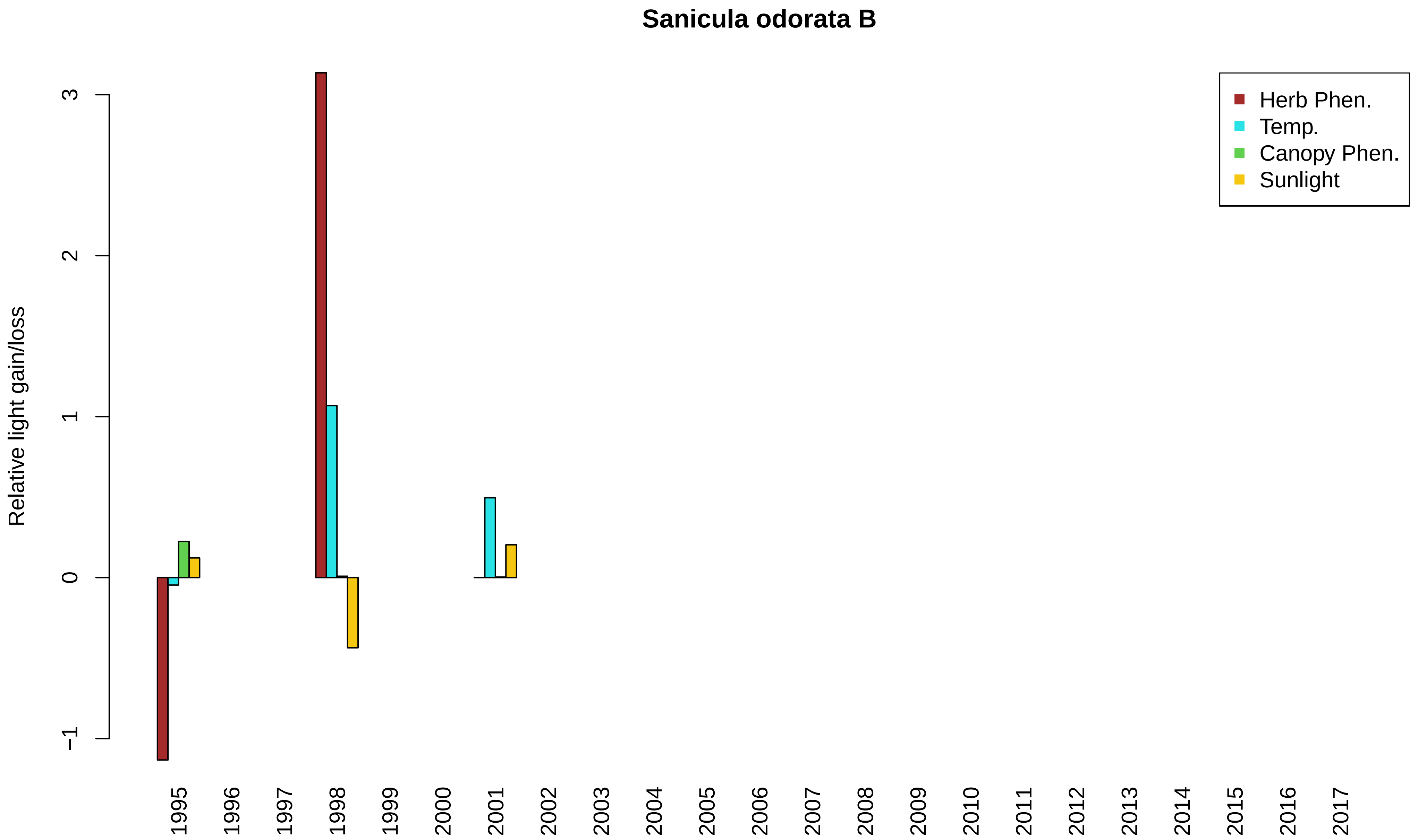

Sanicula odorata B
Relative light gain/loss
3
2
1
0
−1
1995
1996
1997
1998
1999
2000
2001
2002
2003
2004
2005
2006
2007
2008
2009
2010
2011
2012
2013
2014
2015
2016
2017
Herb Phen.
Temp.
Canopy Phen.
Sunlight

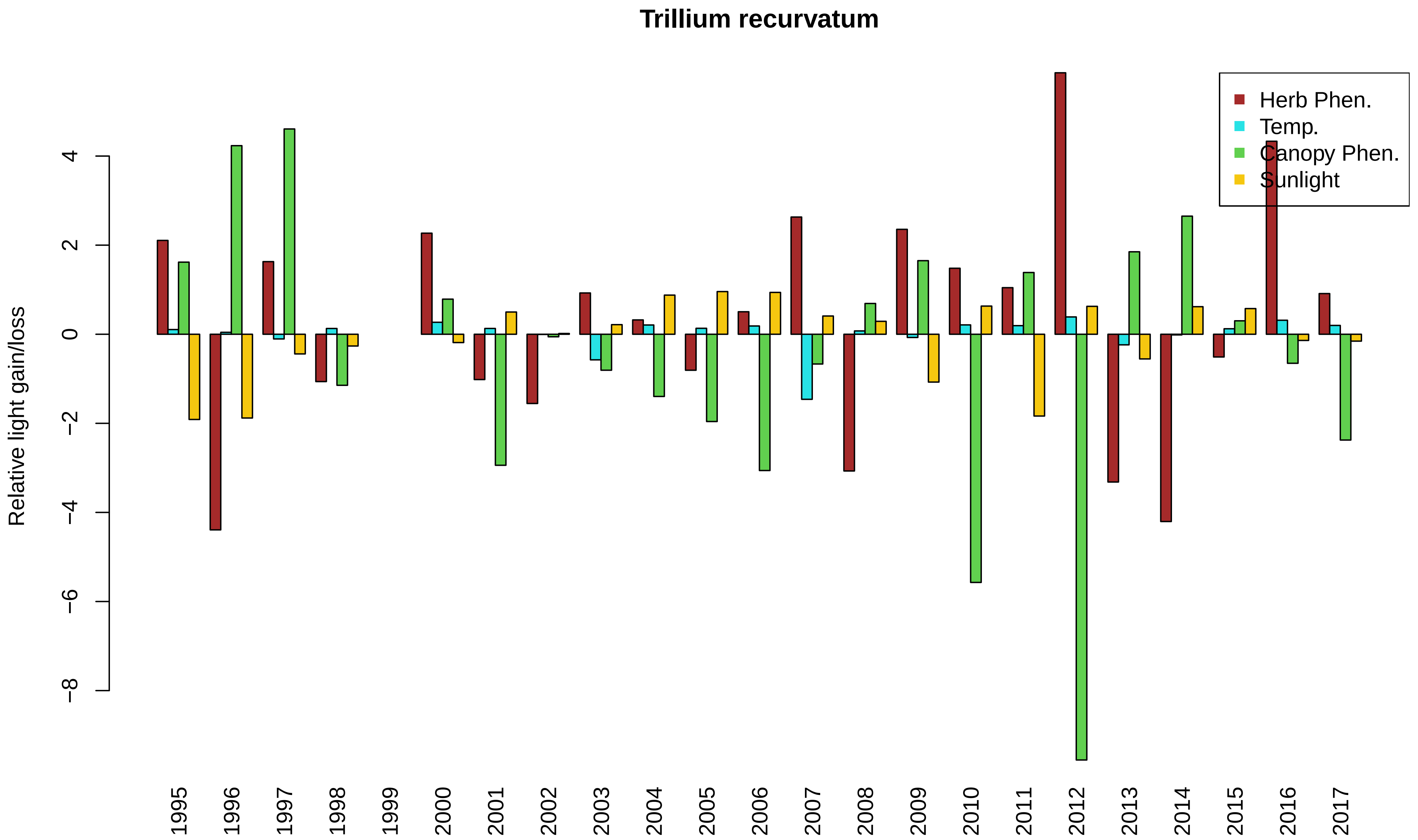
Trillium recurvatum
Herb Phen.
Temp.
Canopy Phen.
Sunlight
Relative light gain/loss
4
2
0
−2
−4
−6
−8
1995
1996
1997
1998
1999
2000
2001
2002
2003
2004
2005
2006
2007
2008
2009
2010
2011
2012
2013
2014
2015
2016
2017

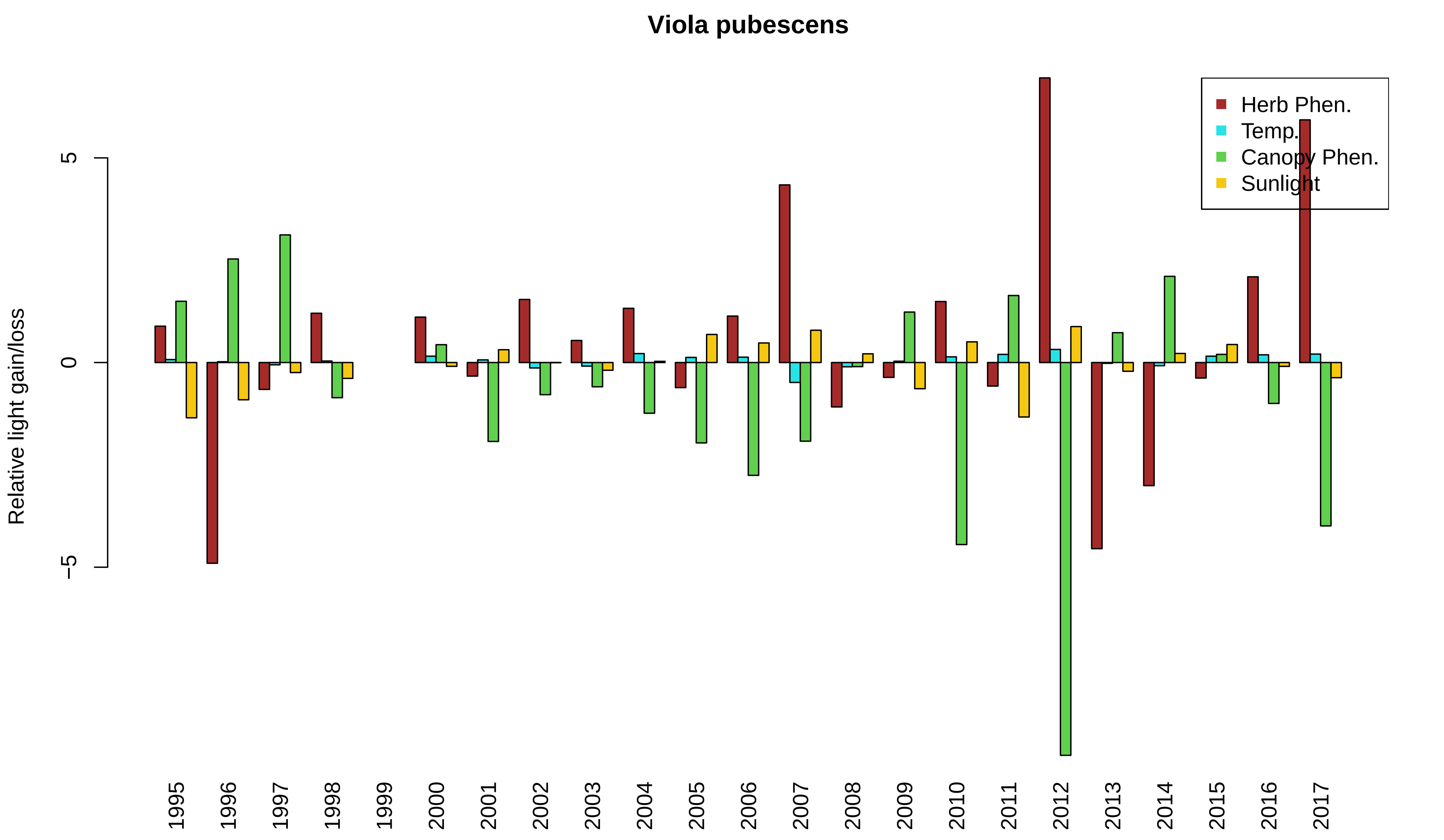
Viola pubescens
Herb Phen.
Temp.
Canopy Phen.
Sunlight
Relative light gain/loss
5
0
−5
1995
1996
1997
1998
1999
2000
2001
2002
2003
2004
2005
2006
2007
2008
2009
2010
2011
2012
2013
2014
2015
2016
2017

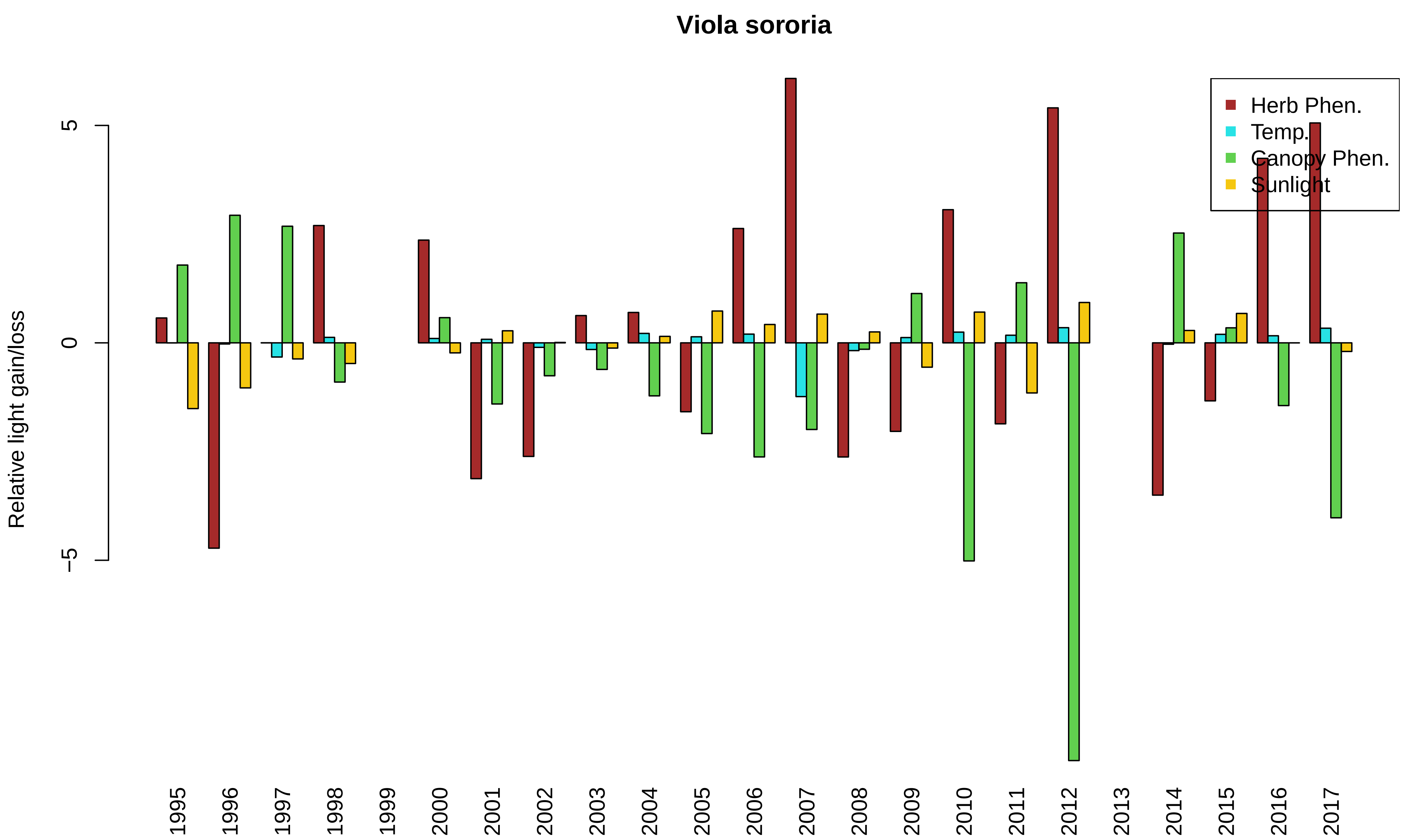

Viola sororia
Herb Phen.
Temp.
Canopy Phen.
Sunlight
Relative light gain/loss
5
0
−5
1995
1996
1997
1998
1999
2000
2001
2002
2003
2004
2005
2006
2007
2008
2009
2010
2011
2012
2013
2014
2015
2016
2017
