# Supplementary material for: Understory plants evade shading in a temperate deciduous forest amid climate variability by shifting phenology in synchrony with canopy trees
Source: PLoS One. 2024 Jun 26;19(6):e0306023. doi: 10.1371/journal.pone.0306023 (PMC11207122; doi:10.1371/journal.pone.0306023)
Supplement: S3 Fig — The y-axis units are relative measures of light interception, and best used for comparisons within species (see Methods: Section 4). Solid lines indicate a factor has a statistically-significant (p < .05) difference of its estimated slope from 0, while dashed lines indicate that this standard was not met. (PDF) [file pone.0306023.s009.pdf]

Supporting Information 9 for Augspurger CK, Salk CF. Understory plants reduce light loss in a temperate deciduous forest amid climate variability by shifting phenology in synchrony with canopy trees. PLoS One. In review.

Supporting Information 9:
Trends in total light interception by sapling species over time, and relative contribution to that trend of sapling phenology, temperature, canopy phenology and solar radiation. The y−axis units are relative measures of light interception, and best used for comparisons within species (see Methods: Section 4). Solid lines indicate a factor has a statistically−significant ($p<.05$) difference of its estimated slope from 0, while dashed lines indicate that this standard was not met.

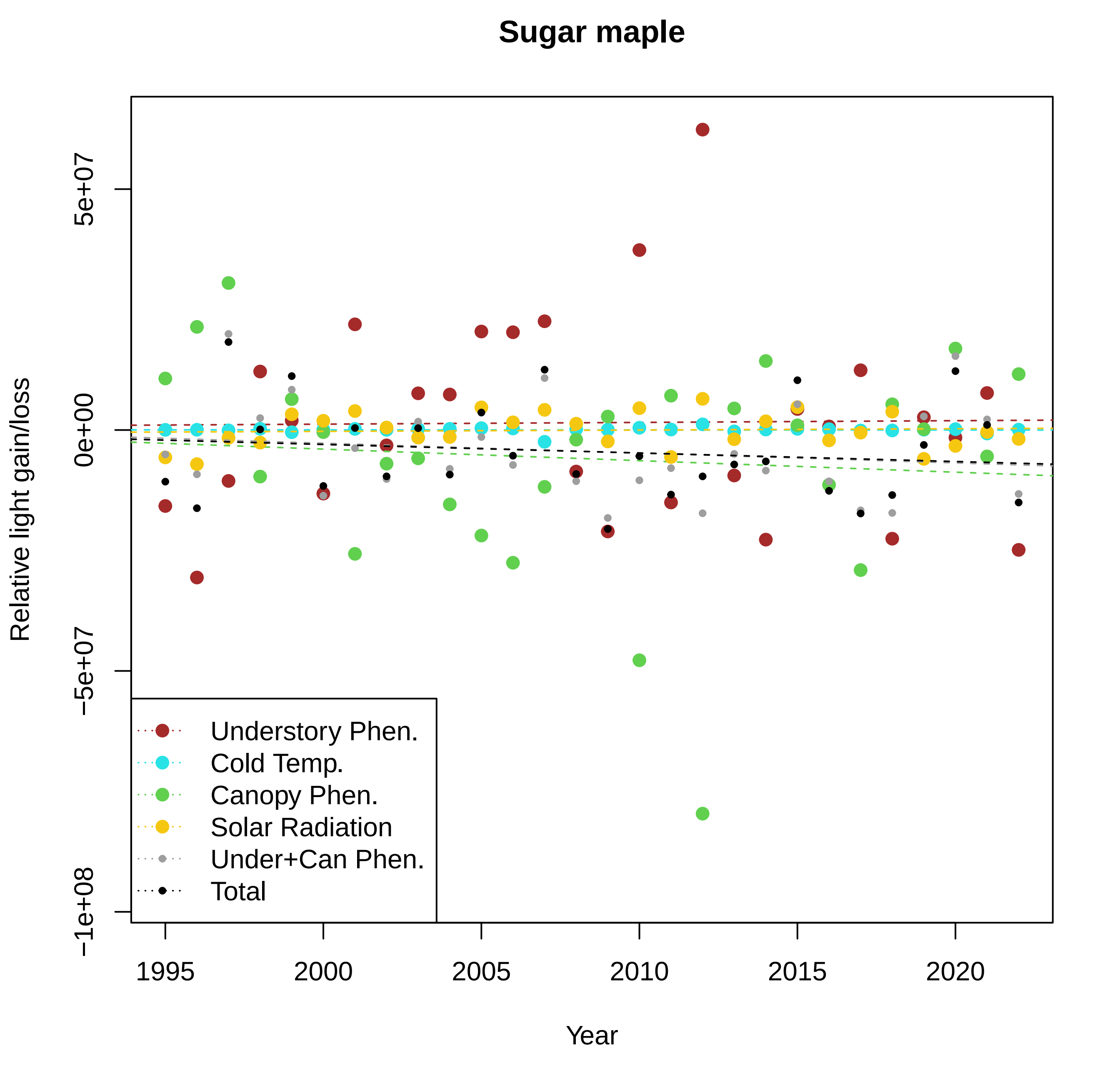

Sugar maple
Relative light gain/loss
5e+07
0e+00
−5e+07
−1e+08
1995
2000
2005
2010
2015
2020
Year
Understory Phen.
Cold Temp.
Canopy Phen.
Solar Radiation
Under+Can Phen.
Total

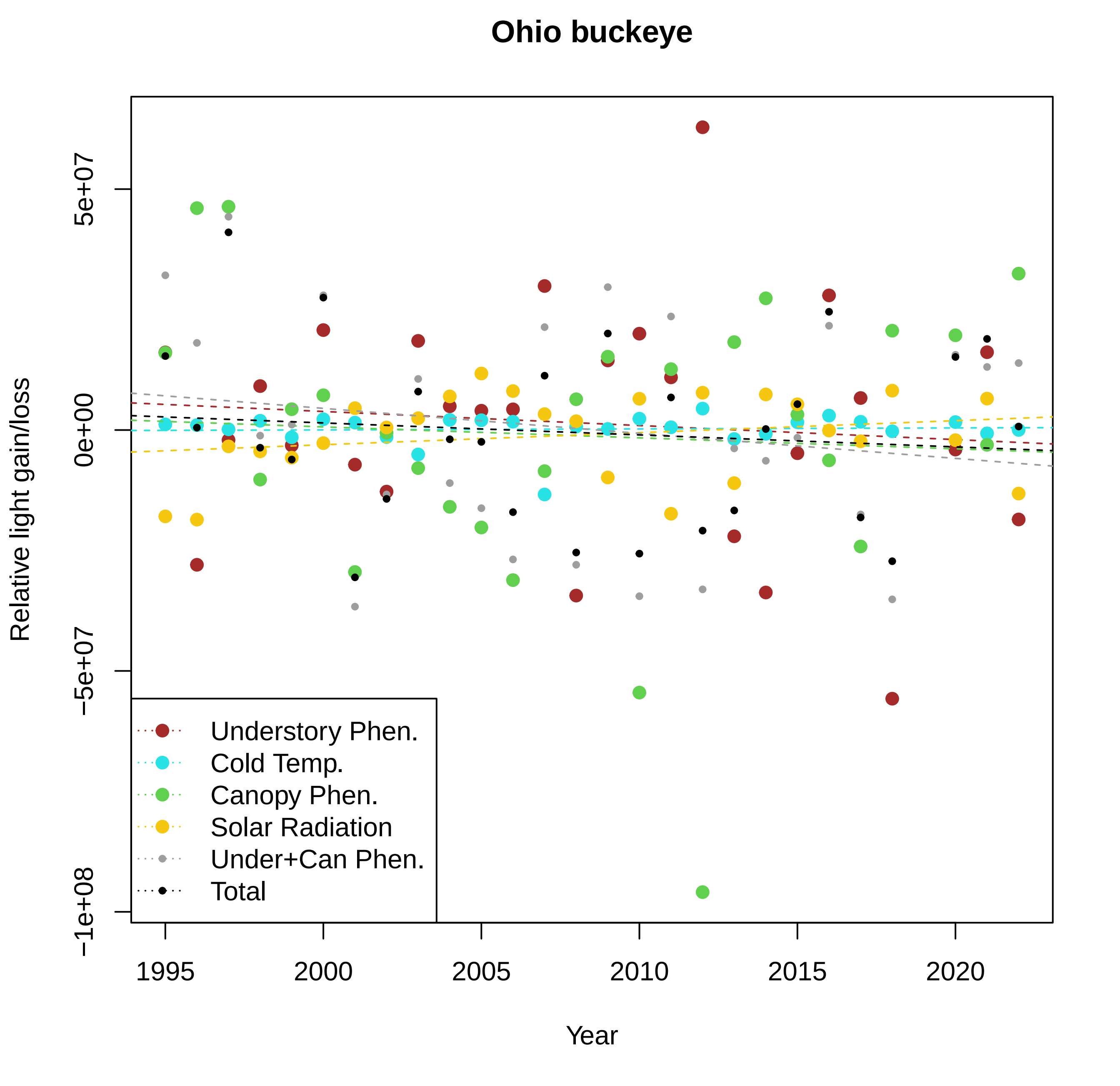

Ohio buckeye
Relative light gain/loss
5e+07
0e+00
−5e+07
−1e+08
1995
2000
2005
2010
2015
2020
Year
Understory Phen.
Cold Temp.
Canopy Phen.
Solar Radiation
Under+Can Phen.
Total

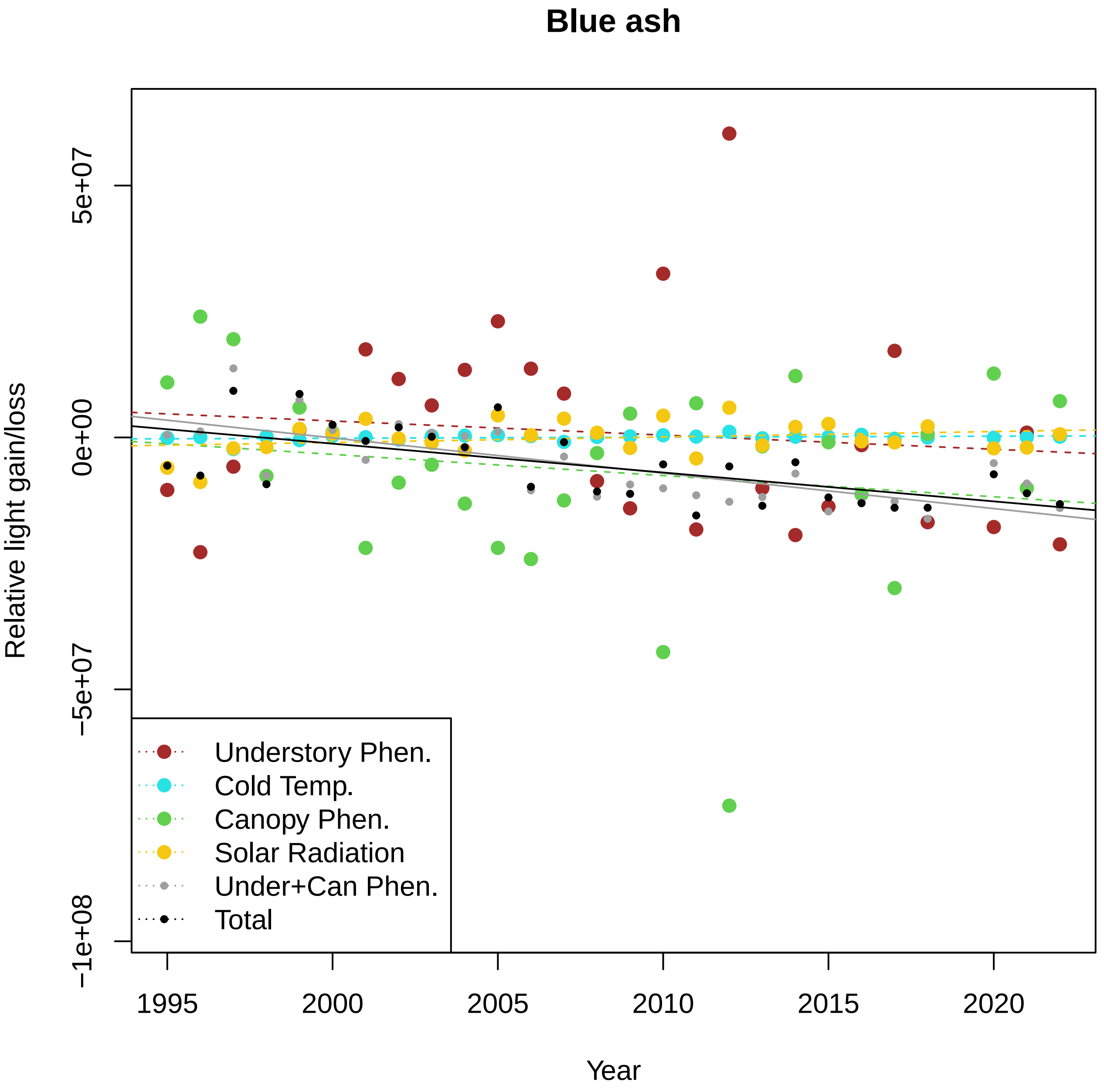
Blue ash
Relative light gain/loss
5e+07
0e+00
−5e+07
−1e+08
1995
2000
2005
2010
2015
2020
Year
Understory Phen.
Cold Temp.
Canopy Phen.
Solar Radiation
Under+Can Phen.
Total
