# Supplementary material for: Understory plants evade shading in a temperate deciduous forest amid climate variability by shifting phenology in synchrony with canopy trees
Source: PLoS One. 2024 Jun 26;19(6):e0306023. doi: 10.1371/journal.pone.0306023 (PMC11207122; doi:10.1371/journal.pone.0306023)
Supplement: S4 Fig — The y-axis units are relative measures of light interception, and best used for comparisons within species (see Methods: Section 4). Solid lines indicate a factor has a statistically-significant (p < .05) difference of its estimated slope from 0, while dashed lines indicate that this standard was not met. (PDF) [file pone.0306023.s010.pdf]

Supporting Information 10 for Augspurger CK, Salk CF. Understory plants reduce light loss in a temperate deciduous forest amid climate variability by shifting phenology in synchrony with canopy trees. PLoS One. In review.

Supporting Information 10:
Trends in total light interception by herb species over time, and relative contribution to that trend of herb phenology, temperature, canopy phenology and solar radiation. The y−axis units are relative measures of light interception, and best used for comparisons within species (see Methods: Section 4). Solid lines indicate a factor has a statistically−significant ($p<.05$) difference of its estimated slope from 0, while dashed lines indicate that this standard was not met.

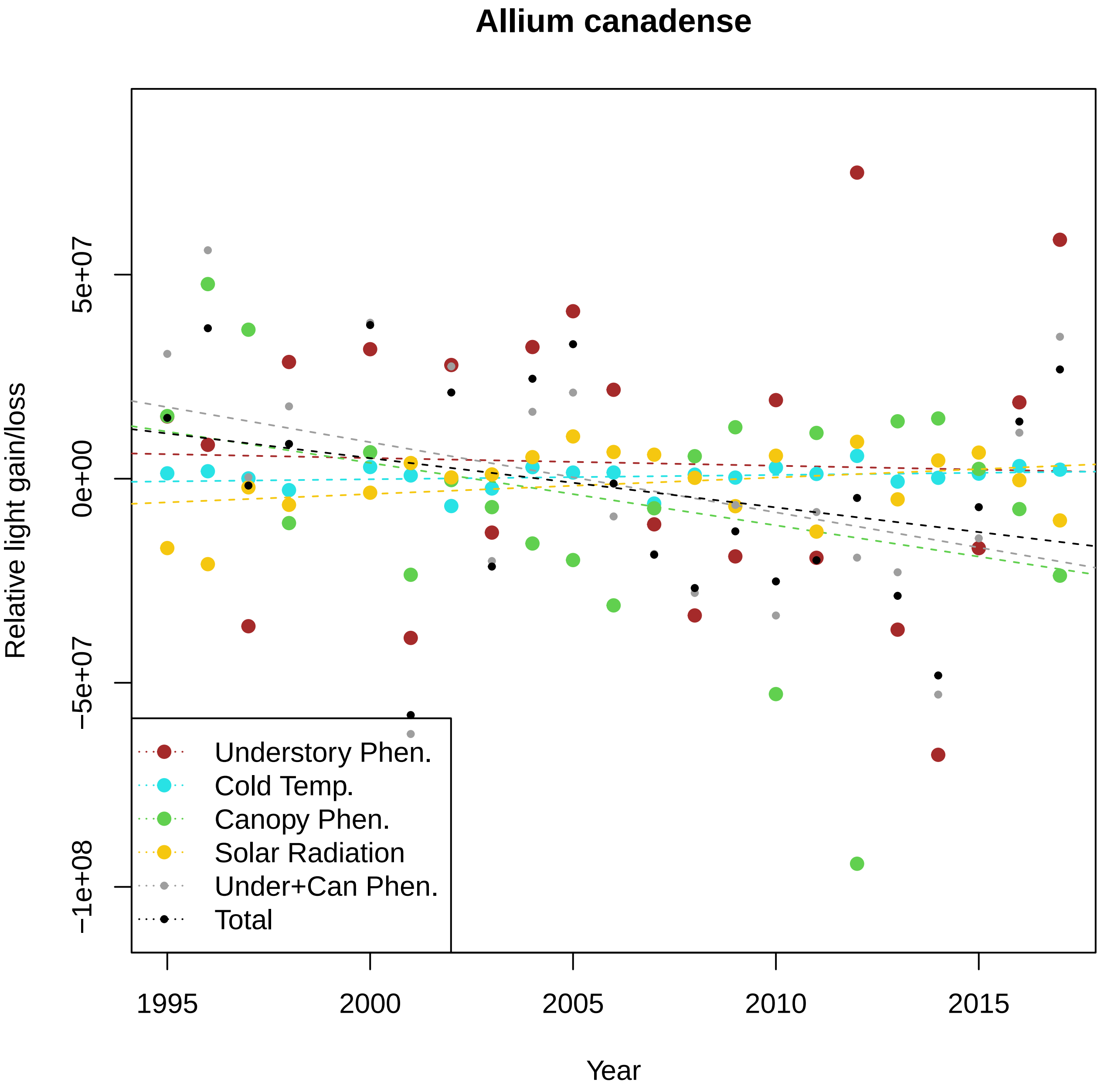

Allium canadense
Relative light gain/loss
5e+07
0e+00
−5e+07
−1e+08
1995
2000
2005
2010
2015
Year
Understory Phen.
Cold Temp.
Canopy Phen.
Solar Radiation
Under+Can Phen.
Total

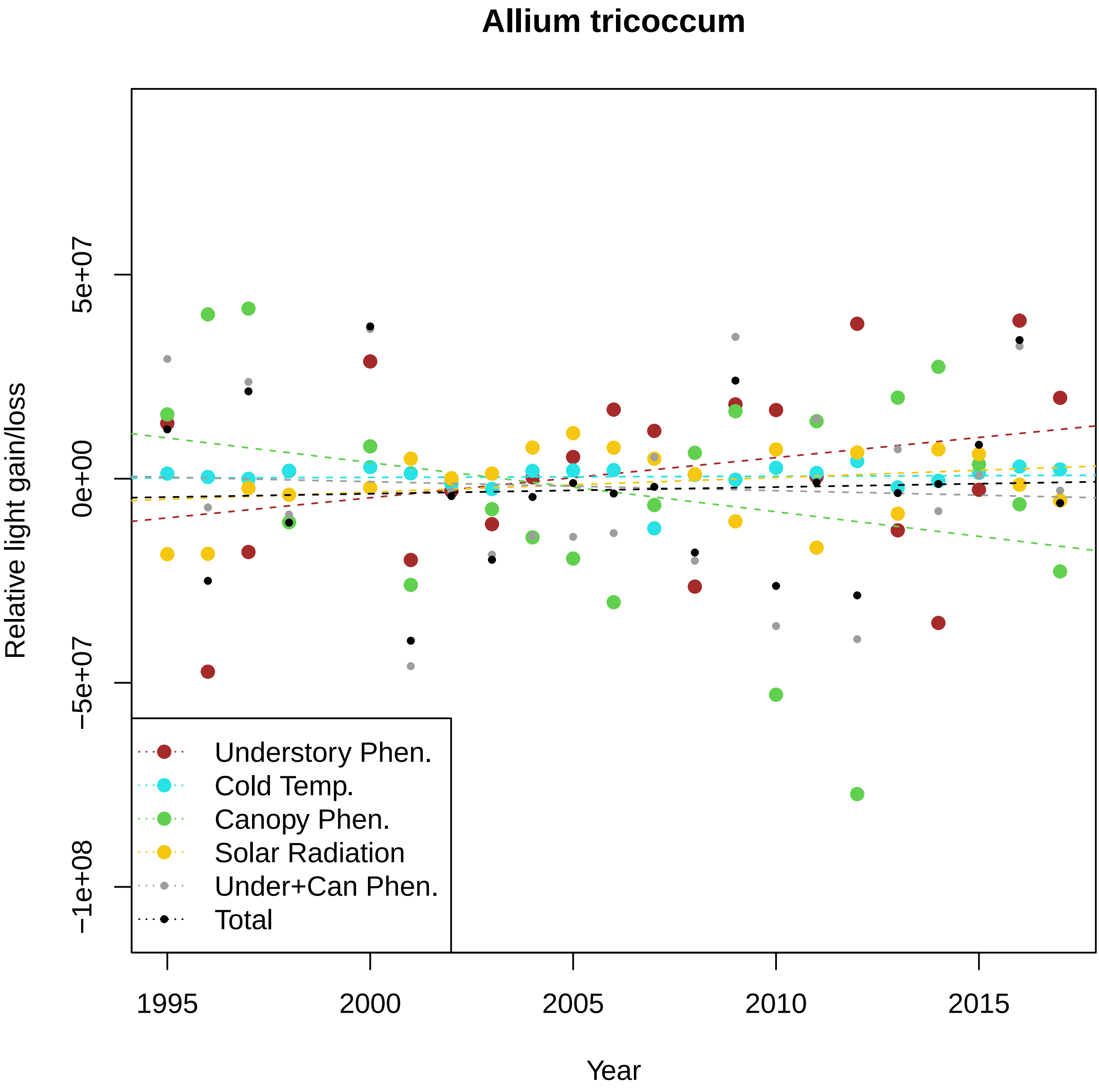
Allium tricoccum
Relative light gain/loss
5e+07
0e+00
−5e+07
−1e+08
1995
2000
2005
2010
2015
Year
Understory Phen.
Cold Temp.
Canopy Phen.
Solar Radiation
Under+Can Phen.
Total

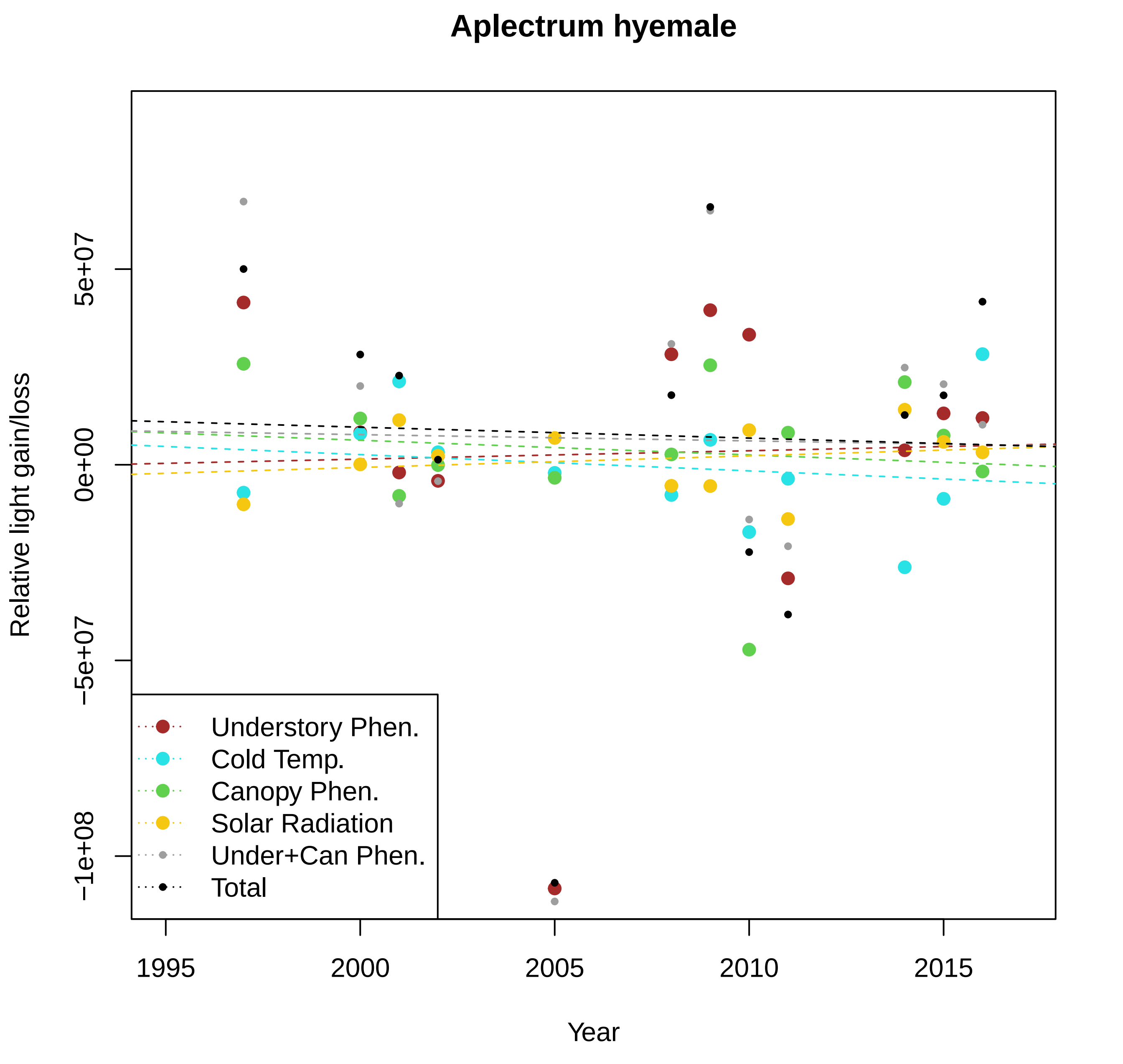
Aplectrum hyemale
Relative light gain/loss
5e+07
0e+00
−5e+07
−1e+08
1995
2000
2005
2010
2015
Year
Understory Phen.
Cold Temp.
Canopy Phen.
Solar Radiation
Under+Can Phen.
Total

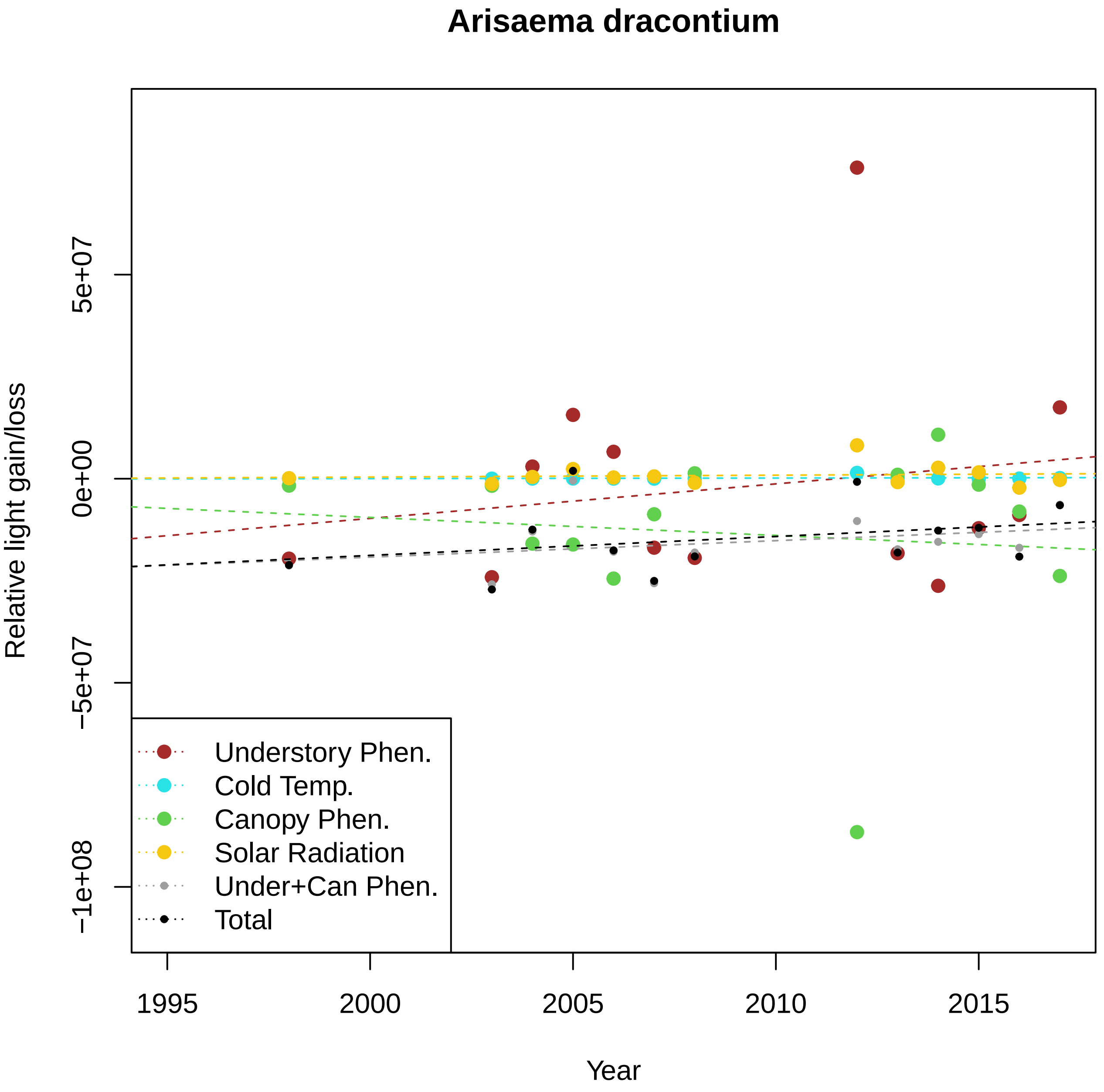

Arisaema dracontium
Relative light gain/loss
5e+07
0e+00
−5e+07
−1e+08
1995
2000
2005
2010
2015
Year
Understory Phen.
Cold Temp.
Canopy Phen.
Solar Radiation
Under+Can Phen.
Total

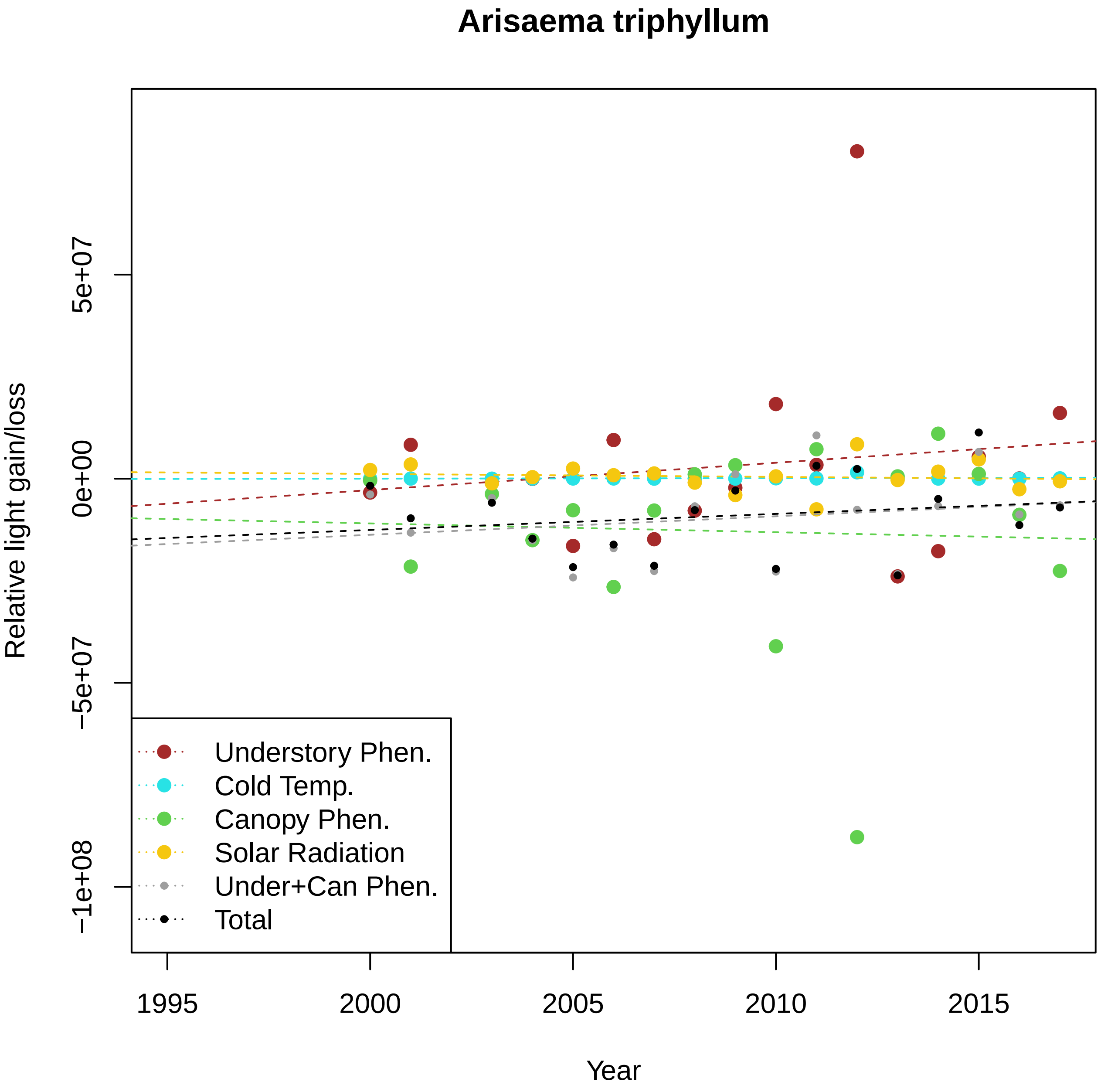

Arisaema triphyllum
Relative light gain/loss
5e+07
0e+00
−5e+07
−1e+08
1995
2000
2005
2010
2015
Year
Understory Phen.
Cold Temp.
Canopy Phen.
Solar Radiation
Under+Can Phen.
Total

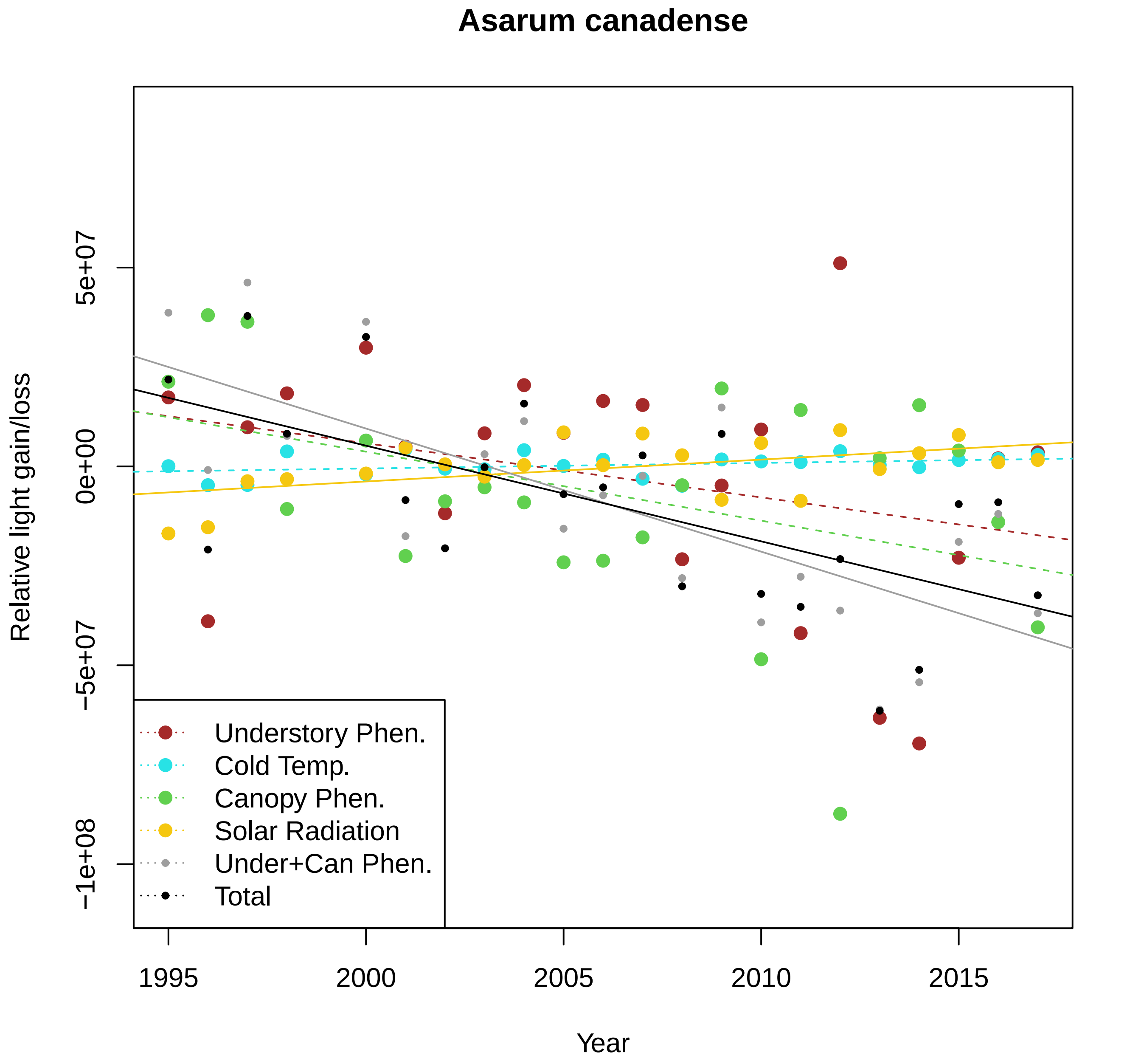

Asarum canadense
Relative light gain/loss
5e+07
0e+00
−5e+07
−1e+08
1995
2000
2005
2010
2015
Year
Understory Phen.
Cold Temp.
Canopy Phen.
Solar Radiation
Under+Can Phen.
Total

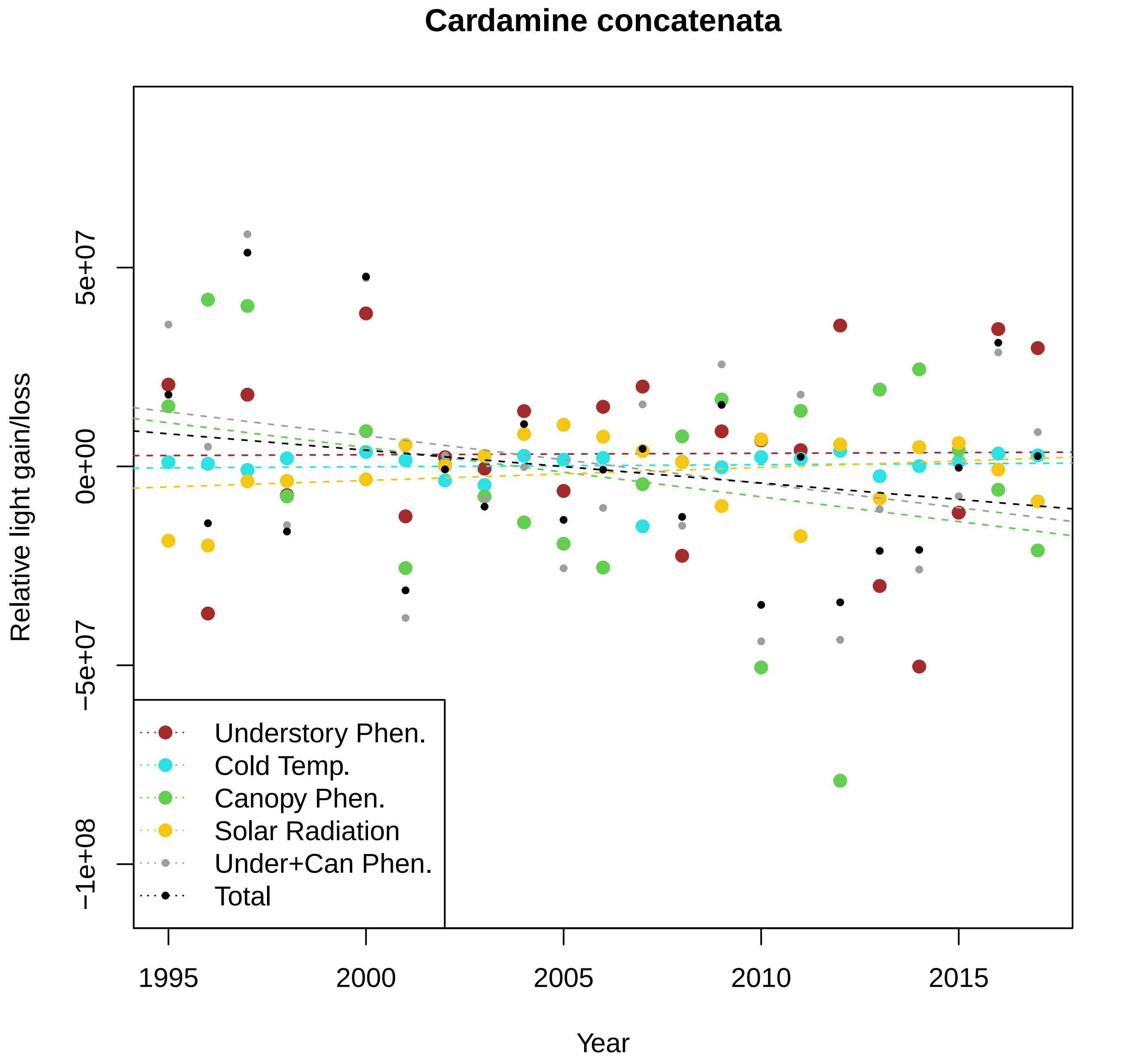

Cardamine concatenata
Relative light gain/loss
5e+07
0e+00
−5e+07
−1e+08
1995
2000
2005
2010
2015
Year
Understory Phen.
Cold Temp.
Canopy Phen.
Solar Radiation
Under+Can Phen.
Total

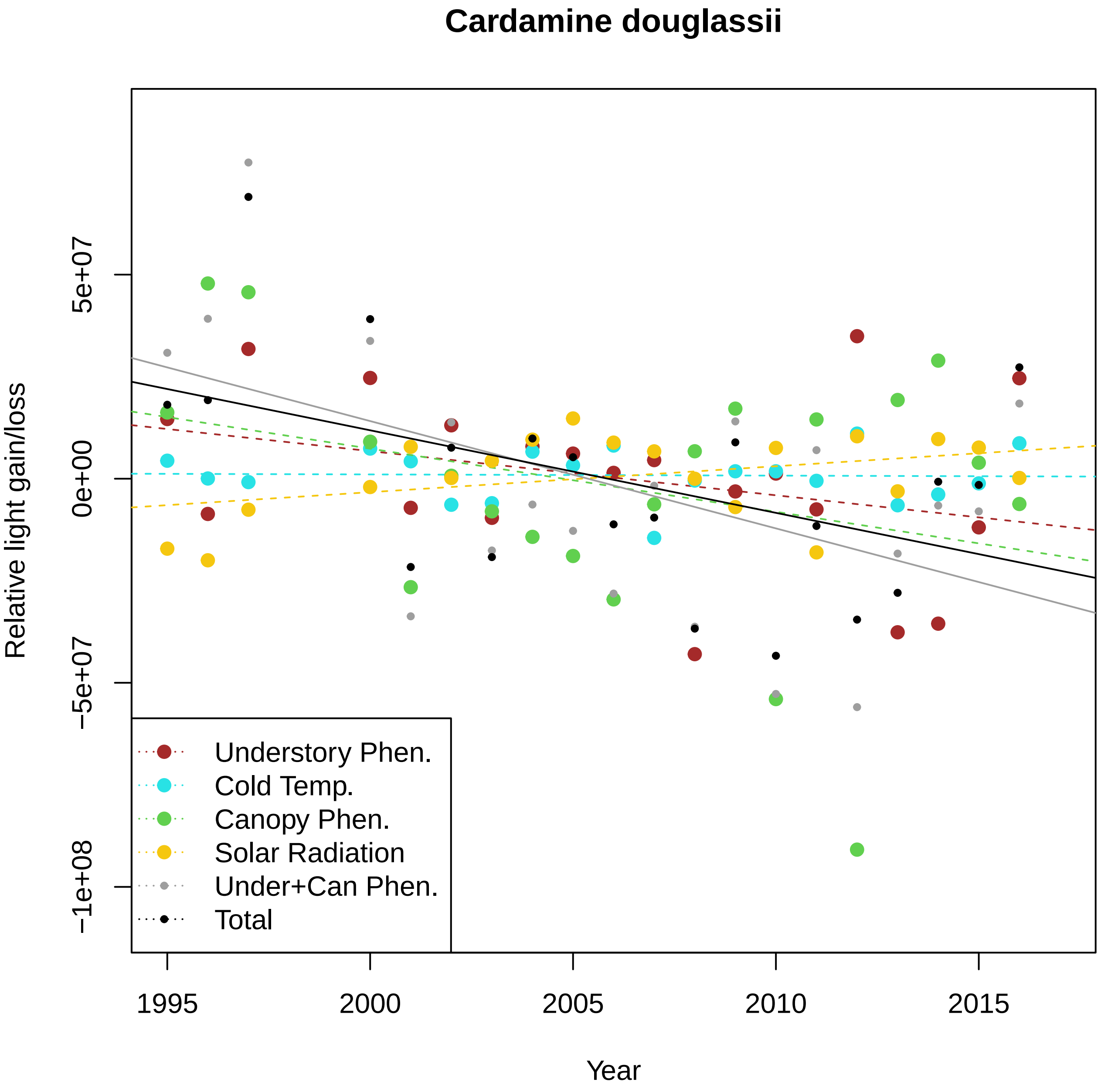

Cardamine douglassii
Relative light gain/loss
Year
5e+07
0e+00
−5e+07
−1e+08
1995
2000
2005
2010
2015
Understory Phen.
Cold Temp.
Canopy Phen.
Solar Radiation
Under+Can Phen.
Total

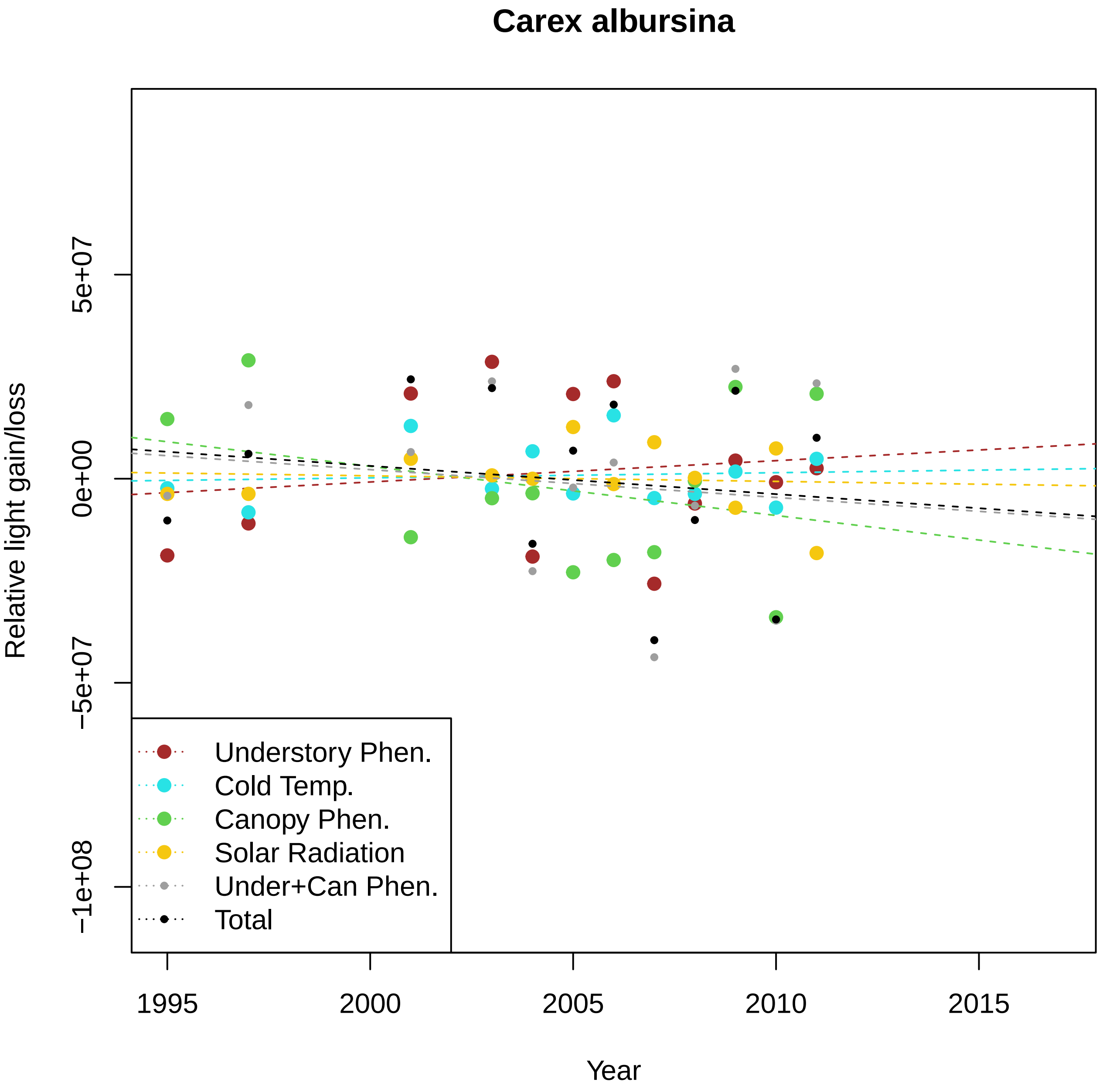

Carex albursina
Relative light gain/loss
5e+07
0e+00
−5e+07
−1e+08
1995
2000
2005
2010
2015
Year
Understory Phen.
Cold Temp.
Canopy Phen.
Solar Radiation
Under+Can Phen.
Total

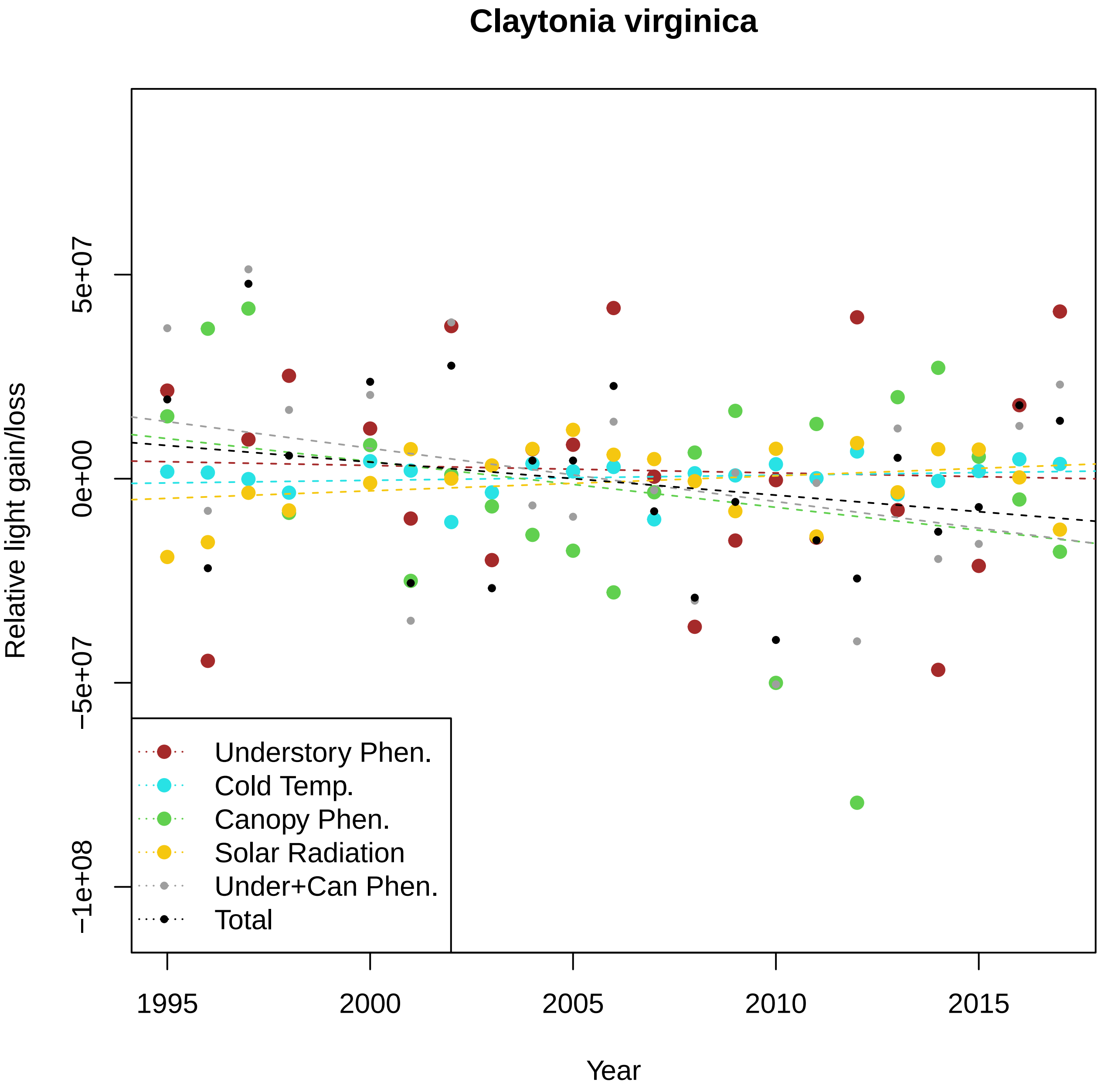

Claytonia virginica
Relative light gain/loss
5e+07
0e+00
−5e+07
−1e+08
1995
2000
2005
2010
2015
Year
Understory Phen.
Cold Temp.
Canopy Phen.
Solar Radiation
Under+Can Phen.
Total

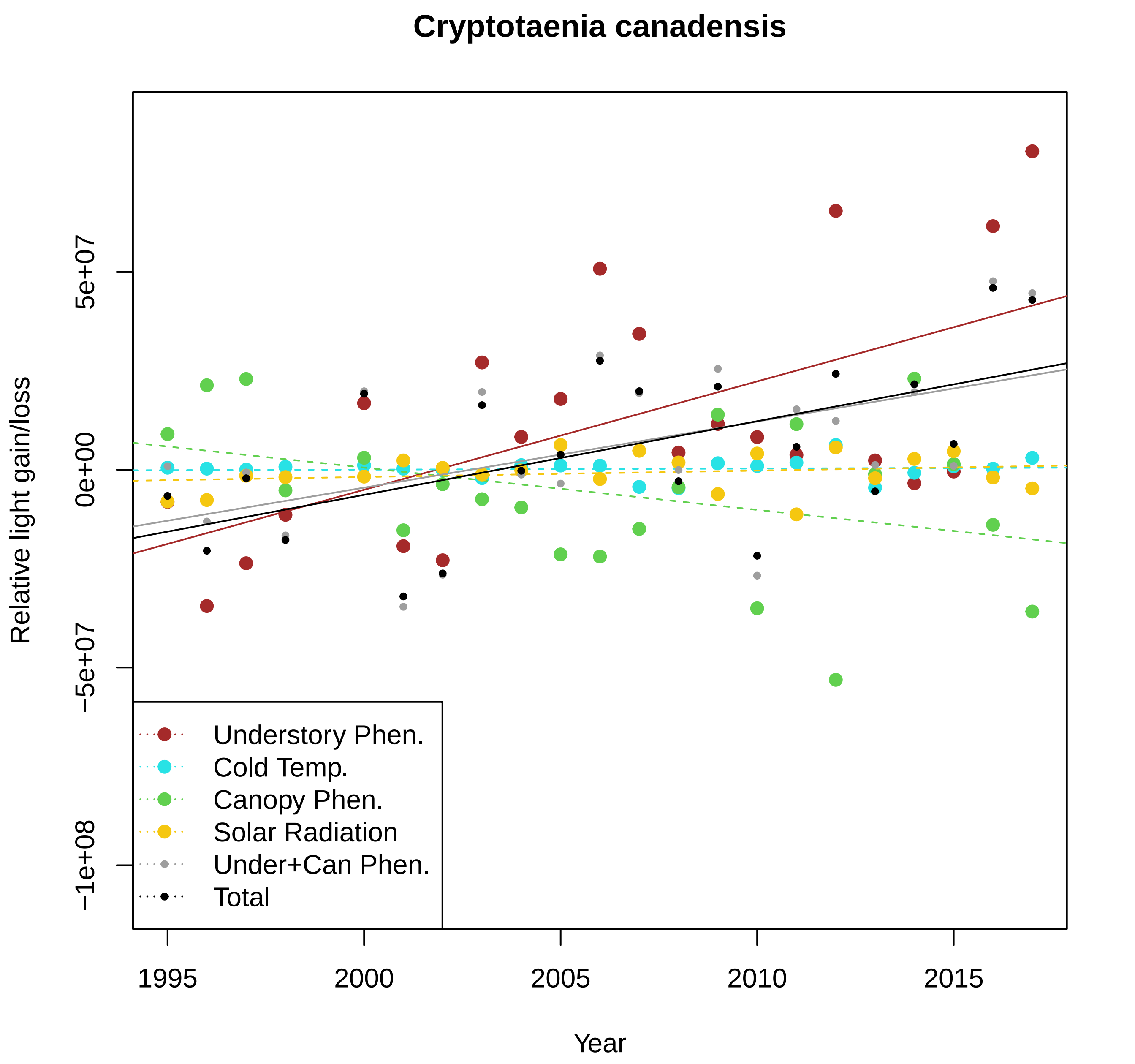

Cryptotaenia canadensis
Relative light gain/loss
5e+07
0e+00
−5e+07
−1e+08
1995
2000
2005
2010
2015
Year
Understory Phen.
Cold Temp.
Canopy Phen.
Solar Radiation
Under+Can Phen.
Total

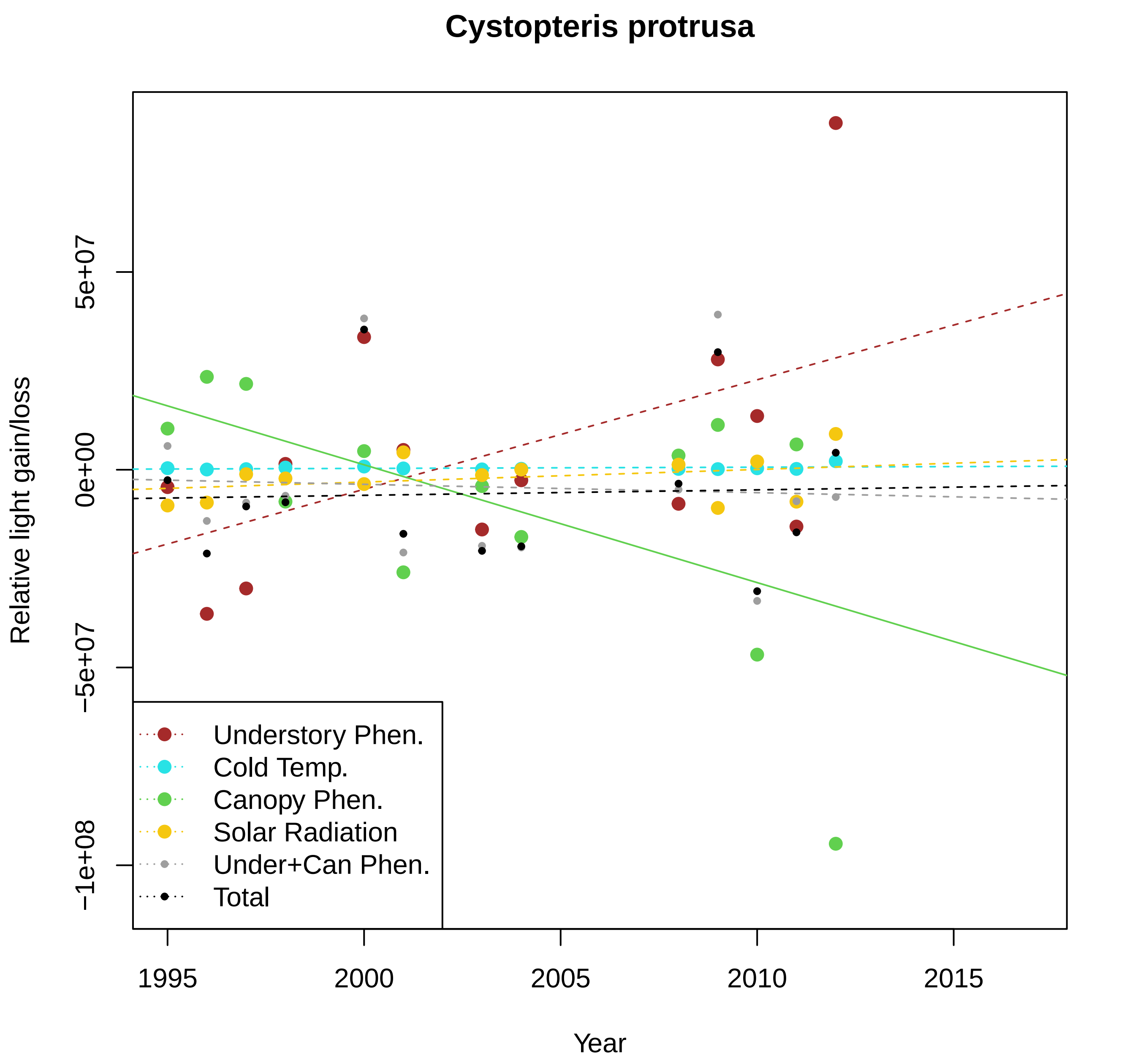
Cystopteris protrusa
Relative light gain/loss
5e+07
0e+00
−5e+07
−1e+08
1995
2000
2005
2010
2015
Year
Understory Phen.
Cold Temp.
Canopy Phen.
Solar Radiation
Under+Can Phen.
Total

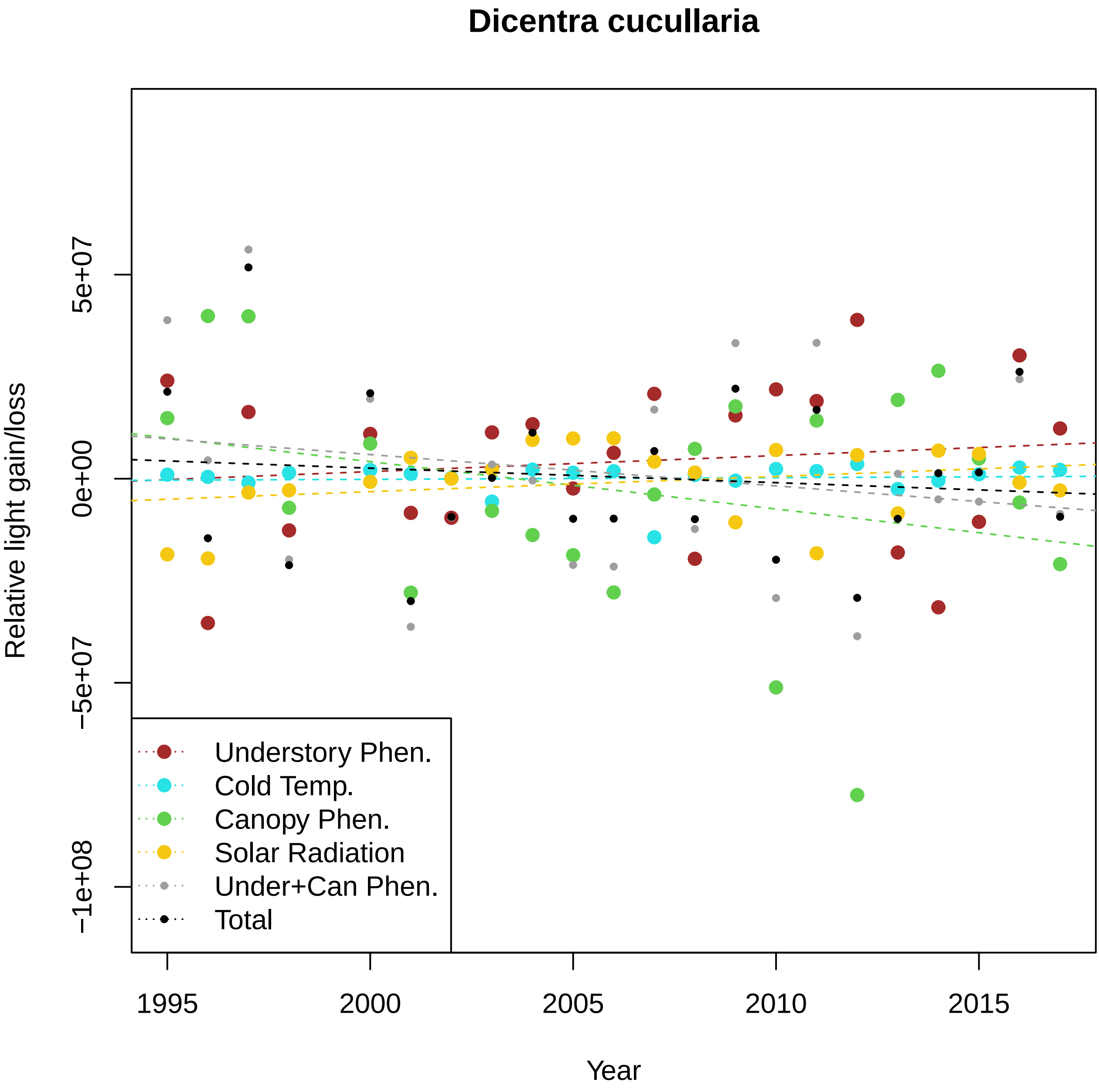

Dicentra cucullaria
Relative light gain/loss
5e+07
0e+00
−5e+07
−1e+08
1995
2000
2005
2010
2015
Year
Understory Phen.
Cold Temp.
Canopy Phen.
Solar Radiation
Under+Can Phen.
Total

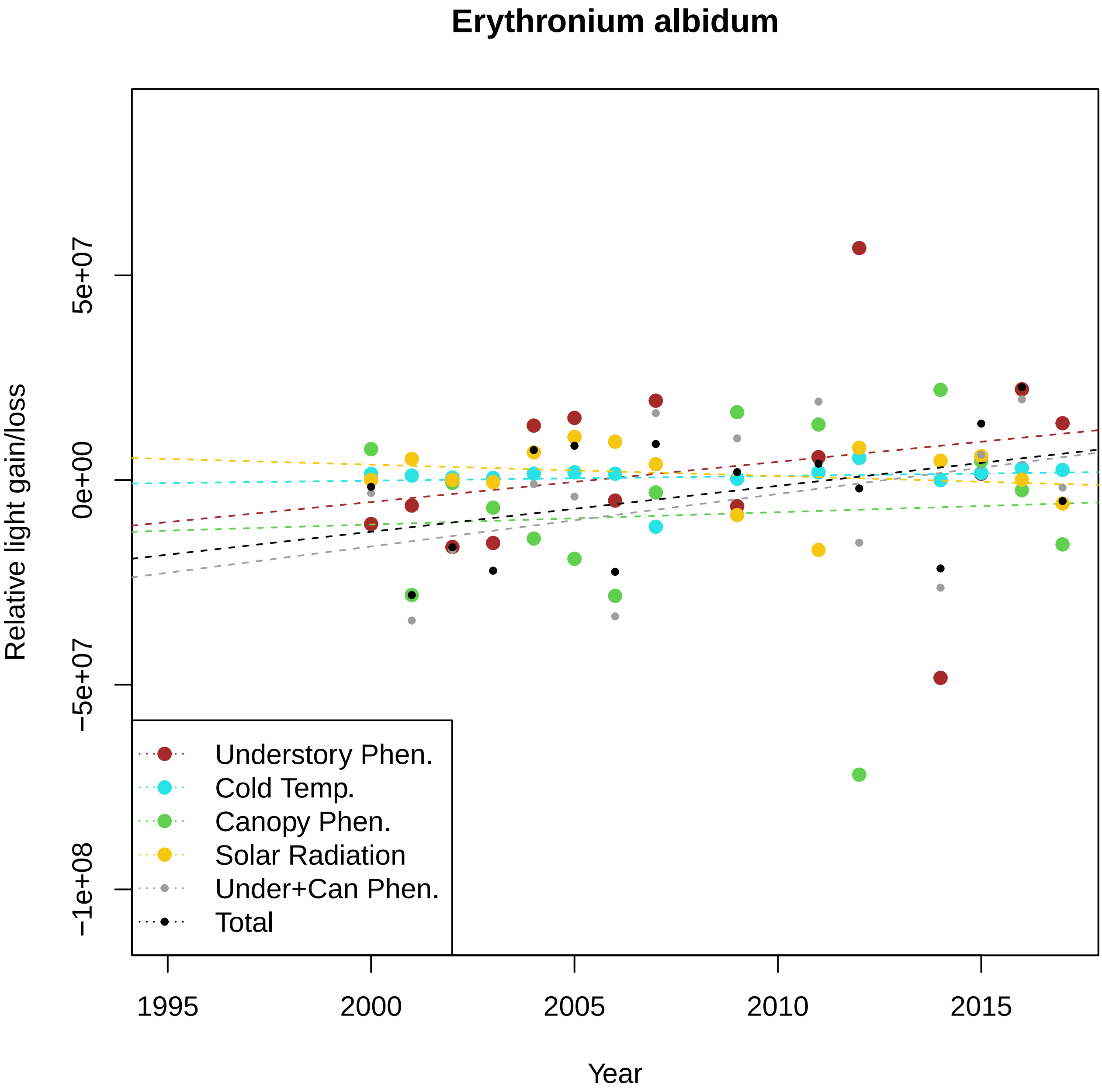
Erythronium albidum
Relative light gain/loss
5e+07
0e+00
−5e+07
−1e+08
1995
2000
2005
2010
2015
Year
Understory Phen.
Cold Temp.
Canopy Phen.
Solar Radiation
Under+Can Phen.
Total

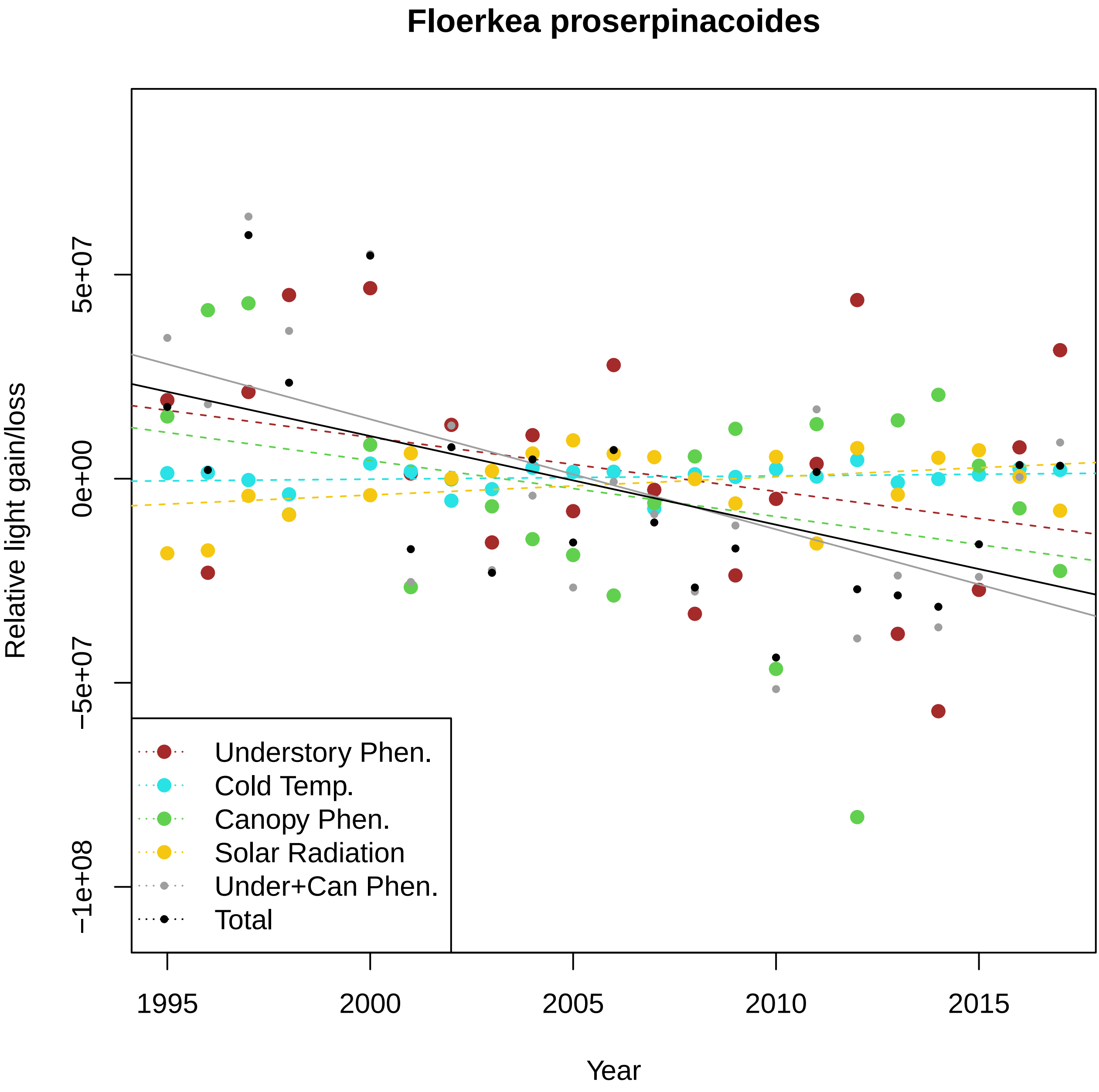
Floerkea proserpinacoides
Relative light gain/loss
5e+07
0e+00
−5e+07
−1e+08
1995
2000
2005
2010
2015
Year
Understory Phen.
Cold Temp.
Canopy Phen.
Solar Radiation
Under+Can Phen.
Total

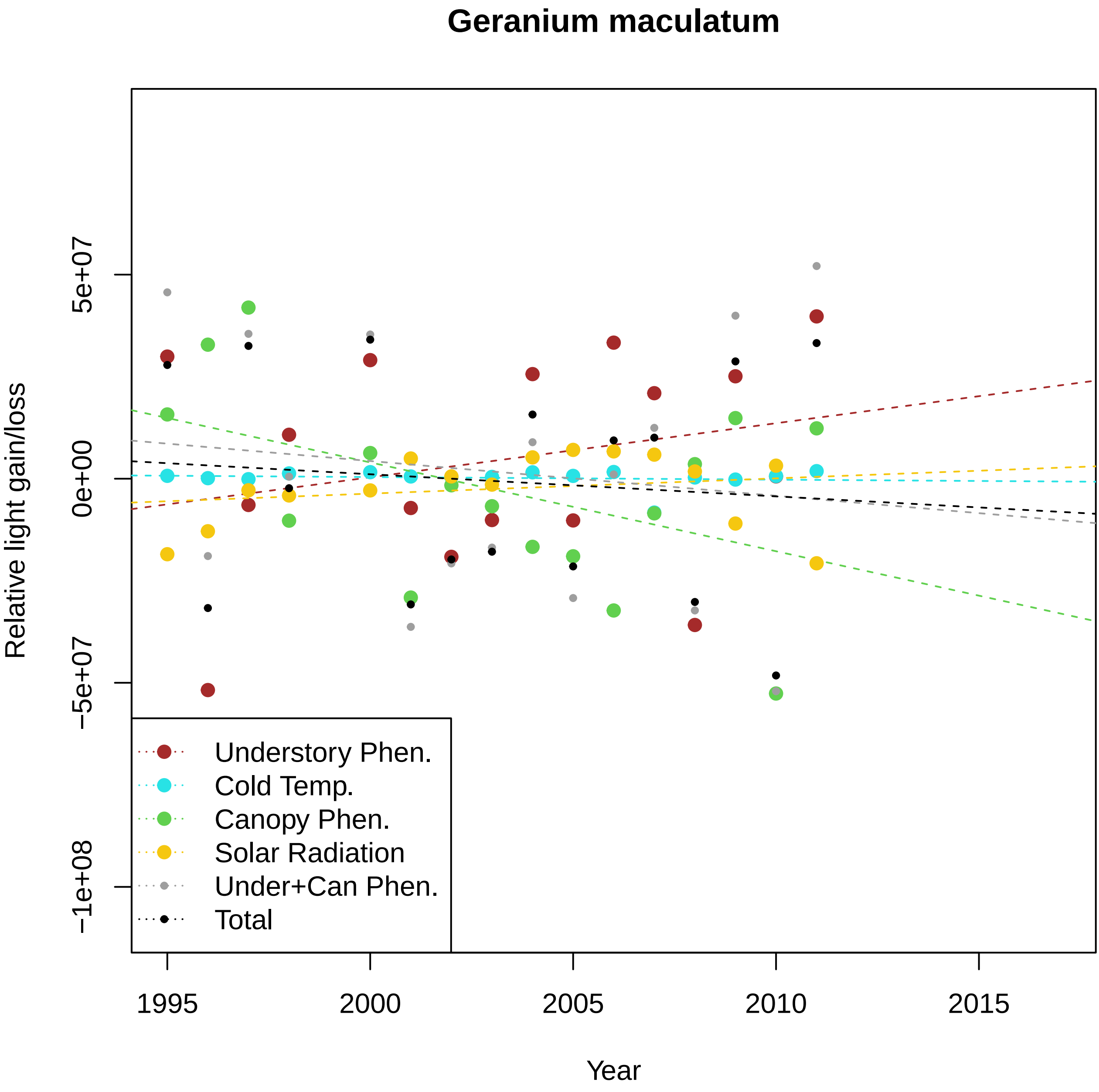

Geranium maculatum
Relative light gain/loss
5e+07
0e+00
−5e+07
−1e+08
1995
2000
2005
2010
2015
Year
Understory Phen.
Cold Temp.
Canopy Phen.
Solar Radiation
Under+Can Phen.
Total

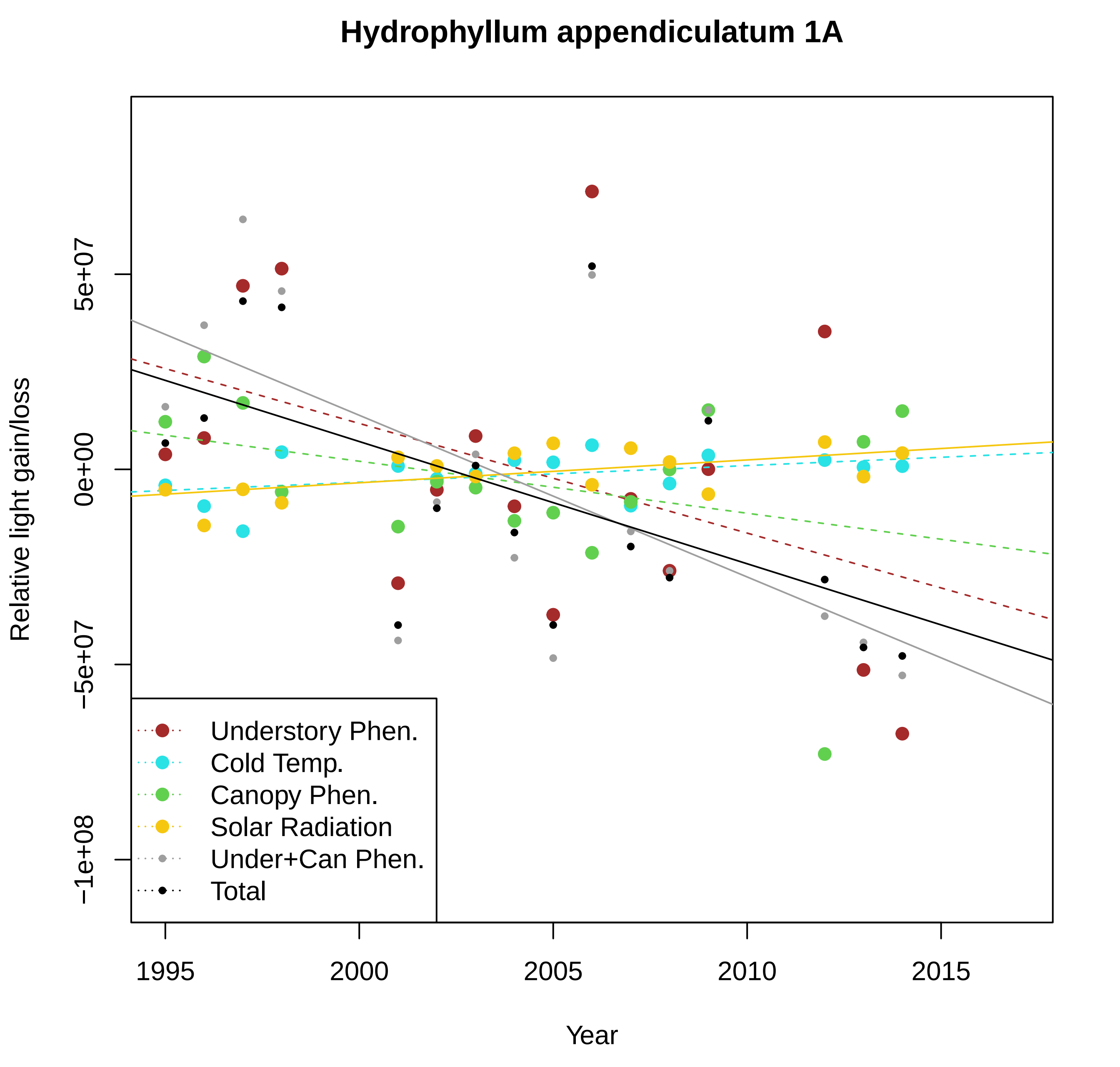

Hydrophyllum appendiculatum 1A
Relative light gain/loss
5e+07
0e+00
−5e+07
−1e+08
1995
2000
2005
2010
2015
Year
Understory Phen.
Cold Temp.
Canopy Phen.
Solar Radiation
Under+Can Phen.
Total

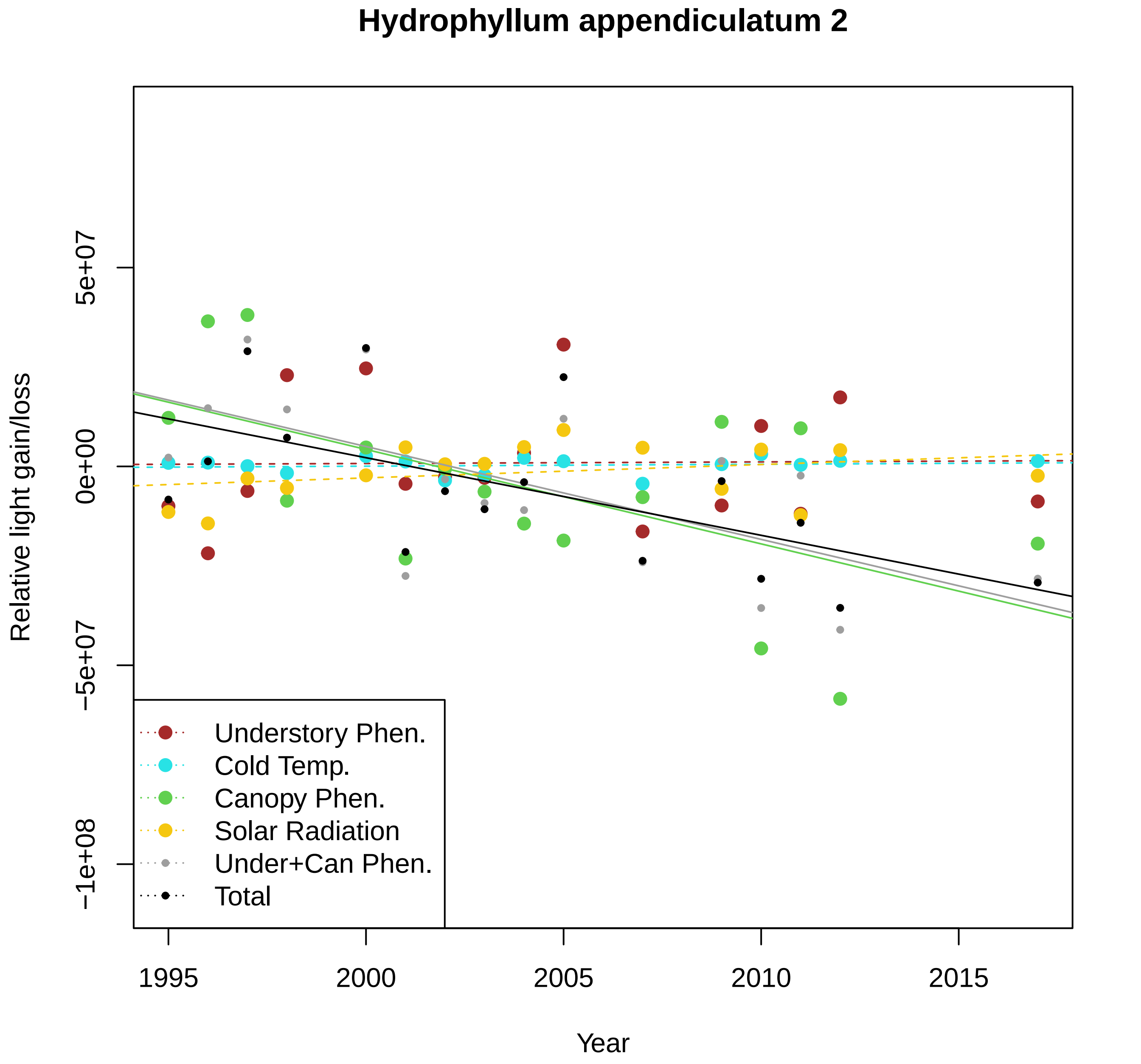

Hydrophyllum appendiculatum 2
Relative light gain/loss
5e+07
0e+00
−5e+07
−1e+08
1995
2000
2005
2010
2015
Year
Understory Phen.
Cold Temp.
Canopy Phen.
Solar Radiation
Under+Can Phen.
Total

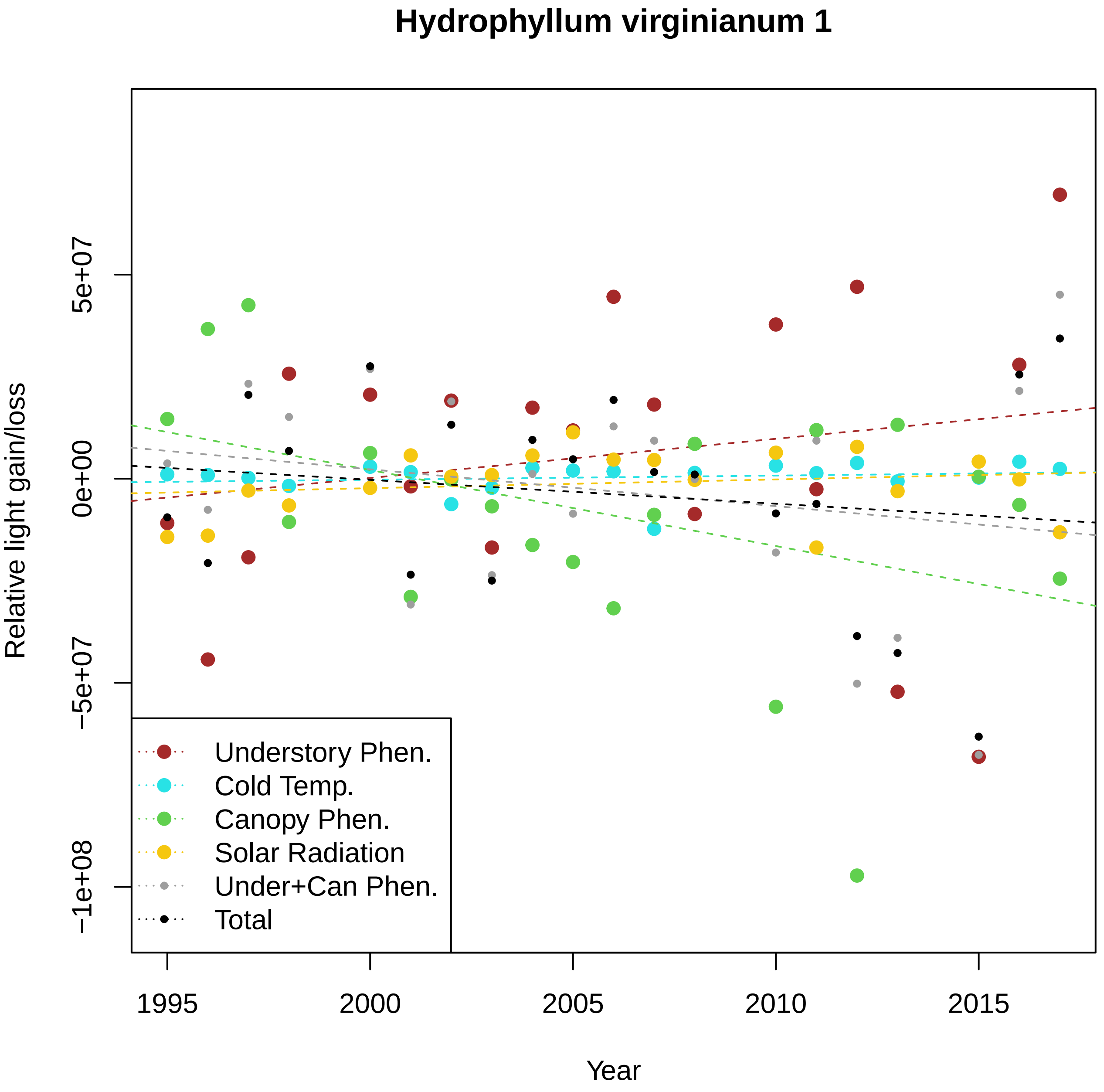

Hydrophyllum virginianum 1
Relative light gain/loss
5e+07
0e+00
−5e+07
−1e+08
1995
2000
2005
2010
2015
Year
Understory Phen.
Cold Temp.
Canopy Phen.
Solar Radiation
Under+Can Phen.
Total

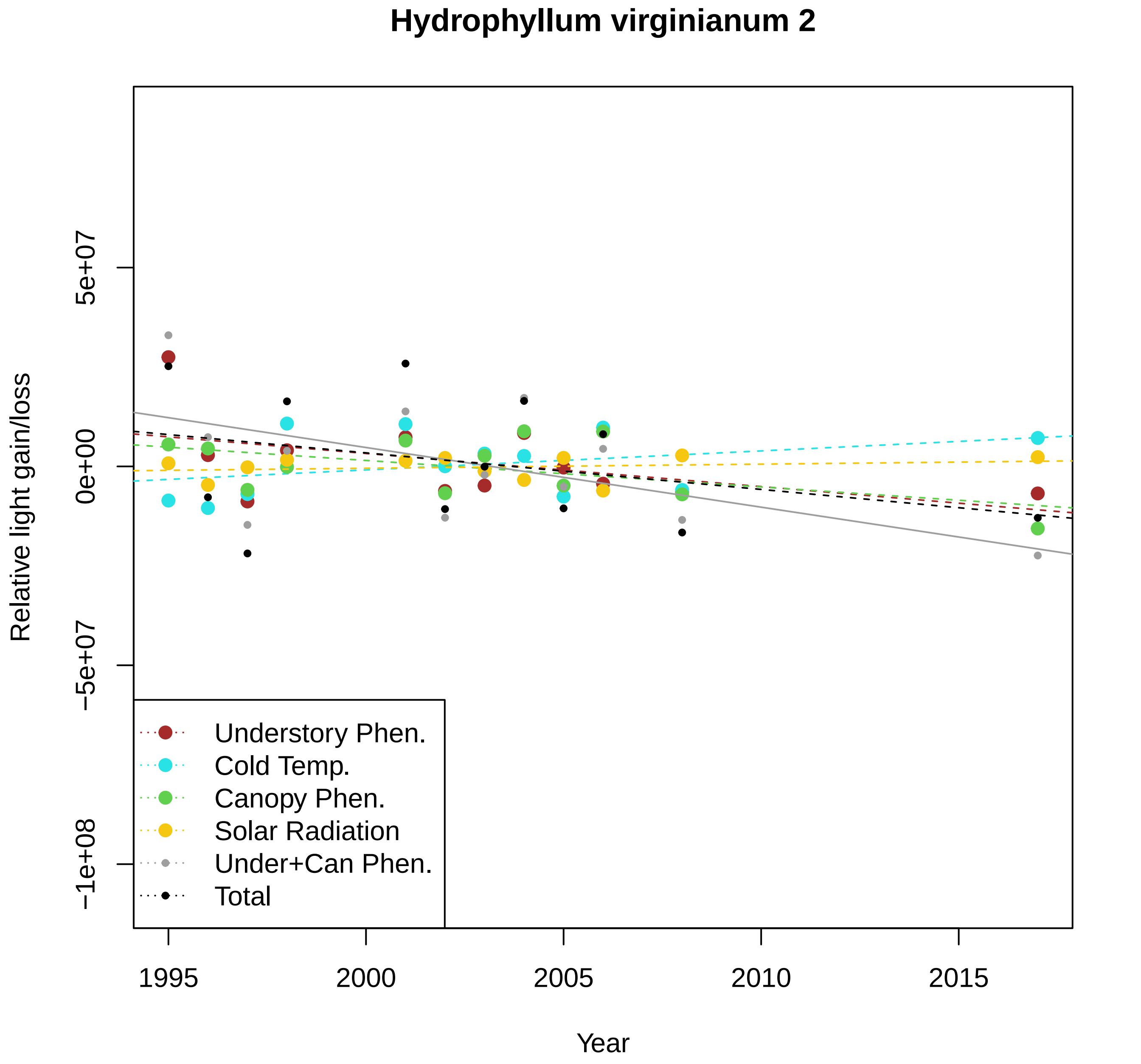

Hydrophyllum virginianum 2
Relative light gain/loss
5e+07
0e+00
−5e+07
−1e+08
Year
1995
2000
2005
2010
2015
Understory Phen.
Cold Temp.
Canopy Phen.
Solar Radiation
Under+Can Phen.
Total

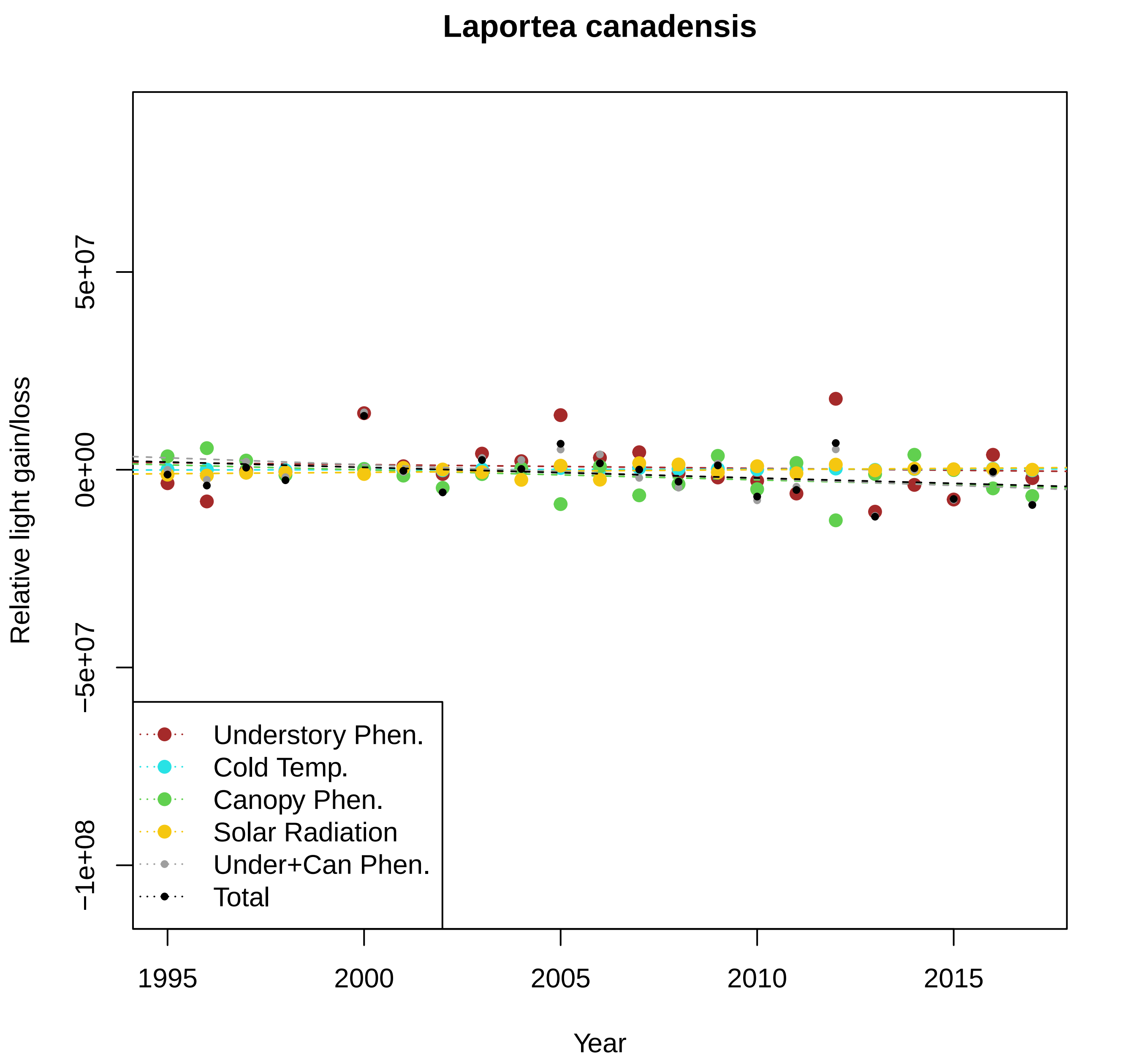
Laportea canadensis
5e+07
0e+00
−5e+07
−1e+08
Relative light gain/loss
1995
2000
2005
2010
2015
Year
Understory Phen.
Cold Temp.
Canopy Phen.
Solar Radiation
Under+Can Phen.
Total

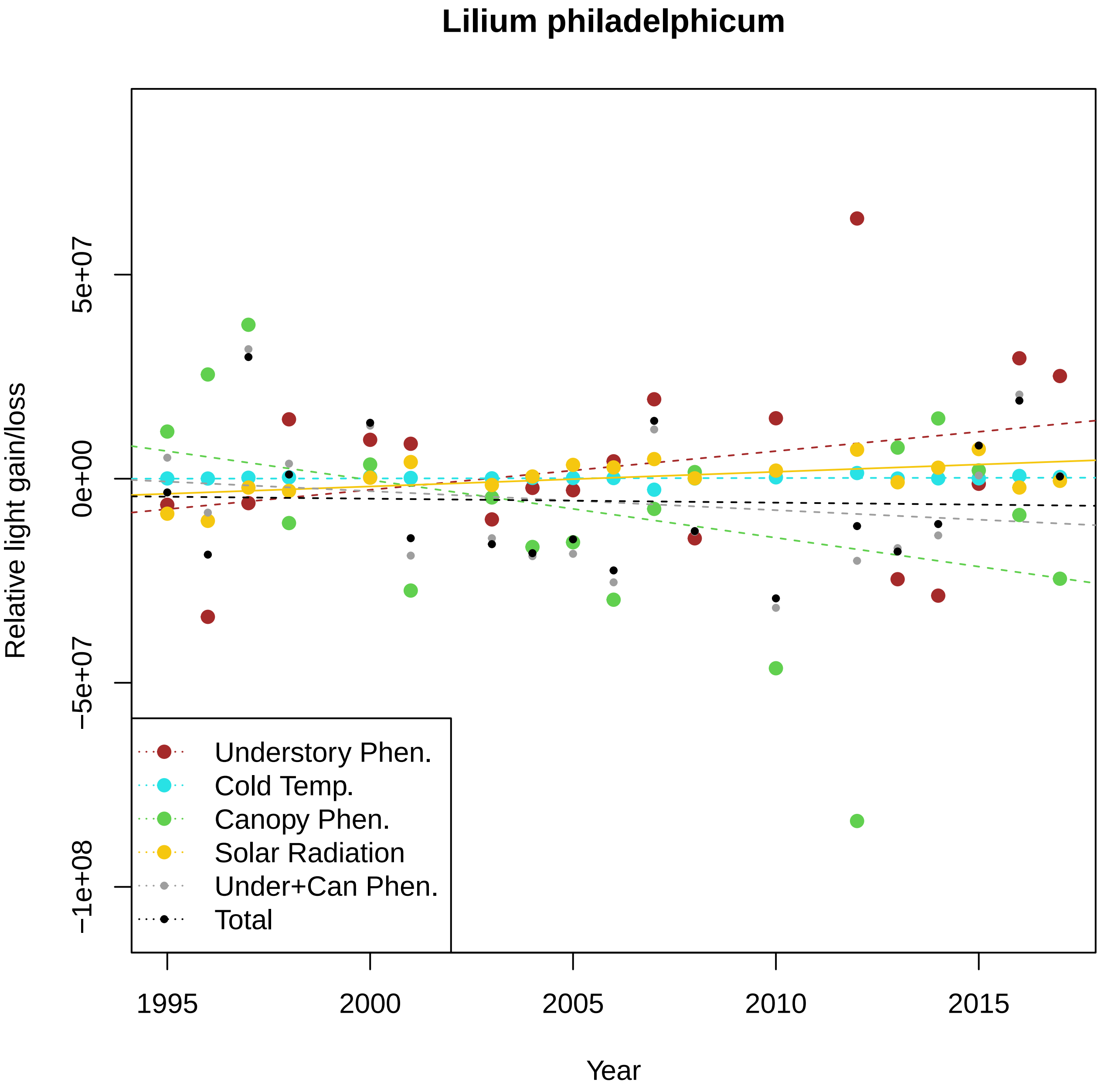

Lilium philadelphicum
Relative light gain/loss
5e+07
0e+00
−5e+07
−1e+08
1995
2000
2005
2010
2015
Year
Understory Phen.
Cold Temp.
Canopy Phen.
Solar Radiation
Under+Can Phen.
Total

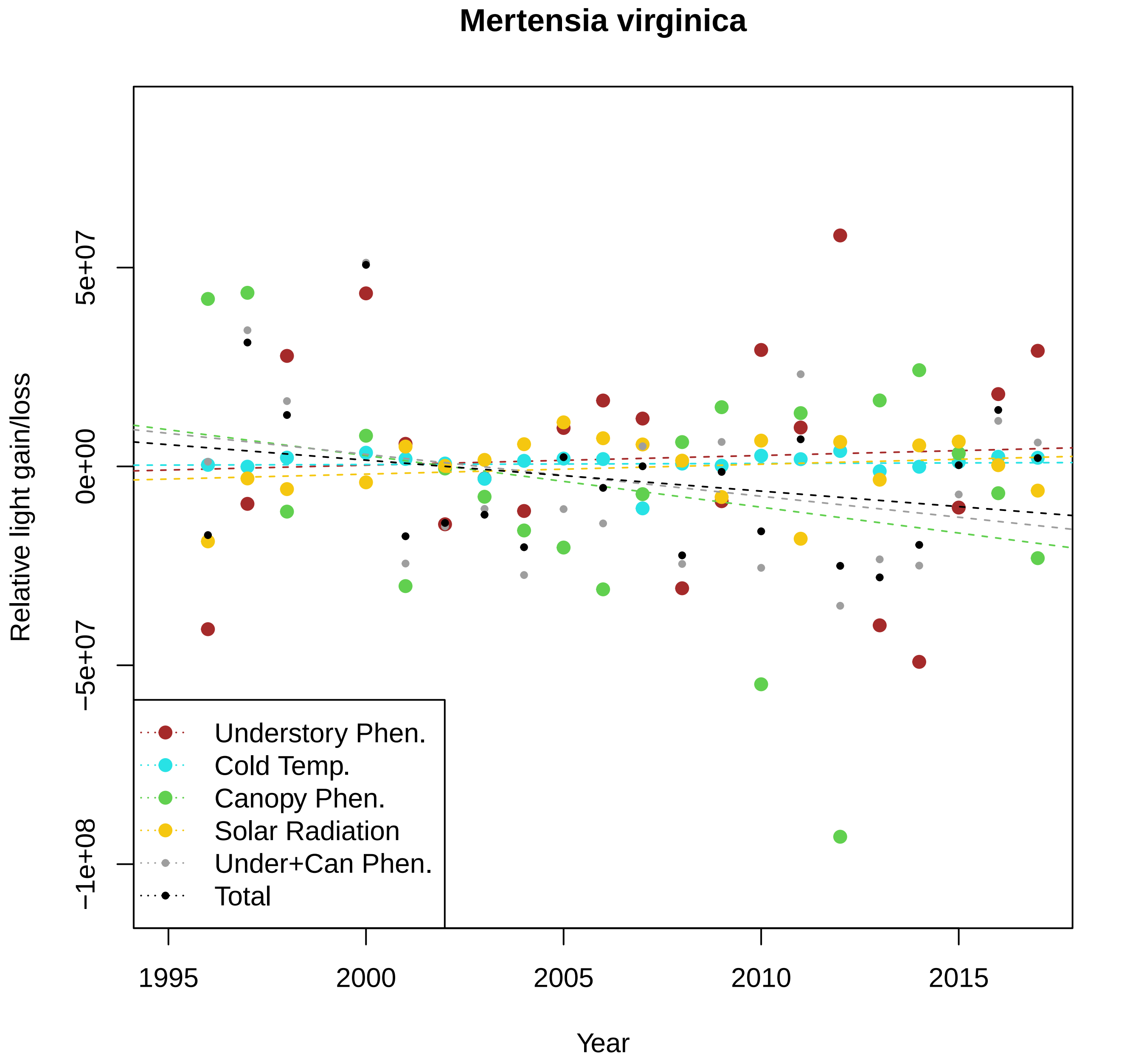

Mertensia virginica
Relative light gain/loss
5e+07
0e+00
−5e+07
−1e+08
1995
2000
2005
2010
2015
Year
Understory Phen.
Cold Temp.
Canopy Phen.
Solar Radiation
Under+Can Phen.
Total

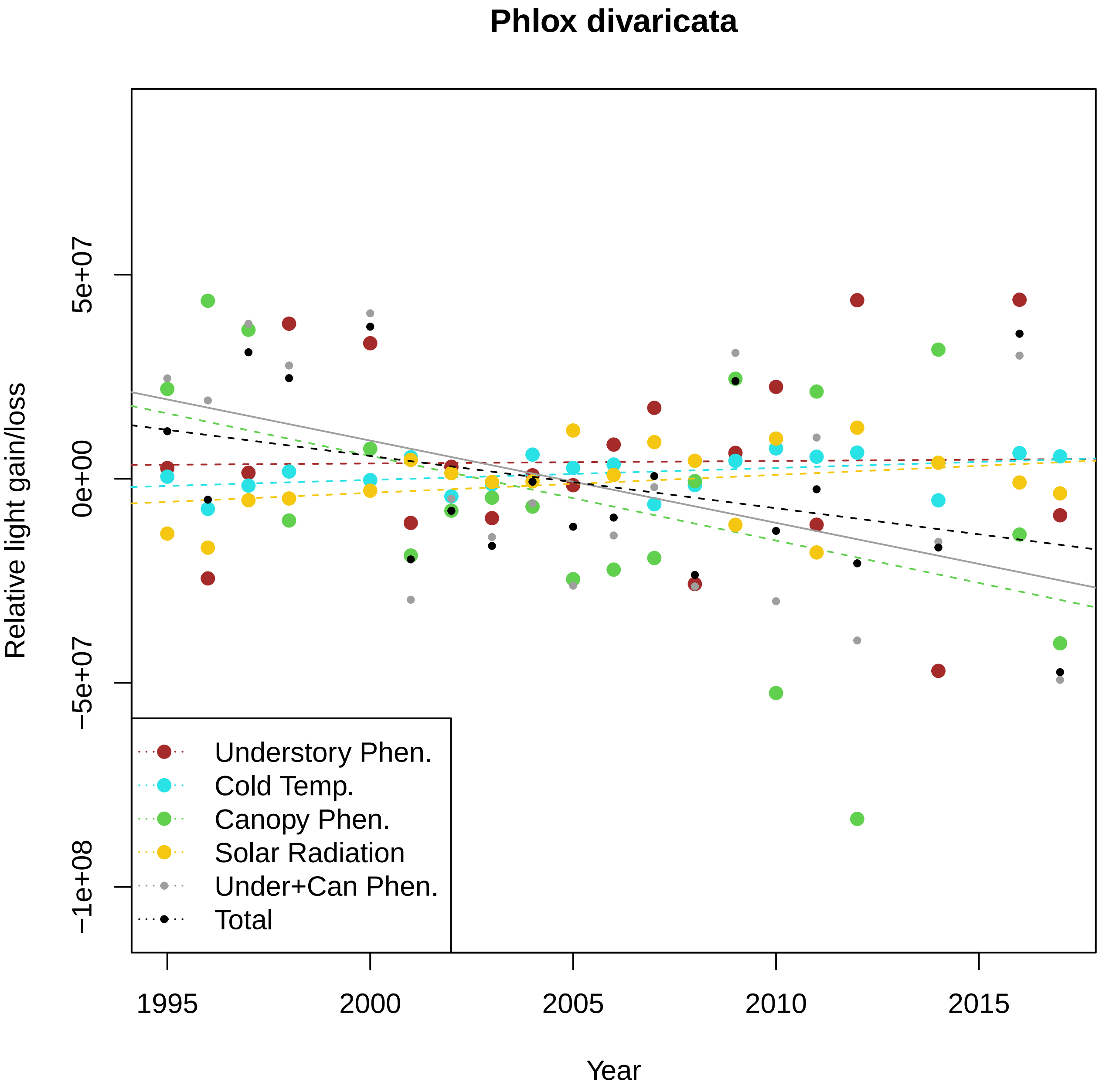

Phlox divaricata
Relative light gain/loss
5e+07
0e+00
−5e+07
−1e+08
1995
2000
2005
2010
2015
Year
Understory Phen.
Cold Temp.
Canopy Phen.
Solar Radiation
Under+Can Phen.
Total

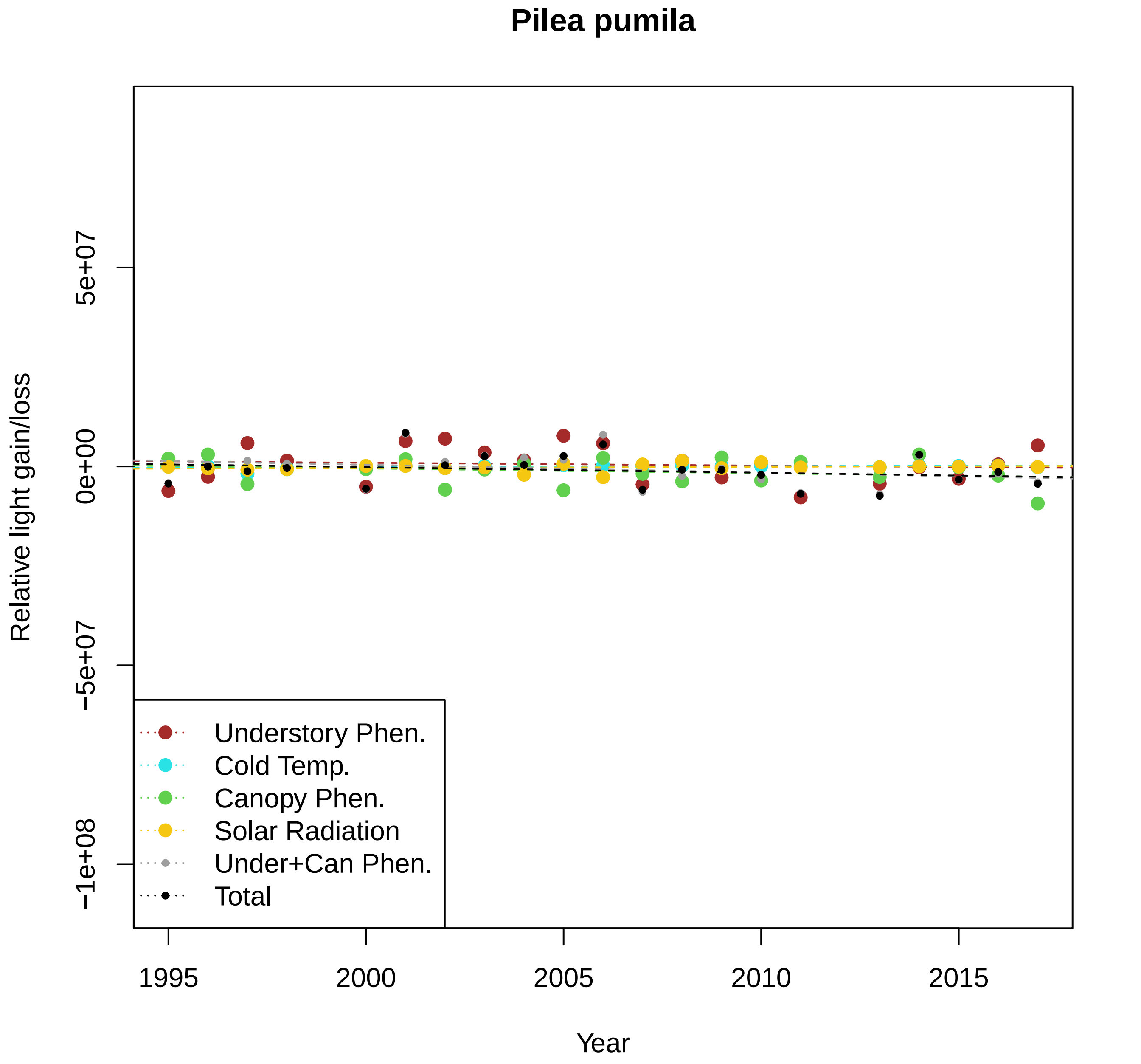

Pilea pumila
Relative light gain/loss
5e+07
0e+00
−5e+07
−1e+08
1995
2000
2005
2010
2015
Year
Understory Phen.
Cold Temp.
Canopy Phen.
Solar Radiation
Under+Can Phen.
Total

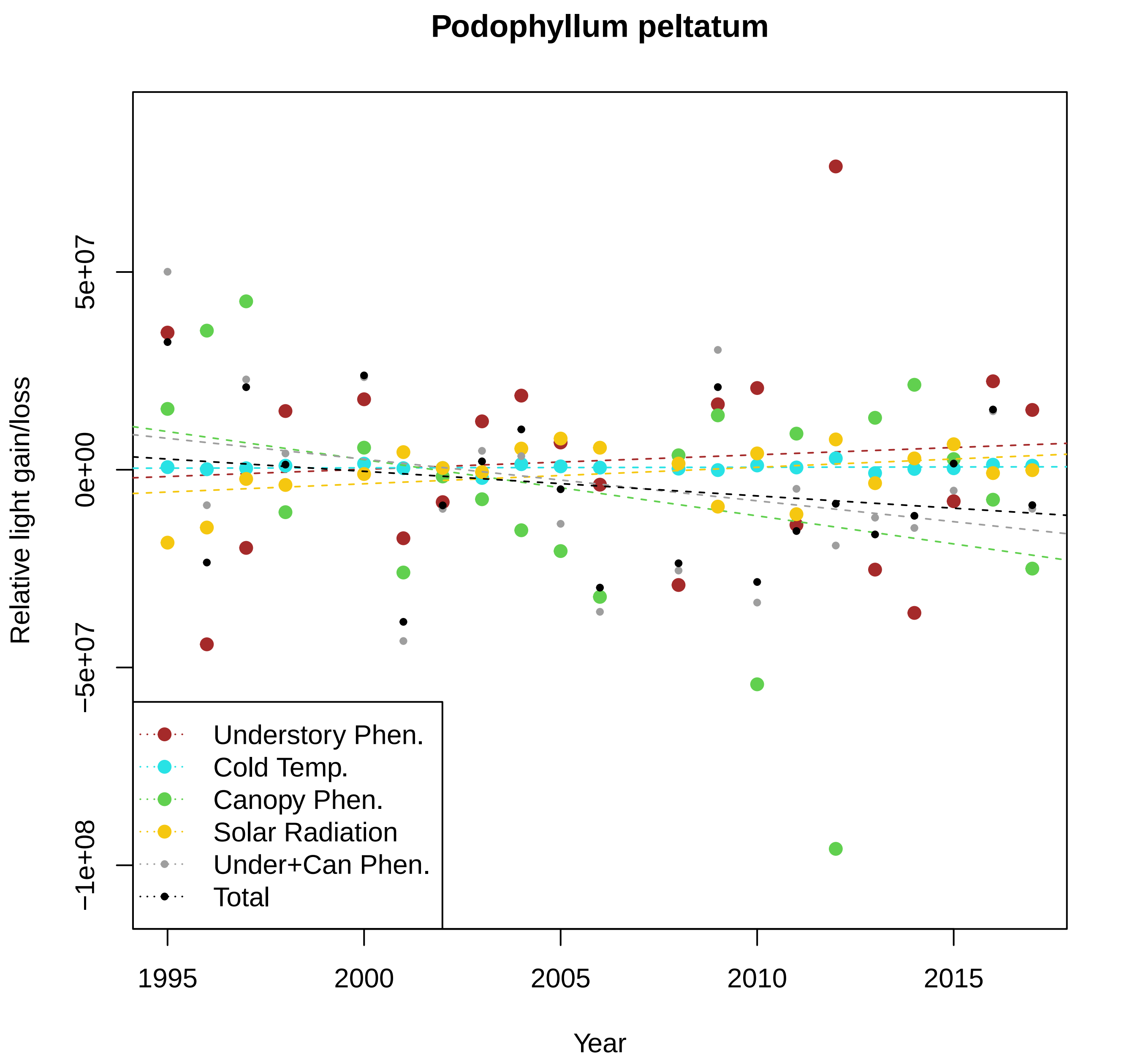

Podophyllum peltatum
Relative light gain/loss
5e+07
0e+00
−5e+07
−1e+08
1995
2000
2005
2010
2015
Year
Understory Phen.
Cold Temp.
Canopy Phen.
Solar Radiation
Under+Can Phen.
Total

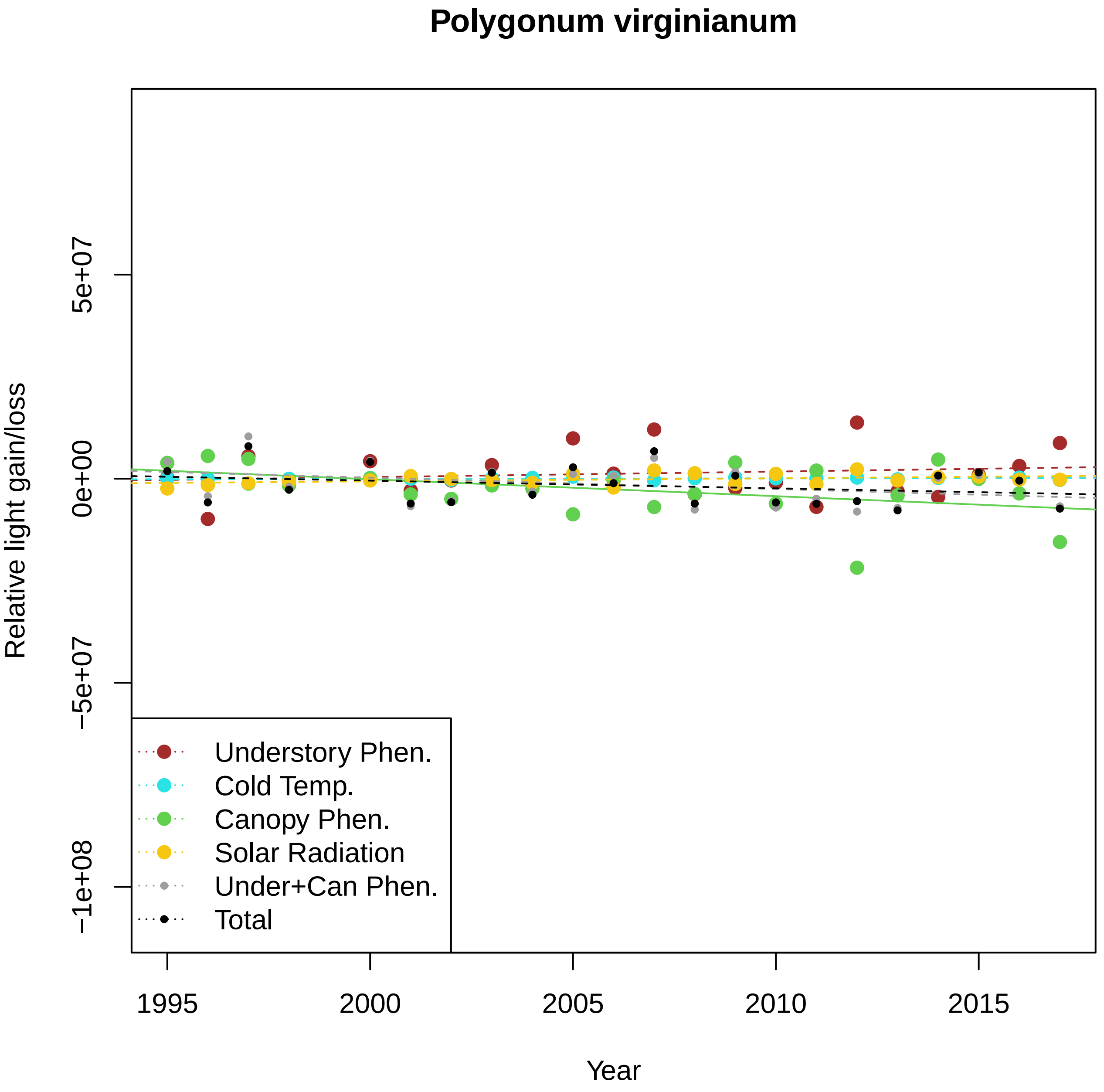

Polygonum virginianum
Relative light gain/loss
5e+07
0e+00
−5e+07
−1e+08
1995
2000
2005
2010
2015
Year
Understory Phen.
Cold Temp.
Canopy Phen.
Solar Radiation
Under+Can Phen.
Total

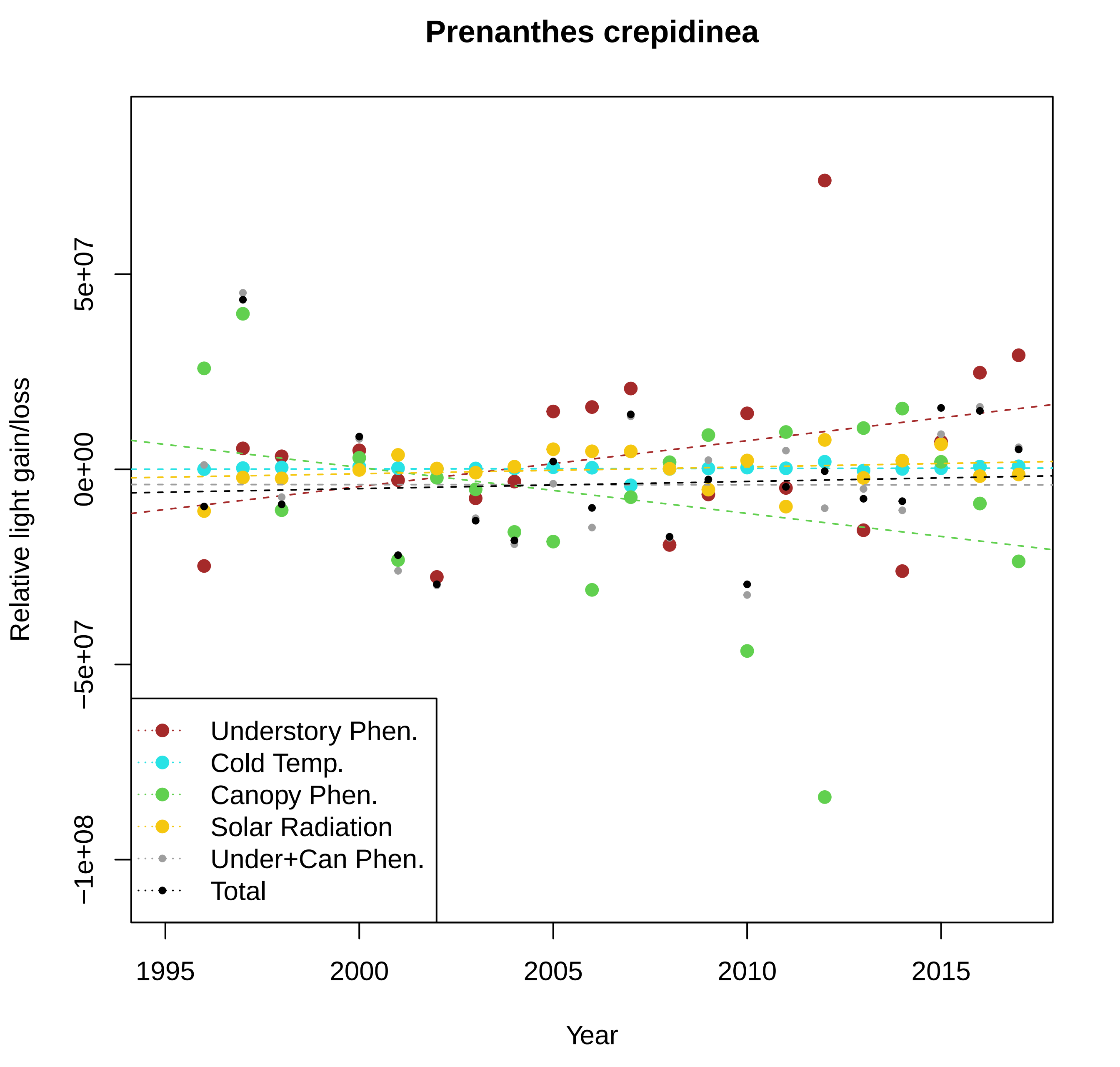
Prenanthes crepidinea
Relative light gain/loss
5e+07
0e+00
−5e+07
−1e+08
1995
2000
2005
2010
2015
Year
Understory Phen.
Cold Temp.
Canopy Phen.
Solar Radiation
Under+Can Phen.
Total

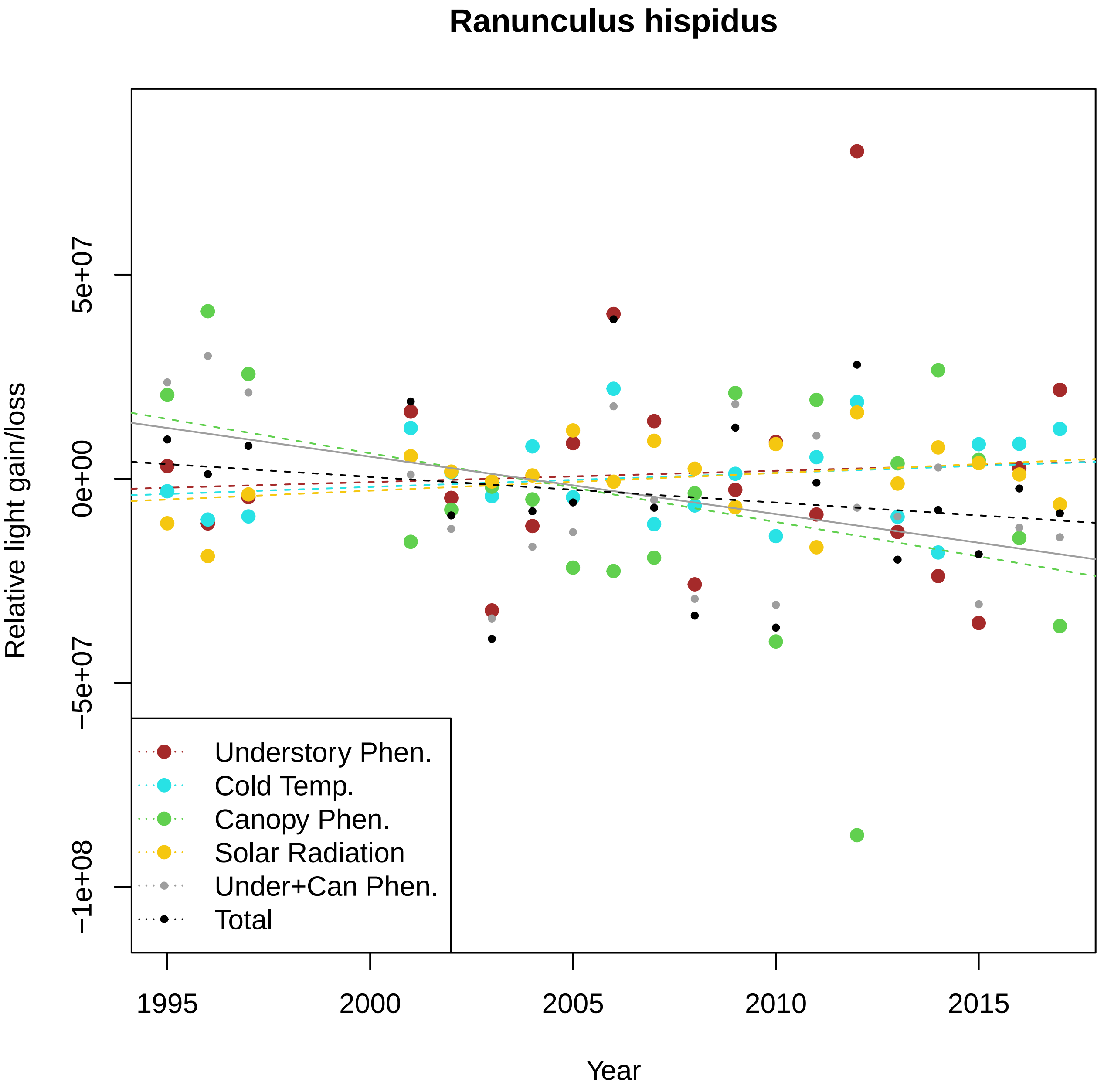
Ranunculus hispidus
Relative light gain/loss
5e+07
0e+00
−5e+07
−1e+08
1995
2000
2005
2010
2015
Year
Understory Phen.
Cold Temp.
Canopy Phen.
Solar Radiation
Under+Can Phen.
Total

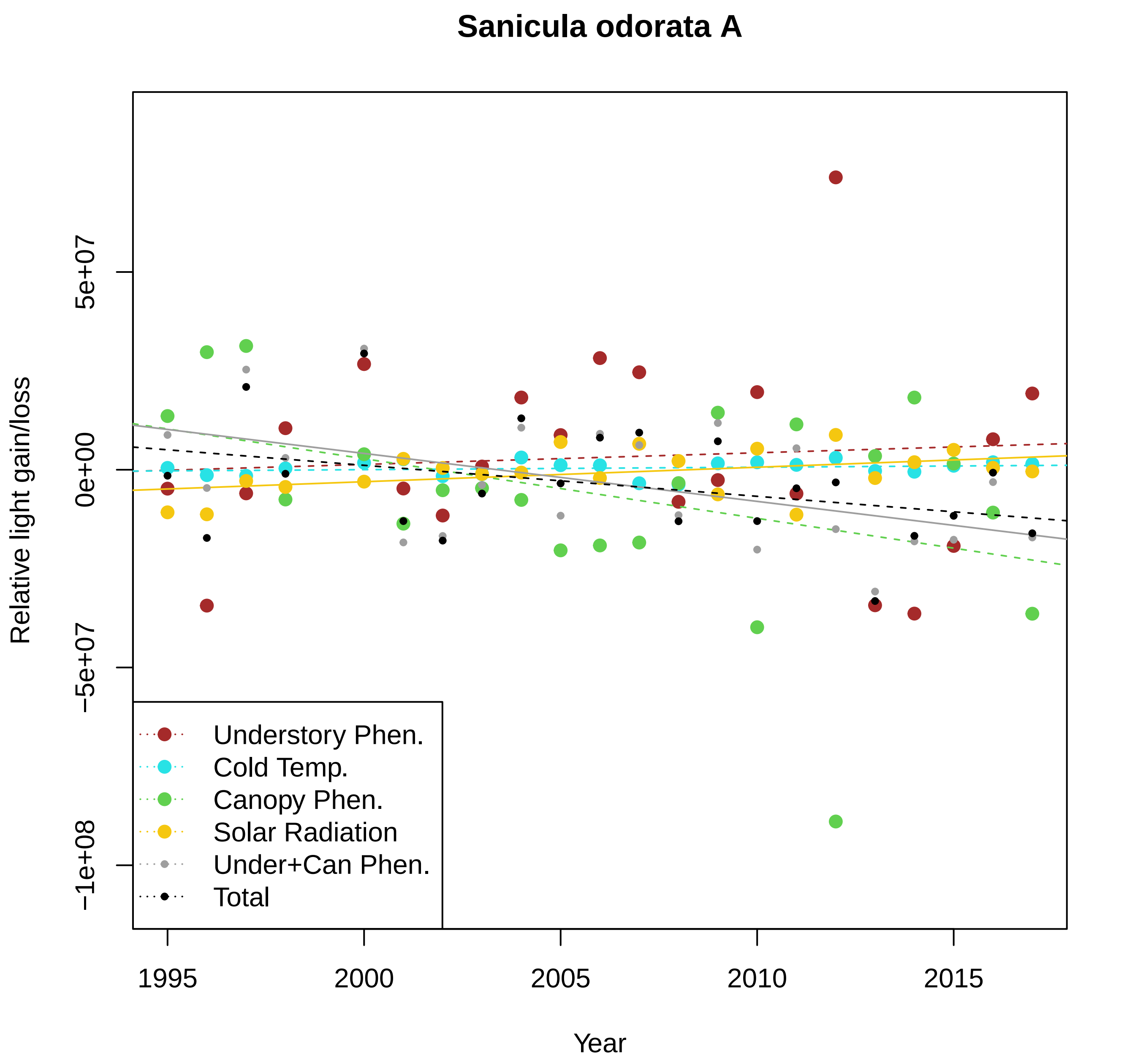

Sanicula odorata A
Relative light gain/loss
5e+07
0e+00
−5e+07
−1e+08
1995
2000
2005
2010
2015
Year
Understory Phen.
Cold Temp.
Canopy Phen.
Solar Radiation
Under+Can Phen.
Total

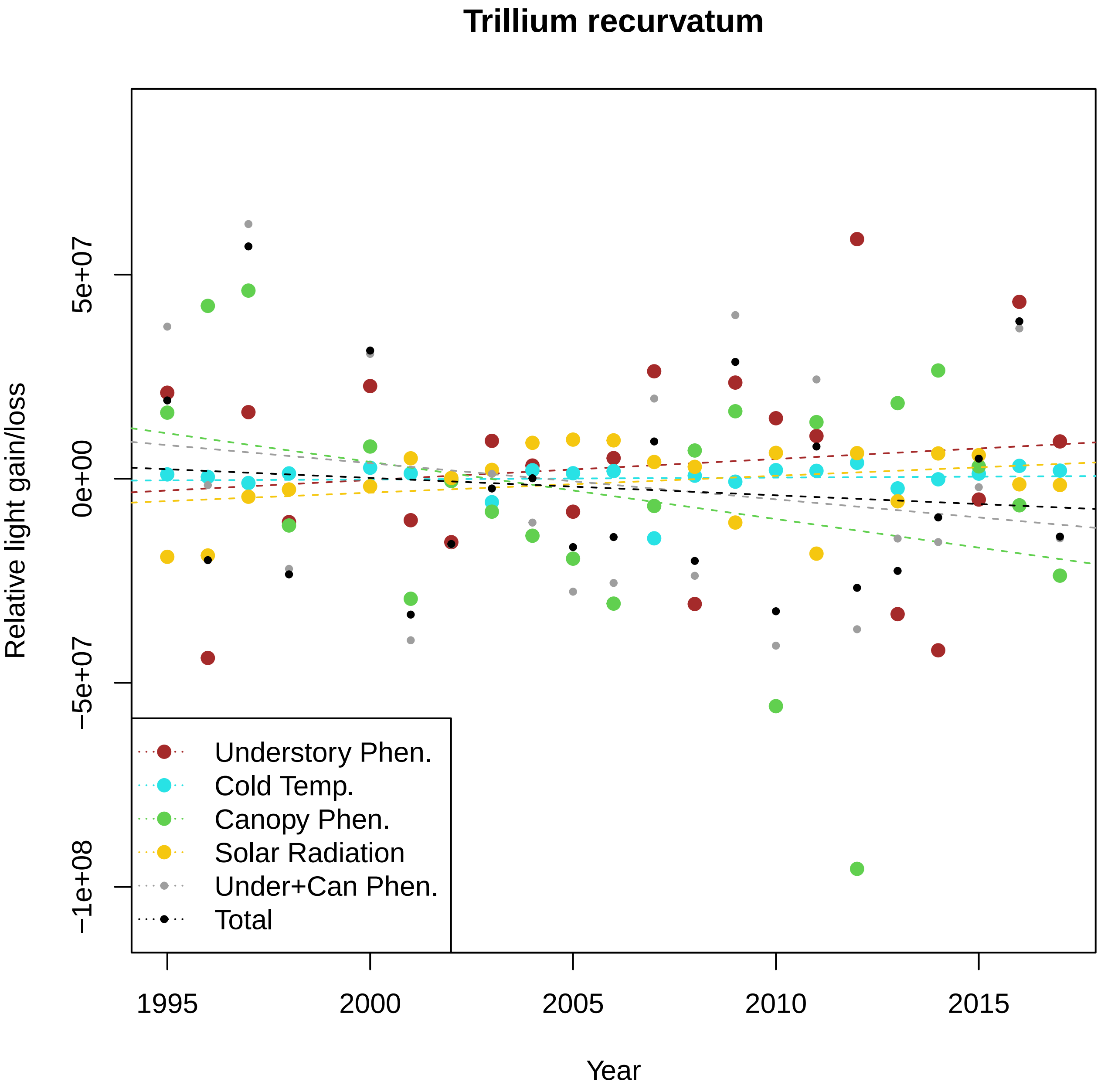

Trillium recurvatum
Relative light gain/loss
5e+07
0e+00
−5e+07
−1e+08
1995
2000
2005
2010
2015
Year
Understory Phen.
Cold Temp.
Canopy Phen.
Solar Radiation
Under+Can Phen.
Total

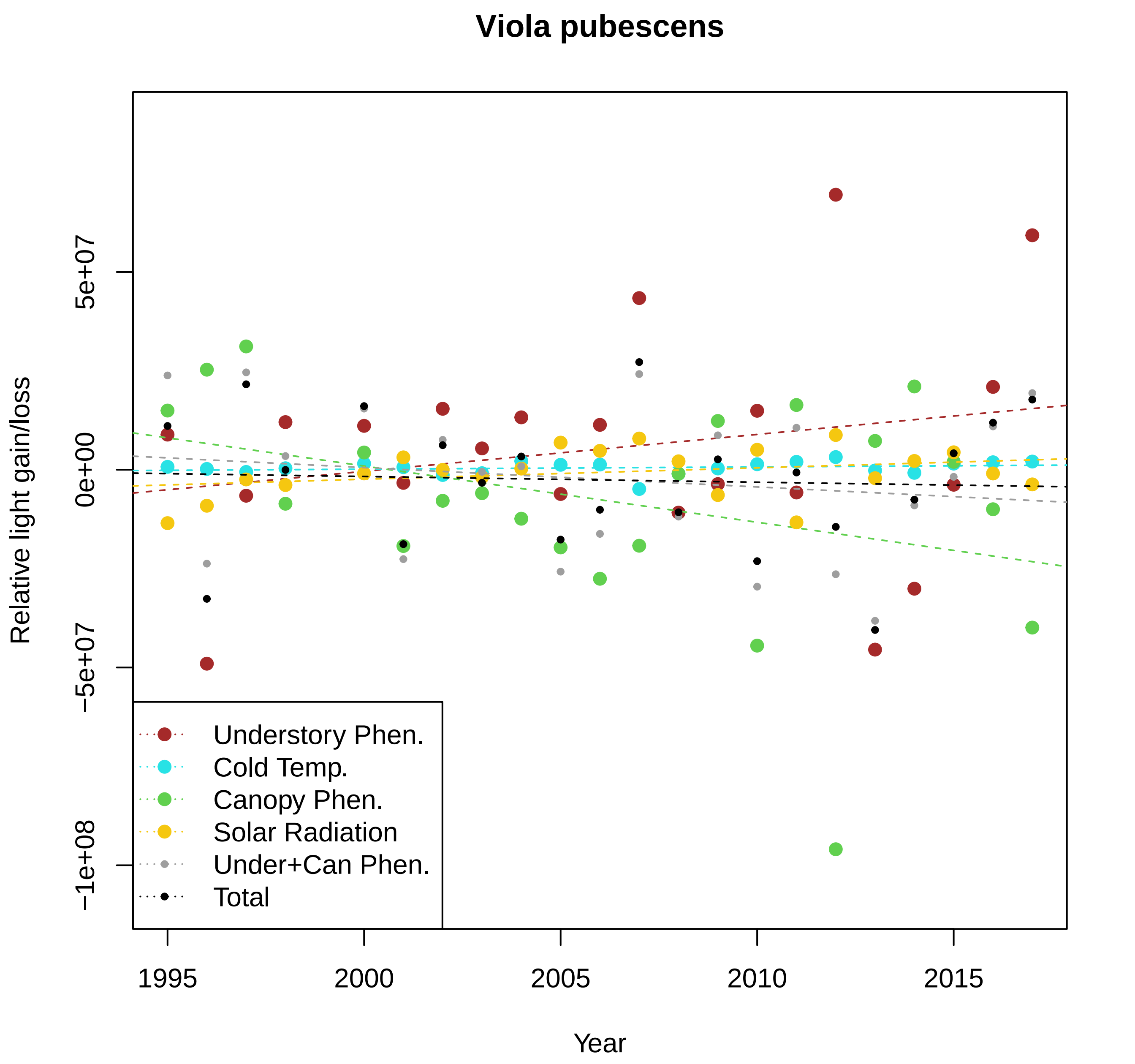

Viola pubescens
Relative light gain/loss
5e+07
0e+00
−5e+07
−1e+08
1995
2000
2005
2010
2015
Year
Understory Phen.
Cold Temp.
Canopy Phen.
Solar Radiation
Under+Can Phen.
Total

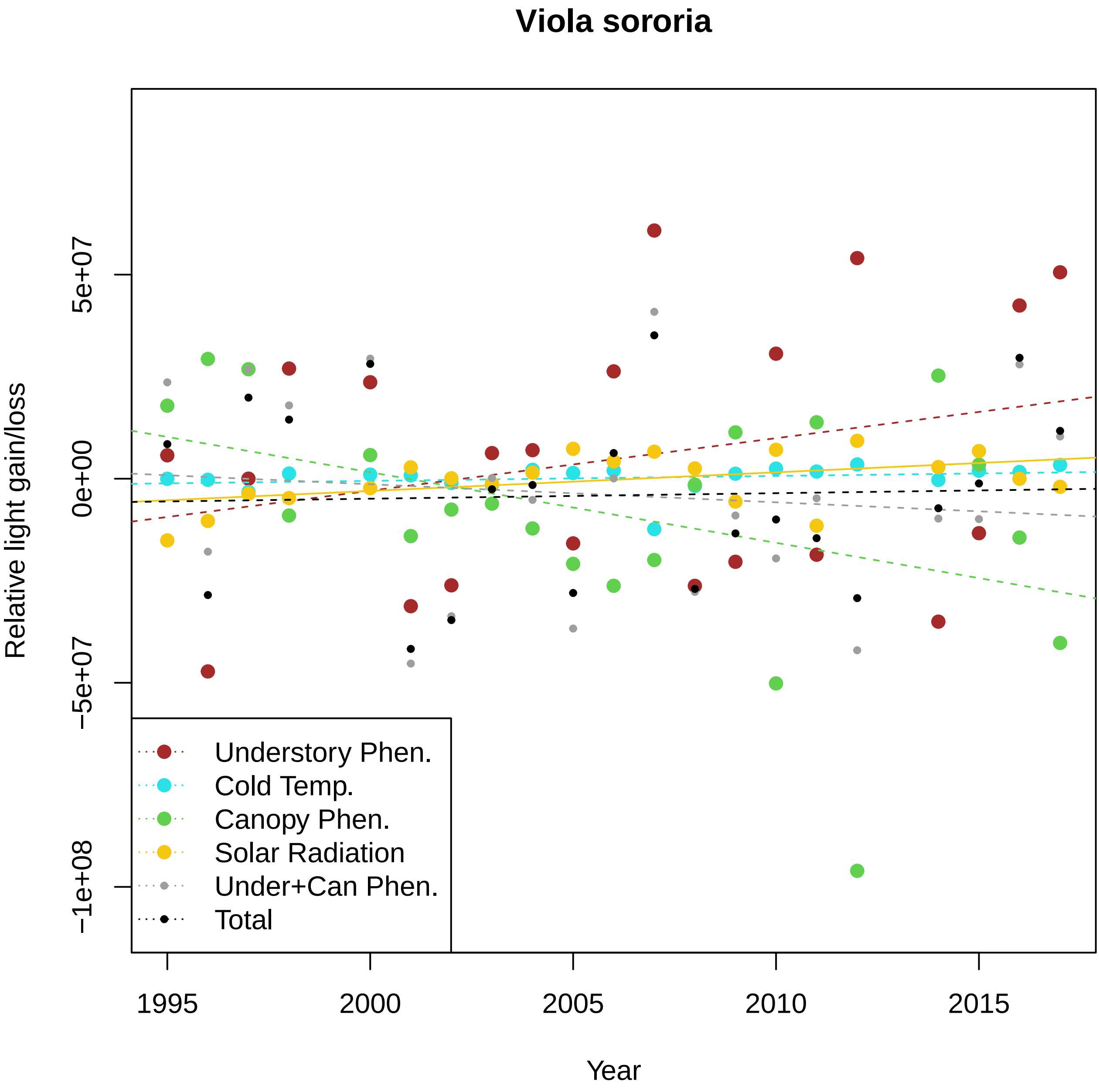

Viola sororia
Relative light gain/loss
5e+07
0e+00
−5e+07
−1e+08
1995
2000
2005
2010
2015
Year
Understory Phen.
Cold Temp.
Canopy Phen.
Solar Radiation
Under+Can Phen.
Total
