# Supplementary material for: Understory plants evade shading in a temperate deciduous forest amid climate variability by shifting phenology in synchrony with canopy trees
Source: PLoS One. 2024 Jun 26;19(6):e0306023. doi: 10.1371/journal.pone.0306023 (PMC11207122; doi:10.1371/journal.pone.0306023)
Supplement: S5 Fig — This integrative measure of spring temperature means that warmer springs fall to the left on the x-axis. Solid lines indicate a statistically-significant (p < .05) difference of the estimated slope from 0, while dashed lines indicate that this standard was not met. (PDF) [file pone.0306023.s011.pdf]

Supporting Information 11 for Augspurger CK, Salk CF. Understory plants reduce light loss in a temperate deciduous forest amid climate variability by shifting phenology in synchrony with canopy trees. PLoS One. In review.

Supporting Information 11:
Trends in sapling species' light interception as a function of date on which the 48−day running average temperature first exceeded 13° C (see Methods: Section 6). This integrative measure of spring temperature means that warmer springs fall to the left on the x−axis. Solid lines indicate a statistically−significant ($p<.05$) difference of the estimated slope from 0, while dashed lines indicate that this standard was not met.

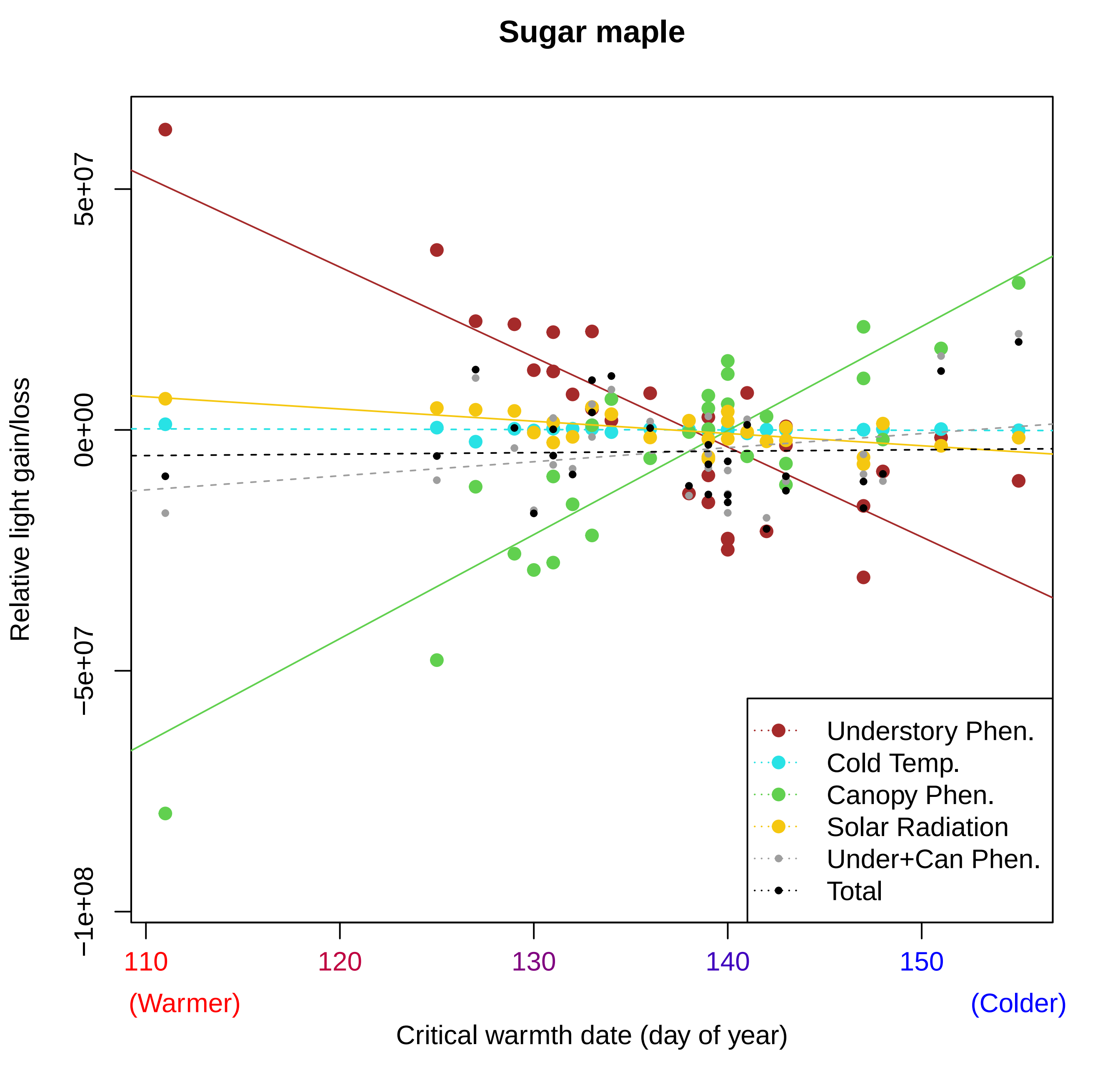
Sugar maple
Relative light gain/loss
5e+07
0e+00
−5e+07
−1e+08
110
120
130
140
150
(Warmer)
(Colder)
Critical warmth date (day of year)
Understory Phen.
Cold Temp.
Canopy Phen.
Solar Radiation
Under+Can Phen.
Total

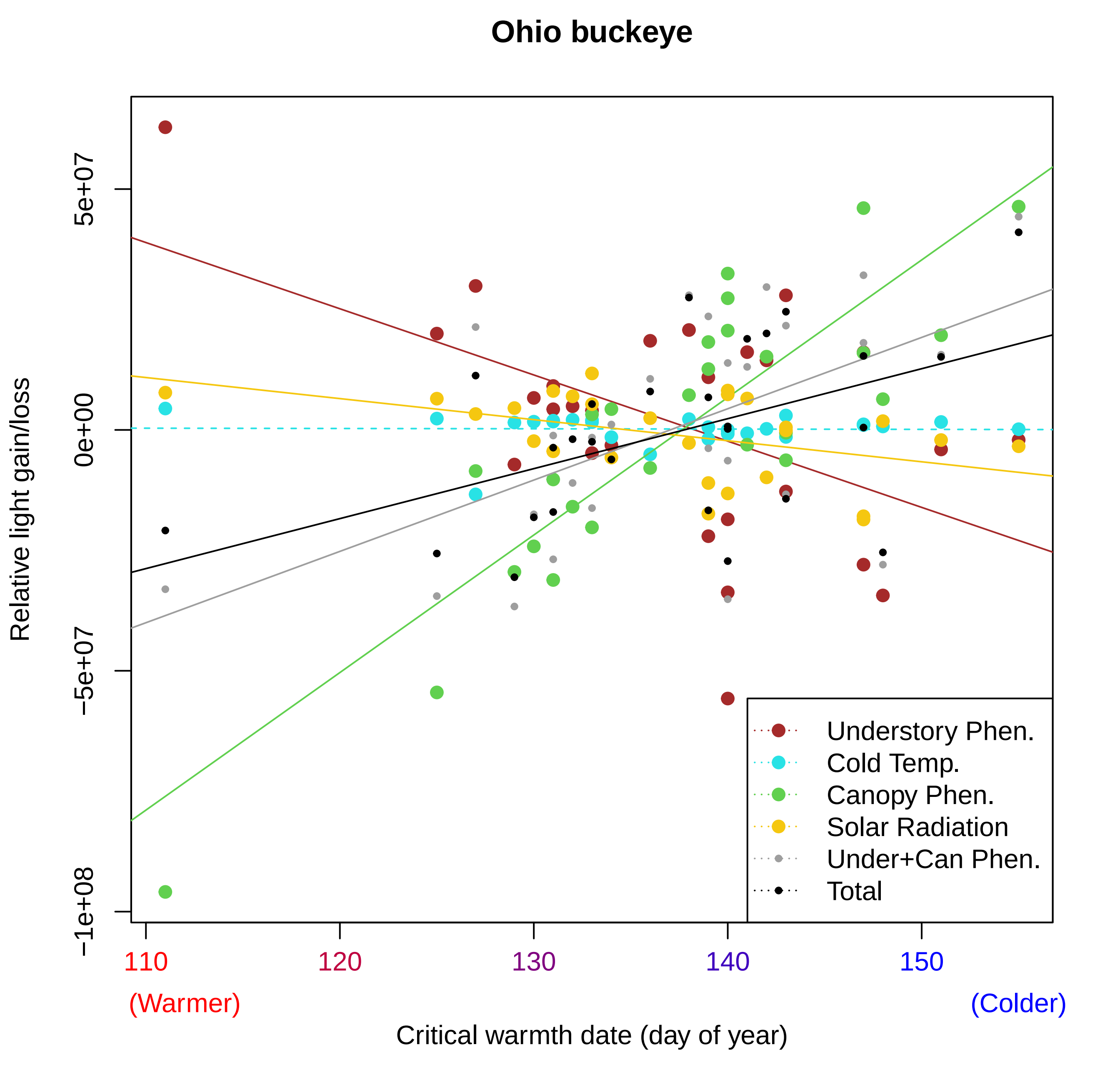

Ohio buckeye
Relative light gain/loss
5e+07
0e+00
−5e+07
−1e+08
110
120
130
140
150
(Warmer)
(Colder)
Critical warmth date (day of year)
Understory Phen.
Cold Temp.
Canopy Phen.
Solar Radiation
Under+Can Phen.
Total

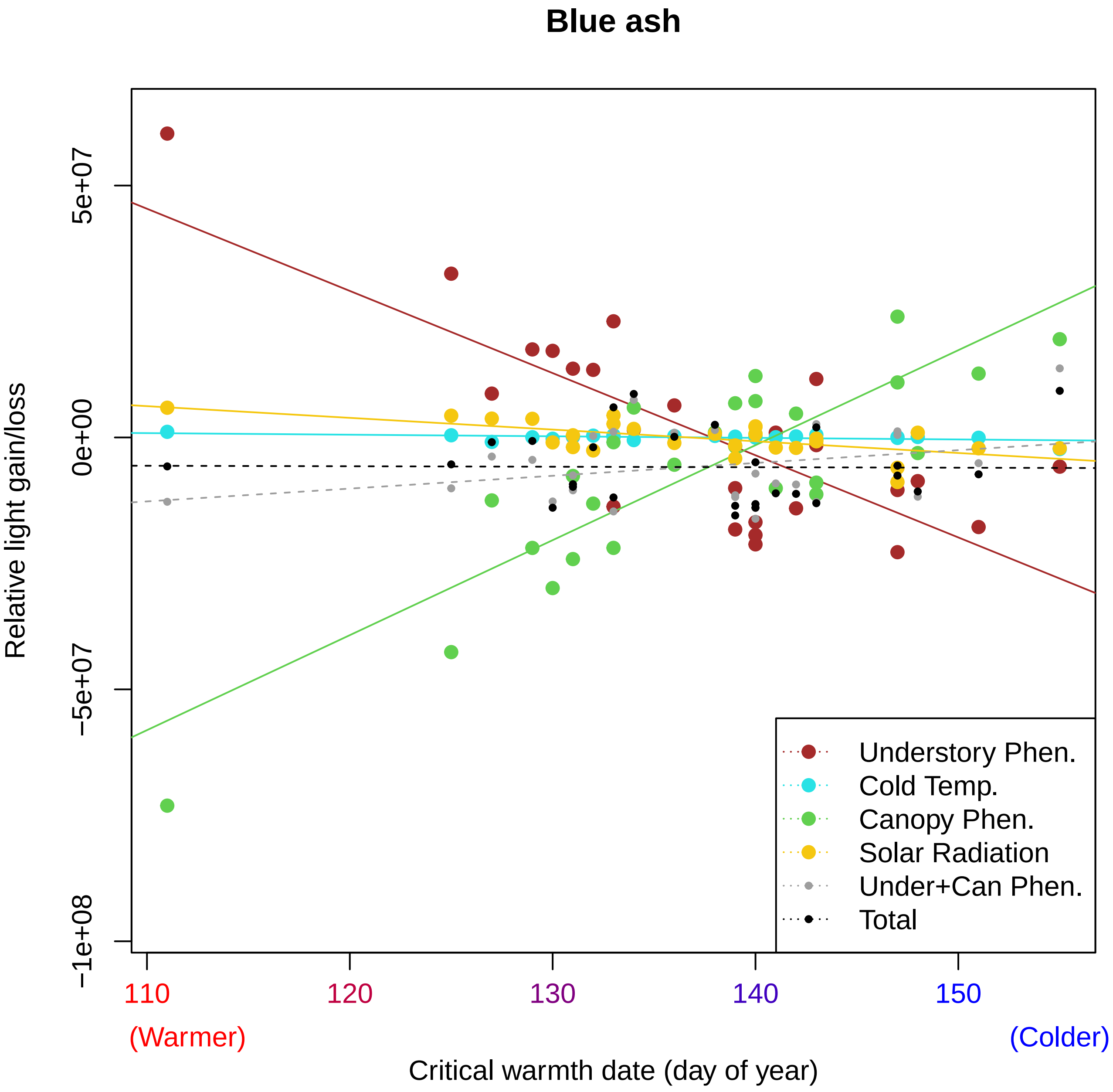

Blue ash
Relative light gain/loss
5e+07
0e+00
−5e+07
−1e+08
110
120
130
140
150
(Warmer)
(Colder)
Critical warmth date (day of year)
Understory Phen.
Cold Temp.
Canopy Phen.
Solar Radiation
Under+Can Phen.
Total
