# Supplementary material for: Understory plants evade shading in a temperate deciduous forest amid climate variability by shifting phenology in synchrony with canopy trees
Source: PLoS One. 2024 Jun 26;19(6):e0306023. doi: 10.1371/journal.pone.0306023 (PMC11207122; doi:10.1371/journal.pone.0306023)
Supplement: S6 Fig — This integrative measure of spring temperature means that warmer springs fall to the left on the x-axis. Solid lines indicate a statistically-significant (p < .05) difference of the estimated slope from 0, while dashed lines indicate that this standard was not met. (PDF) [file pone.0306023.s012.pdf]

Supporting Information 12 for Augspurger CK, Salk CF. Understory plants reduce light loss in a temperate deciduous forest amid climate variability by shifting phenology in synchrony with canopy trees. PLoS One. In review.

Supporting Information 12:
Trends in herb species' light interception as a function of date on which the 48−day running average temperature first exceeded 13° C (see Methods: Section 6). This integrative measure of spring temperature means that warmer springs fall to the left on the x−axis. Solid lines indicate a statistically−significant (p<.05) difference of the estimated slope from 0, while dashed lines indicate that this standard was not met.

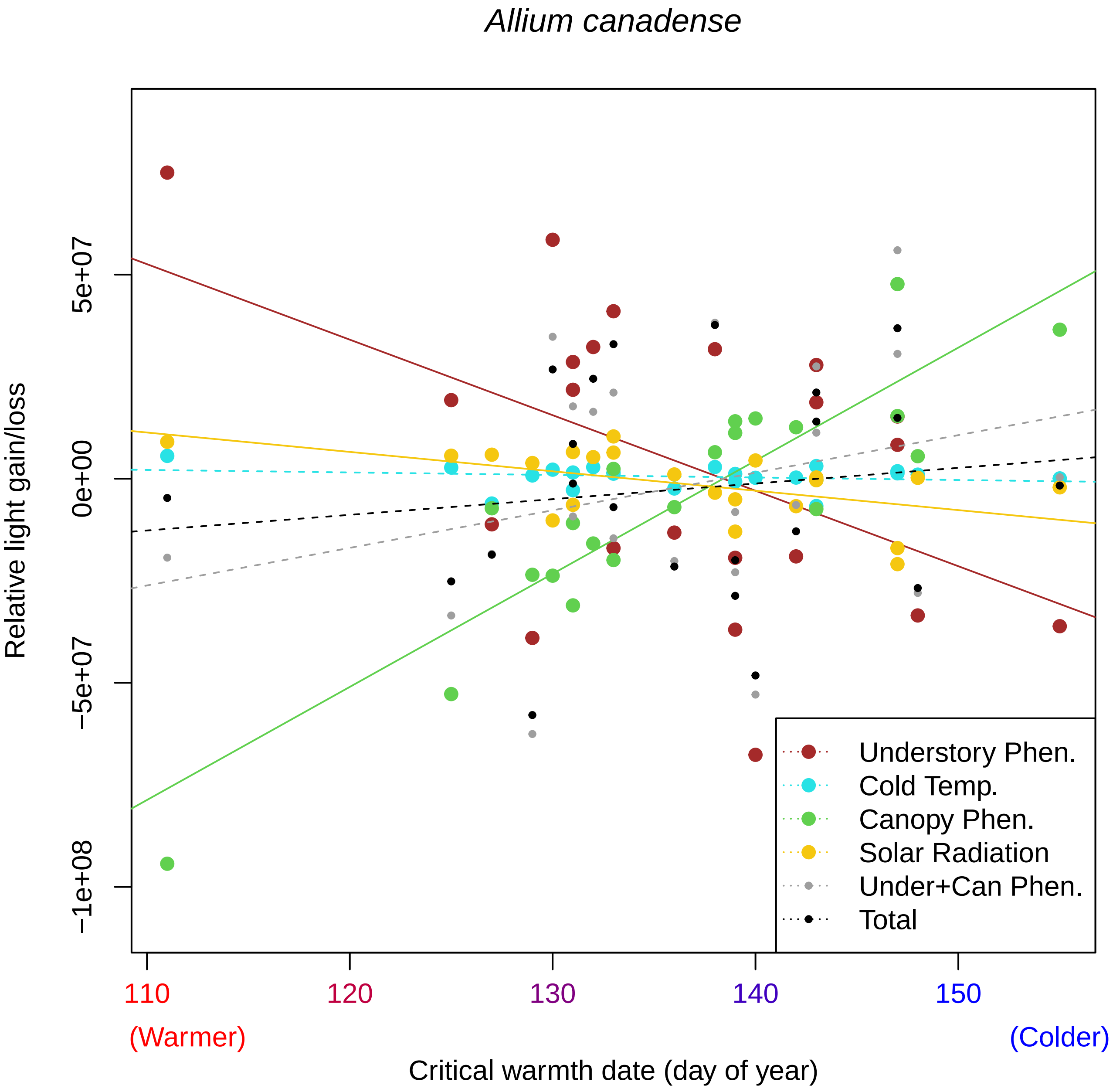

Allium canadense
5e+07
0e+00
−5e+07
−1e+08
Relative light gain/loss
110
120
130
140
150
(Warmer)
(Colder)
Critical warmth date (day of year)
Understory Phen.
Cold Temp.
Canopy Phen.
Solar Radiation
Under+Can Phen.
Total

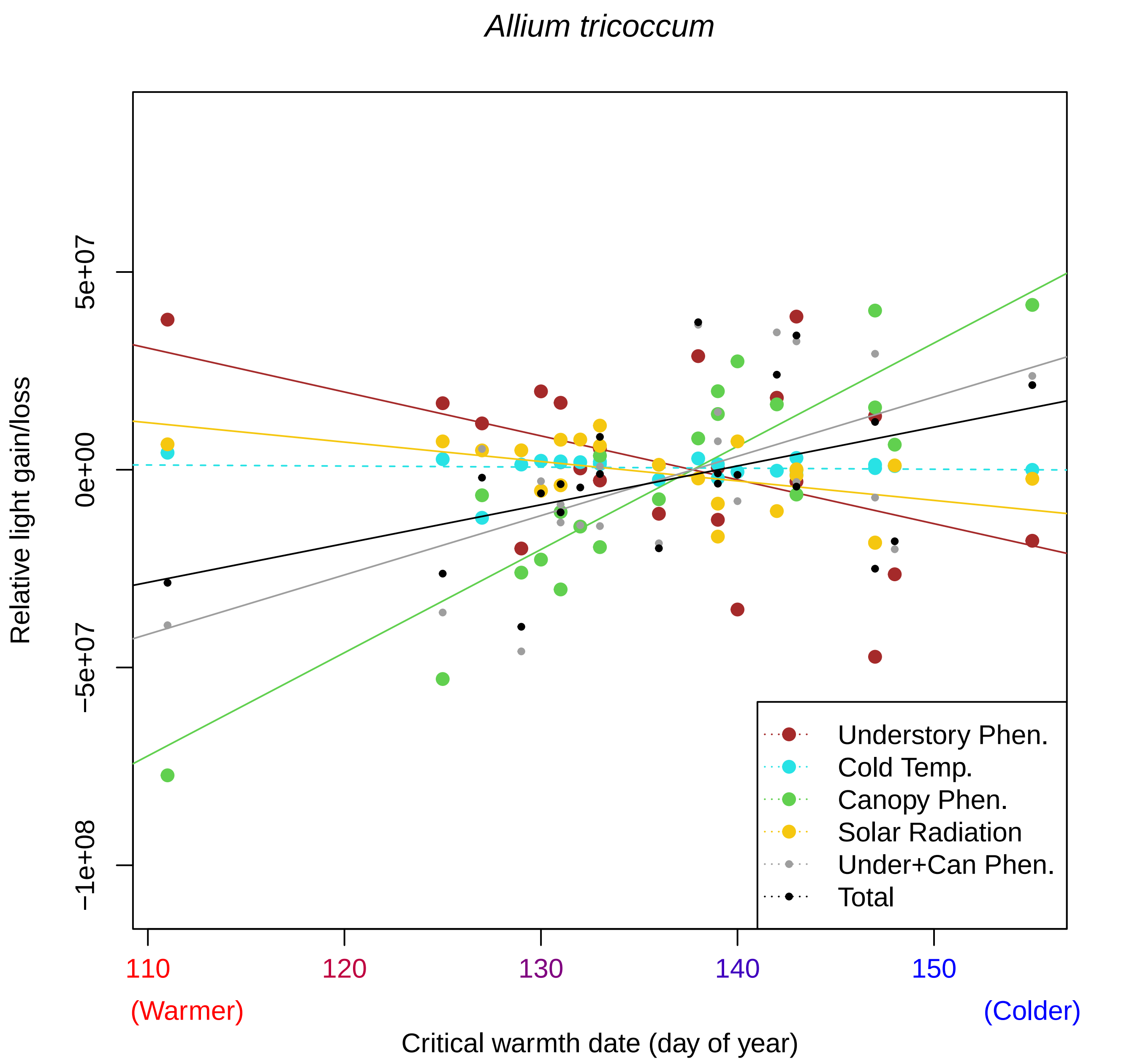

Allium tricoccum
Relative light gain/loss
5e+07
0e+00
−5e+07
−1e+08
110
120
130
140
150
(Warmer)
(Colder)
Critical warmth date (day of year)
Understory Phen.
Cold Temp.
Canopy Phen.
Solar Radiation
Under+Can Phen.
Total

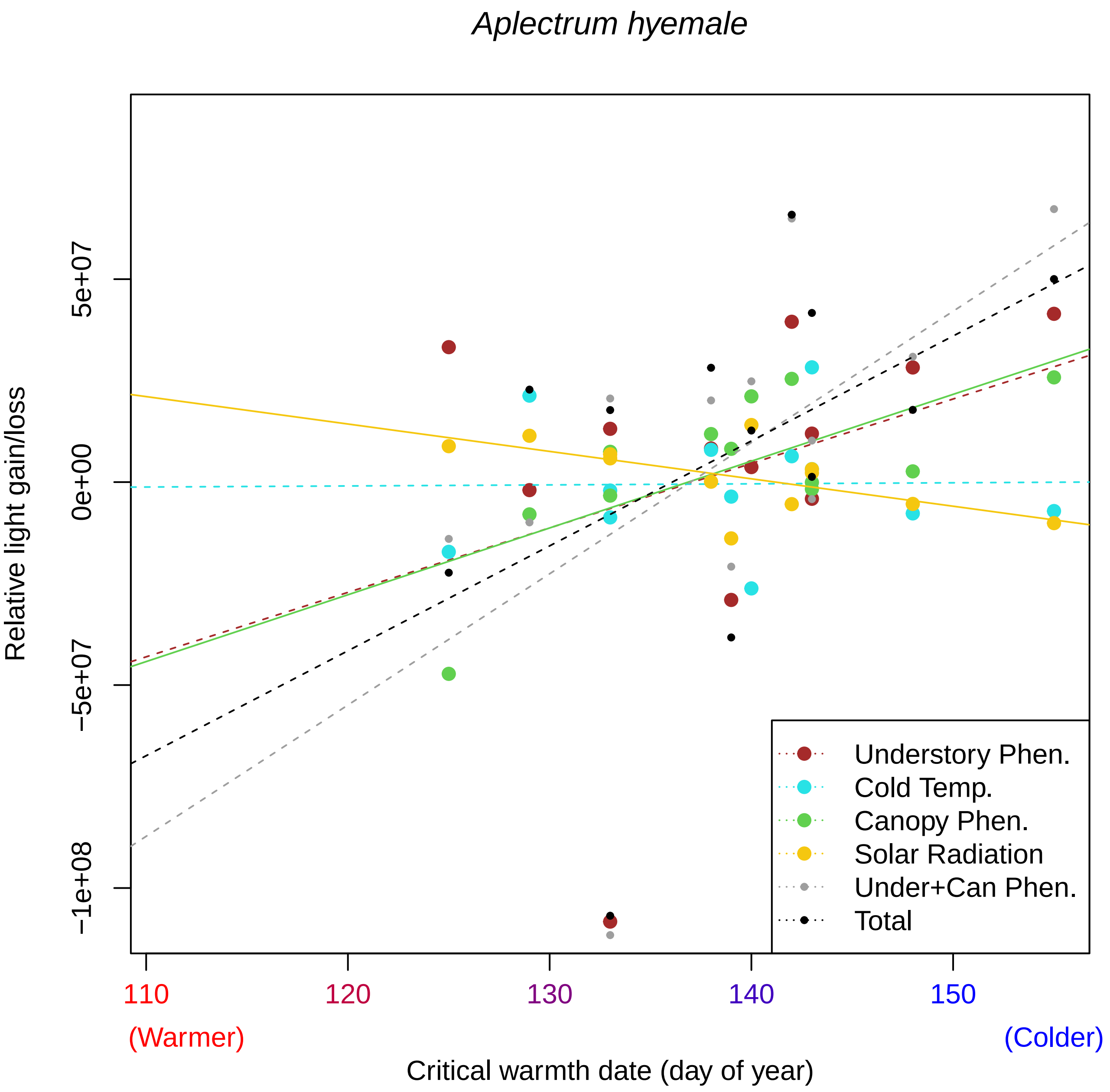

Aplectrum hyemale
Relative light gain/loss
5e+07
0e+00
−5e+07
−1e+08
110
120
130
140
150
(Warmer)
(Colder)
Critical warmth date (day of year)
Understory Phen.
Cold Temp.
Canopy Phen.
Solar Radiation
Under+Can Phen.
Total

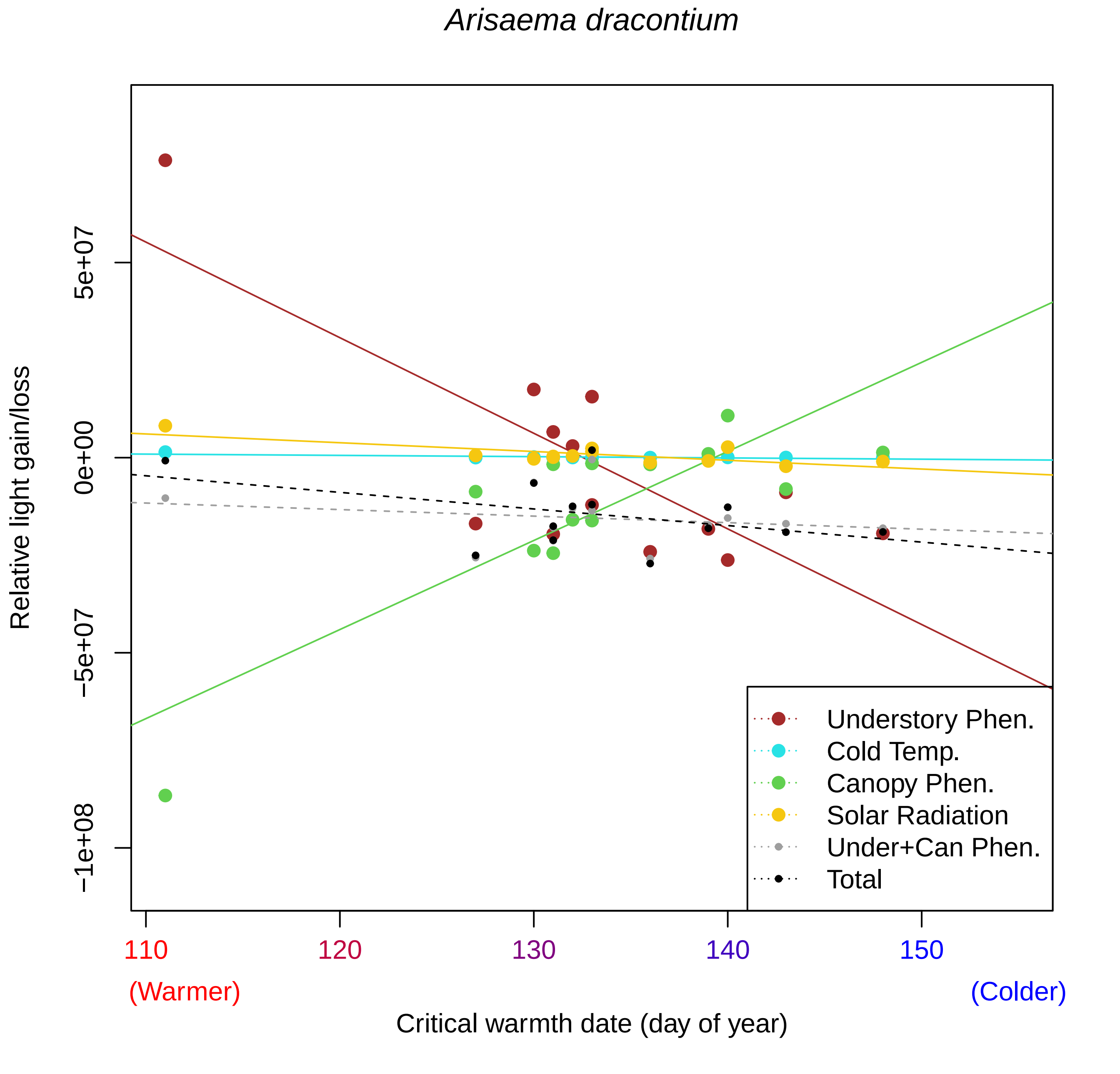
Arisaema dracontium
Relative light gain/loss
5e+07
0e+00
−5e+07
−1e+08
Understory Phen.
Cold Temp.
Canopy Phen.
Solar Radiation
Under+Can Phen.
Total
110
120
130
140
150
(Warmer)
(Colder)
Critical warmth date (day of year)

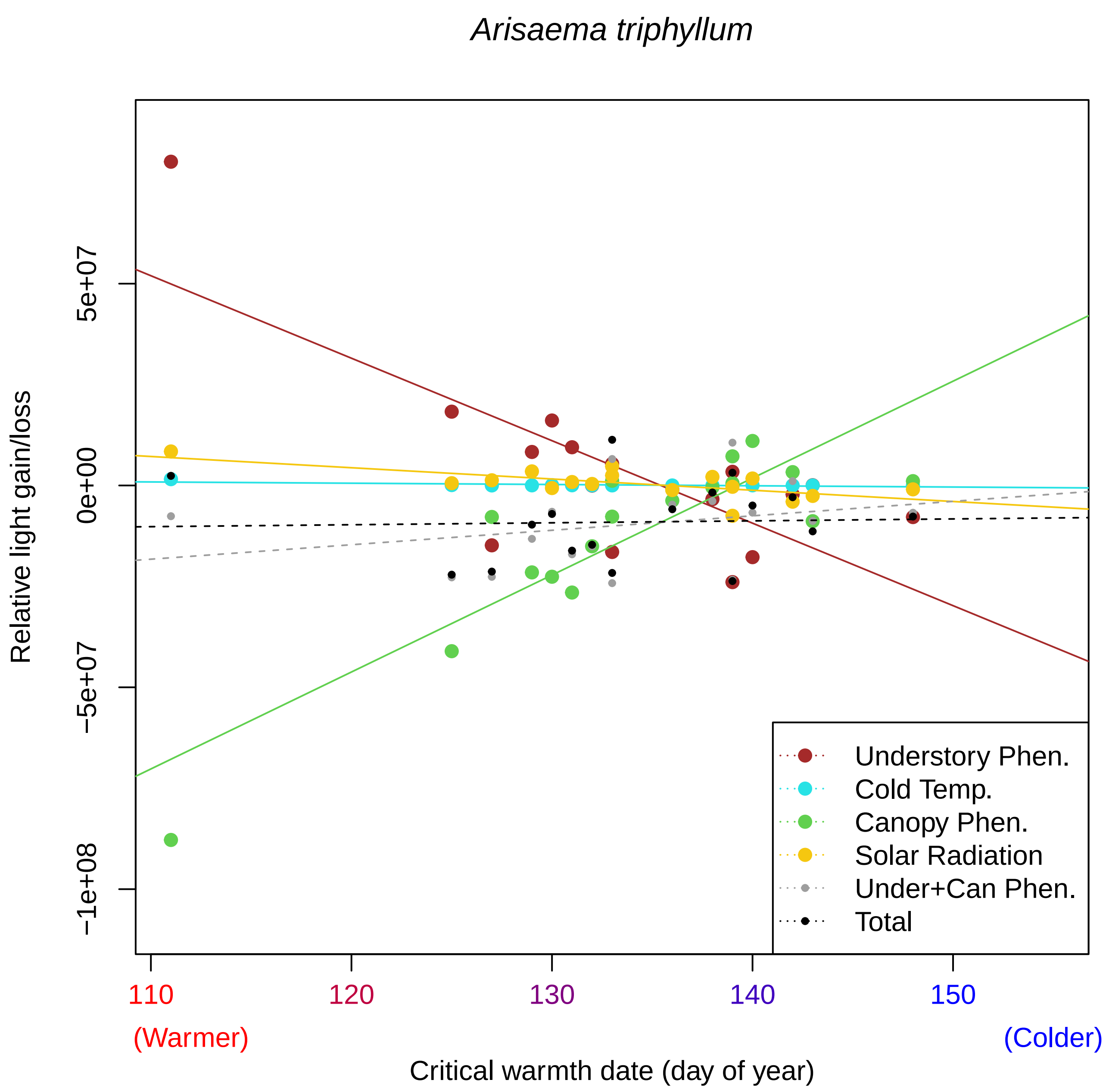

Arisaema triphyllum
Relative light gain/loss
5e+07
0e+00
−5e+07
−1e+08
Understory Phen.
Cold Temp.
Canopy Phen.
Solar Radiation
Under+Can Phen.
Total
110
120
130
140
150
(Warmer)
(Colder)
Critical warmth date (day of year)

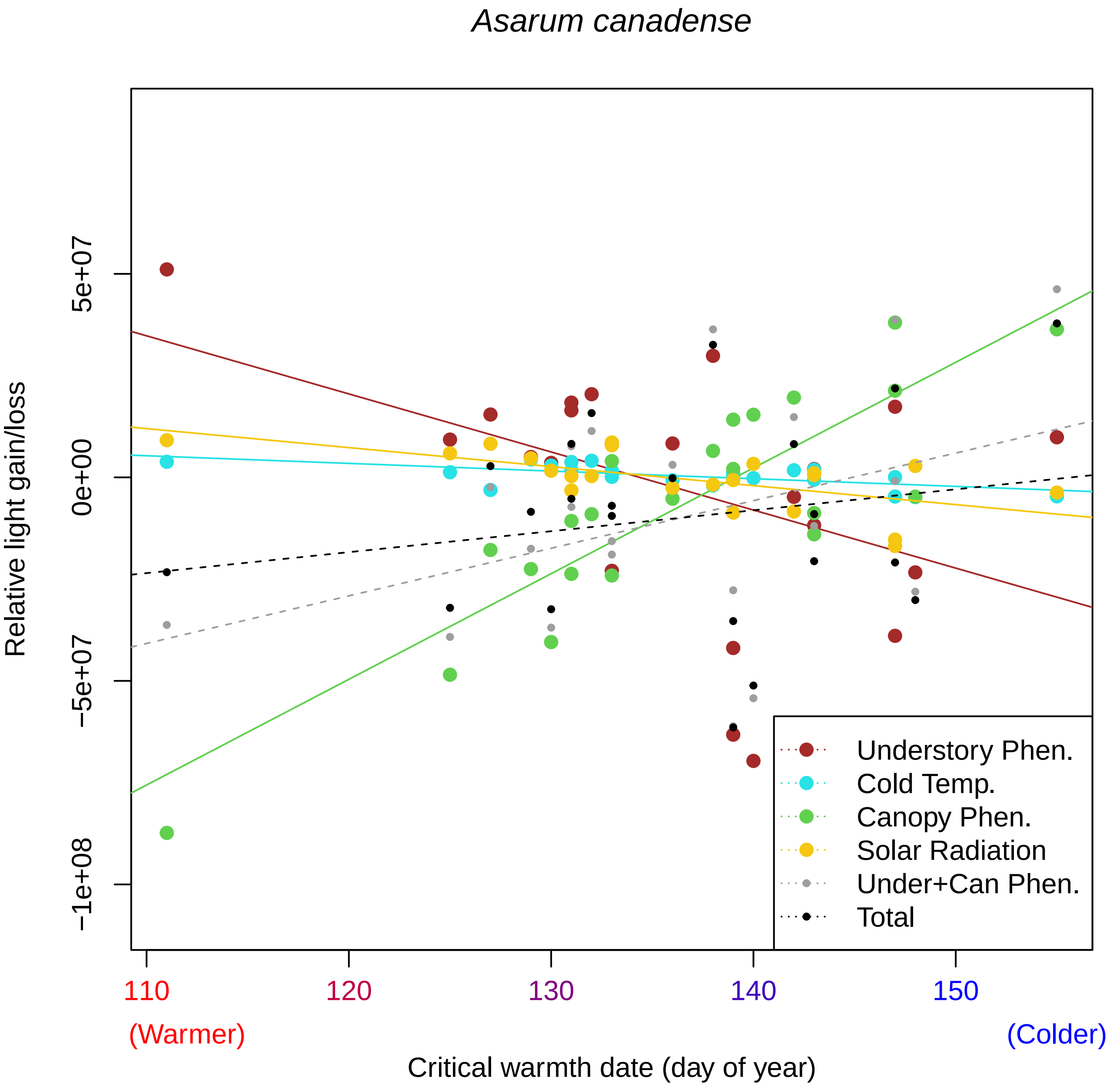

Asarum canadense
Relative light gain/loss
5e+07
0e+00
−5e+07
−1e+08
110
120
130
140
150
(Warmer)
(Colder)
Critical warmth date (day of year)
Understory Phen.
Cold Temp.
Canopy Phen.
Solar Radiation
Under+Can Phen.
Total

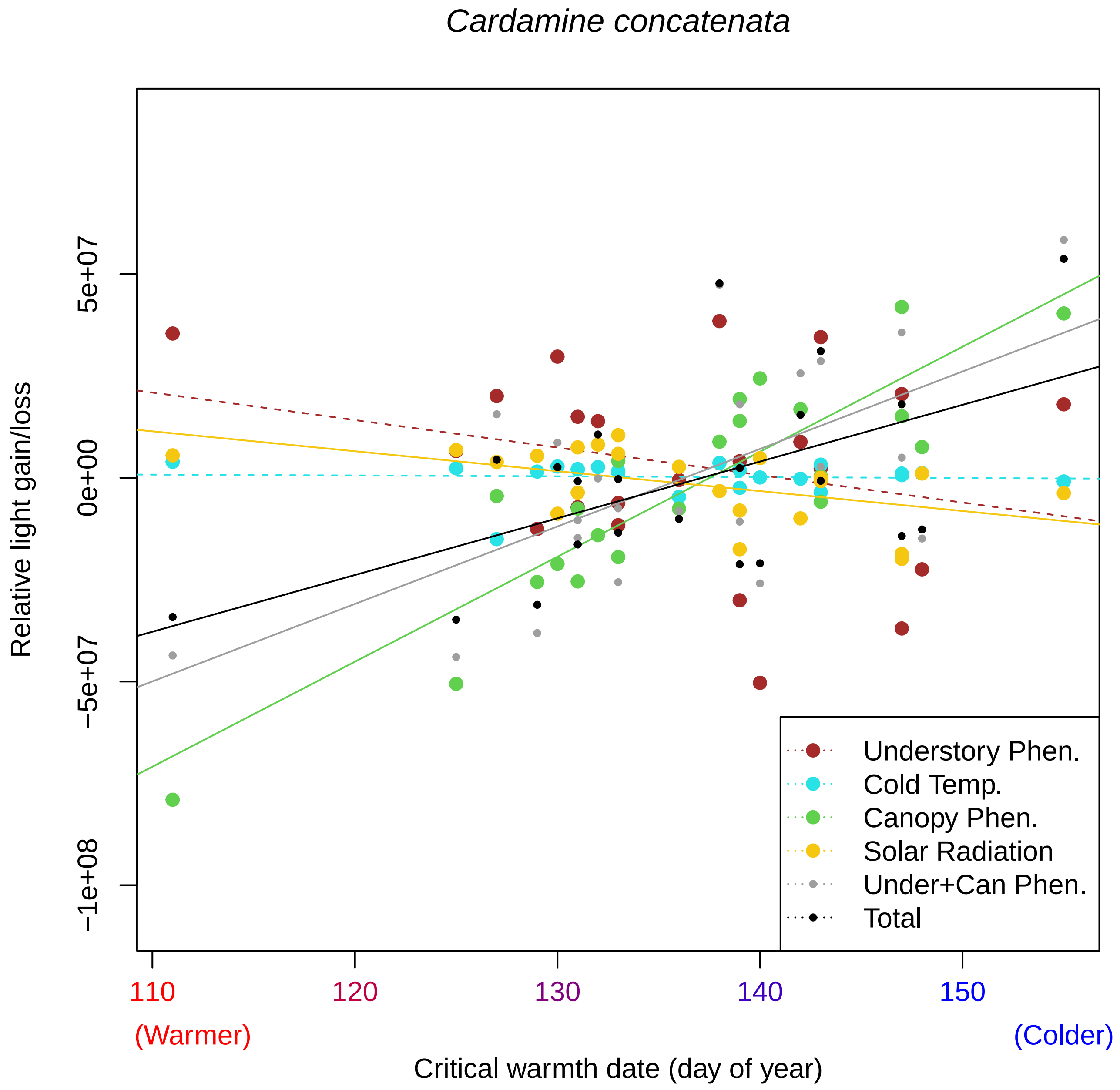

Cardamine concatenata
Relative light gain/loss
5e+07
0e+00
−5e+07
−1e+08
110
120
130
140
150
(Warmer)
(Colder)
Critical warmth date (day of year)
Understory Phen.
Cold Temp.
Canopy Phen.
Solar Radiation
Under+Can Phen.
Total

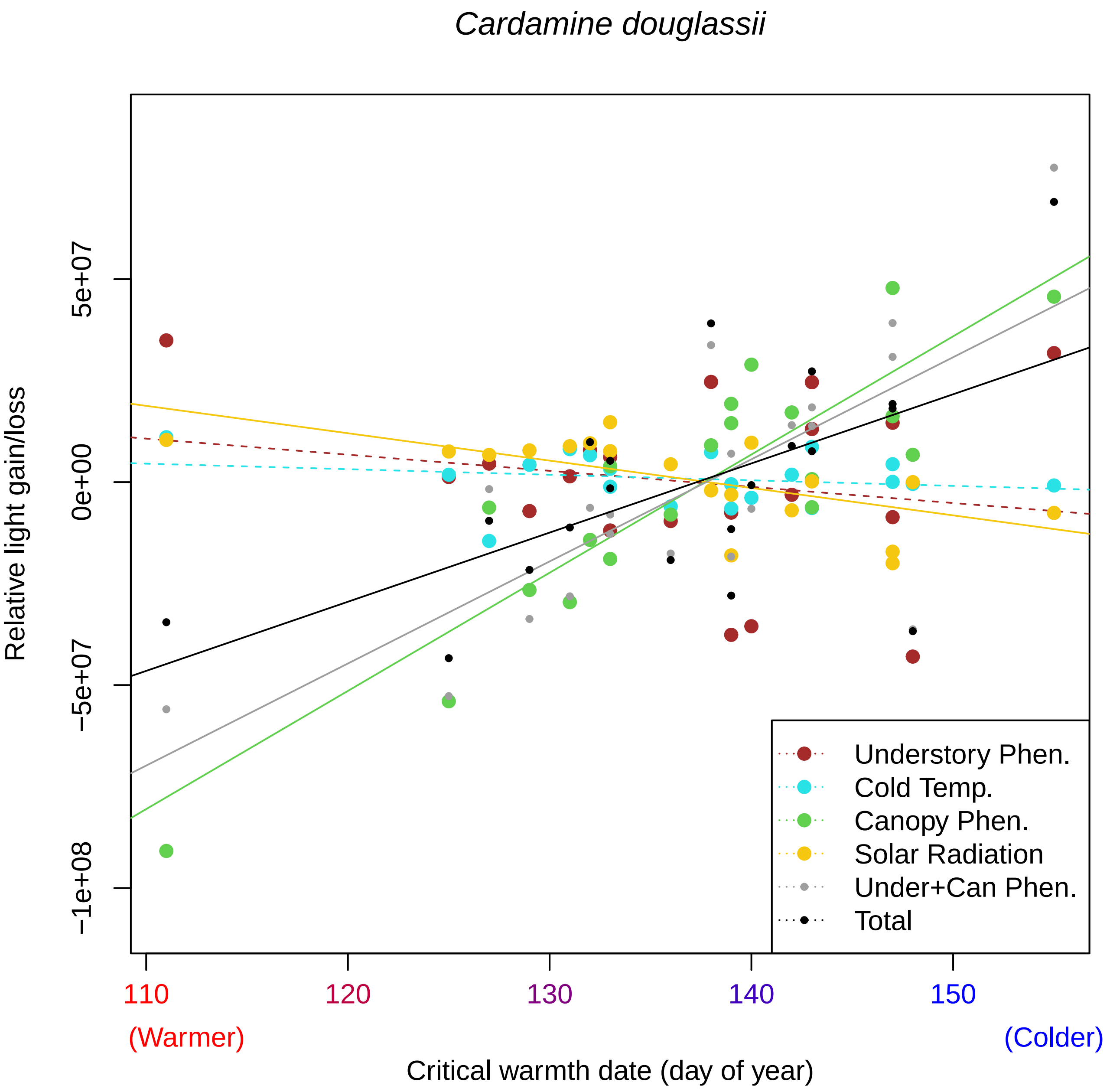

Cardamine douglassii
Relative light gain/loss
5e+07
0e+00
−5e+07
−1e+08
110
120
130
140
150
(Warmer)
(Colder)
Critical warmth date (day of year)
Understory Phen.
Cold Temp.
Canopy Phen.
Solar Radiation
Under+Can Phen.
Total

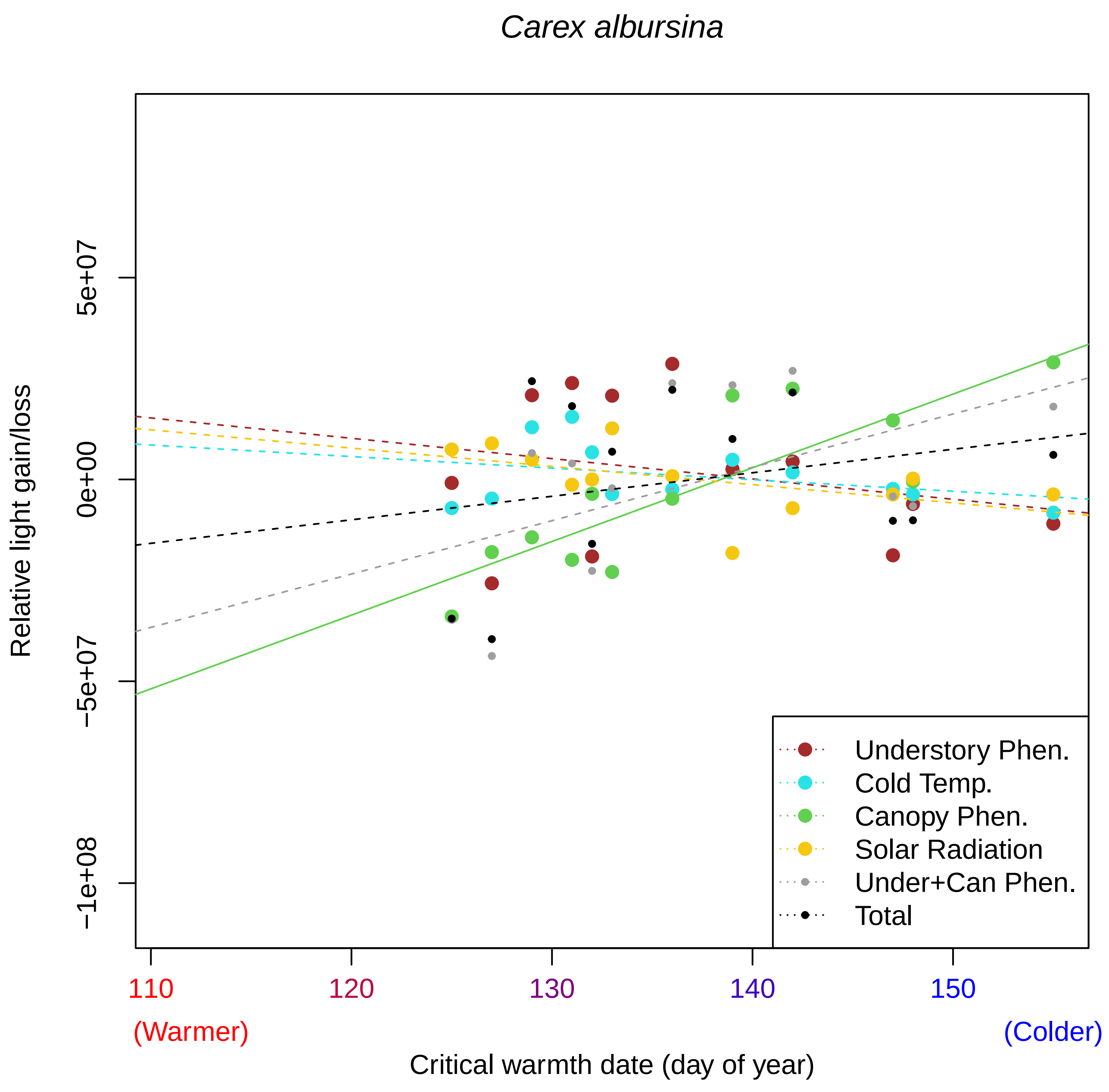

Carex alburSina
5e+07
0e+00
−5e+07
−1e+08
Relative light gain/loss
110
120
130
140
150
(Warmer)
(Colder)
Critical warmth date (day of year)
Understory Phen.
Cold Temp.
Canopy Phen.
Solar Radiation
Under+Can Phen.
Total

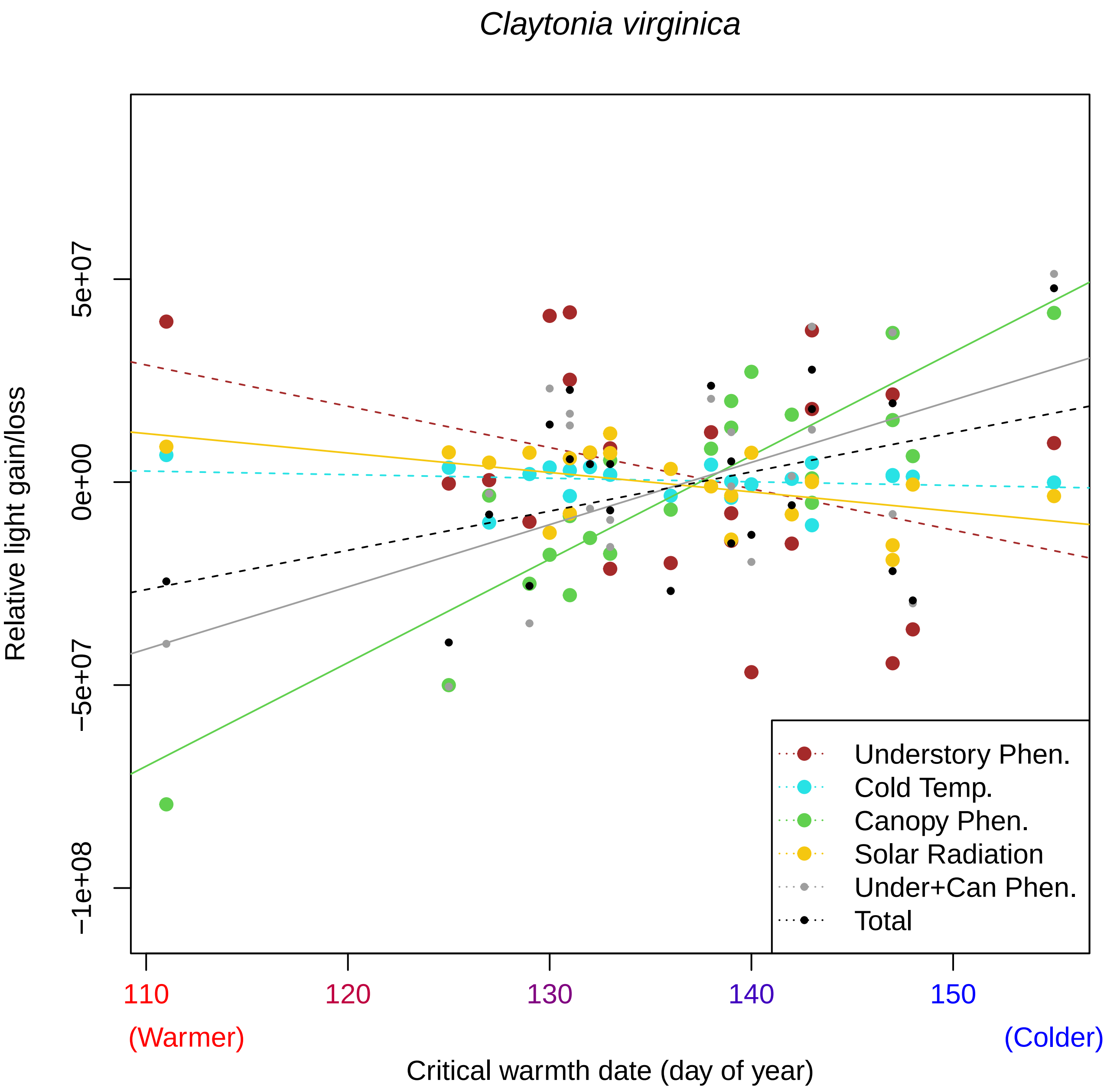

Claytonia virginica
Relative light gain/loss
5e+07
0e+00
−5e+07
−1e+08
110
120
130
140
150
(Warmer)
(Colder)
Critical warmth date (day of year)
Understory Phen.
Cold Temp.
Canopy Phen.
Solar Radiation
Under+Can Phen.
Total

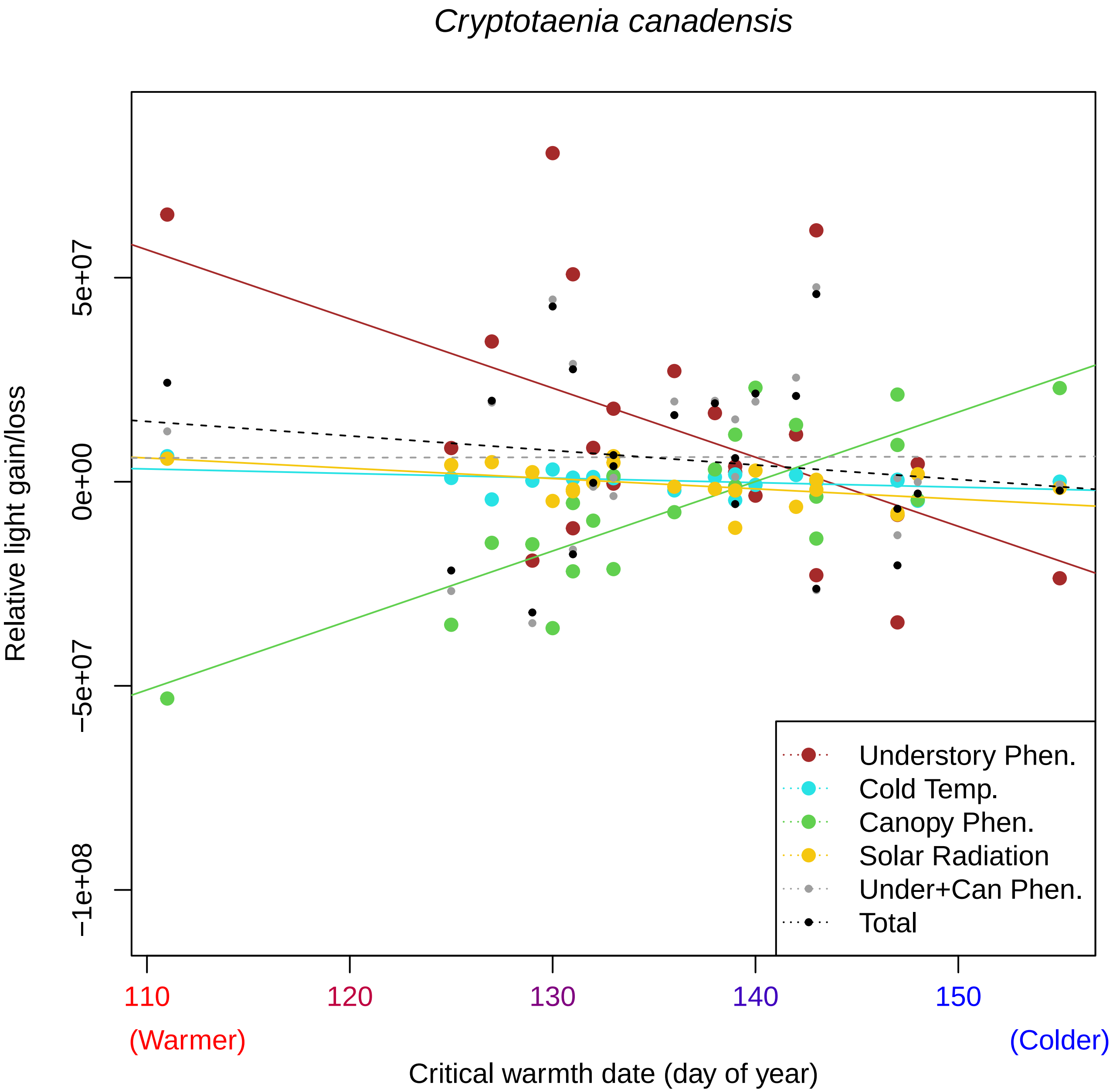

Cryptotaenia canadensis
Relative light gain/loss
5e+07
0e+00
−5e+07
−1e+08
110
120
130
140
150
(Warmer)
(Colder)
Critical warmth date (day of year)
Understory Phen.
Cold Temp.
Canopy Phen.
Solar Radiation
Under+Can Phen.
Total

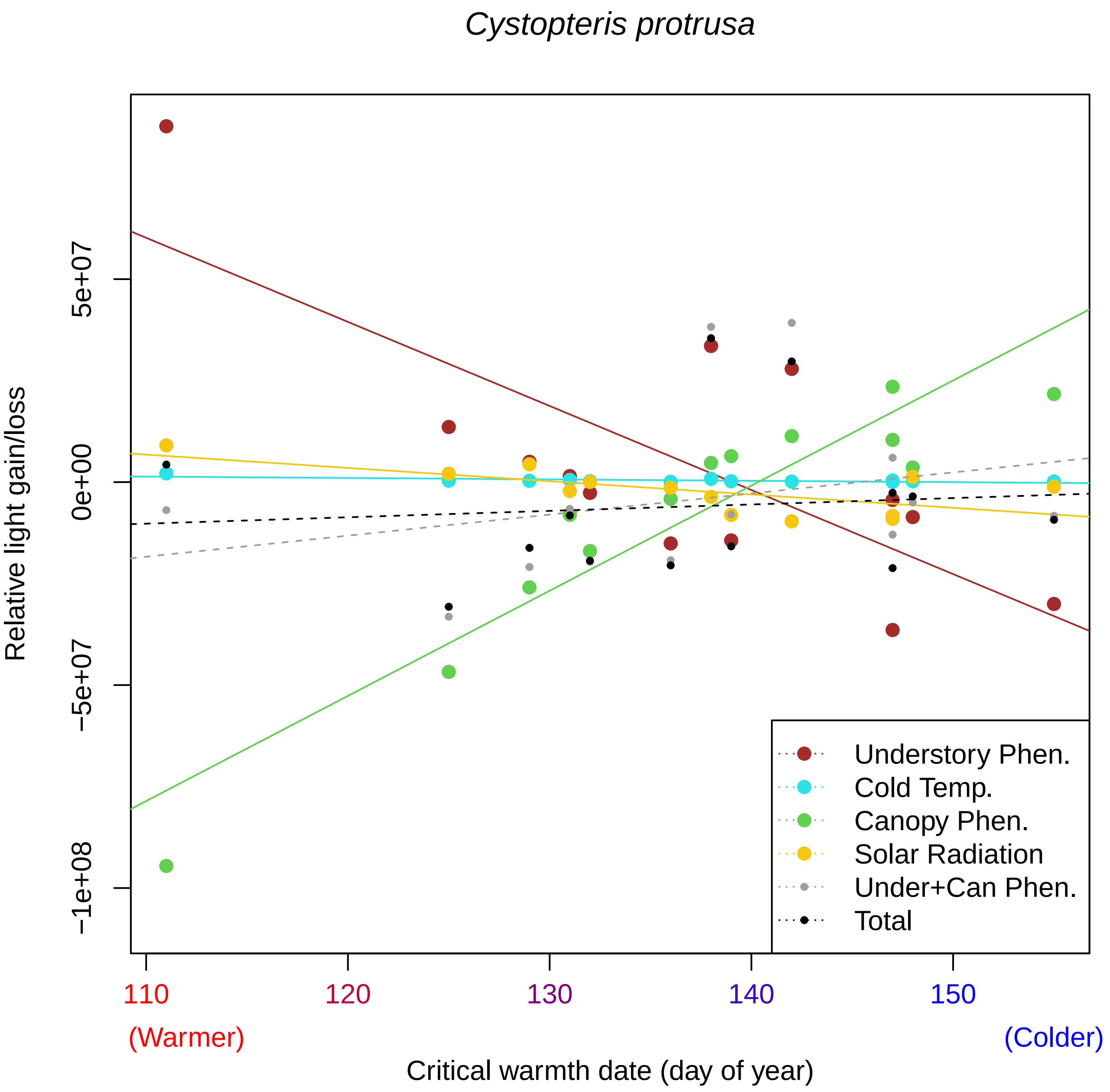

Cystopteris protrusa
Relative light gain/loss
5e+07
0e+00
−5e+07
−1e+08
110
120
130
140
150
(Warmer)
(Colder)
Critical warmth date (day of year)
Understory Phen.
Cold Temp.
Canopy Phen.
Solar Radiation
Under+Can Phen.
Total

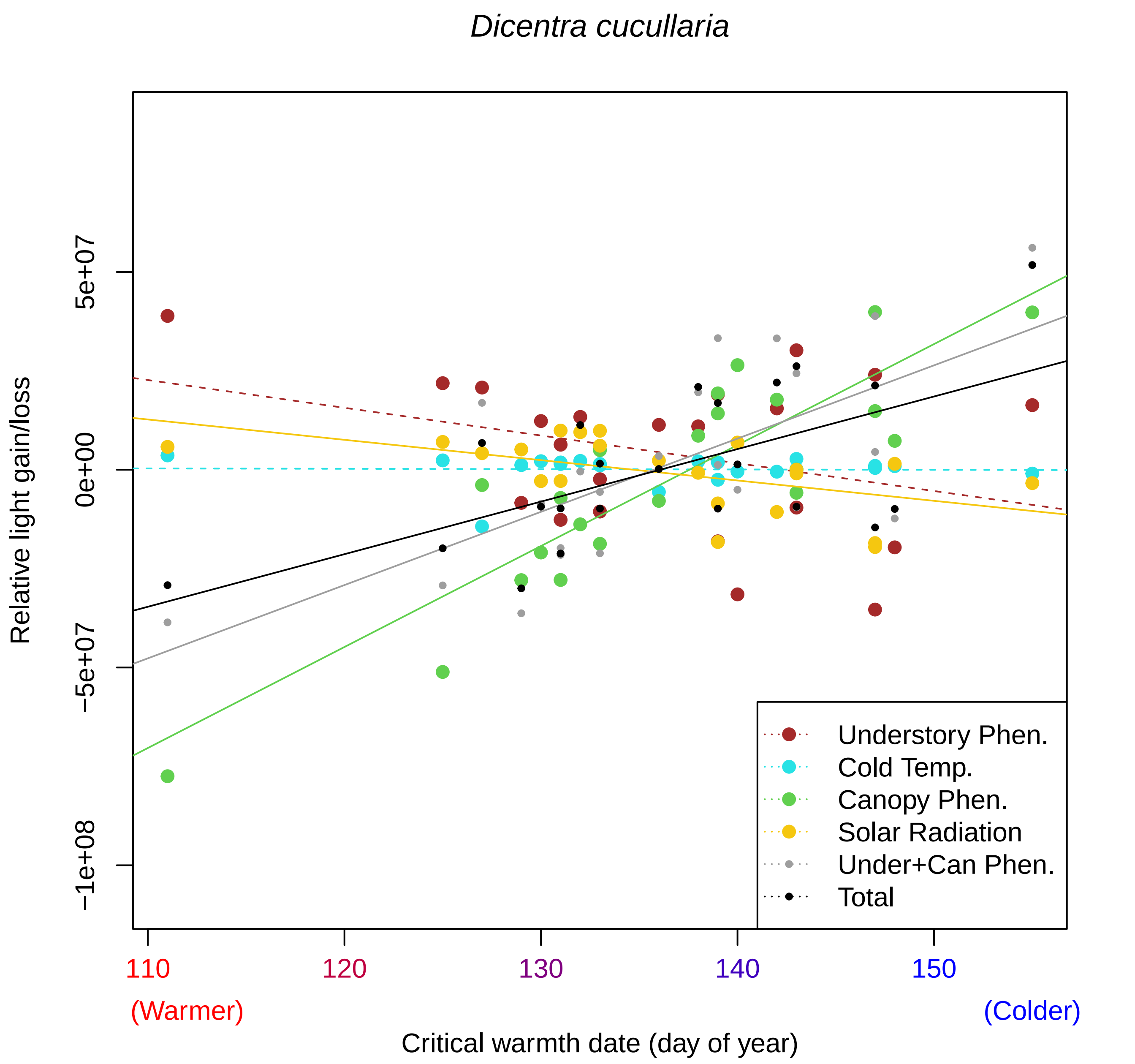

Dicentra cucullaria
Relative light gain/loss
5e+07
0e+00
−5e+07
−1e+08
110
120
130
140
150
(Warmer)
(Colder)
Critical warmth date (day of year)
Understory Phen.
Cold Temp.
Canopy Phen.
Solar Radiation
Under+Can Phen.
Total

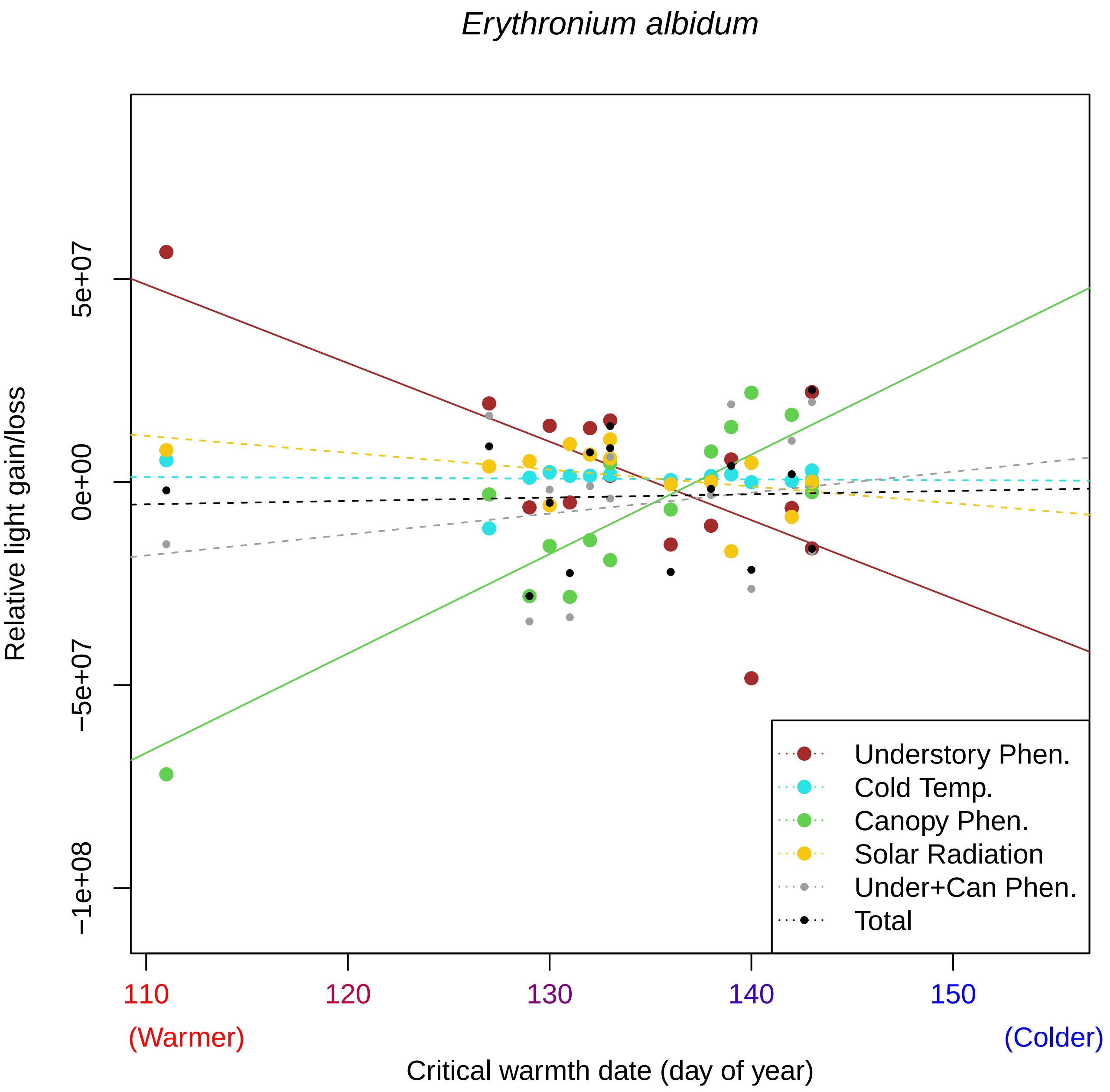

Erythronium albidum
Relative light gain/loss
5e+07
0e+00
−5e+07
−1e+08
110
120
130
140
150
(Warmer)
(Colder)
Critical warmth date (day of year)
Understory Phen.
Cold Temp.
Canopy Phen.
Solar Radiation
Under+Can Phen.
Total

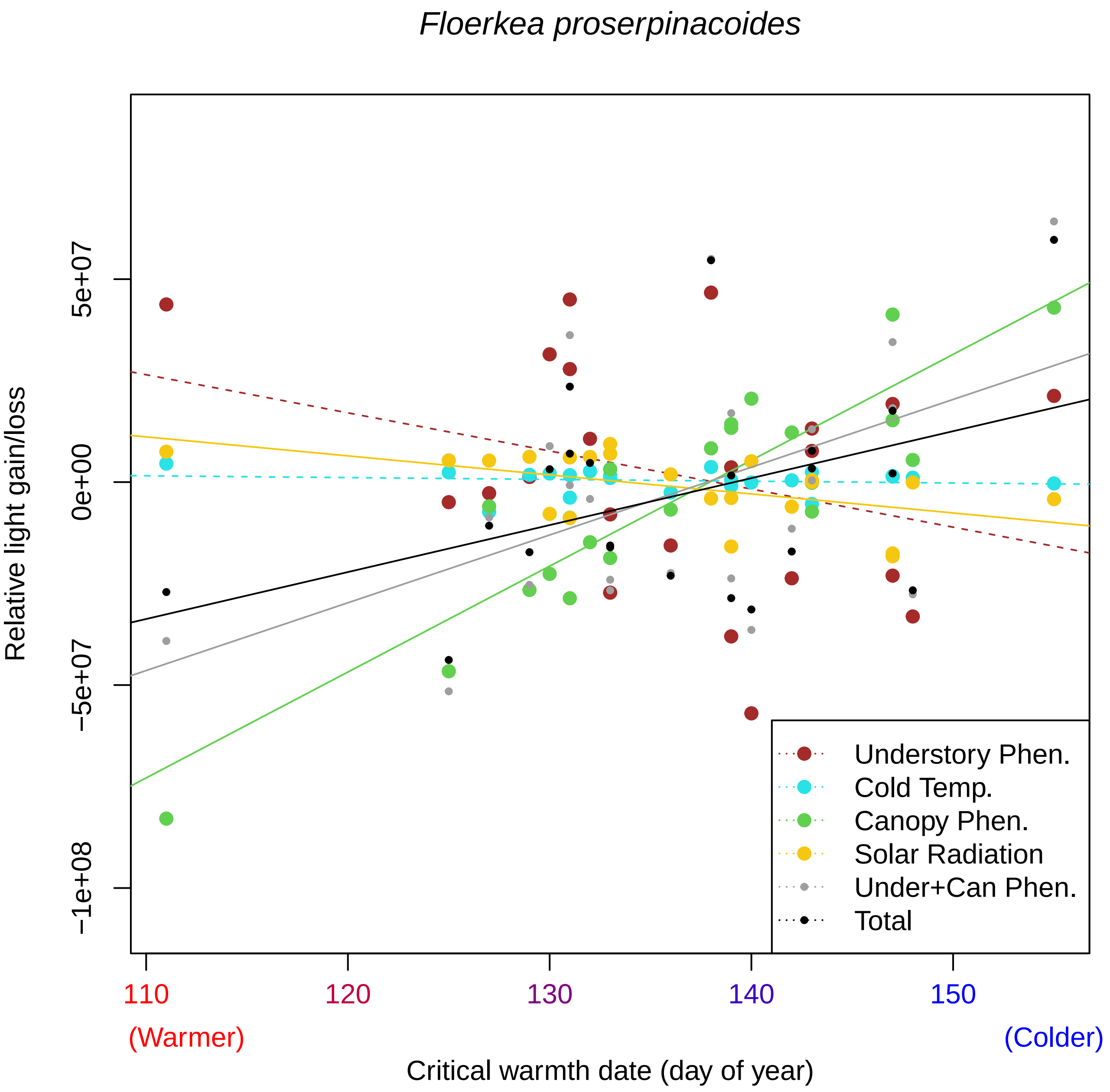

Floerkea proserpinacoides
Relative light gain/loss
5e+07
0e+00
−5e+07
−1e+08
110
120
130
140
150
(Warmer)
(Colder)
Critical warmth date (day of year)
Understory Phen.
Cold Temp.
Canopy Phen.
Solar Radiation
Under+Can Phen.
Total

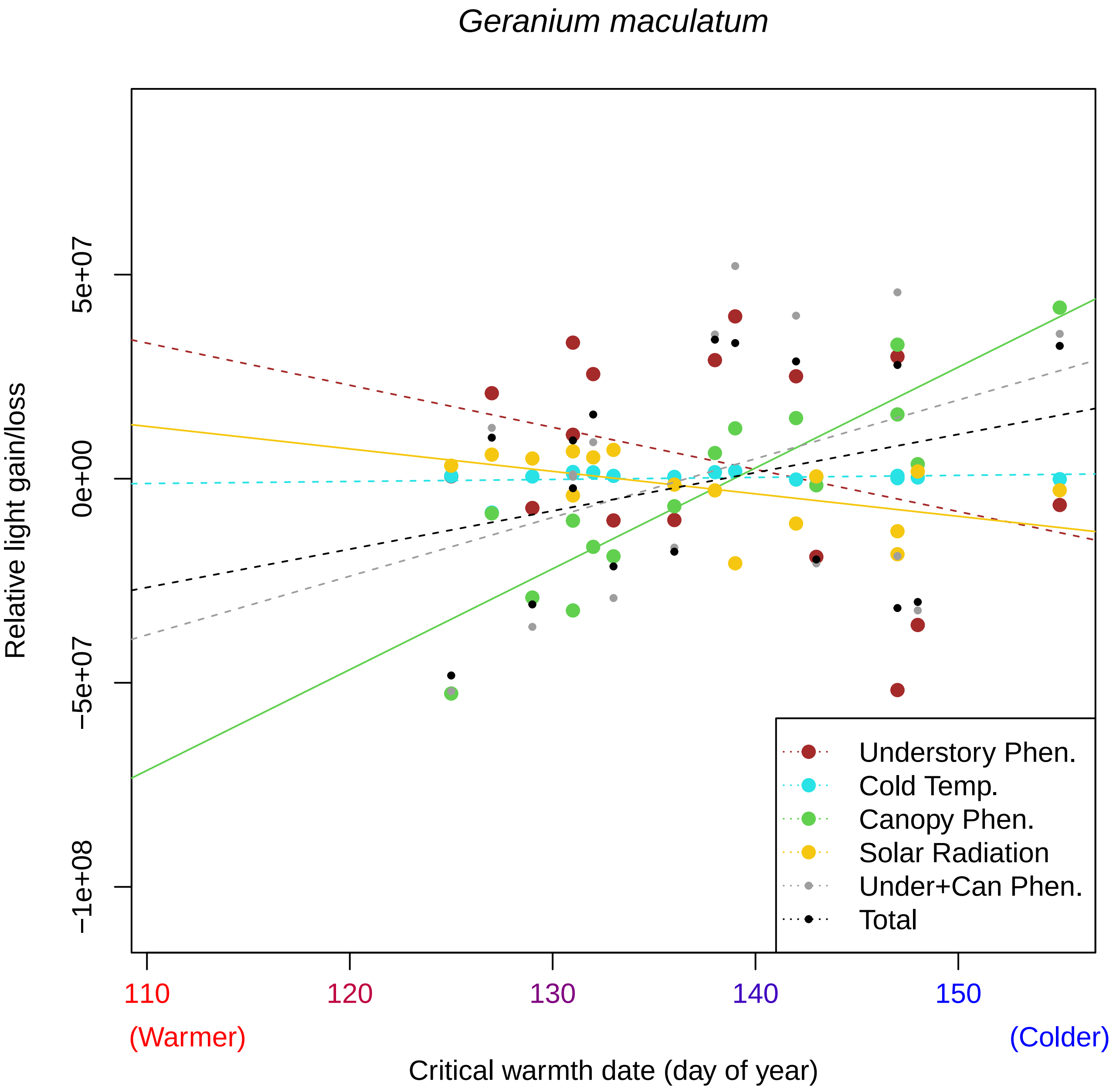

Geranium maculatum
Relative light gain/loss
5e+07
0e+00
−5e+07
−1e+08
110
120
130
140
150
(Warmer)
(Colder)
Critical warmth date (day of year)
Understory Phen.
Cold Temp.
Canopy Phen.
Solar Radiation
Under+Can Phen.
Total

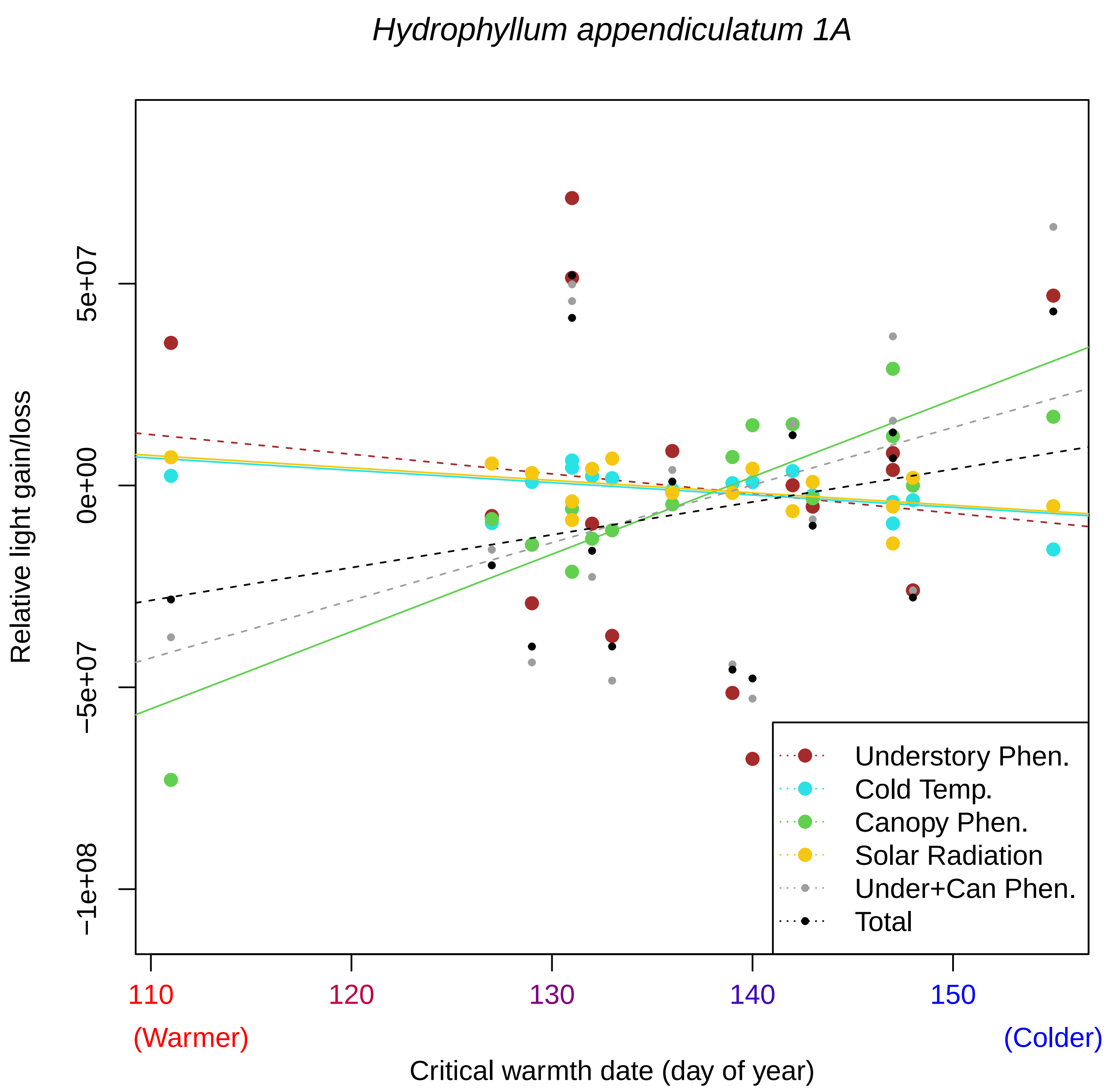

Hydrophyllum appendiculatum 1A
Relative light gain/loss
5e+07
0e+00
−5e+07
−1e+08
110
120
130
140
150
(Warmer)
(Colder)
Critical warmth date (day of year)
Understory Phen.
Cold Temp.
Canopy Phen.
Solar Radiation
Under+Can Phen.
Total

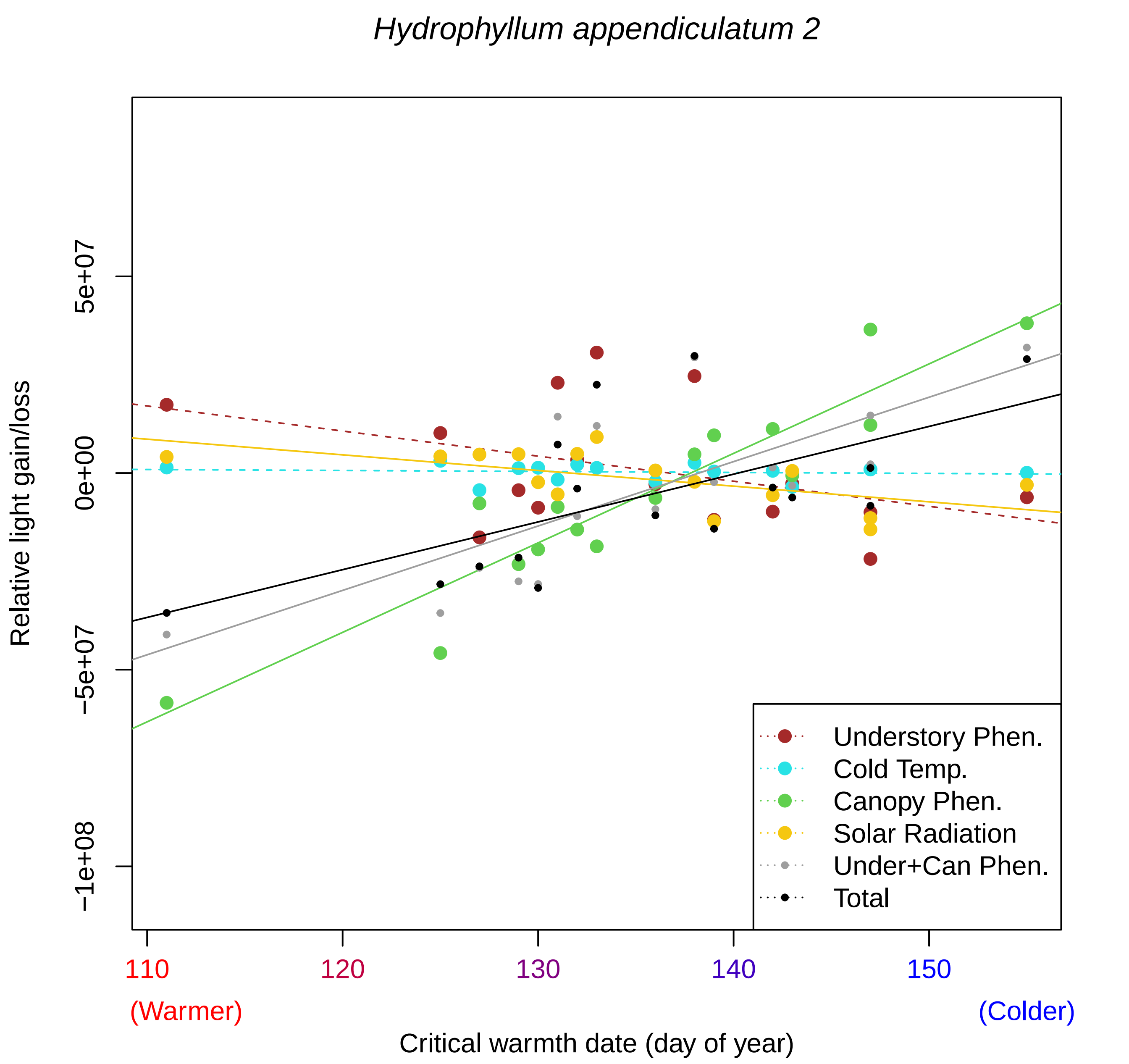

Hydrophyllum appendiculatum 2
Relative light gain/loss
5e+07
0e+00
−5e+07
−1e+08
110
120
130
140
150
(Warmer)
(Colder)
Critical warmth date (day of year)
Understory Phen.
Cold Temp.
Canopy Phen.
Solar Radiation
Under+Can Phen.
Total

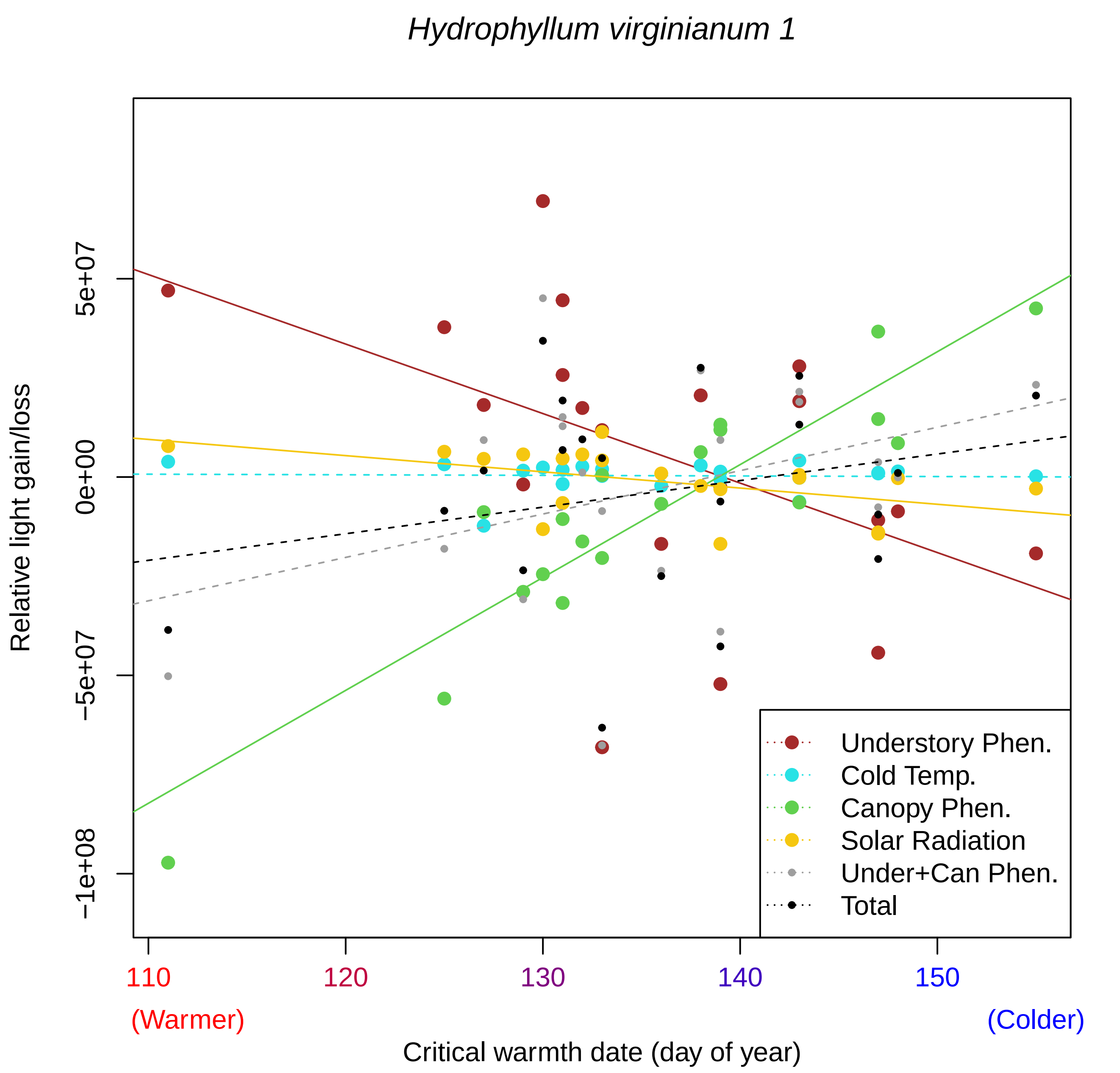

Hydrophyllum virginianum 1
Relative light gain/loss
5e+07
0e+00
−5e+07
−1e+08
110
120
130
140
150
(Warmer)
(Colder)
Critical warmth date (day of year)
Understory Phen.
Cold Temp.
Canopy Phen.
Solar Radiation
Under+Can Phen.
Total

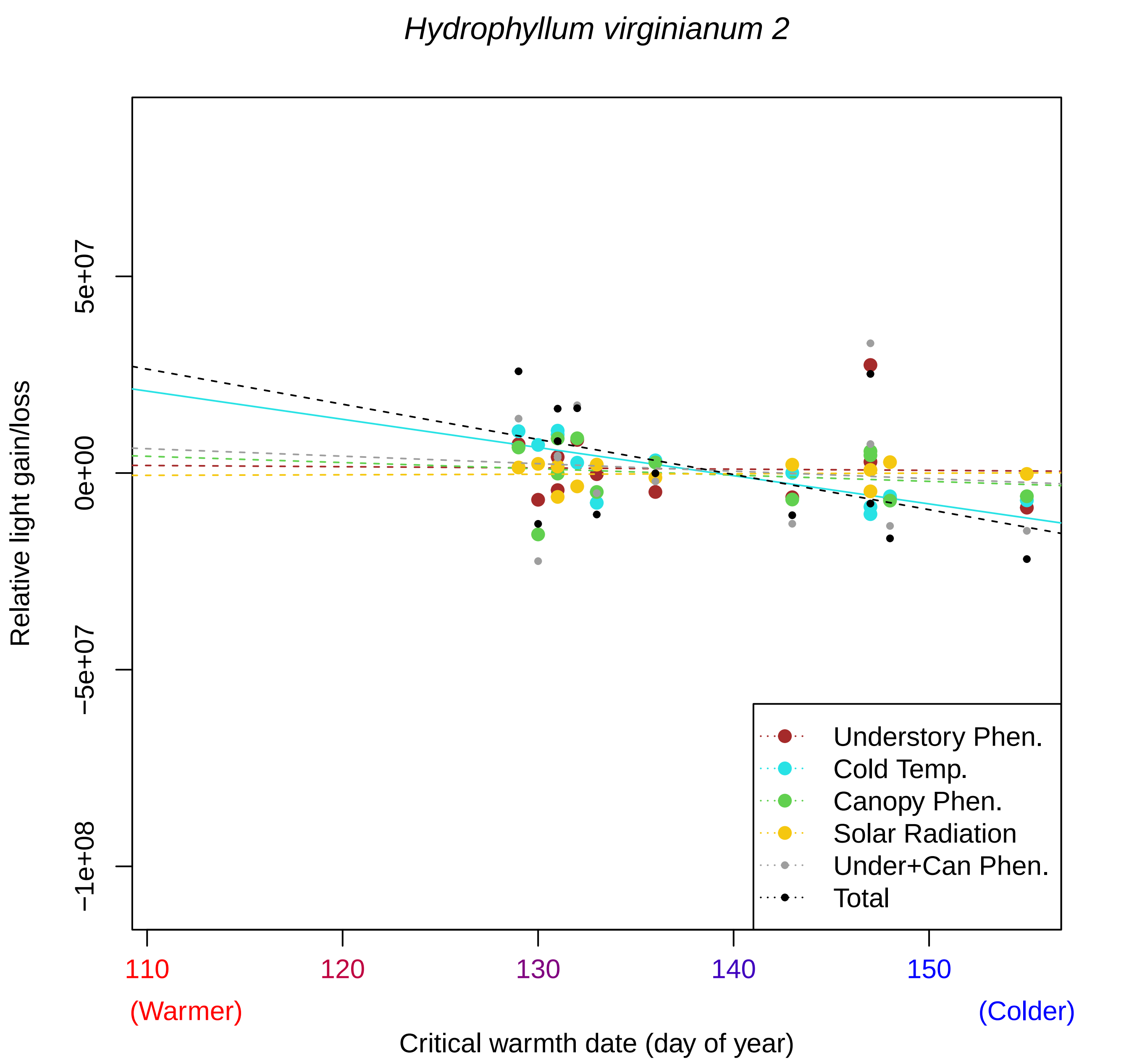

Hydrophyllum virginianum 2
5e+07
0e+00
−5e+07
−1e+08
Relative light gain/loss
Understory Phen.
Cold Temp.
Canopy Phen.
Solar Radiation
Under+Can Phen.
Total
110
120
130
140
150
(Warmer)
(Colder)
Critical warmth date (day of year)

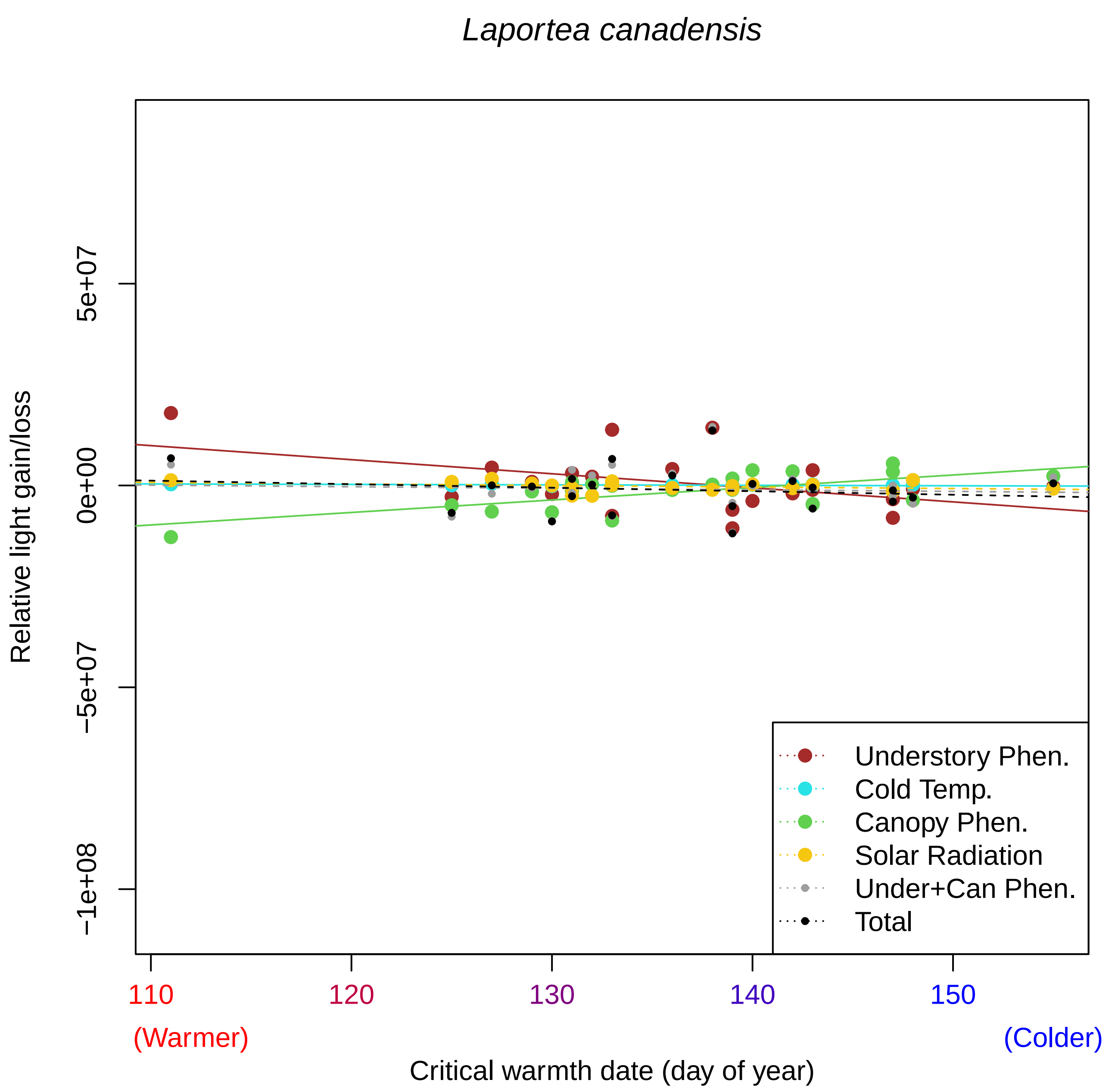

Laportea canadensis
5e+07
0e+00
−5e+07
−1e+08
Relative light gain/loss
110
120
130
140
150
(Warmer)
(Colder)
Critical warmth date (day of year)
Understory Phen.
Cold Temp.
Canopy Phen.
Solar Radiation
Under+Can Phen.
Total

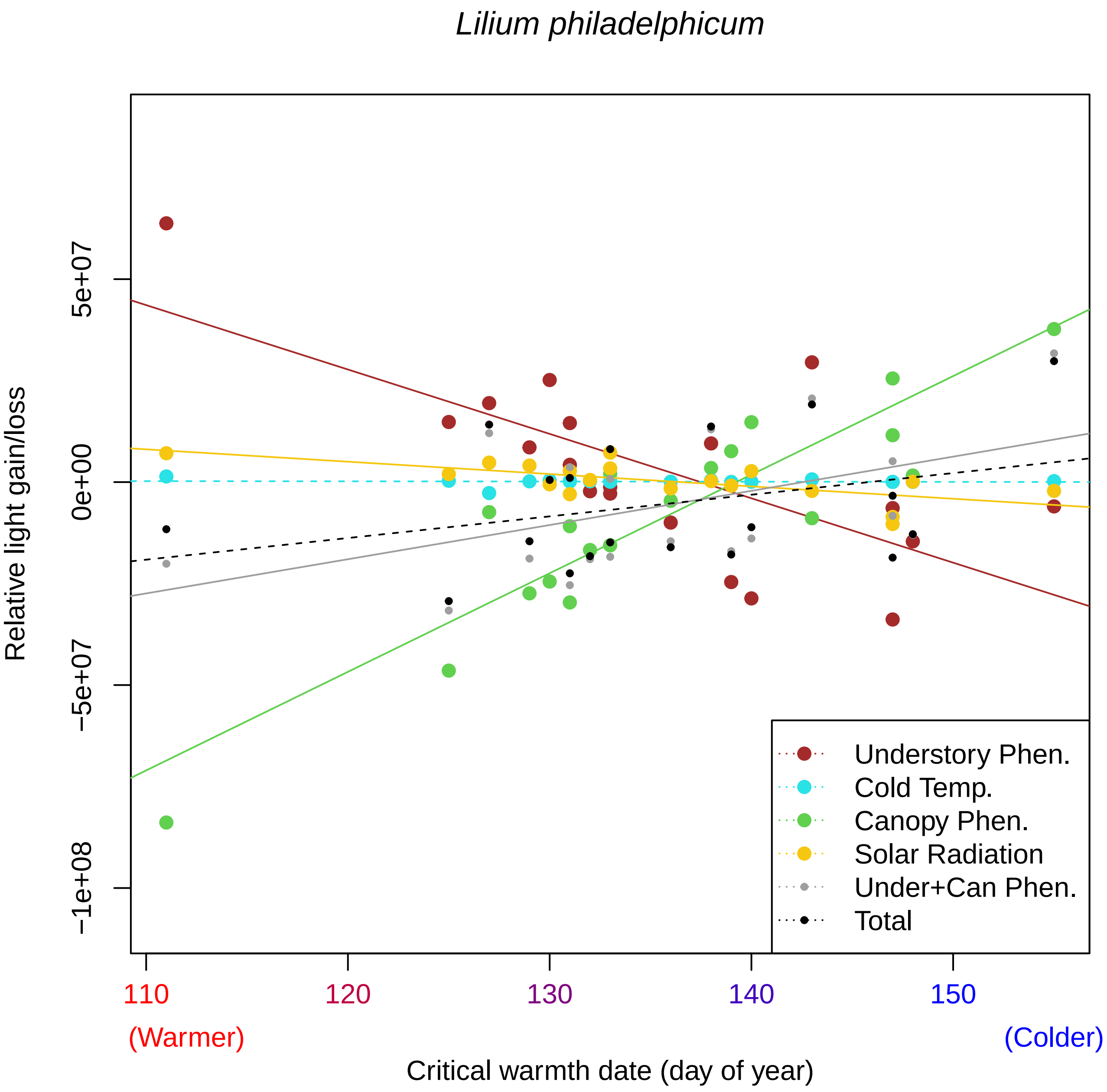

Lilium philadelphicum
Relative light gain/loss
5e+07
0e+00
−5e+07
−1e+08
110
120
130
140
150
(Warmer)
(Colder)
Critical warmth date (day of year)
Understory Phen.
Cold Temp.
Canopy Phen.
Solar Radiation
Under+Can Phen.
Total

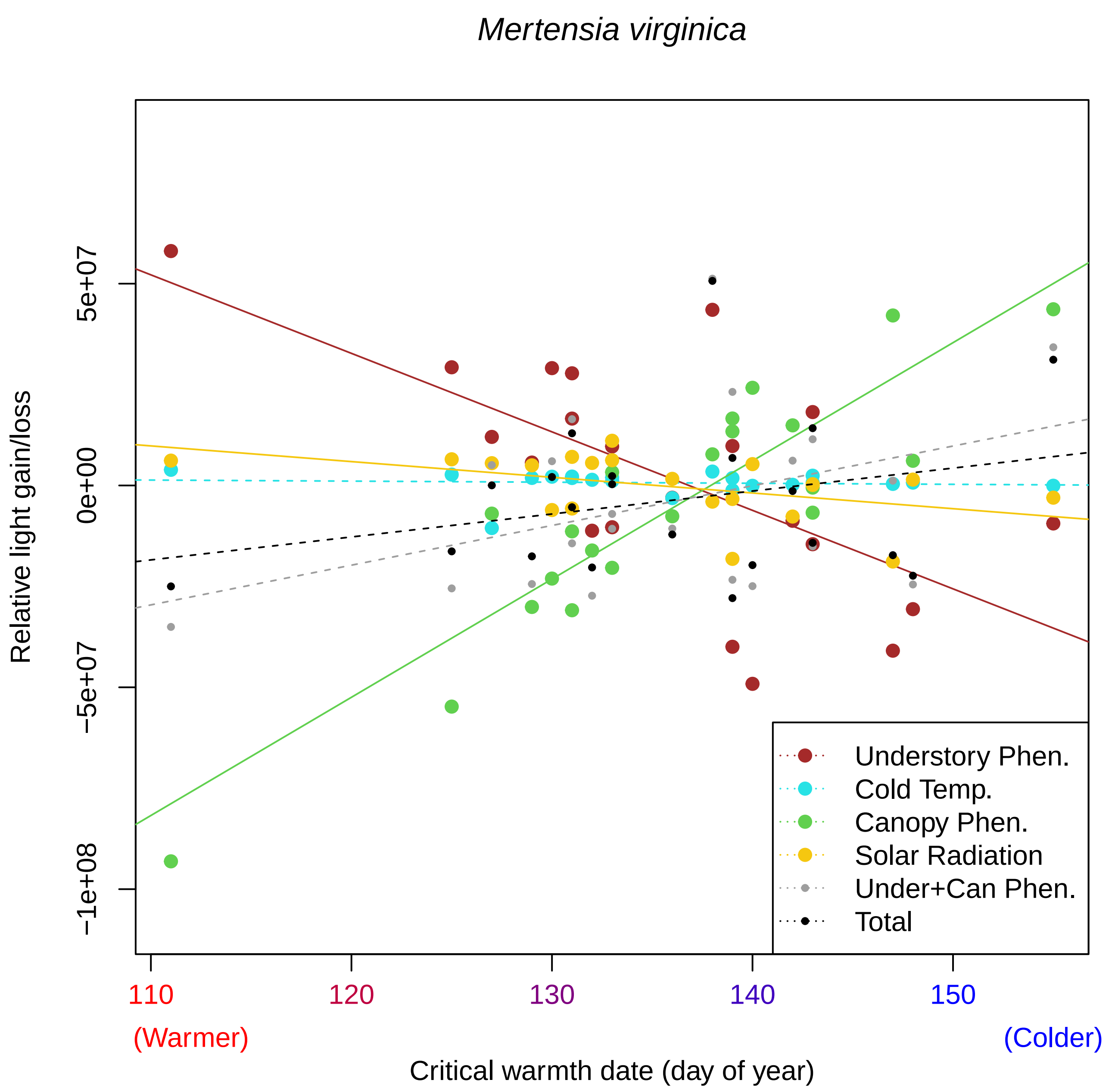

Mertensia virginica
Relative light gain/loss
5e+07
0e+00
−5e+07
−1e+08
Understory Phen.
Cold Temp.
Canopy Phen.
Solar Radiation
Under+Can Phen.
Total
110
120
130
140
150
(Warmer)
(Colder)
Critical warmth date (day of year)

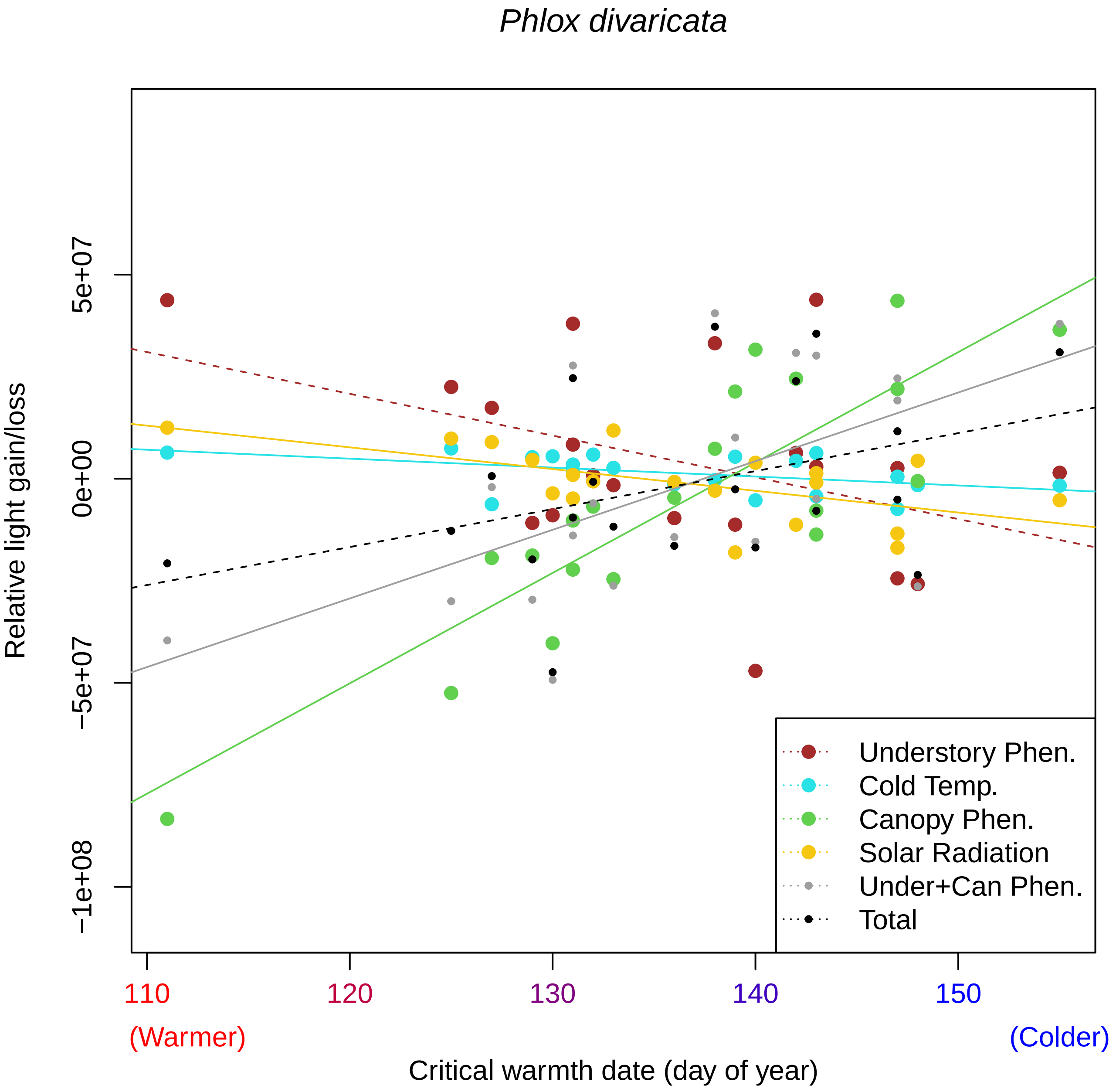

Phlox divaricata
5e+07
0e+00
−5e+07
−1e+08
Relative light gain/loss
110
120
130
140
150
(Warmer)
(Colder)
Critical warmth date (day of year)
Understory Phen.
Cold Temp.
Canopy Phen.
Solar Radiation
Under+Can Phen.
Total

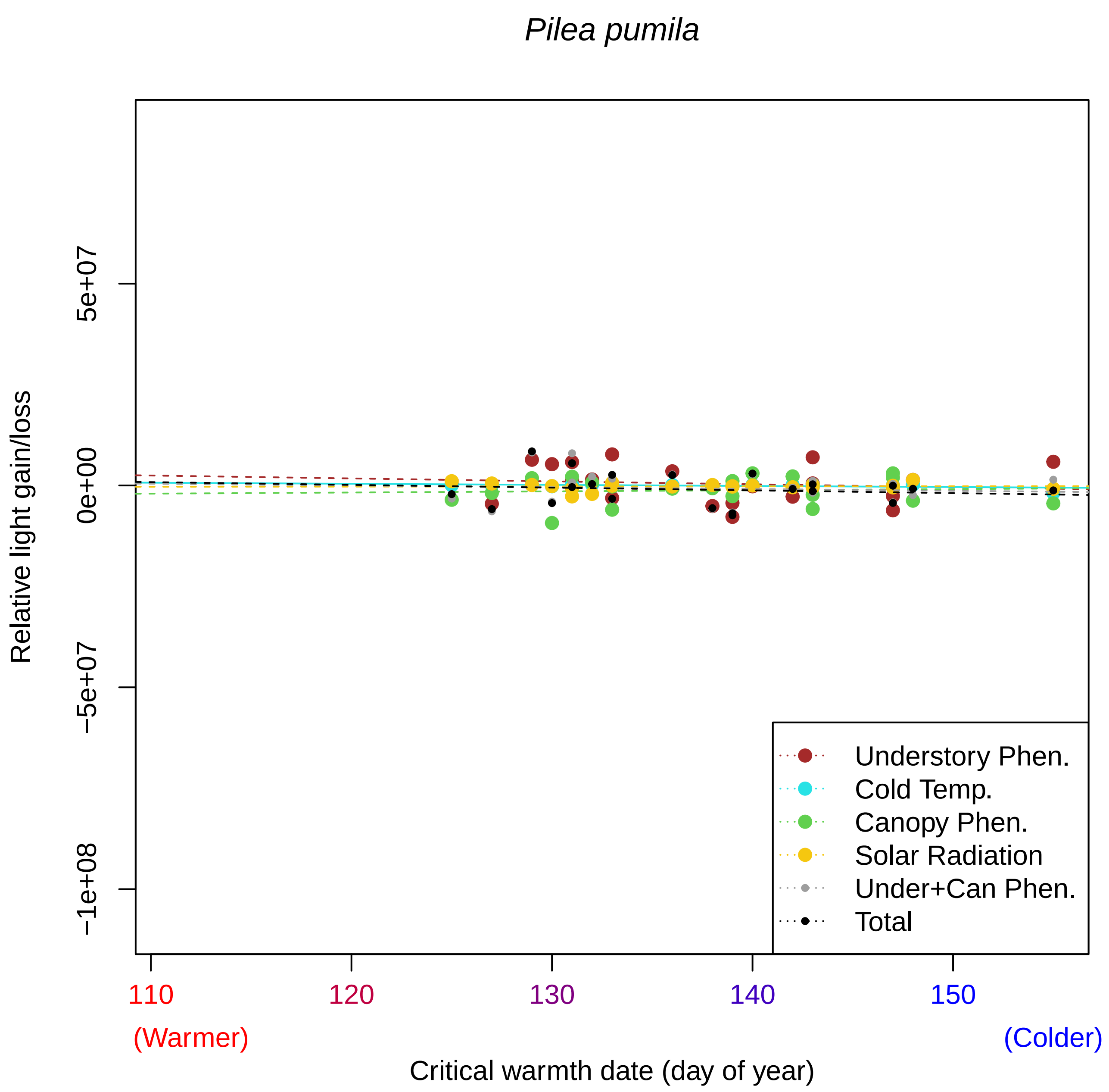

Pilea pumila
5e+07
0e+00
−5e+07
−1e+08
Relative light gain/loss
110
120
130
140
150
(Warmer)
(Colder)
Critical warmth date (day of year)
Understory Phen.
Cold Temp.
Canopy Phen.
Solar Radiation
Under+Can Phen.
Total

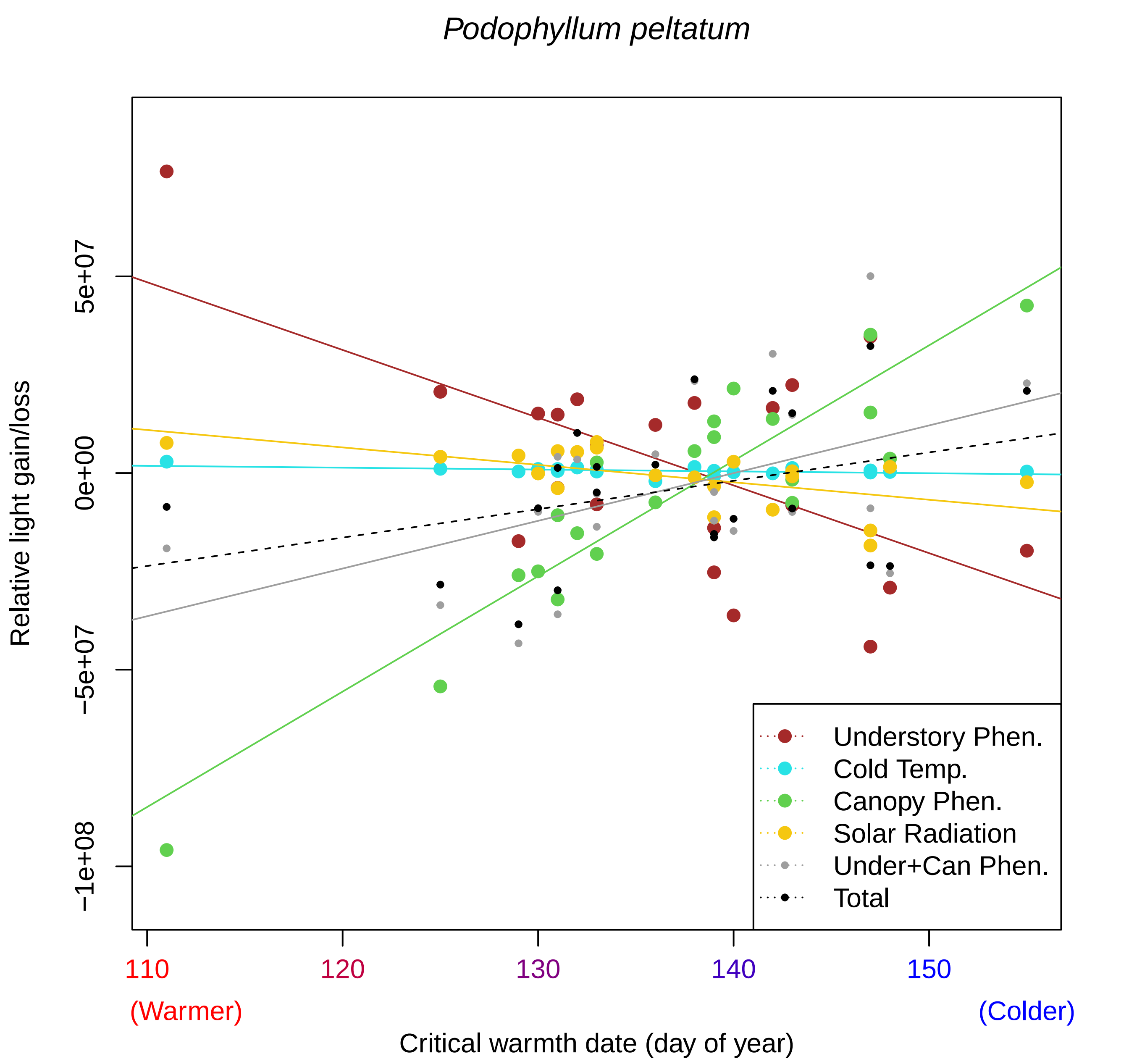

Podophyllum peltatum
Relative light gain/loss
5e+07
0e+00
−5e+07
−1e+08
110
120
130
140
150
(Warmer)
(Colder)
Critical warmth date (day of year)
Understory Phen.
Cold Temp.
Canopy Phen.
Solar Radiation
Under+Can Phen.
Total

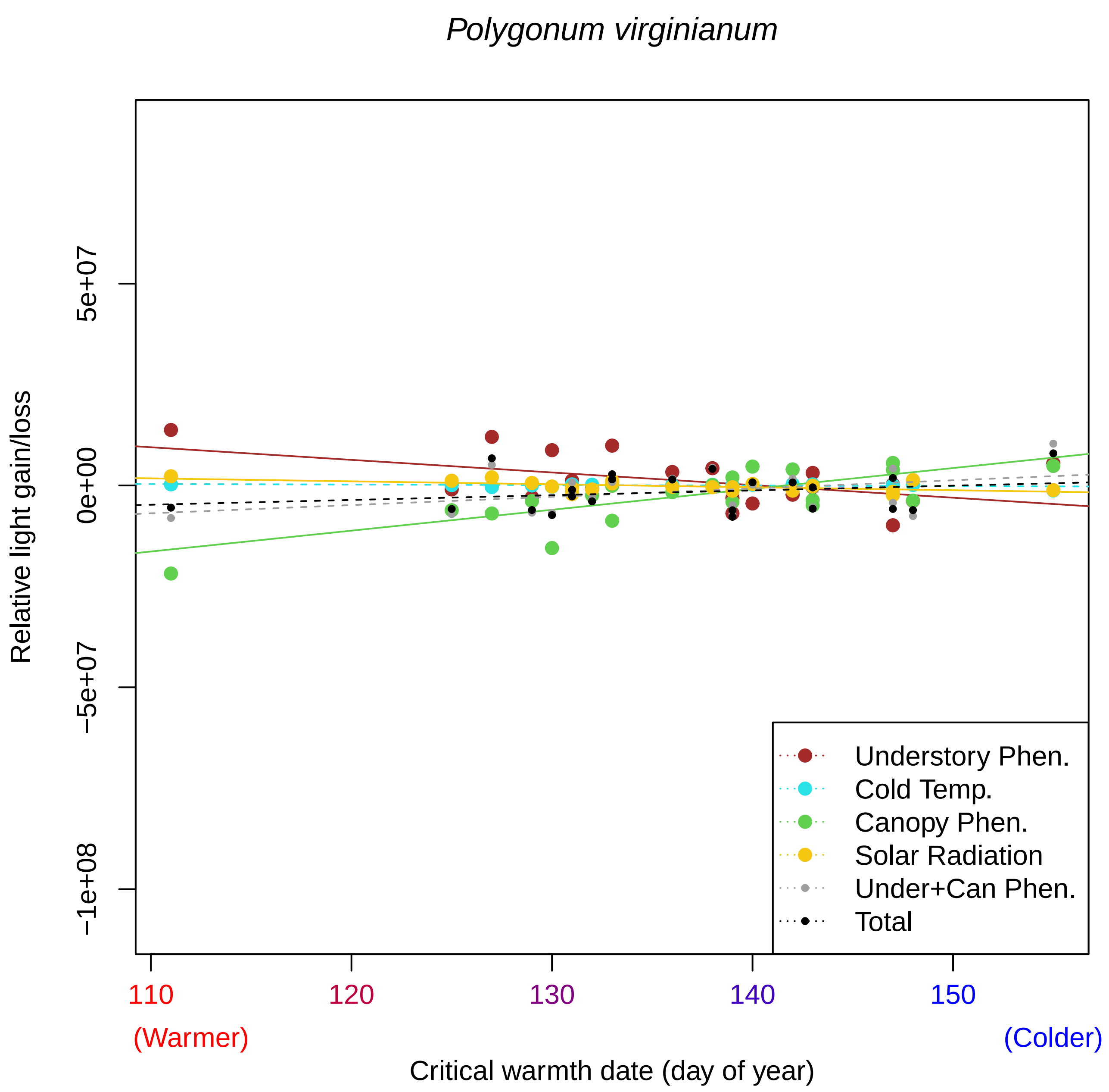

Polygonum virginianum
Relative light gain/loss
5e+07
0e+00
−5e+07
−1e+08
Understory Phen.
Cold Temp.
Canopy Phen.
Solar Radiation
Under+Can Phen.
Total
110
120
130
140
150
(Warmer)
(Colder)
Critical warmth date (day of year)

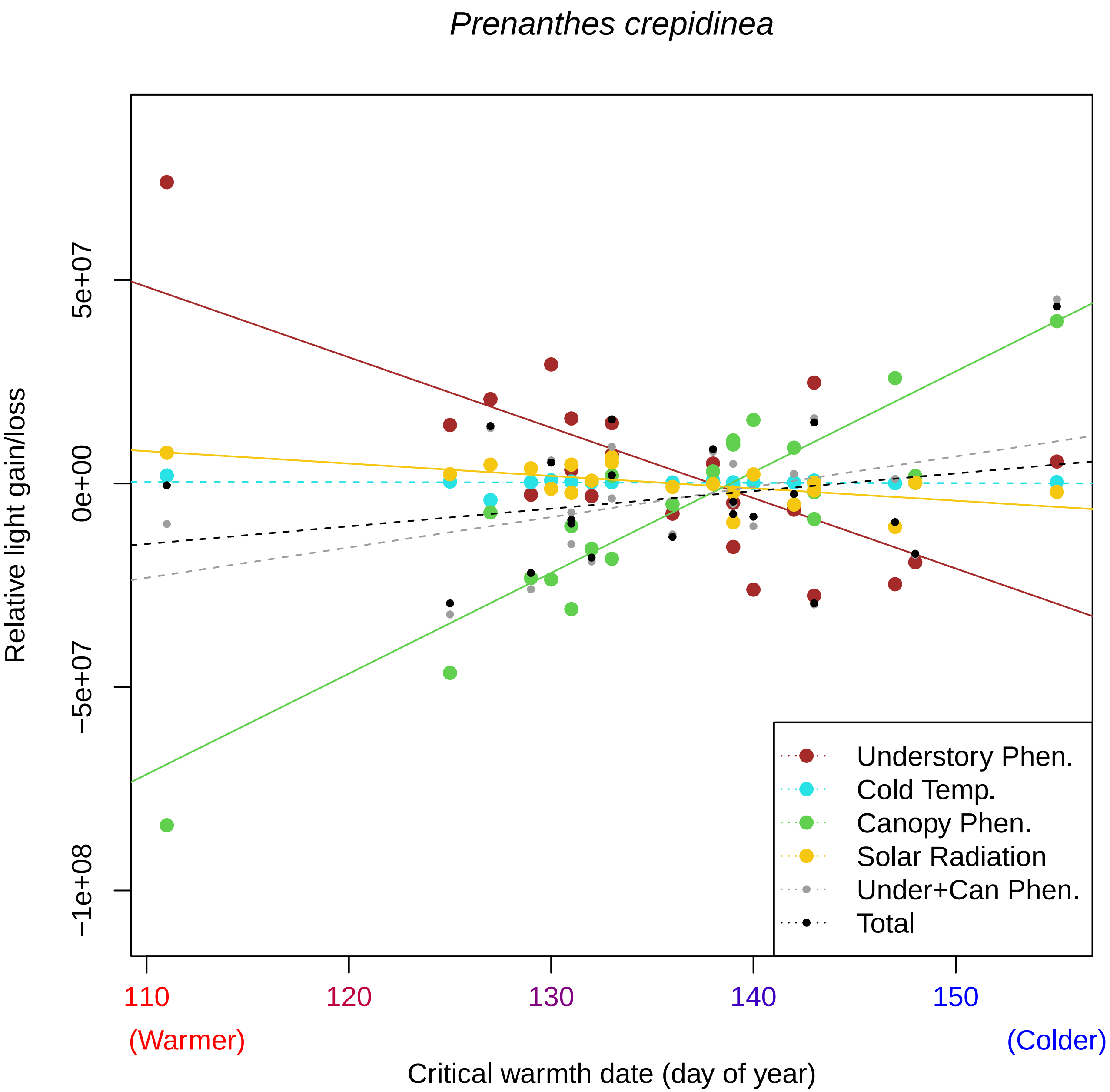

Prenanthes crepidinea
Relative light gain/loss
5e+07
0e+00
−5e+07
−1e+08
110
120
130
140
150
(Warmer)
(Colder)
Critical warmth date (day of year)
Understory Phen.
Cold Temp.
Canopy Phen.
Solar Radiation
Under+Can Phen.
Total

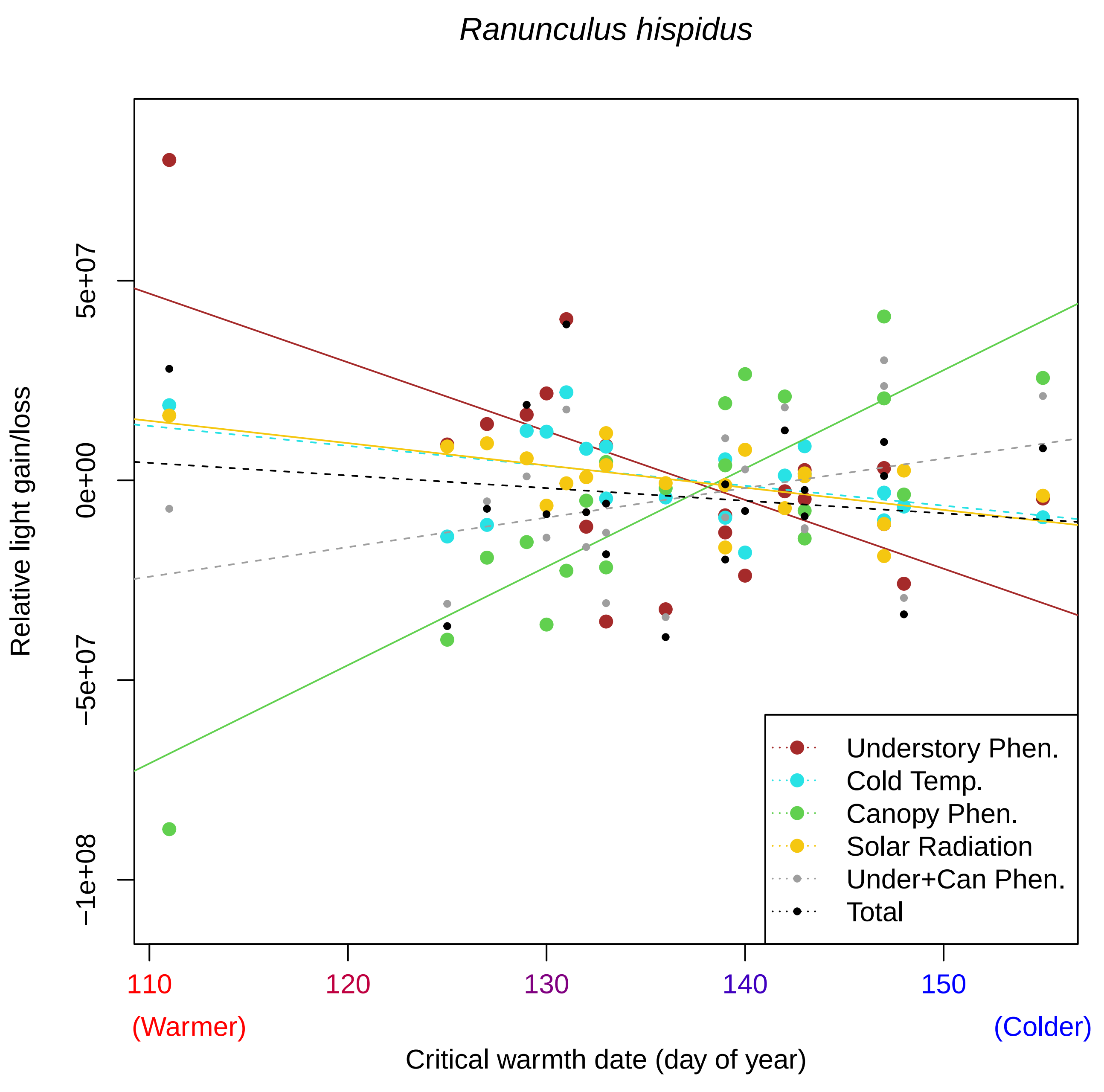

Ranunculus hispidus
Relative light gain/loss
5e+07
0e+00
−5e+07
−1e+08
110
120
130
140
150
(Warmer)
(Colder)
Critical warmth date (day of year)
Understory Phen.
Cold Temp.
Canopy Phen.
Solar Radiation
Under+Can Phen.
Total

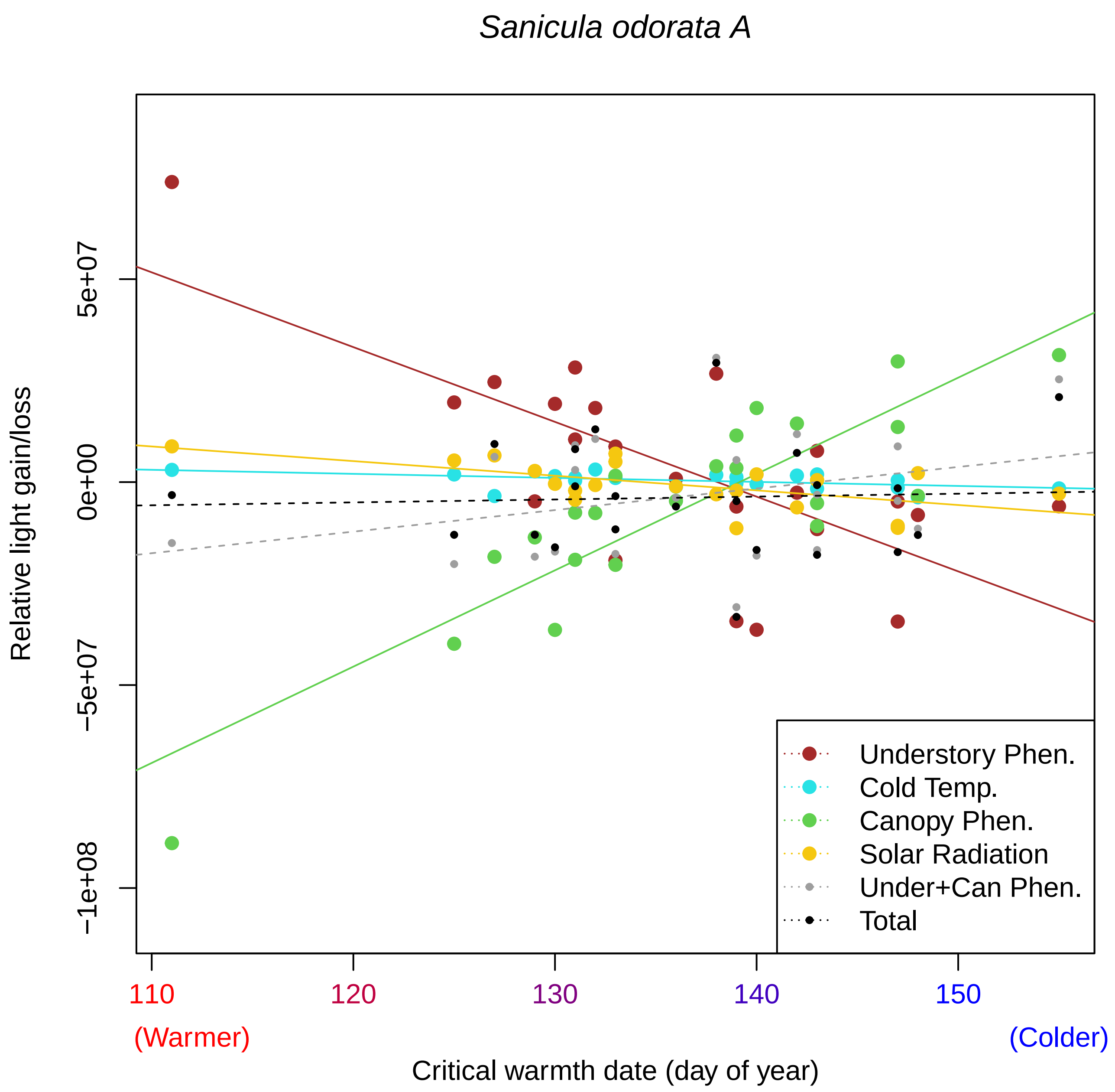

Sanicula odorata A
Relative light gain/loss
5e+07
0e+00
−5e+07
−1e+08
110
120
130
140
150
(Warmer)
(Colder)
Critical warmth date (day of year)
Understory Phen.
Cold Temp.
Canopy Phen.
Solar Radiation
Under+Can Phen.
Total

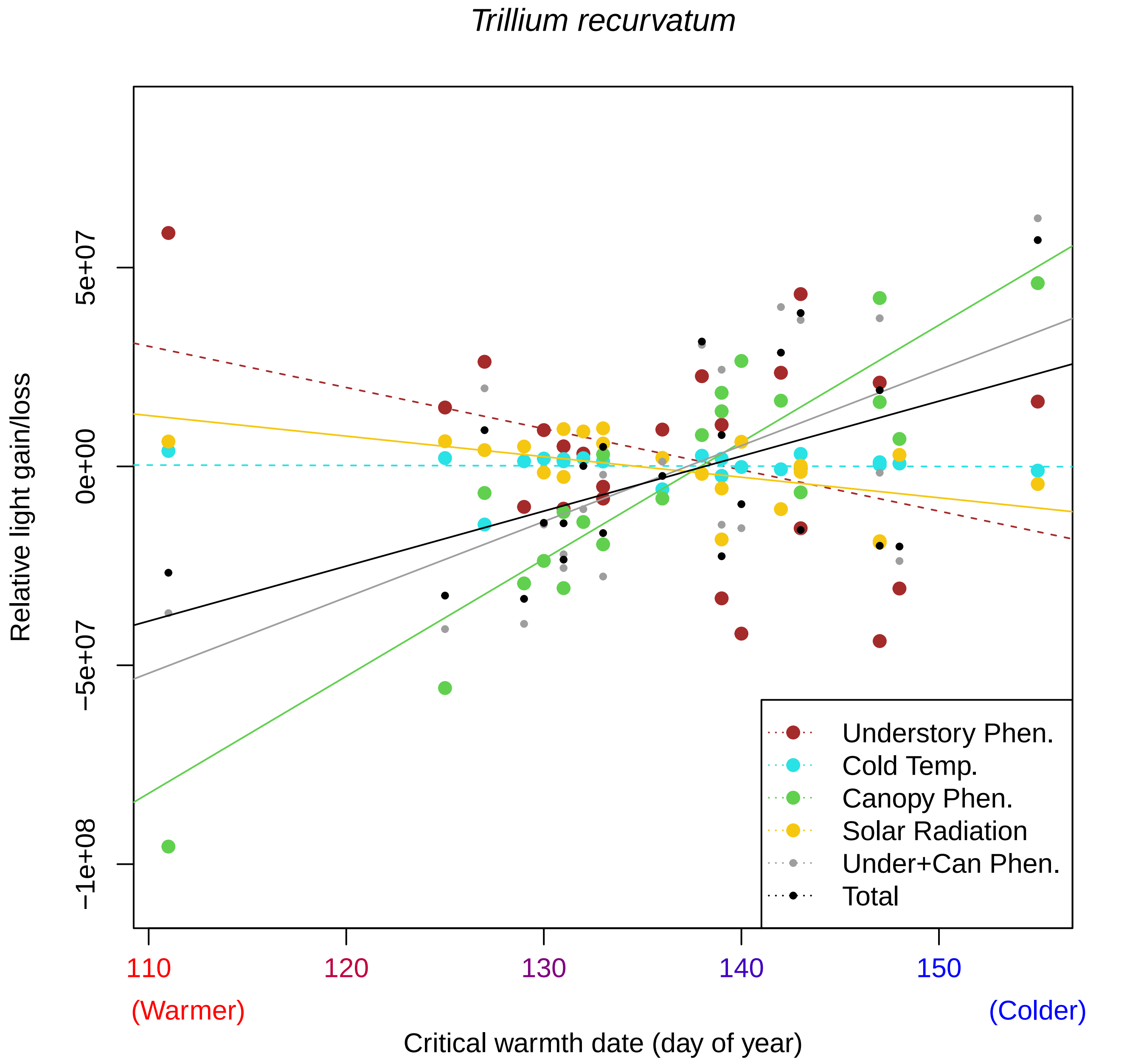

Trillium recurvatum
Relative light gain/loss
5e+07
0e+00
−5e+07
−1e+08
110
120
130
140
150
(Warmer)
(Colder)
Critical warmth date (day of year)
Understory Phen.
Cold Temp.
Canopy Phen.
Solar Radiation
Under+Can Phen.
Total

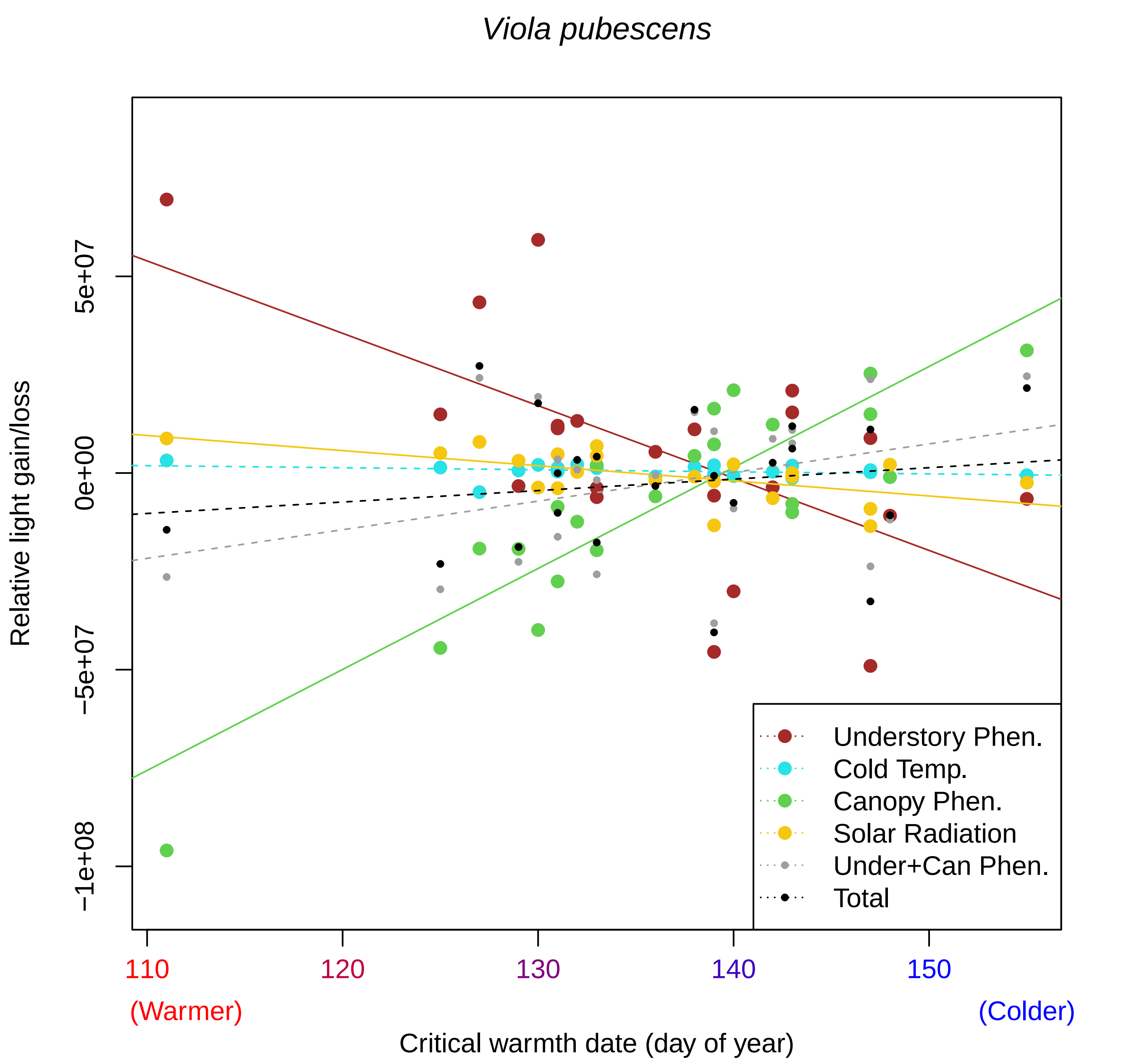

Viola pubescens
Relative light gain/loss
5e+07
0e+00
−5e+07
−1e+08
110
120
130
140
150
(Warmer)
(Colder)
Critical warmth date (day of year)
Understory Phen.
Cold Temp.
Canopy Phen.
Solar Radiation
Under+Can Phen.
Total

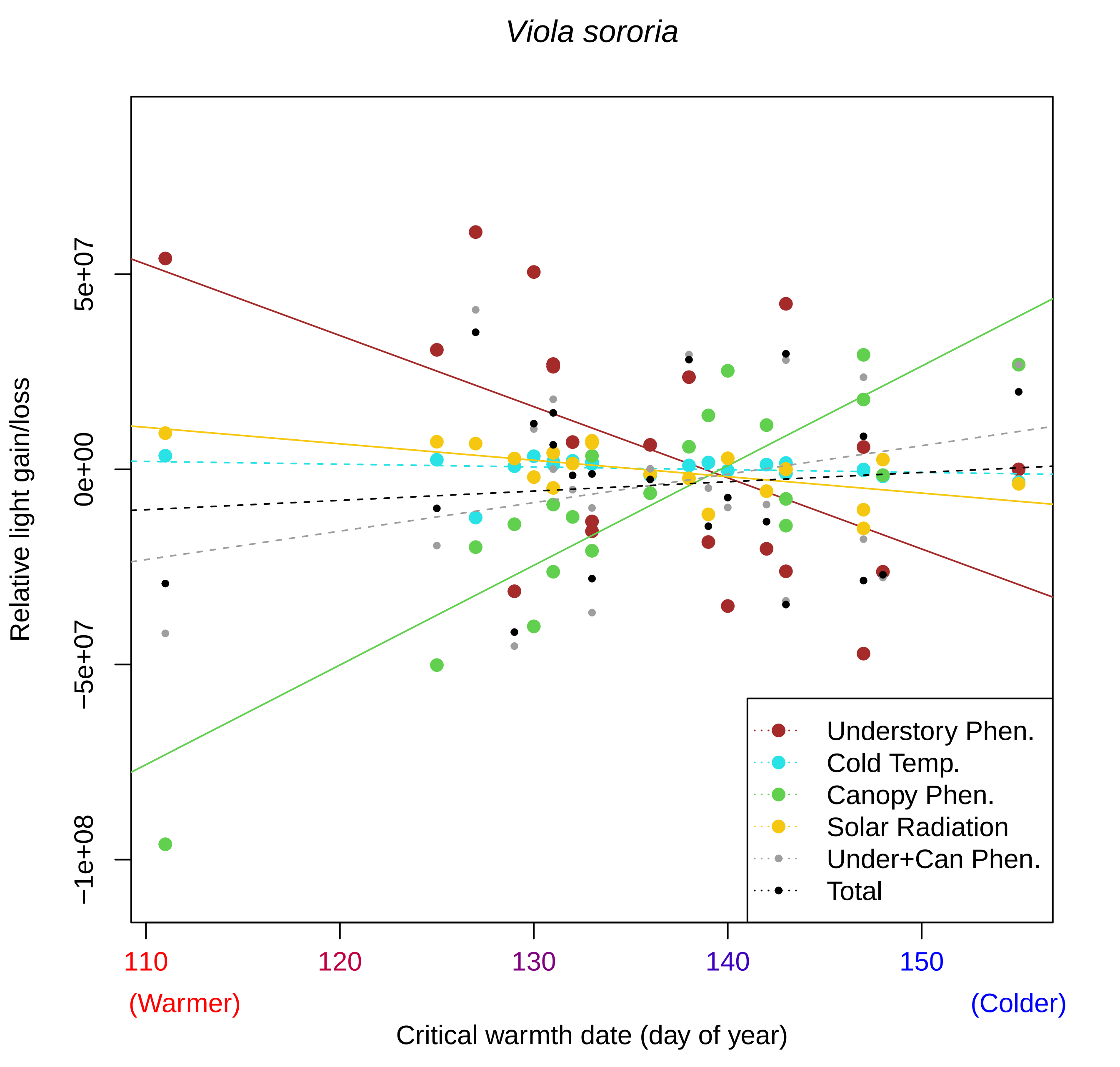

Viola sororia
Relative light gain/loss
5e+07
0e+00
−5e+07
−1e+08
110
120
130
140
150
(Warmer)
(Colder)
Critical warmth date (day of year)
Understory Phen.
Cold Temp.
Canopy Phen.
Solar Radiation
Under+Can Phen.
Total
