# Supplementary material for: Understory plants evade shading in a temperate deciduous forest amid climate variability by shifting phenology in synchrony with canopy trees
Source: PLoS One. 2024 Jun 26;19(6):e0306023. doi: 10.1371/journal.pone.0306023 (PMC11207122; doi:10.1371/journal.pone.0306023)
Supplement: S7 Fig — The first page shows an example year (2004) of calculated daily gross photosynthesis, with canopy light transmittance indicated in the background in gray. The remaining panels show these daily values summed for each year, and how that value has changed over time. Solid lines indicate a statistically significant (p < .05) difference of its estimated slope from 0, while dashed lines indicate that this standard was not met. (PDF) [file pone.0306023.s013.pdf]

Supporting Information 13 for Augspurger CK, Salk CF. Understory plants reduce light loss in a temperate deciduous forest amid climate variability by shifting phenology in synchrony with canopy trees. PLoS One. In review.

Supporting Information 13. Trends in gross annual photosynthesis by herb species by year. The first page shows an example year (2004) of calculated daily gross photosynthesis, with canopy light transmittance indicated in the background in gray. The remaining panels show these daily values summed for each year, and how that value has changed over time. Solid lines indicate a statistically significant ($p<.05$) difference of its estimated slope from 0, while dashed lines indicate that this standard was not met.

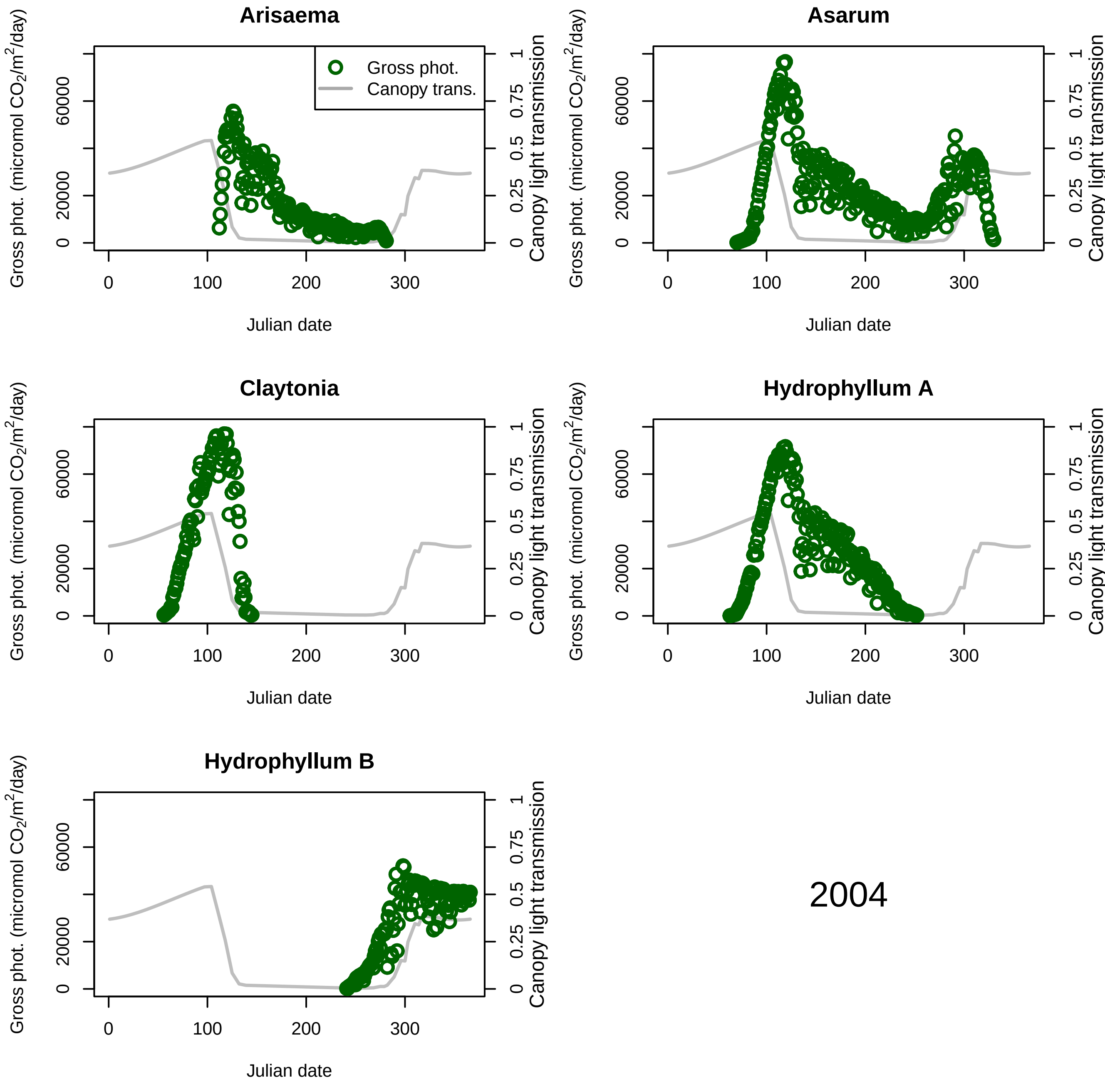
Arisaema
Gross phot.
Canopy trans.
Asarum
Claytonia
Hydrophyllum A
Hydrophyllum B
Gross phot. (micromol CO2/m2/day)
Canopy light transmission
Julian date
0
20000
60000
0.25
0.5
0.75
1
100
200
300
2004

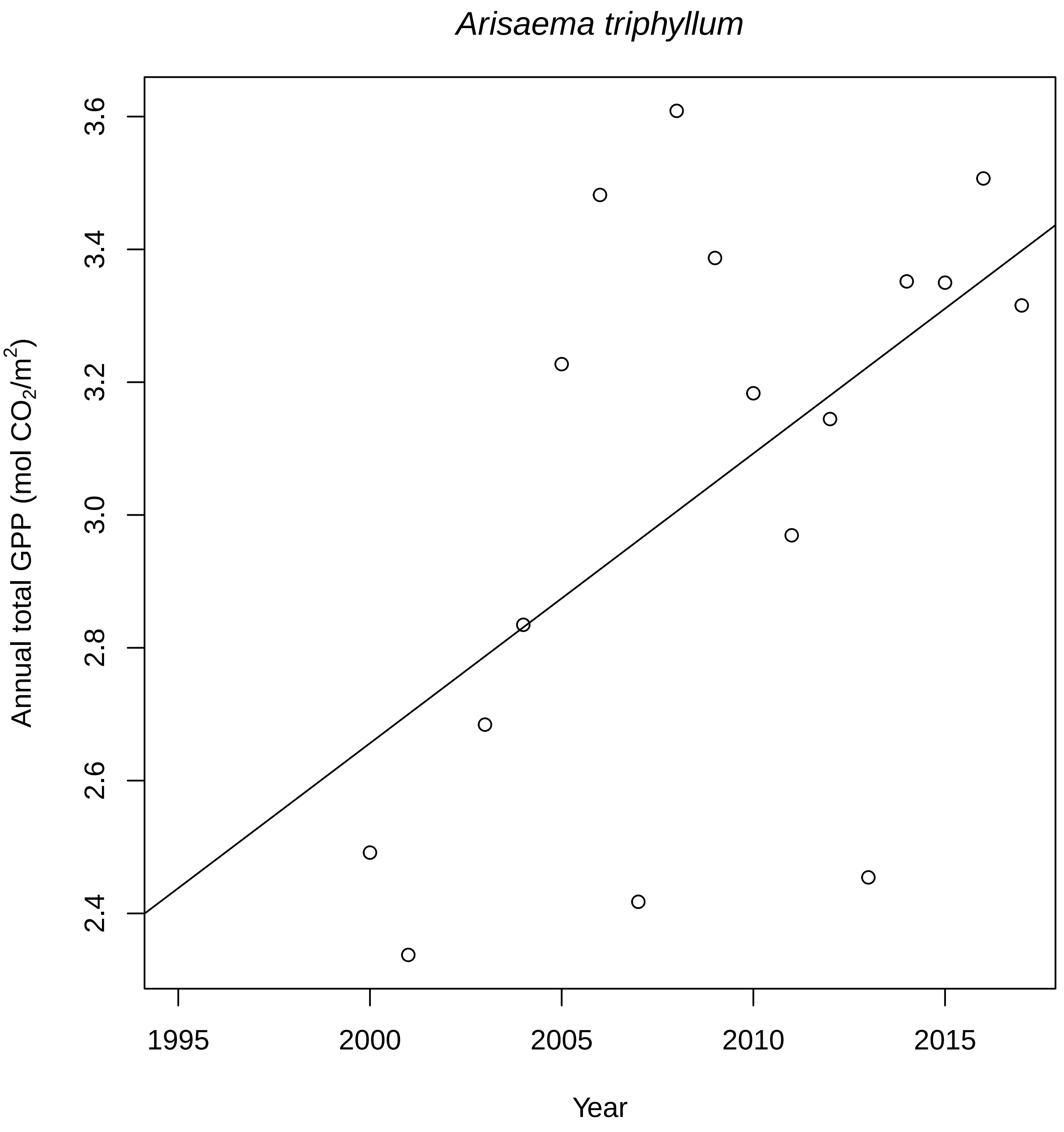

Arisaema triphyllum
Annual total GPP (mol CO_2/m^2)
3.6
3.4
3.2
3.0
2.8
2.6
2.4
1995
2000
2005
2010
2015
Year

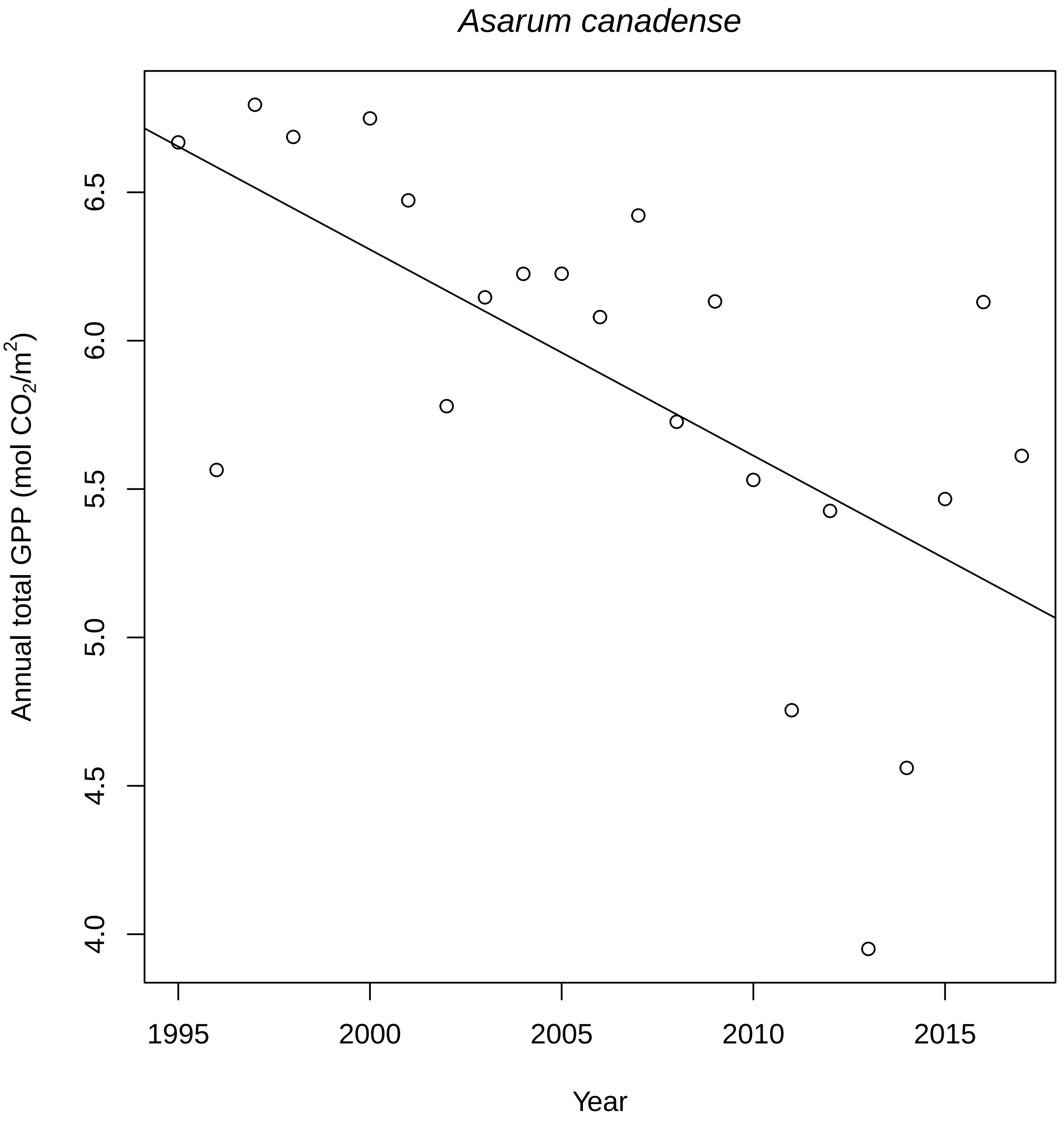

Asarum canadense
Annual total GPP (mol CO_2/m^2)
6.5
6.0
5.5
5.0
4.5
4.0
1995
2000
2005
2010
2015
Year

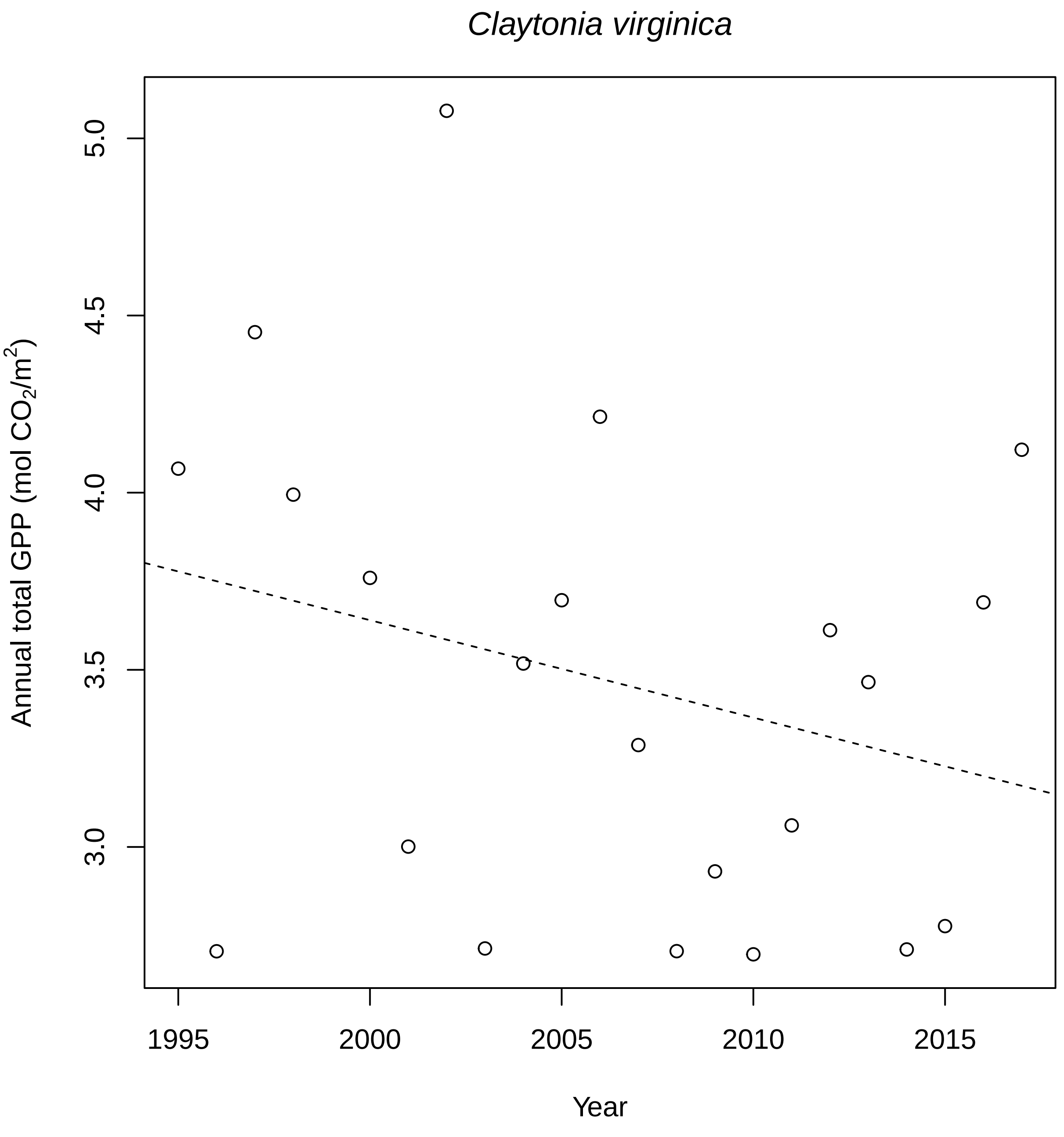
Claytonia virginica
Annual total GPP (mol CO_2/m^2)
5.0
4.5
4.0
3.5
3.0
1995
2000
2005
2010
2015
Year

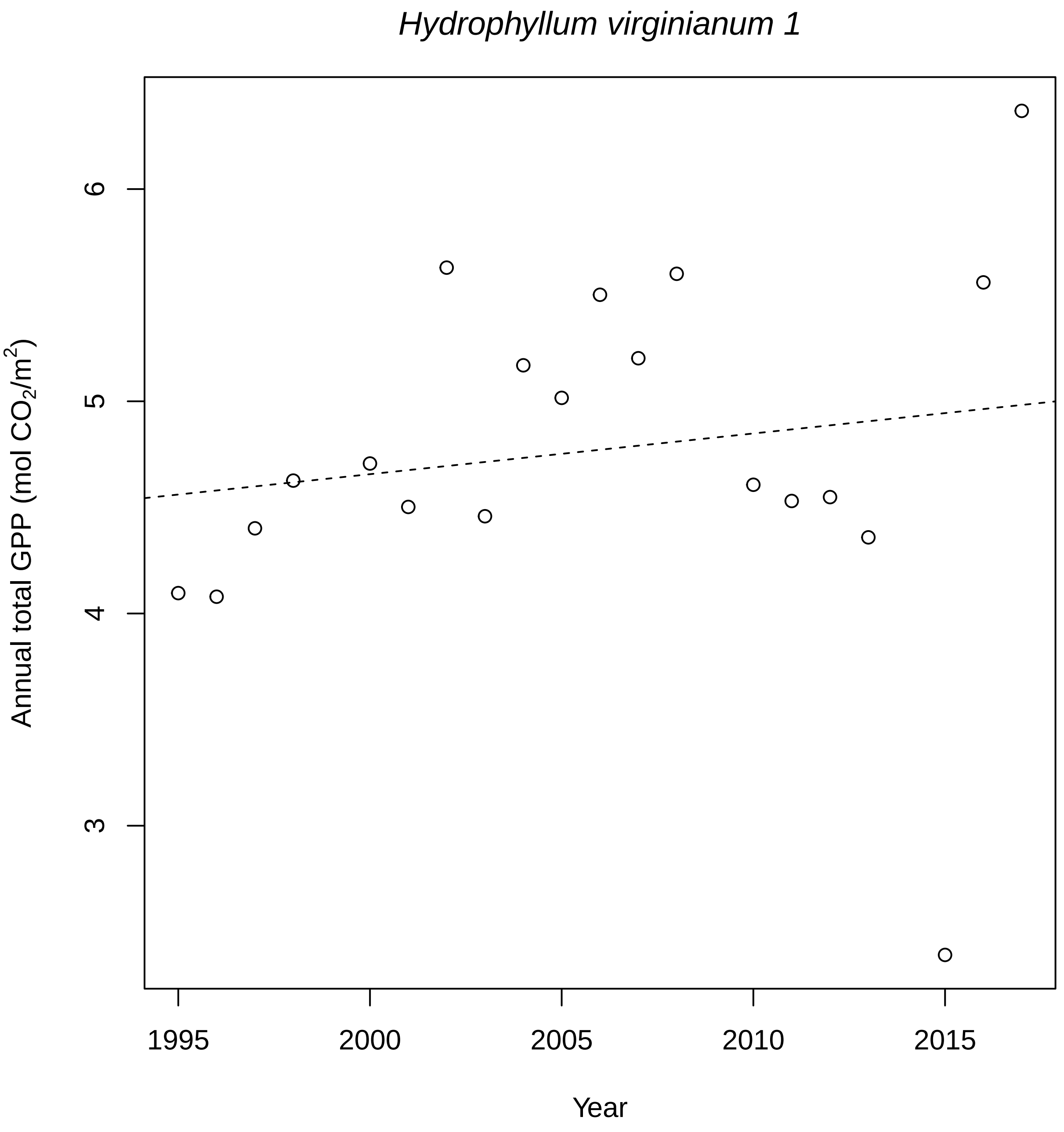

Hydrophyllum virginianum 1
Annual total GPP (mol CO_2/m^2)
3
4
5
6
1995
2000
2005
2010
2015
Year

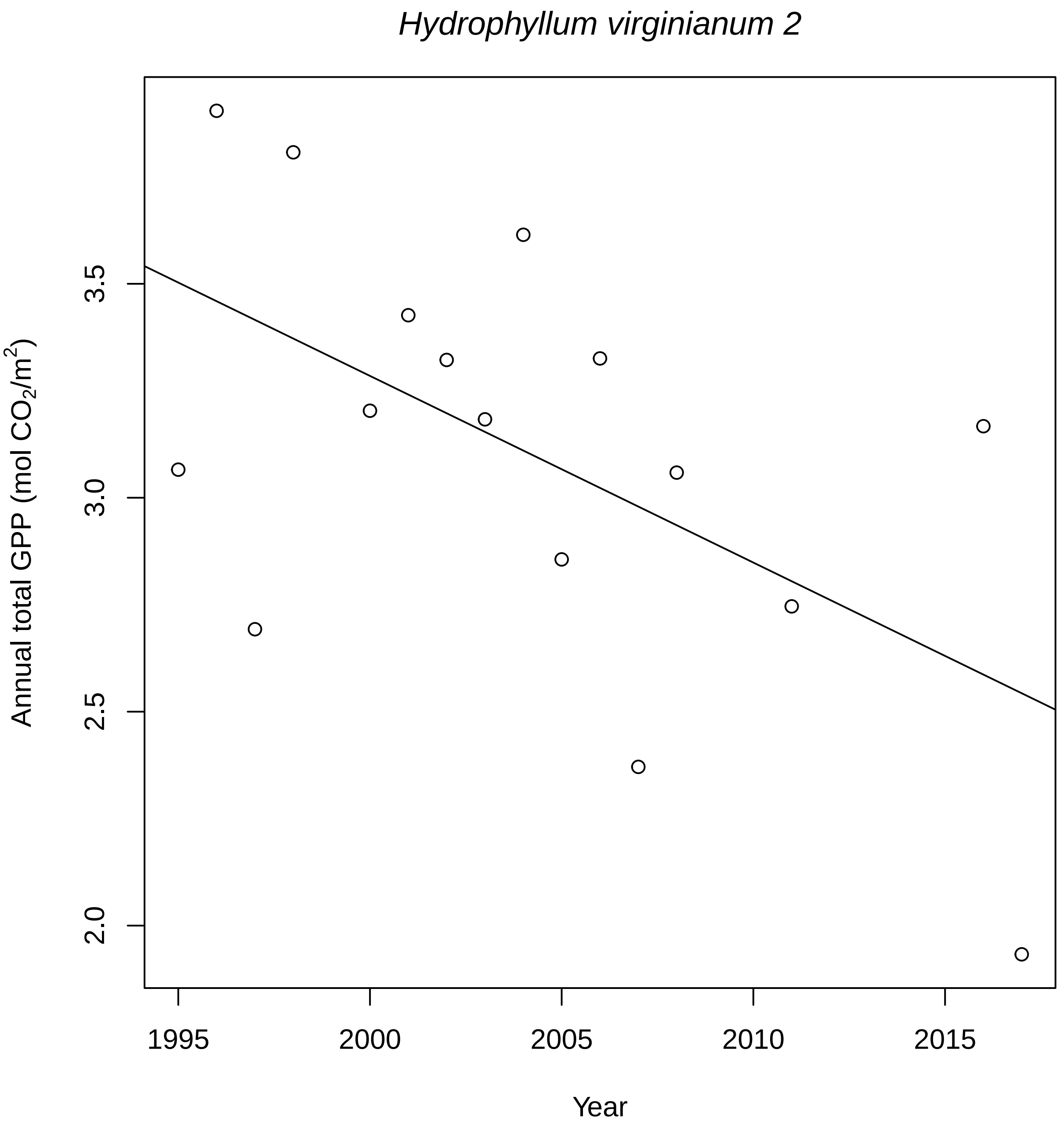
Hydrophyllum virginianum 2
Annual total GPP (mol CO_2/m^2)
3.5
3.0
2.5
2.0
1995
2000
2005
2010
2015
Year
