# Supplementary material for: Understory plants evade shading in a temperate deciduous forest amid climate variability by shifting phenology in synchrony with canopy trees
Source: PLoS One. 2024 Jun 26;19(6):e0306023. doi: 10.1371/journal.pone.0306023 (PMC11207122; doi:10.1371/journal.pone.0306023)
Supplement: S8 Fig — This integrative measure of spring temperature means that warmer springs fall to the left on the x-axis. Solid lines indicate a statistically-significant (p < .05) difference of the estimated slope from 0, while dashed lines indicate that this standard was not met. (PDF) [file pone.0306023.s014.pdf]

Supporting Information 14 for Augspurger CK, Salk CF. Understory plants reduce light loss in a temperate deciduous forest amid climate variability by shifting phenology in synchrony with canopy trees. PLoS One. In review.

Supporting Information 14. Trends in herb species' gross photosynthesis as a function of date on which the 48−day running average temperature first exceeded 13° C (see Methods: Section 6). This integrative measure of spring temperature means that warmer springs fall to the left on the x−axis. Solid lines indicate a statistically−significant ($p<.05$) difference of the estimated slope from 0, while dashed lines indicate that this standard was not met.

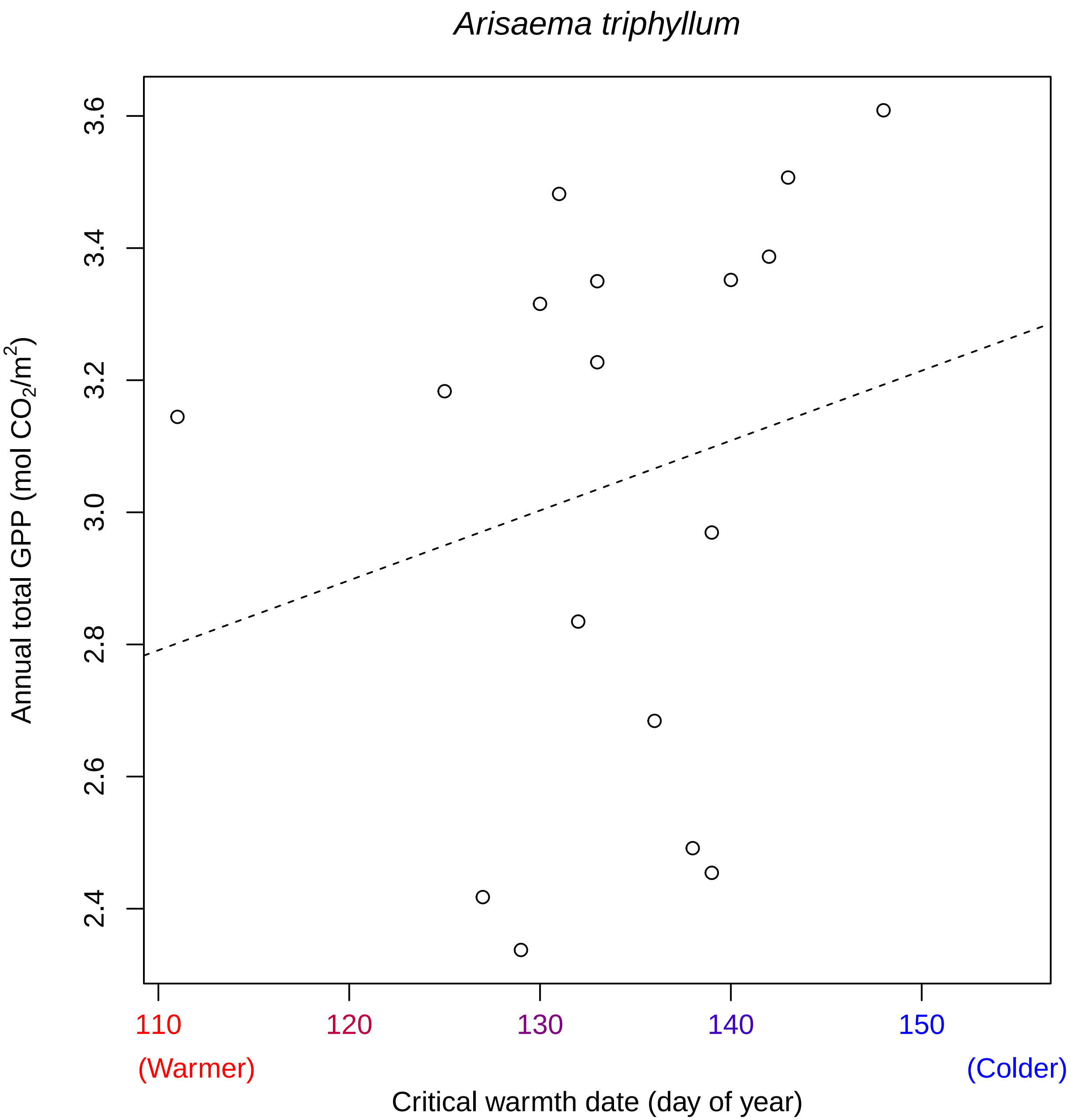

Arisaema triphyllum
Annual total GPP (mol CO_2/m^2)
3.6
3.4
3.2
3.0
2.8
2.6
2.4
110
120
130
140
150
(Warmer)
(Colder)
Critical warmth date (day of year)

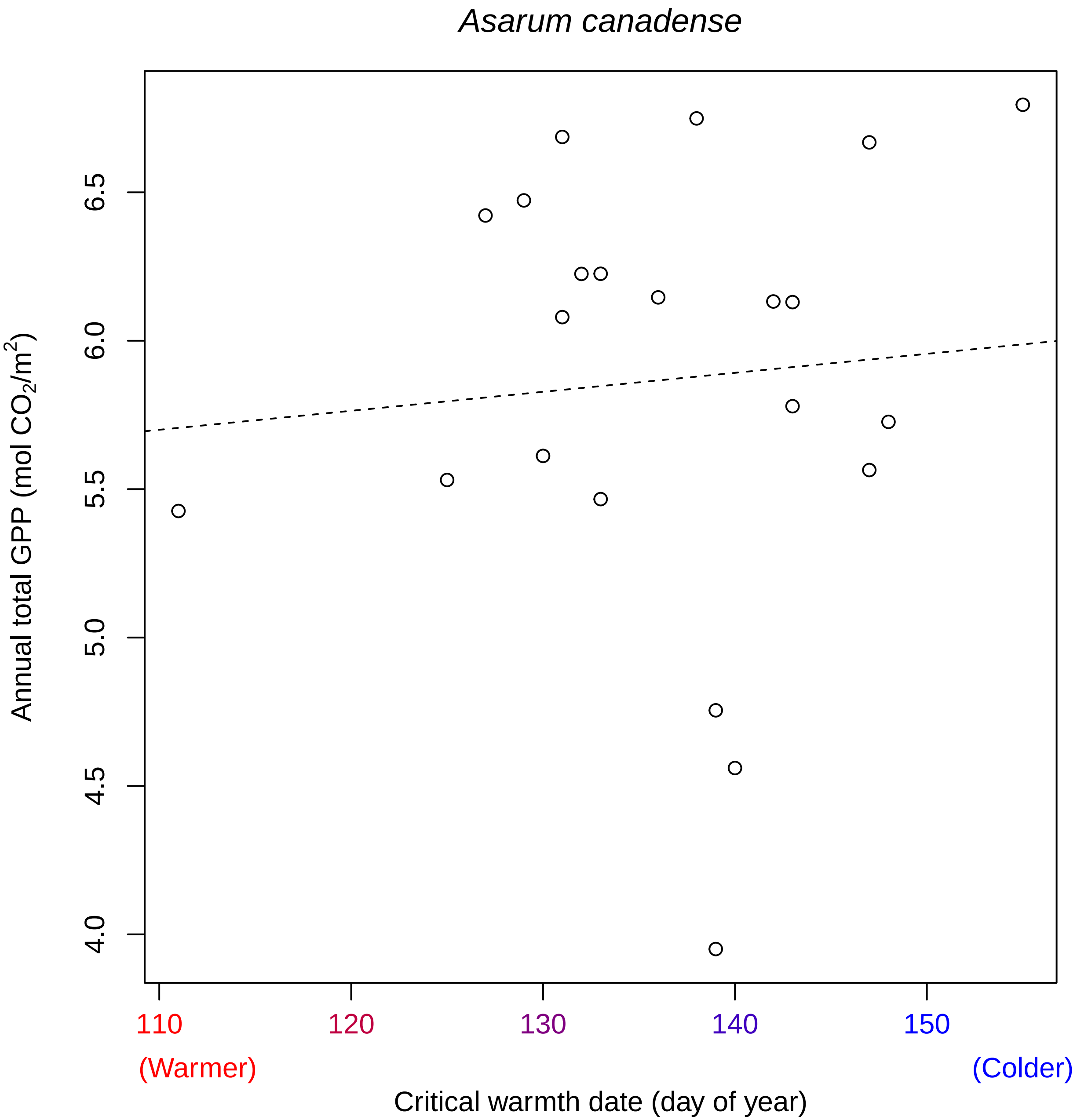

Asarum canadense
Annual total GPP (mol CO2/m2)
6.5
6.0
5.5
5.0
4.5
4.0
110
120
130
140
150
(Warmer)
(Colder)
Critical warmth date (day of year)

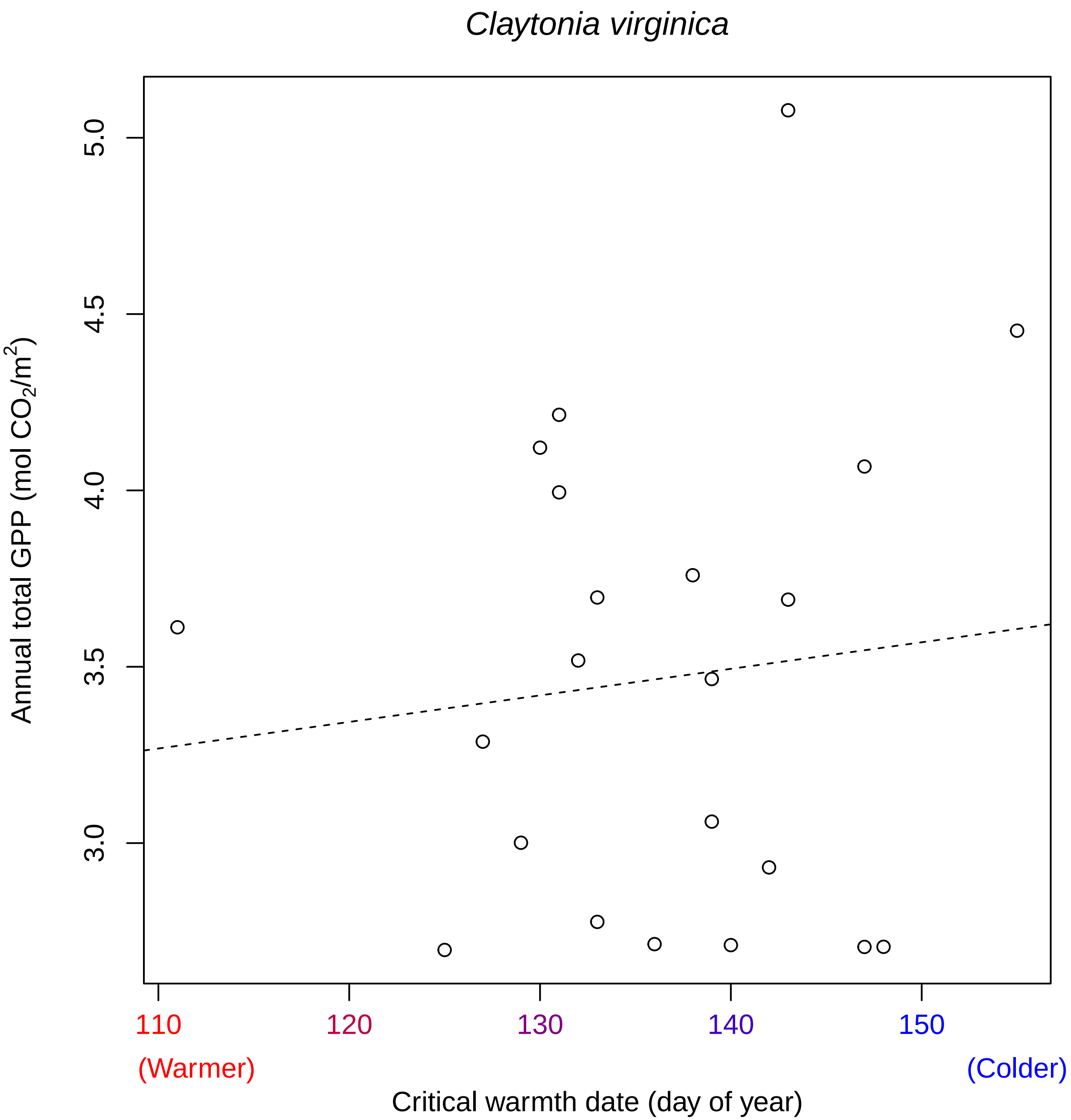
Claytonia virginica
Annual total GPP (mol CO2/m2)
5.0
4.5
4.0
3.5
3.0
110
120
130
140
150
(Warmer)
(Colder)
Critical warmth date (day of year)

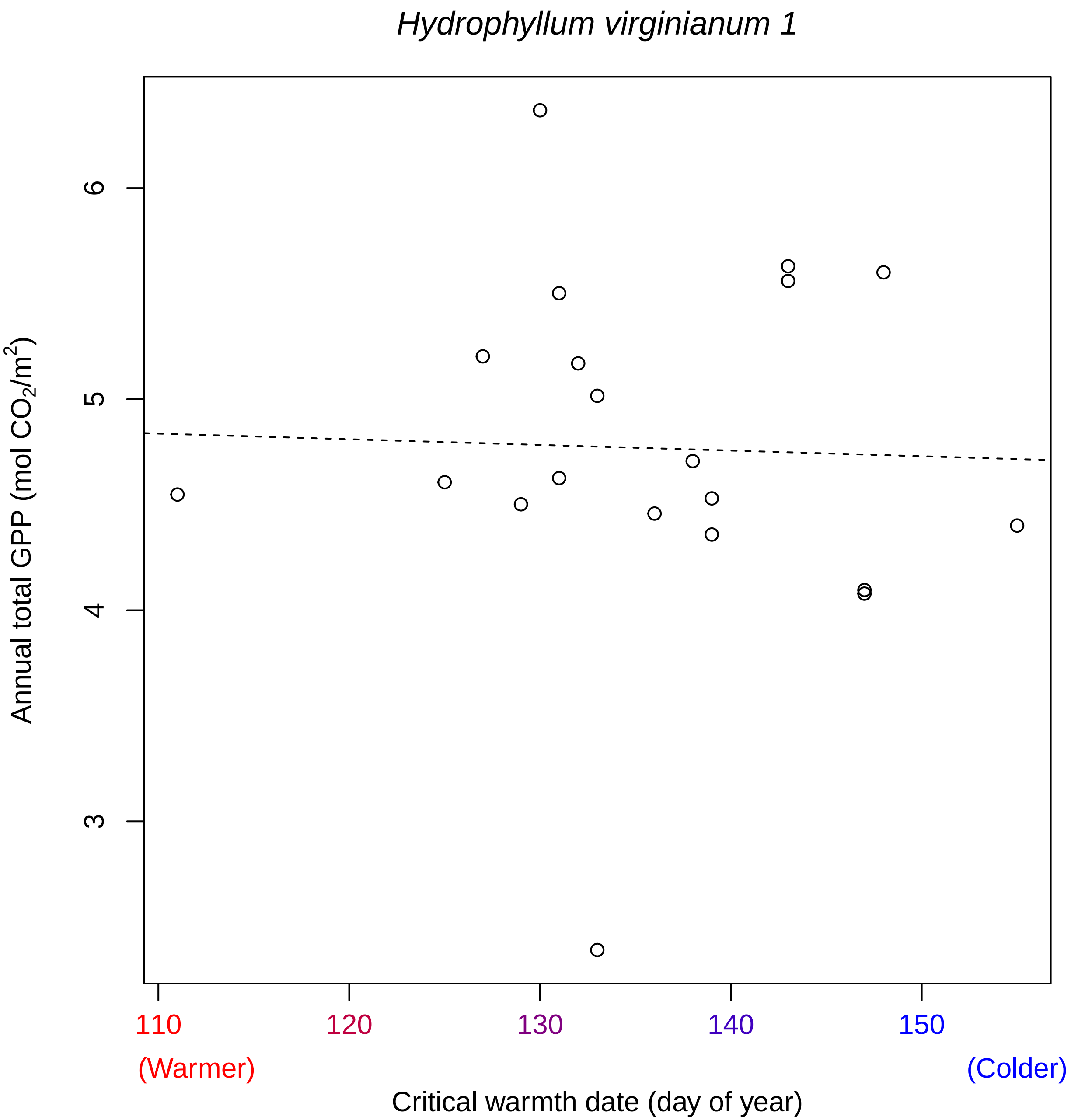

Hydrophyllum virginianum 1
Annual total GPP (mol CO2/m2)
6
5
4
3
110
120
130
140
150
(Warmer)
(Colder)
Critical warmth date (day of year)

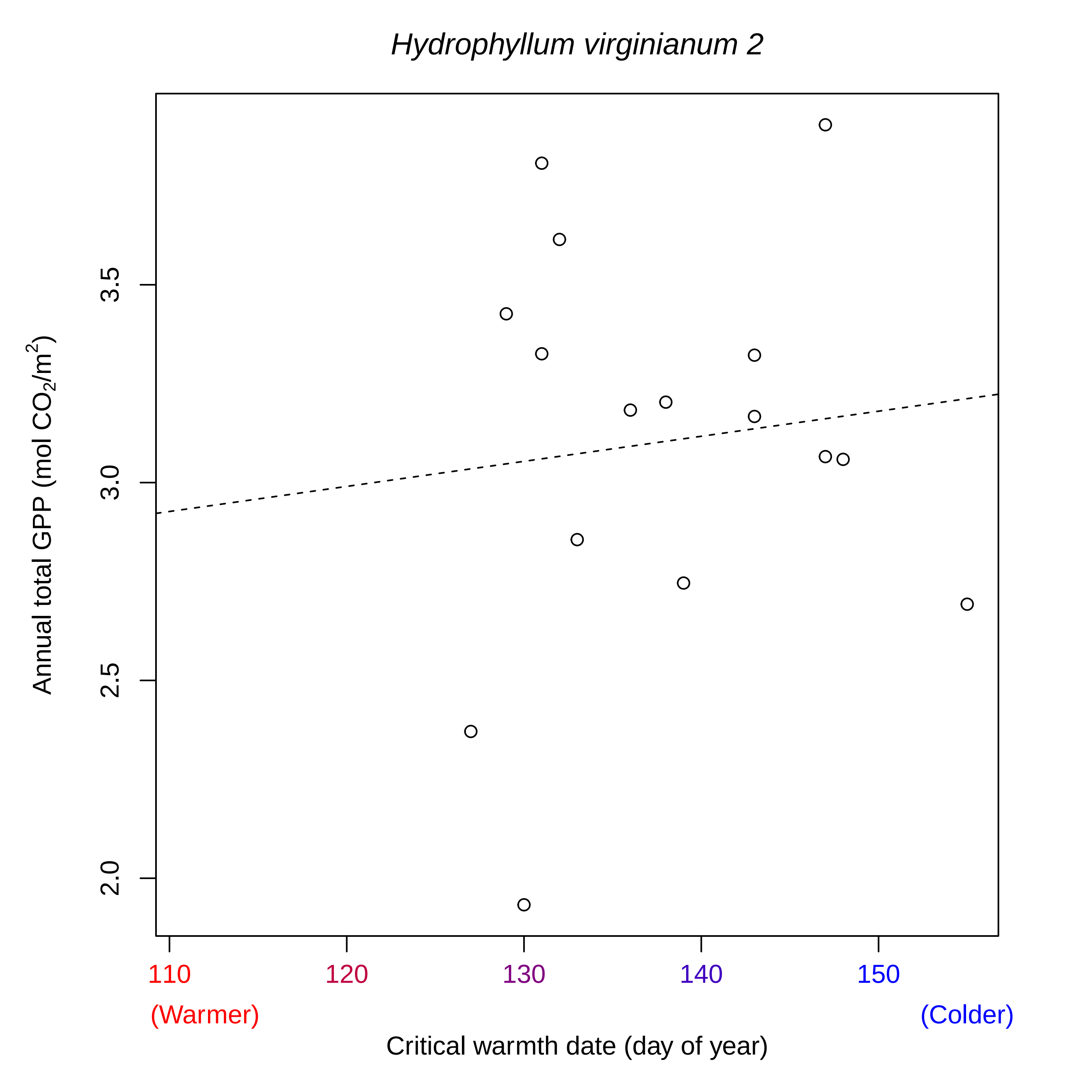
Hydrophyllum virginianum 2
Annual total GPP (mol CO_2/m^2)
2.0
2.5
3.0
3.5
110
120
130
140
150
(Warmer)
(Colder)
Critical warmth date (day of year)
