# Supplementary material for: Understory plants evade shading in a temperate deciduous forest amid climate variability by shifting phenology in synchrony with canopy trees
Source: PLoS One. 2024 Jun 26;19(6):e0306023. doi: 10.1371/journal.pone.0306023 (PMC11207122; doi:10.1371/journal.pone.0306023)
Supplement: Attachment — Submitted filename: Responses to reviews late April.docx [file pone.0306023.s015.docx]

| **Who** | **Comment** | **Response** |
| --- | --- | --- |
| Editor | 1. Please ensure that your manuscript meets PLOS ONE's style requirements, including those for file naming. The PLOS ONE style templates can be found at  https://journals.plos.org/plosone/s/file?id=wjVg/PLOSOne_formatting_sample_main_body.pdf and  https://journals.plos.org/plosone/s/file?id=ba62/PLOSOne_formatting_sample_title_authors_affiliations.pdf | We have revised the manuscript to comply with the PLoS One formatting guidelines. |
| Editor | 2. In your Methods section, please provide additional information regarding the permits you obtained for the work. Please ensure you have included the full name of the authority that approved the field site access and, if no permits were required, a brief statement explaining why. | Inserted in the methods section: “Field site access was given by Steve Buck, Natural Areas Manager, University of Illinois.” |
| Editor | 3. We suggest you thoroughly copyedit your manuscript for language usage, spelling, and grammar. If you do not know anyone who can help you do this, you may wish to consider employing a professional scientific editing service.  Whilst you may use any professional scientific editing service of your choice, PLOS has partnered with both American Journal Experts (AJE) and Editage to provide discounted services to PLOS authors. Both organizations have experience helping authors meet PLOS guidelines and can provide language editing, translation, manuscript formatting, and figure formatting to ensure your manuscript meets our submission guidelines. To take advantage of our partnership with AJE, visit the AJE website (http://learn.aje.com/plos/) for a 15% discount off AJE services. To take advantage of our partnership with Editage, visit the Editage website (www.editage.com) and enter referral code PLOSEDIT for a 15% discount off Editage services.  If the PLOS editorial team finds any language issues in text that either AJE or Editage has edited, the service provider will re-edit the text for free.  Upon resubmission, please provide the following:  The name of the colleague or the details of the professional service that edited your manuscript  A copy of your manuscript showing your changes by either highlighting them or using track changes (uploaded as a *supporting information* file  A clean copy of the edited manuscript (uploaded as the new *manuscript* file). | We are both native English speakers and have copyedited our manuscript thoroughly, and have now re-checked our edited version. |
| Editor | 4. We note that you have stated that you will provide repository information for your data at acceptance. Should your manuscript be accepted for publication, we will hold it until you provide the relevant accession numbers or DOIs necessary to access your data. If you wish to make changes to your Data Availability statement, please describe these changes in your cover letter and we will update your Data Availability statement to reflect the information you provide. | We have now deposited the data files and relevant metadata at Dryad: doi:10.5061/dryad.pg4f4qrww  The data files can be viewed by reviewers/editors at the following temporary URL:  <https://datadryad.org/stash/share/reTE446z3X7c9VQ8GObD6VGHQ5kLgE_ISoeGok7cxDI>  The above DOI will serve as a permanent link to the Dryad archive upon publication. |
| Reviewer 1 | I reviewed the manuscript titled “Understory plants reduce light loss in a temperate deciduous forest amid climate variability by shifting phenology in synchrony with canopy trees” for the journal PLOS ONE. The authors of this paper investigated four different factors that could limit understory plant access to spring light, a critical limiting factor for the growth, survival, and reproduction of various understory species. The authors found that the majority of understory plant species were able to track light availability with their phenology, suggesting that most species should be capable of maintaining access to this critical resource, with a small minority of species predicted to lose substantial access to spring light under warmer spring conditions.  I want to start off by congratulating the authors on writing a paper that was surprisingly fun to read given how methods-intensive it was. This is the type of research where it really directly translates how I (and I hope others) tend to think about ecology in all its complicatedness in an effort to get meaningful insight from a messy web of information. I was also impressed by the dataset itself, novel in its phylogenetic breadth of species studied, especially considering the nearly 3 decades that data were recorded over. Overall, however, I did have some concerns related to the writing and analysis. | Thank you for taking the time to thoroughly review our manuscript. We realize it is not exactly a short text, and appreciate both your detailed insights and the positive approach you have taken to this review. |
| Reviewer 1 | Starting with the writing, I wanted much more discussion than was provided. You have just under 7 pages devoted to results and discussion compared to the more than 21 pages of methods. While I appreciate that the methods section is necessarily long to adequately address all of the assumptions and statistical nuances employed in your analysis (a benefit to the paper, in my opinion), I would like to see more discussion on how your many assumptions could have affected your overall findings. This is particularly important because I felt some of your assumptions were overly simplistic (see analysis comments starting in next paragraph), and could definitely have skewed your results in various ways that should be addressed. On a less important note, I point out in my line comments several places where you seem to have overlooked various papers that do in fact address some of the knowledge gaps presented in the introduction. | First, we want to express our appreciation for understanding that the very long methods section was probably the best way to present our work. We had initially tried organizing the manuscript so that the bulk of the detail on methods was in a supplement, but could never figure out a way to do this in a way that we found satisfying. So yes, our discussion and results sections ended up way shorter than the methods, which is how we feel it should be under the circumstances. You and other reviewers do however bring up many points which we agree are important and we now discuss them in our Discussion section. We address these points in later comments as they come up.  Regarding analytical assumptions, we agree that there are a lot, and we did struggle with an unavoidable tradeoff between making these assumptions and making the analysis even more complex than it already is. Thus, the present form of the analysis reflects our best judgment on how to balance these two conflicting values, and we will address specific points where you make them below.  We have incorporated the references that you mention into our revised introduction. We don’t mean to downplay the importance of these publications’ (and many others - too many to fully review!) contributions to reducing the research gaps that we outline in our introduction. However, our perspective is that the unique combination of multi-decade phenological data, coverage of all important canopy species in a tree community, and availability of key ancillary datasets allows us to address these gaps in a more comprehensive way than any other study we are aware of has so far been able to achieve. We have revised the introduction in a way that we hope makes this point clearer. |
| Reviewer 1 | In terms of analysis, I want to first say that I appreciate the overall approach that you take in this paper. It seems to me that you started with a list of potential limitations to understory light availability and then pieced together an extensive analysis that would allow you to address all of them within the same framework – very ambitious and, in my opinion, a very worthwhile attempt at it. And while the amount of information was a strength of this paper, I found that the number and quality of assumptions needed to leverage this information was simultaneously a weakness. | We are happy that you appreciate the overall goals and approach of our analysis. We agree that there are some important assumptions made in this process, and we have tried not to shy away from them in our manuscript. Your point is well taken that they need to be addressed head on. We have expanded treatment of these assumptions in our revised manuscript. The Discussion section now contains several paragraphs on assumptions, and these are also addressed in the Methods section where appropriate. Our point-by-point responses to your specific comments can be found below. |
| Reviewer 1 | Throughout the paper, you make clear that you are only addressing the light interception by understory plant species, stopping short of extrapolating out to carbon gain, plant performance, etc. You even go so far as to state on L551 that “We recommend interpreting differences among species in the relative sizes of the limiting factors cautiously. They can be used to indicate the variability, and thus relative importance of the different factors to individual species, but, due to species’ physiological differences, should not be seen as direct indicators of downstream consequences such as carbon sequestration.” While I think you are correct in this statement, I also think that this statement takes a lot of importance away from this research. The reason why light availability is important is because it directly affects carbon sequestration, so there is really no way to address this topic without coming back to carbon in the end. For me, I wanted to see more in the discussion extrapolating out to carbon dynamics and I wanted to see more in the methods addressing how some of your simplifying assumptions are not realistic in terms of how plants in this system assimilate carbon. | We completely agree that light availability is only an intermediate link in the causal chain from phenology to plant reproductive success. You correctly point out that carbon assimilation is closer to the end of this chain, although it is something that we didn’t directly evaluate. Based on your comments below, we now do carbon calculations for a few species with available data – see our revised text and responses below.  We also agree that the text you quote is probably unnecessarily cautious, especially for appearing in the methods section, and particularly since we have now added some calculations that address carbon consequences with data rather than just speculation. For this reason, we have deleted this sentence in favor of more discussion of these and related topics in the Discussion section.  We have updated various points in the manuscript to better position our work in the broader context that you outline here. Even though we cannot conclusively determine what growth and reproductive consequences changing light limitation will have for understory plants, we are now a bit less limited due to including some carbon calculations thanks to your suggestion. These additional results add value to our manuscript by reaching later (but still intermediate) stages of understanding about the impact of changing phenology on plants’ growth and demography. |
| Reviewer 1 | One example of this is that many understory plant species (particularly those that maintain their leaves into summer) adjust their photosynthetic machinery in ways that allow them to maintain positive carbon gain despite extremely low light levels. In fact, many understory plant species (woody and herbaceous both) have been shown to maintain positive carbon gain throughout much of summer despite full canopy shading (Heberling et al., 2019; Lee & Ibáñez, 2021b, 2021a), indicating that that there is no neat cutoff at which point light becomes limiting. Furthermore, even if there were such a cutoff, it would differ substantially among species – light compensation points (the light level at which carbon lost to respiration equals the carbon gained from photosynthesis) differ drastically among species and are considered a major component of shade tolerance characteristics for understory plant species. Thus, even if you do not want to express overall results in terms of carbon budgets, you should still account for these dynamics in the assumptions of your model. | We fully agree with this critique. Our analysis does account for non-zero summer light levels in the understory, something which we do have direct data on from the I/Io measurements. However, as you allude here, what is effectively zero light from a carbon assimilation perspective depends on the whole-plant compensation point, something that is notoriously hard to measure, and as you rightly point out, is very likely species-specific and a moving target that is dynamically readjusted to a changing light (and also temperature) environment. We now address this more directly in the Discussion section.  After much calculation and communication with authors of two papers that you mention here, we were able to add gross carbon uptake calculations for four herb species (including two leaf cohorts of one of them, so we refer to five species-cohort combinations in our manuscript text). Unfortunately, for reasons we outline in our revised manuscript, we weren’t able to include the *Acer saccharum* calculations from the Lee & Ibáñez paper you point out because we were missing some key environmental variables used in that paper’s models. We decided not to include net photosynthesis results as we are convinced that respiration measurements at approximately monthly intervals are not nearly at a fine enough scale to capture complex environmentally mediated changes in this variable. This point is now mentioned in the Methods and Discussion sections of our revised manuscript. |
| Reviewer 1 | A really good example of why this important is from one of the papers you missed in your intro. Lee & Ibáñez (2021a) found that warmer springs would lead to greater understory light access for temperate deciduous tree seedlings, which on its face would suggest greater carbon gain in future decades. However, after accounting for photosynthetic rates, and especially after accounting for how respiration responds much more strongly to increases in temperature compared to photosynthetic carbon assimilation, those authors found that seedlings would lose proportionally more carbon from summer respiration than from spring carbon gain. Thus, light availability only communicates part of the overall picture and can be largely meaningless without the photosynthetic information to support it. | We now cite this paper in the introduction (see above) and include similar calculations from the overlapping herb species from the Heberling et al. paper where we had sufficient data to do those calculations.  We agree heartily that respiration is a huge issue in understory plants’ carbon budgets. In fact, we feel so strongly about this that we are uncomfortable adding net photosynthesis calculations to our manuscript, so instead report gross photosynthesis. As we have noted above, respiration is too variable, with a highly path-dependent temperature sensitivity, to realistically include in these calculations, even with the substantial published dataset of Heberling et al. that we had at our disposal. This point is now reiterated at several places in our manuscript, especially in the Discussion section. |
| Reviewer 1 | I mention all this because of your emphasis on potentially mitigating effects of warmer temperatures. Warmer temperatures can just as easily exacerbate negative effects of shading or reduce the positive effects of increased light availability. I did not feel like your characterization of temperature as mitigating painted a full picture, especially since you invoke photosynthetic limitations without including photosynthesis in your analysis. | Great point! We completely agree that this was an oversight in our original manuscript, and it is even more important now that we include some carbon assimilation calculations. In the Discussion section we now say that “The role of respiration in this process should not be underestimated; although warmer temperatures may reduce photosynthetic limitations, they also increase respiration in complex ways.” We also include similar language in the Methods section where we justify our choice of gross rather than net photosynthesis calculations. |
| Reviewer 1 | If you wanted to, photosynthetic information is published and available for several of the species you use in this analysis. For example, Heberling et al. (2019) addresses photosynthetic carbon gain and how it is affected by access to spring light in several understory herbaceous species. Even if this information is not available for each of the 40-odd species in your analysis, I think your analysis would benefit from picking even just one or two species as case studies to show that light availability is meaningful. In other words, you address four limitations related to light interception, but the fifth, implied assumption is that plants are using that intercepted light for positive carbon assimilation, which is not necessarily the case. To paint a complete picture of how light availability changes and of why that is important, I think you need to at least partially address photosynthesis and carbon assimilation. | We have followed this suggestion and now include gross photosynthesis calculations for the four species in common with Heberling et al.’s (2019) publication. Please see also our responses above and revised text for an explanation of why we chose not to include calculations for net carbon uptake.  These calculations now inform our revised Discussion section. |
| Reviewer 1 | Another example, not to beat a dead horse, is that many plants respire much more than they photosynthesize during leaf expansion, meaning that having leaves is not enough to be considered photosynthetically useful. However, the duration of leaf expansion is changing with climate change on a species by species basis (Buonaiuto & Wolkovich, 2021), meaning that some species, if their duration of leaf expansion is growing, may actually be losing days of activity under high light availability, even if the amount of light they are exposed to is increasing. Again, the fifth limitation that you are missing in this analysis is photosynthetic activity. You can’t go back in time and collect this data, but you can (at best) try to quantitatively address this by using others’ data collected on the same species or (at worst) address the importance of these assumptions and how they affect your interpretation of the results in discussion. | These are important points. To address a specific aspect of the carbon-phenology relationship that you brought up, we note that the idea that “many plants respire much more than they photosynthesize during leaf expansion” is far from clear or universally supported. See for example: “Keel, S. G., & Schädel, C. (2010). Expanding leaves of mature deciduous forest trees rapidly become autotrophic. Tree physiology, 30(10), 1253-1259.”  On the issue of changing leaf-expansion duration, while this is not something that we explicitly mention, our observational dataset includes starting and ending dates of the expansion process for herbs, and more detailed information for saplings, so any year-to-year changes in this factor, whether there is a systematic long-term trend or not, are baked into our analysis.  However, as we mentioned in the previous and other earlier comments, we decided to include carbon assimilation calculations for a few overlapping species, and this added information strengthens our discussion of just what inferences can be drawn from our analyses.  We now also directly address the issue of photosynthesis/respiration by expanding leaves in the introduction: “Studies show mixed results on the relative importance of photosynthesis and respiration occurring during leaf expansion of trees.” This text is followed by a citation of both the Buonaiuto & Wolkovich (2021) paper you mention as well as the Keel & Schädel publication that we mentioned above. |
| Reviewer 1 | I’ll finish by acknowledging that I just did the thing that I hate when reviewers do to me, which is to make the entire review about something that is beyond or other than the explicitly stated goal of the paper. I get it, and I apologize (to a certain extent) for doing so. However, I really do think that you cannot tell the full story on overstory/understory phenological mismatch without addressing photosynthetic carbon assimilation. Even if your main conclusions (related to changes in understory plant light exposure) stand on their own, which I think they do for the most part, I think it is important and necessary that you fully address the potential implications for plant performance mediated through carbon assimilation. I have added line comments below. | Thank you for acknowledging this! It is natural that reviewers will focus on the aspects of a manuscript that they know best. Even if it is not precisely the same as what we are studying, your perspective has contributed valuable insights that we think have made our manuscript useful to a wider audience.  As we have noted in our above responses, we now include photosynthetic calculations for a few species and develop the issue of the relationship between light, carbon, growth and reproduction more fully in the discussion section. We still don’t think it is the full story, but at least it is a bit more of the story now. |
| Reviewer 1 | L58-66: Authors could add references for similar dynamics in juvenile tree seedlings (Lee & Ibáñez, 2021a, 2021b). This would be especially helpful in linking spring light access to growth and survival of woody plants. | Thank you for this suggestion. We now include citations of these papers in the first and second paragraphs of the introduction, as well as a few other places later in the manuscript |
| Reviewer 1 | L94-95: See (Lee & Ibáñez, 2021b, 2021a) for papers that address entire growing season dynamics for two species of tree seedlings. | Thank you. As noted above we now cite these papers at several points. |
| Reviewer 1 | L114-118: Again, see papers by Lee and Ibanez for examples of comparing understory phenology directly to observed light levels. | These suggested references have been added to this paragraph. |
| Reviewer 1 | L127-131: Again, see papers by Lee and Ibanez for existing examples of what you cite as not yet existing (regional studies; (at least partially) accounting for differences in canopy composition; etc.) | Our text in this section now reads: “Studies at the local level ideally take into account tree species composition above individual understory plants, because variation in phenology among canopy tree species affects understory phenology, growth and reproduction [Lee + Ibáñez 2021b].” |
| Reviewer 1 | Paragraph beginning L166: All data should include units of measurement, especially since Fig. 1 is pretty low resolution and even zooming in does not allow for the reader to easily interpret the units shown on the axes. | The units of measurement have been added to the sections of the Methods that describe the external sources of data or how primary data was collected in the forest. Note that we do not yet introduce the units in the second paragraph that gives the broad overview as we wish to keep this section as simple as possible. We haven’t included these units in Figure 1 since, as you note, there is already quite a lot there. We mean for it to be a broad overview, and for readers interested in details to refer to the corresponding sections of the Methods text. |
| Reviewer 1 | L170: “Government agencies” could be a bit more specific, even if it is listed later in the methods as well. | This sentence now includes the text: “Environmental data on (1e) temperature and (1f) solar radiation came from the National Weather Service and NOAA Surface Radiation Monitoring (SURFRAD), respectively.” |
| Reviewer 1 | Section 2b (beginning L364): It is unclear to me, as currently written, at which spatial resolution basal area was aggregated. Did you include all trees in the 50m x 50m plot? Or did you account for trees that were within a certain distance of the point measurements only? Given the difference in average leaf out times among canopy tree species (e.g., cherry and maple are much earlier than oaks), it makes sense to me to try to pick a distance around a quadrat and include only the trees with basal area overlapping or within that set distance. Is this possible? If not, can you explain more in the text about how the assumptions you make here could affect the analysis? | The description in Methods section 1d has been clarified to now read “Species’ basal areas (cm^2^), aggregated to per-species proportions of total study-forest-wide basal area, were calculated for stems larger than...”  Late in Methods section 2b we now say “These values were aggregated to the community-level canopy light interception by leaves (*L_c_*) by taking means weighted by each species’ proportional study-site-level basal area (see Section 1d).” (The underlined text was added to clarify this point.)  Unfortunately, geolocated positions of each censused tree in 2005 were not available to use only trees within a set distance from each census tree, a point we now mention in the discussion. |
| Reviewer 1 | L523-527: You focus here on cold-limitations to photosynthesis, but temperature effects on photosynthesis are much more complicated. For example, positive carbon gain is impossible at high temperatures because respiration costs scale more steeply with increasing temperatures than does carbon assimilation. Temperature also interacts with light availability for similar reasons – darker conditions require lower temperatures in order for positive carbon assimilation (which only occurs with high-enough light availability) to outweigh negative respiration costs (which are an issue all day long, including overnight when light is never available). Freezing limitations are good to include, but you are potentially under-counting the capacity for photosynthesis to be limited by other aspects of temperature, which I think could potentially lead to an underestimate in how important temperature is as an overall limitation. | Again, we fully agree with this point. In fact, it is exactly what led us to report gross rather than net photosynthesis in the carbon uptake calculations for the four herb species with available photosynthetic data. We now work this information in to the revised version of our Discussion, and it is also mentioned in the Methods. |
| Reviewer 1 | L528-36: Species differ significantly in how much light is needed to conduct positive photosynthesis (often referred to as the light compensation point). In this sense, a canopy transmittance value of ~0.1 (in your analytical framework) may be entirely limiting for one species, but not at all limiting for another, even under conditions that are otherwise exactly the same. This is, importantly, a large component of what is considered when researchers quantify shade tolerance among different species. Furthermore, it often changes throughout the growing season in species whose leaves are maintained for longer than a few months. Species-level photosynthetic efficiency information is missing from this estimate of canopy light transmittance limitations and could lead to biased estimates of how much of a limitation this variable causes. Specifically, this limitation would be over-emphasized for shade-tolerant species (which can maintain photosynthetic efficiency even in full shade) and under-emphasized for shade-intolerant species for which even a small decrease in understory light availability could result in complete loss of positive carbon gain. I understand that your goal for this paper is not to extrapolate all the way to carbon budgets, but this is a major (and in my experience, incorrect) assumption that all of these species are equally light-limited. If possible (especially if available from elsewhere in the literature), species-specific photosynthetic efficiencies should be incorporated to account for light limitations. If that is not possible, the implications from this assumption should be clarified in the text. | Thanks for bringing up this point. It is indisputably true, but it was not as present in our minds as it probably should have been when we wrote the initial version of our manuscript. As you note at various points, the compensation point of plants changes seasonally and as a function of temperature. We view the temperature dependence of respiration to be even more complex than you have described it here (see references mentioned above). In our view, this makes clear inference on the net carbon assimilation consequences of changes in understory light levels nearly impossible without much more extensive (and species specific) data on seasonality and temperature dependence of whole-plant compensation points. Our revised Discussion section now includes several additional paragraphs on assumptions, including on this issue.  In addition, the newly-included photosynthesis calculations on a few of the herb species are at least a step in the direction of addressing this point. |
| Reviewer 1 | L551-555: You write “We recommend interpreting differences among species in the relative sizes of the limiting factors cautiously. They can be used to indicate the variability, and thus relative importance of the different factors to individual species, but, due to species’ physiological differences, should not be seen as direct indicators of downstream consequences such as carbon sequestration.” This seems to contradict some of your earlier justification for why this research is novel and important. Photosynthetic parameters are likely available for some of these species from other manuscripts (see, for example, Heberling et al., 2019). Would it be possible to extrapolate these “downstream consequences” for at least a few select species where this information is available? For me, it would go a long way in justifying the rest of this very complicated approach. | We don’t mean to downplay the novelty of our research by saying this, but simply want readers to be fully aware of what we can and cannot do with the available data. As indicated several times above, we now include gross photosynthesis calculations for several of the herb species, and these results show broadly similar patterns to the corresponding light results. Of course, even the gross photosynthesis calculations are still a few steps away from plant demographic outcomes, but we are happy that you have encouraged us to take the first step in that direction. We hope that future researchers with more physiological approaches are able to build on this work and take it even further. Our revised manuscript now reflects this thinking by including these carbon assimilation calculations and discussion of why they still are not a complete answer to what will happen to understory plant populations as a result of phenological shifts. |
| Reviewer1 | L696-697: This holds true only in winter, whereas the opposite is generally true in summer. | This characterization of canopy vs. understory temperature was made implicitly considering the times when there is most light in deciduous forest understories, namely the winter and early spring. You are clearly right that it is not generally true in the summer. We have reworded to “Air at canopy level is generally colder than air in the understory during the winter and early spring when temperate deciduous forest understories are brightest…” |
| Reviewer 1 | L728-730: Isn’t it more likely that this positive association comes from higher temperatures on non-cloudy days? Meaning that past springs with fewer clouds are likely to be warmer, but not necessarily that warmer spring temperatures will reduce future cloudiness. | Yes, certainly! We didn’t mean to imply causation in any direction, and as non-meteorologists we don’t want to wade into that issue too deeply. The re-worded sentence now ends with “... warmer springs may have been less cloudy over the data-collection period.” |
| Reviewer 1 | L741-742: This compensation may not be valid considering you are not accounting for high temperature limitations to photosynthetic efficiency (see Lee & Ibáñez, 2021a ) for an example of how high temps are exacerbating, not ameliorating to overall carbon dynamics in warmer summers). | You are certainly correct that higher temperatures are not necessarily better for plants in all circumstances; see multiple points above. However, in the context of our analysis, we believe our text that you cite is correct. As mentioned above, we now include discussion of the issues of temperature dependence of respiration and how that affects whole-plant compensation points so that they become complex moving targets. |
| Reviewer 1 | Papers Cited:  Buonaiuto, D. M., & Wolkovich, E. M. (2021). Differences between flower and leaf phenological responses to environmental variation drive shifts in spring phenological sequences of temperate woody plants. Journal of Ecology, 109(8), 2922–2933. https://doi.org/10.1111/1365-2745.13708  Heberling, J. M., Cassidy, S. T., Fridley, J. D., & Kalisz, S. (2019). Carbon gain phenologies of spring-flowering perennials in a deciduous forest indicate a novel niche for a widespread invader. New Phytologist, 221(2), 778–788. https://doi.org/10.1111/nph.15404  Lee, B. R., & Ibáñez, I. (2021a). Improved phenological escape can help temperate tree seedlings maintain demographic performance under climate change conditions. Global Change Biology, gcb.15678. https://doi.org/10.1111/gcb.15678  L(2021b)ee, B. R., & Ibáñez, I. . Spring phenological escape is critical for the survival of temperate tree seedlings. Functional Ecology, 1365-2435.13821. https://doi.org/10.1111/1365-2435.13821 | Thanks! As noted above in response to multiple specific queries, we now make liberal reference to all of these publications in our manuscript. |
| Reviewer 2 | The study examines whether phenological shifts in 31 herbs respond to four factors that could limit their light interception. This adds to the growing literature investigating how canopy phenology might influence understory herbs. I really appreciated the time spent on why a local study addresses key questions about understory-canopy mismatch that a broad synthesis cannot, as well as the step by step modelling decisions. | Thank you for your review of our manuscript and appreciating our goals in writing it! |
| Reviewer 2 | However, the methods section is extremely difficult to read. I include specific questions below, but overall recommend some simplifying/summarizing statements at the beginning/end of each sub-section (or if the bulk of the text could be simplified and the details moved to an appendix). I could also use some discussion on the collinearity of a lot of these measurements, which are subsequently multiplied together, as well as clearer figures (improved Figure 1 and some presentation of the differences in slopes). | We are fully aware that the methods section is not simple, and appreciate your effort to grapple with it and give suggestions on how to make it more user friendly.  We have implemented your suggestion to include simplifying (or really guiding) statements to begin each section of the methods, with the exception of the subsections in Section 1 on data sources. We feel that the Section 1 subheadings are relatively self-explanatory, but we are open to suggestions if you can think of ways to improve them.  We had initially tried to organize the manuscript with most of the methods in an appendix, but could never find a way to do it that was more satisfying and less confusing than what we finally settled on. We readily concede that the methods are complex, but argue that this complexity is necessary to achieve the goals of the manuscript.  Some of the variables we work with, for example leaf area and sunlight, are certainly collinear. However, as we are not using these as supposedly independent predictor variables in a linear model or something similar, this does not present a fundamental problem to our analysis. All of these variables are independently measured and enter our calculations that way.  We have made various improvements to Figure 1: most arrows are now straight and color-coded based on where they come from. The subpanels are now aligned except for the final one which is larger than the others to emphasize its importance. The final panel also shows spring temperature on its x-axis rather than year, emphasizing the importance of this perspective on the results. See below for more details on these changes. |
| Reviewer 2 | Abstract: It’s a little difficult to follow negative/positive shifts, “beneficial” shifts (especially given there’s no fitness measurement), and synchronous shifts. It would be worth setting up, even in the Abstract, which direction phenology needs to be shifting in order to be synchronized with light, and then use that term to describe the results. | Thanks for this constructive suggestion. In the abstract we have dropped use of ‘beneficial’ and ‘synchronous’ shifts in favor of saying things like ‘in the same direction’ for synchronous. We now define ‘negatively’ as meaning reduced understory light interception and  ‘positively’ as increased understory light interception. So, for instance, understory light interception shows a positive change if understory phenology shifts earlier than canopy in spring and/or later than canopy in autumn. |
| Reviewer 2 | Introduction: L57: Add citation. | Lee, B. R., & Ibáñez, I. (2021a), Lee, B. R., & Ibáñez, I. (2021b), and Heberling et al. (2019) are now cited here. |
| Reviewer 2 | L77: Air temperatures for herbs as well? | We have clarified this to read “Herbs first require warmer soil temperatures to break dormancy of underground structures (8) ; secondarily and later after emergence, they respond to warming air temperatures.“ |
| Reviewer 2 | L137: While I understand that this last paragraph of the Introduction is further outlining how this study differs from previous work, it feels very much like a methods section. Suggest rewriting to focus on hypotheses – what directions of sensitivities do we expect to see? What should we see if the slopes are mismatched or not? The methods details here should be integrated elsewhere. | The last paragraph of the Introduction has been revised, eliminating details of methods and uniqueness of study. Instead, it focuses on the study’s emphasis on evaluation of light availability to understory plants, as limited by canopy and understory phenology, temperature, and solar radiation. Requirements needed to demonstrate a mismatch detrimental to understory species are spelled out in newly created Table 1, in general and as a function of warmer temperatures. |
| Reviewer 2 | Methods L230: Can you explain this cohort grouping more clearly? What does it mean biologically? Did it make a difference when analyzing species/cohort vs. species? | The cohorts represented 1st year vs. 2nd year individuals of a biennial species and early-season leaves vs. a much later, completely new set of late-season leaves of a perennial species. Cohorts of all species that had distinct phenologies were analyzed separately. The text was altered to:  “Herbs of 33 species were observed, including two distinct cohorts of leaves in perennial species or year 1- vs year 2-individuals in a biennial species, for a total of 37 species-cohort combinations. As the cohorts within a species had contrasting phenologies, they were analyzed separately; we hereafter refer to these combinations as “species” for simplicity.” |
| Reviewer 2 | L277: What was the date choice for light interception here? Was this the same value for all species in the study? Or were different Julian calendar dates for temp limitation used for each species based on some mean leaf-out time? Or is this that every Julian day of the weekly observation had this 4-step process applied? | We aren’t completely clear what you mean by “date choice for light interception” here, but we think it might refer to whether a day’s temperature was suitable for photosynthesis, which is what the section said where L277 fell in our original manuscript. The same value was used for all species. Of course, for any plant that had not grown leaves yet, the assignment of a day as having sufficient or insufficient temperatures for photosynthesis is completely irrelevant. This is a consequence of the hierarchical relationship among the four limiting variables that we explain in the 4^th^ paragraph of Methods section 5. But for the purposes of calculations, we had to make this decision for each day in the dataset, from 1995-2022.  Again, we are not totally certain what you mean by “every Julian day of the weekly observation.” This 4-step process was applied to every day from 1995-2022. This is completely independent of the mostly ~weekly phenological observations". |
| Reviewer 2 | L349: Is the caption for Fig. 2 supposed to be floating here? | We agree that this is a bit unusual, but the PLoS One instructions say that “each figure caption should appear directly after the paragraph in which they are first cited.” See: https://journals.plos.org/plosone/s/file?id=wjVg/PLOSOne_formatting_sample_main_body.pdf |
| Reviewer 2 | L374: How was tree phenological stage converted to transmittance value? | This is explained in detail in the paragraphs following this one. To guide readers that this will soon be explained, we have added a parenthetical note to the sentence so it now reads “To do this, we first calculated the species-level phenological stage by converting each tree’s phenological stage to a transmittance value (see following paragraphs for explanation of this process) and averaging these.” |
| Reviewer 2 | L395: Optimal values for what? Interception or transmission? | Thanks for pointing out this ambiguity. We have reworded so it now reads “The unknown values of E1 … F3 are a six-dimensional optimization problem, so finding precise values that optimize the fit between observed and estimated canopy-level light interception for each stage is computationally prohibitive.” |
| Reviewer 2 | Section 2b is a little hard to follow. Perhaps some summary at the beginning about estimating transmission, then interception, then something about adjusting for shading values? | We have reorganized the first half of the first paragraph of Section 2b. It now reads “In this section we explain how the seasonally changing percentage of light intercepted by canopy leaves was calculated (the thick green line in Figure 2). Canopy light transmission considering leaf phenology must be modeled with respect to the trunk and branch light-interception model derived in the previous section. This separate leaf interception model is necessary because canopy transmission measurements were made only on selected dates during 2002…”  Also, thanks to your comment here, we have realized that we did not really explain what was meant by “shading values.” This is simply how much light is intercepted by leaves at a particular phenological stage. Since we mostly use terminology of “light interception,” we have replaced all mention of “shading values” with “light-interception values” or something similar. |
| Reviewer 2 | L407: What does this variance by stage-specific shading do, relative to the equal spacing in the paragraph prior? | The equal spacing is the simplest assumption we could think of as a starting point for the optimization. Therefore, the random variation in the next paragraph (the original manuscript’s L407 that you mention) begins from this point of equal spacing in the first of the 10,000 iterations, but it does not stay there for long as the algorithm iterates toward increasingly better estimates of each stage’s contribution to light interception. We now word the section where the equal spacing occurs as follows: “First, each stage was assigned an initial light-interception value between 0 and 1 with equal spacing among stages, so E1=1/6, E2=2/6 … F3=6/6, chosen as an easy-to-calculate starting point for the iterative optimization (see following paragraph) that conforms to the three rules listed above.” Note that this sentence now contains the wording “light-interception value” to make the link to the randomly varying jumps in the next paragraph explicit. |
| Reviewer 2 | L456: Isn’t this leaf area measurement completely colinear with phenology given how it was estimated? This needs more justification. | Yes, the phenological observations and calculated leaf area are absolutely collinear, which is a feature of our analysis, not a bug! Our goal in Section 2b of the calculations was to turn the ordinal phenological observations into numerical values relating to canopy light interception. We then use these stage-specific estimates as a basis for calculating how much light saplings are able to intercept based on the percentage of their summer maximum leaf display. Your comment has made us realize that our use of the word “area” here is perhaps a bit misleading. We did this because seasonal changes in light interception by plants is primarily a function of displayed leaf area. Obviously, the density of chlorophyll and other pigments in the expanding leaves is an important secondary factor on which we don’t have direct data. However, our estimates of phenological-stage specific light interception do implicitly take such changes into account – we simply have no way of explicitly separating leaf size from pigmentation, and this is not needed for the purposes of our analysis. This section of the text now reads: “We compute these values on a relative scale of 0 to 1 because total leaf areas were impractical to measure, and our ultimate interest is in understanding the relative impact of phenological shifts on species’ light interception, not leaf area as such. Therefore, although we refer to this variable as “leaf area” for the sake of simplicity, it also encompasses other aspects of leaf function, namely the amount of light-absorbing pigment present. Because the canopy calculations estimate overall light interception, they encompass both of these processes. That saplings follow a similar light-interception trajectory to canopy trees is an assumption that we make at this point, whose implications we address in the discussion section.” |
| Reviewer 2 | L461: But this measure of interception was not measured in 2b? Why? What’s the difference? | Leaf senescence and leaf drop are two separate and semi-independent processes, something that we discuss in the last part of Methods Section 2b on autumn light interception calculations. There we note that we initially tried to include senescence in the model, but it didn’t make any difference so was eliminated to streamline the calculations: “We first tried modeling this process as the product of two separate processes, leaf coloration (senescence) and leaf drop. The logic was that the amount of light intercepted by the canopy is proportional to how many leaves are left, and also that the light blocked by leaves depended on the extent they had senesced. However, our initial attempts to fit stage-specific parameters indicated that model fit was best when senescence led to essentially no reduction in light-blocking by leaves. For this reason, we simplified the model to include only the leaf-drop process.” This means that, unlike in spring, the canopy light interception modeling process gives us no information about photosynthetically-relevant light interception, so we have to to make some sort of assumption about how this process works in the autumn. We now address the consequences of this assumption in the expanded Discussion section. |
| Reviewer 2 | L523: Why not use continuous temperature from the weather station? Temperature is likely still affecting phenology so this grouping doesn’t make much sense. | The challenge in using continuous temperature is that parameterizing such a model would involve extensive physiological investigations that were well beyond our capacity to undertake, and which would have been at least one entire additional manuscript worth of results. Further, we don’t claim to be addressing all aspects of how temperature affects photosynthesis – just a simplistic look at how low temperatures do so. For discussion of respiration and how it is influenced by increasingly warm temperatures, please see our responses to several of the comments made by Reviewer 1 above, our discussion of this point in the Discussion section, and also our explanation of why we include gross, and not net, photosynthesis calculations in our additional carbon assimilation calculations. |
| Reviewer 2 | L598: Why is more variable == more limiting? Why not a mean low value? | A low mean value would be misleading in that, from the way these calculations were done, the long-term averages of all of these factors are near zero, although not necessarily identical to zero due to their hierarchical relationships to one another (i.e. differences in shading by the canopy are irrelevant to a sapling with no leaves). Thus, the positive years tend to balance out the negative years in the long run. Therefore, the mean value alone is not really informative. We use the standard deviation as a measure of variability since it has the same units (unlike variance) as the calculated annual values for these limiting factors. We have added a sentence clarifying this to the first paragraph of Section 6 of the Methods reading “Note that we do not simply use the mean values of these factors because the partitioning process described in Methods Section 5 leads to them having long-term averages near zero (the deviation from exactly zero is due to the hierarchical relationship among the factors).” |
| Reviewer 2 | L617: I agree with this logic, but does the date chosen vary for each species? | No. We don’t have any way of determining a species-specific value for most of the species. It could be done for the three sapling species (based on those species’ canopy trees), but not any of the herb species. Since the herb species all have to use the same value, we eliminate the complexity of having separate values for the three sapling species. We now clarify this immediately following the text that you mention: “Since these values are calculated for the canopy as a whole, we do not have separate calculations for the different understory species.” |
| Reviewer 2 | L619: chosen how? | We have reworded this sentence to try to make it clearer that this sentence is the explanation of how these values were chosen. The text now reads: “We use the date on which the 48-day running-average temperature first exceeds 13° C as our measure of spring temperature. The values of 48 days and 13° C give the lowest root-mean-square error (RMSE) between predicted and observed dates on which the Trelease Woods canopy reached 95% closure.” |
| Reviewer 2 | L634: How would temperature itself be “limiting”, especially if it’s measured as a grouping of 0-5 or >5 (L523)? Would this be better calculated as growing degree days to get at heat limitation? | I’m not sure we are talking about the same thing here, but we will attempt to understand the implications of this comment. Growing degree days can be a measure of how well-suited temperatures are to plant growth and production, especially in agricultural contexts. Is the idea to use something like that as a measure of how well-suited ambient temperatures are to growth of understory plants in our study site, and then incorporate those numbers into our process for estimating limitations caused by different factors? The problem arises that respiration is a big piece of the net photosynthetic equation, and it is strongly influenced by temperature in a way that is not a simple one-to-one relationship, but is highly dependent on acclimation to short-term changes in ambient temperatures. This is a big issue that came up in response to Reviewer 1’s queries – see our responses to those questions for more detail if you are interested. So, to summarize, this is a real issue that you bring up, but there is no way of adequately addressing it without extremely extensive physiological studies, and low-temperature limitation is the one aspect of this that we think we can treat sufficiently well. |
| Reviewer 2 | L648-664: Please rewrite in terms of what’s happening. Negative trend, when it’s unclear whether we’re thinking of the slopes as advances in phenology or decreased light interception, is not informative because the predictor variables are muddled. The last paragraph of the Results (L665) is the most clear. | Thanks for this idea! We have reworded so that “positive” and “negative” are clarified to “more light interception” and “less light interception” or other similar wordings. |
| Reviewer 2 | L696: How is this conclusion about temperature drawn? | Reviewer 1 also pointed out that this was not entirely correct; we had been thinking about only certain times of the year when writing this sentence. It is now reworded to “Air at canopy level is generally colder than air in the understory during the winter and early spring when temperate deciduous forest understories are brightest, and soil temperature changes are lagging and muted responses to air temperature. “ |
| Reviewer 2 | Figure 1: Is chaotic. Perhaps even just straight arrows would help. “Leaf status” not used in main text. I was expecting some figure showing differences/mismatches in slopes based on the abstract, or a figure in relation to spring temperatures. | We had intended this figure as a graphical overview of the methods, but not a way of communicating the results. As such, we still find it to be effective (imagine trying to understand this method without a figure!), and appreciate the feedback on ways to improve it. In response to this and other comments, the new figure now features straight arrows (except for one that has to go around an obstacle) that are color-coded, based on what data source they come from. The subparts of the figure are now better aligned, except for the final analysis plot (bottom right), which is still larger than the other plots to emphasize its importance. This final panel also now shows light loss/gain as a function of spring temperature rather than year to better connect the ultimate results to a climate change focus. The confusing “Leaf status” label has been changed to “understory leaf area.”  In addition to this figure, we now include a table that outlines how different scenarios of changes in the four limiting factors affect the overall light budgets of understory plants, which we hope helps clarify the different potential outcomes of the mismatch hypothesis. |
| Reviewer 3 | It was my pleasure to review the manuscript titled, “Understory plants reduce light loss in a temperate deciduous forest amid climate variability by shifting phenology in synchrony with canopy trees” by Carol Augspurger and Carl Salk. In this manuscript, the authors integrate several long-term data sets to estimate how light availability for understory plants has changed over time and to weigh the relative impacts of understory plant phenology, canopy phenology, temperature, and solar radiation on this factor. Using their light availability metric as lens to investigate the potential impacts of warming climate and shifting phenology, the authors find that generally little change has occurred in the amount of light available to understory plants, though some species exhibited idiosyncratic responses. In my opinion, the results detailed in this paper are a valuable addition to the literature on climate change impacts on plants. The authors make excellent use of unique, long-term, detailed observational data to track light availability over the span of decades. This manuscript is well-written, and the descriptions of data sources and analytical methods are detailed and clear.  I do have some suggestions for how this manuscript might be improved: | Thank you for your review of our manuscript and for appreciating what we are trying to do with it. We know it was long, and we are particularly grateful that you made the effort to understand its complexities. |
| Reviewer 3 | 1. Figure 1 is difficult to take in. The arrows end up making a tangle that is hard to make sense of, and the axis labels on plots are small and difficult to read. I suggest arrows with right angles might be easier to track. Vertically aligning the plots in the left side “Models” split would also aid readability. The caption describing this figure could also be expanded to add clarity. Walking through panel by panel would be beneficial, I think. | Thank you for your feedback on this figure – the other reviewers had a similar reaction to it, and we have some detailed description of the changes to the figure above in this document. Responding to your specific points, we now have straight arrows (except for one that has to go around another panel) that are color-coded based on where they originate. The panels are now vertically aligned except for the last one showing the results; it is bigger than the others to emphasize its importance.  As for the need to walk through the figure panel by panel, this is done in the second paragraph of the methods; trying to add this to the legend would be a repeat of that section. Our hope is that the typeset version of this eventual article will place this figure near that text. We have expanded the first sentence of the legend to call readers’ attention to the paragraph where the broad picture of the methods is explained: “A conceptual diagram of this study's analysis to complement the overview given in the second paragraph of the Methods.” |
| Reviewer 3 | 2. I think reader’s understanding of the paper’s results might be improved with a figure or table that walks through expected patterns in parameters under several possible scenarios. The authors should consider including a table with columns for: “Changes in canopy spring phenology”, “Changes in understory spring phenology”, “Changes in canopy autumn phenology”, “Changes in understory autumn phenology”, and for changes in light availability and each of the potential drivers of light availability under these scenarios. With each row representing a different scenario, the authors could indicate whether how variables would change given particular set of phenological shifts. Giving readers a tool such as this to think through how the influence of different drivers might be altered under different phenological scenarios could aid in their interpretation of results. Including such a figure/table might also help readers to better understand how solar radiation’s influence might vary from year to year, as key phenological windows hit at different seasonal timings. | Good idea! Ideally, we would like to have made a figure in response to this comment, but it was beyond our graphical design skill levels.  Instead, we have created a table along the lines you suggest. It is introduced in the last paragraph of the introduction where we lay out some of these possible outcomes. We then refer back to these scenarios in the discussion to put our actual results in this context. |
| Reviewer 3  done | 3. I would like to see the plots showing the light availability over the years for each species in addition to the plot showing the breakdown of the potential drivers’ influence over time. These could be included as new supplement. Showing this output would allow readers to clearly see whether there is a trend in light availability over time for the understory species considered here (regardless of which drivers might be influencing that availability). | This information is in the plots in SI 9 and 10, referred to in Results in lines 652-657 of our originally submitted manuscript, but we obviously haven’t made that clear enough! After toying with different ways to make this clearer, we have ended up describing what these supplemental files contain when they are first mentioned in the results.  The first sentence of the key results paragraph now reads “None of the four individual limiting factors separately showed a statistically-significant trend over time for any sapling species (SI 9 Fig; n.b., SI 9 and SI 10 Figs show trends over time and spring temperature, respectively, in all four factors and their combinations individually for each species)” Underlining indicates newly added text. |
| Reviewer 3 | 4. Citations justifying the use of the same temperature thresholds for photosynthesis for all species included in the study should be provided in the paragraph on lines 273 through 289. | We aren’t able to find specific references for all species, and probably some of these have not ever been specifically studied in this context. We now cite a few references on the topic:  41. Regehr DL, Bazzaz FA. Low temperature photosynthesis in successional winter annuals. Ecology. 1976; 57(6):1297-1303.  42. Adams MS. Adaptations of Aplectrum hyemale to the environment: effects of preconditioning temperature on net photosynthesis. Bull Torrey Bot Club. 1970; 97:219-224.  The broad response, however, is that low temperatures caused so little limitation to most species that it is likely unimportant if the thresholds are off by a few degrees. We now address this point in the Discussion section. |
| Reviewer 3 | 5. I am curious if there are other, established practices for partitioning the influence of component variables (U, C, T, R here) to a derived variable (L). While the authors’ method for assessing the importance of sub-factors is described thoroughly, I lack the expertise to evaluate whether their method is entirely appropriate. Citations pointing to other works using similar approaches would be reassuring. Or, alternatively, a brief discussion justifying the need for an apparently novel approach that indicates the shortcomings of existing methods would also help. Because the paper’s conclusions hinge on these values, additional justification for the chosen approach would strengthen readers’ trust in the stated results. | We aren’t aware of any other attempts at or methods for this kind of partitioning, which is why we created this very complex method in the first place. If there were good existing techniques to adopt, we would happily have used them and avoided the effort required to create our own. This is something that we now explicitly state in the second-to-last paragraph of the Introduction: “We are unaware of any previous study that has separately quantified how multiple factors individually affect understory plants’ light availability.”  We are certainly sensitive to the extent that we rely on this new and complicated procedure to partition the various factors limiting understory light availability. This is why we include such a detailed description of them in the main text of our manuscript; earlier versions that relegated most of the methods details to supplements felt unsatisfying to us, as we want to get these processes out in the open so that readers can judge for themselves how believable they find them. |
| Reviewer 3 | I also have a small number of minor edits to suggest: L23: I suggest “solar radiation” in place of “sunlight” | We have made this edit as suggested. |
| Reviewer 3 | L69: I suggest “typically” be inserted before “resulting” to clarify that this is not universally true | Good suggestion. We have added this. |
| Reviewer 3 | L73: Some folks consider different hypotheses, including trophic mismatches, and call them “phenological mismatch hypotheses”. Consider changing or clarifying terminology here. | This has been edited so it now reads “The phenological mismatch hypothesis, when applied to plant strata, addresses…” with the underlined text now included to emphasizes that we are not addressing trophic mismatches, etc. |
| Reviewer 3 | L138: I suggest “Data sources were available…” to maintain past tense. | This sentence was eliminated when responding to some of Reviewer 1’s comments. But overall, this paragraph is entirely present tense now, so at least it is consistent. But we concede that it would make just as much sense entirely in the past tense. |
| Reviewer 3 | L185: I suggest adding a comma after “level”. | Comma added as suggested. |
| Reviewer 3 | L178: I suggest R in place of S for consistency. | We have replaced Irradiance with Radiation and ‘S’ with ‘R.’ Thanks for catching this! |
| Reviewer 3 | L202: What magnification were the binoculars? | We have changed this to “At 7-day intervals during spring and autumn, binoculars (8X magnification) were used …” |
| Reviewer 3 | L433: I suggest “vary” rather than “drift freely” | We have made this change as suggested. |
| Reviewer 3 | L606: I am unclear what “limitation values” are being referred to here. Please clarify what these are. | We have altered this sentence so it reads…’Based on these calculated values for the four limiting factors, we…’ |
| Reviewer 3 | L633: Do you mean “solar radiation (R)” here? | Yes, we changed abbreviations in an earlier draft, but missed this instance. Thanks for catching it. We have changed S to R so it is consistently the abbreviation for “solar radiation.” |
| Reviewer 3 | L703: I suggest softening this statement to, “This study shows which responses are really happening” | This change has been implemented as suggested. |
