# Supplementary material for: Understory plants evade shading in a temperate deciduous forest amid climate variability by shifting phenology in synchrony with canopy trees
Source: PLoS One. 2024 Jun 26;19(6):e0306023. doi: 10.1371/journal.pone.0306023 (PMC11207122; doi:10.1371/journal.pone.0306023)
Supplement: Attachment — Submitted filename: Responses to re-reviews.docx [file pone.0306023.s016.docx]

| **Who** | **Comment** | **Response** |
| --- | --- | --- |
| Editor | Journal Requirements:  Please review your reference list to ensure that it is complete and correct. If you have cited papers that have been retracted, please include the rationale for doing so in the manuscript text, or remove these references and replace them with relevant current references. Any changes to the reference list should be mentioned in the rebuttal letter that accompanies your revised manuscript. If you need to cite a retracted article, indicate the article’s retracted status in the References list and also include a citation and full reference for the retraction notice. | We have double checked all the references we have cited on the journals’ websites where possible, and none are indicated as having been retracted. In a few cases of articles from the 1970s, the articles are now archived on JSTOR rather than the journal-specific website, so we checked for notification of retractions there instead. |
| Editor | Additional Editor Comments:  Dear Carl,  Thank you for submitting the revised manuscript titled "Understory plants evade shading in a temperate deciduous forest amid climate variability by shifting phenology in synchrony with canopy trees" to PLOS ONE.  The reviewers have provided their feedback, noting that the quality of the manuscript has improved substantially. However, a minor revision is necessary to address the reviewers’ comments fully and meet PLOS ONE publication criteria. You can find the reviewers' comments at the bottom of this letter. | Thank you for this round of reviews. We respond to the reviewers’ specific comments below. |
| Reviewer 1 | Thank you for your thoughtful responses to my initial comments. The paper looks great and I have no further comments. | Thank you again for your helpful feedback on our work. |
| Reviewer 3 | Dear editor, Thank you for the opportunity to review the revised manuscript, “Understory plants evade shading in a temperate deciduous forest amid climate variability by shifting phenology in synchrony with canopy trees.” The authors have responded adequately to the suggestions I made in my prior review. Figure 1 has been much improved, and I find the table summarizing some likely scenarios to be a helpful tool for thinking through the interactions between the evaluated drivers of light availability. | Thank you for your additional careful feedback on our manuscript. |
| Reviewer 3 | I did notice a few small line items the might be improved during my review:  L 135: I think you mean “species-specific” here | Thank you for catching this! We have revised as suggested. |
| Reviewer 3 | L 136: I’m not sure “futuristic” is the right word choice. I suggest, “forward-looking” instead. | On revisiting this sentence, we have decided that it was a bit unclear, for this and additional reasons. It now reads “Ultimately, long-term and forward-looking assessments of how changes in mismatches affect plant fitness are desirable.” |
| Reviewer 3 | L 147: This sentence gets confusing. Consider listing the four factors in parentheses to clarify? | We have re-arranged this sentence, including your suggestion, in a way that we hope is clearer. It now reads “We quantify the relative importance of four potentially limiting factors (understory phenology, suitably warm temperature for photosynthesis, canopy phenology, and solar radiation) on light availability for each understory species over more than two decades.” |
| Reviewer 3 | L 167: I think you mean to say the that table does NOT include all possibilities, correct? If so, this needs to be corrected here. | Thank you for catching this – you are correct that we meant to include a “not” there. It has been revised as suggested. |
| Reviewer 3 | L 669: I suggest “left” in place of “leaves” to maintain past tense | We have made this change as suggested, and also made a similar change to the preceding sentence. |
| Reviewer 3 | L 827: Consider “cannot” in place of “can’t” | We have made this change as suggested. |
